# Muscle activity and airflow dynamics upon gradual weight-gain volumetric enlargement or acute surgical reduction of the tongue base during chewing and swallowing

**DOI:** 10.1371/journal.pone.0352976

**Published:** 2026-07-29

**Authors:** Doris Haydee Rosero Salazar, Edward M. Weaver, Zi-Jun Liu

**Affiliations:** 1 Department of Orthodontics, University of Washington, Seattle, United States of America; 2 Department of Pharmaceutical Sciences, Faculty of Engineering, Design, and Applied Sciences, Universidad Icesi, Cali, Colombia; 3 Department of Otolaryngology, University of Washington, Seattle, United States of America; Showa University: Showa Daigaku, JAPAN

## Abstract

We aimed to understand changes in activity of oropharyngeal muscles and respiratory airflow during chewing and swallowing in two clinically important conditions: gradual weight-gain volumetric enlargement and acute surgical reduction of the tongue base. Six same-sex sibling pairs of 8-to-9-month-old Yucatan minipigs, 3-each-sex, were studied. Of each pair one was fed with high-calorie pellets to reach obesity with a BMI > 50 (enlargement group), and the other was normal-weighted (BMI < 38) and underwent surgical volumetric reduction of the tongue base (reduction group). Electromyograms (EMG) of the tongue (genioglossus and styloglossus), palatal (tensor veli palatini and levator veli palatini), hyoid/pharyngeal (thyrohyoid and middle pharyngeal constrictor), and jaw (masseter and digastric) muscles were recorded simultaneously with respiratory airflow monitoring and x-ray videofluoroscopy during unrestrained feeding of pellets mixed with barium-sulfate. Videofluoroscopic images and activity-bursts of the digastric and thyrohyoid muscles were references to detect chewing cycles and swallowing episodes. Recording sessions for both groups occurred at baseline (before surgery in reduction group), and weeks 1, 3, and 5 (after surgery in reduction group). EMG activity amplitudes, burst-durations, onsets (timings of muscle activity), and respiratory airflow were evaluated. Altered chewing EMG included delayed onsets of the tensor veli palatini and styloglossus in both groups at weeks 1, 3, and 5 compared to the baseline (p < 0.05). In addition, the activity amplitudes of the styloglossus and masseter muscles in the enlargement group were higher in comparison to those of the reduction group (p < 0.05). Effects on swallowing involved delayed timings of the palatal and tongue muscle activities in the reduction group with potential impaired swallowing after surgery. The enlargement group was susceptible to breathing alterations evidenced by decreased airflow velocity, airflow pressure and tidal volumes in comparison to those of the reduction group (p < 0.05). These findings reveal adaptations to ensure synchronized feeding and respiration under volumetric alterations of the tongue base, with risks of impaired swallowing and respiratory patency.

## Introduction

The tongue base is a critical structure in mastication and respiration with a high susceptibility to its volumetric changes with relevant functional adaptations.

Enlarged tongue or macroglossia is a disorder often linked to conditions such as Beckwith-Wiedemann syndrome, Down syndrome, some forms of cancer, and severe infections [1,2]. Moreover, the increased prevalence of obesity unveils enlargement of oropharyngeal structures such as the tongue base, due to extensive infiltration of adipose tissue, which may cause breathing disorders and swallowing impairment [3–6].

In contrast, reduced tongue or microglossia occurs due to malformations with impaired tongue development or procedures with partial resection of the tongue [7,8]. Surgical volumetric reduction of the tongue base (tongue base ablation) is commonly used in the treatment of trauma, cancer, obstructive sleep apnea (OSA) and other oropharyngeal disorders [8,9].

One of the surgical approaches for volumetric reduction of the tongue base is called controlled ablation or coblation, that breaks the targeted area using a bipolar low-frequency energy in a conductive fluid creating a plasma field. This is a target-oriented procedure that preserves surrounding tissues and diminishes the risk of massive bleeding during surgery [10–13].

Even though gradual weight-gain volumetric enlargement and acute surgical reduction of the tongue base are both clinically relevant pathological models, their effects on the oropharyngeal function in chewing and swallowing are poorly understood. This limits clinical evaluations on outcomes and potential complications out of these conditions.

Our previous study in normal young adult minipigs indicated a prominent activity of the tongue, palatal and pharyngeal muscles in chewing and swallowing. There was increasing airflow velocity with decreasing airflow pressure from chewing phases to swallowing [14].

Using the same breed of minipigs, the present study aimed to investigate the consequences on vital oropharyngeal functions of two distinct and opposite clinically important states of the tongue base: adipose accumulation (gradual volumetric enlargement) and ablation (acute volumetric reduction). Theoretically, using the tongue base volume as the primary comparison axis in the present study is justified by 1) the crucial role of the tongue base in swallowing; 2) the obstructive effects caused by oversized oropharyngeal structures due to gradual fat infiltration and reduced obstructive effects of acute surgical tongue reduction; and 3) the potential impaired hydrostat nature of the tongue movements occurring due to surgical injury.

In humans, data indicate that gradually increased tongue base volume due to fat infiltration leads to narrow pharyngeal airway spaces with higher apneic events during sleep [6]. In contrast, acute surgical tongue reduction can improve obstructive sleep apnea in cases where the obstruction occurs at the tongue base [15]. Thus, these two types of tongue volume change are clinically important in the management of obstructive sleep apnea. Furthermore, some cases of gradual tongue enlargement or acute surgical tongue reduction can alter swallowing and lead to dysphagia [16–19]. However, further effects on airflow dynamics and muscle activity are not reported. Thus, in the present study, it was hypothesized that during feeding, a surgically volume-reduced tongue base may induce acute swallowing disorders whereas a gradual weight-gain volume-enlarged tongue base may induce alterations in the respiratory dynamics. The outcomes from these two clinically relevant pathological models will provide a deeper understanding of the mechanisms involved in breathing and swallowing disorders that progressively develop with functional impairments.

## Materials and methods

### Animals

Six 8–9-month-old same sex sibling pairs (3 in each sex, total of 12, Premier BioSource, CA, USA) of Yucatan minipigs were studied.

Upon the preorder, the Vendor was requested to separate a sibling pair for the normal (tongue base reduction) and obese (tongue base enlargement) groups 10–12 weeks before the delivery.

**Fig 1:**
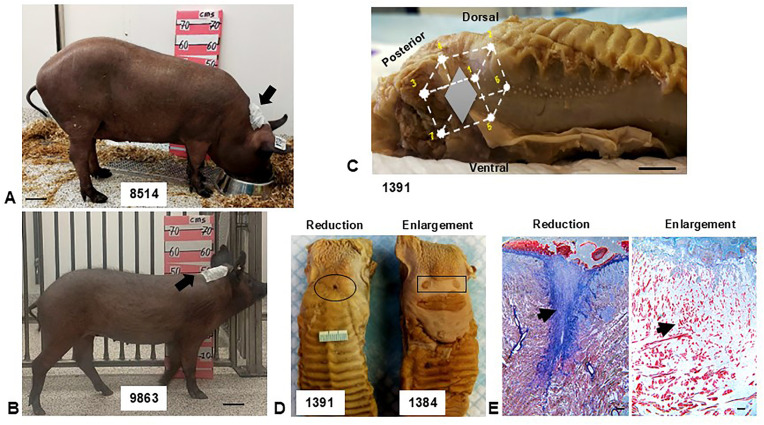
A and B: Minipigs after implantation of ultrasound crystals and skin button (arrows pointing at the dressing). **A.** Obese with volume-enlarged tongue base (8514). **B.** Normal weighted with volume-reduced tongue base (9863). Scale: 10 cm. **C.** Posterior half of the dissected tongue illustrating 8 ultrasound crystal implantation array in the tongue base (numbered circles). **D.** Illustration of surgical volumetric reduction in normal weighted minipigs. A coblation wand plugged into a radiofrequency device (Coblator) was used and the volumetric reduction was within the circumscribed region by the 8 implanted ultrasound crystals (numbered empty circles). CP: Circumvallate papilla. **E.** Harvested tongues after fixation by perfusion in the terminal day. Scar tissue (circle, 1391) is observed in the tongue base of the volume-reduced tongue base. Circumvallate papilla (box, 1384) anatomically maintained is observed in the volume-enlarged group. Scale: 1.5 cm. **F.** Representative images at week 5. Trichrome staining exhibits scar tissue formation (arrow, blue area) in the volume-reduced tongue base and increased adipose tissue accumulation (arrow, empty areas) in the volume-enlarged group. Scale: 200µm.

The normal one was fed as routine with the required body mass index (BMI) ≤ 35 Kg/m^2^, and the obese one was fed high-calorie chow pellet *ad libitum* to reach the required BMI ≥ 50 Kg/m^2^ (**Fig 1A** and **1B**). After delivery, each minipig was housed one per pen and acclimated for 5–7days receiving water *ad libitum*.

During housing, the normal-weighted minipig was fed with regular pig chow pellet twice a day, and the obese one was fed *ad libitum* high-calorie chow pellet provided by the vendor. All pens were equipped with environmental enrichment, 12-hours light/dark cycles, and room temperature at 22-24^o^C provided by the Animal Research and Care Facility.

Daily training was performed during light cycles three days after arrival until the last timepoint. During this training all minipigs were unrestrainedly fed on a customized table to get them used to the personnel and the procedures involved.

Food was deprived 12-16h before anesthesia and recording sessions. All procedures were adhered to the ARRIVE (Animal Research Reporting of In Vivo Experiments) guidelines and approved by the Institutional Animal Care and Use Committee, University of Washington (protocol# 3393–05).

### Ultrasound crystal implantation

After acclimation eight barbed ultrasound crystals with a skin button set (Sonometrics Co., London, ON, Canada) were surgically implanted in the tongue base forming a cubic-shaped area as initially done in the normal weighted minipigs [20]. In brief, a small incision was made 10 cm posterior to the occipital protuberance to insert a sterilized 1.0 cm open trocar for subcutaneous tunneling to the posterior submandibular area. All wires connected to the crystals were led through this tunnel and the crystals were implanted using the hyoid bone as reference of the tongue base location. Four crystals (#1–4, **Fig 1C**) were implanted in the dorsal region followed by four additional crystals (#5–8, **Fig 1C**) implanted in the ventral region about 20 mm vertical to the dorsal ones. The leading wires of each crystal were sutured to the nearby tissue, and the incisions were closed in layers.

Lastly, the female interface of the skin button set with a protected cap was fixed to the nearby skin using 0−0 non-absorbable polypropylene monofilament suture. Analgesia and post-operative follow up were provided as prescribed.

This procedure was conducted for longitudinal recordings of 3D shape changes of the tongue base and the results will be reported elsewhere.

### Baseline recordings

Under anesthesia, eight pairs of 0.1 mm fine wire electromyographic (EMG) electrodes were inserted in the left tongue (genioglossus and styloglossus), palatal (tensor veli palatini and levator veli palatini), hyoid/pharyngeal (thyrohyoid and middle pharyngeal constrictor), and jaw (masseter and digastric) muscles via 25G or spinal needles. The reference electrode was placed in the forehead. All electrodes were taped to the nearby skin area and attached to EMG leads then connected to the MP160 data acquisition system (Biopac Inc, CA).

A nasal catheter was inserted close to the nasopharyngeal region (50 mm deep in the left nostril) and sutured to the snout skin. The external end of the catheter was connected to the pneumotach sensors to detect airflow velocity and airflow pressure (TSD160A-TSD237F via MP160, Biopac, Inc, CA).

After anesthesia withdrawal, the minipig unrestrainedly ate chow pellet with or without barium sulfate suspension (Vet-Paque, Jorgensen Laboratories Inc. USA) by standing on the custom-made feeding table. The EMG and respiratory airflow signals were recorded simultaneously before and during spontaneous feeding for 8–10 min. The EMG sampling rate was 1000 Hz using AcqKnowledge software (Ver. 5.0, Biopac Inc, CA). Synchronized with EMG, airflow, and ultrasound crystal recordings, the x-ray videofluoroscopy imaging (GE Healthcare, OEC 9900 Elite, USA) of the oropharyngeal region was recorded from the lateral view at 30 frames/second to verify swallowing episodes during unrestrained feeding.

### Surgical tongue base volume reduction

Three days after baseline recording, the surgical volumetric reduction was performed on both tongue base and left masseter muscle in the normal weighted animals. The purpose of masseter muscle included was to create comparable counterpart of myogenic healing and regeneration sites with the tongue muscles, as these two muscle tissues have different embryological origins: the tongue muscles derive from occipital somites, but masseter from the cranial paraxial mesoderm (pharyngeal arch) [21]. The results of these comparisons will be reported elsewhere.

A coblator console (ArthroCare ENT Coblator II, CA, USA) and a plugged wand (ArthroCare ENT Procise EZ View, CA, USA) were connected to a fluid tubing drip (10 drops per minute) and a bag of 500 ml 0.9% saline solution (Baxter, USA). The machine settings were at 7–9 in the coblation mode and at 5–6 in the coagulation mode for controlled tongue base ablation and hemostasis. The saline solution drip was adjusted at a continuous rate throughout surgery. This solution ensures electro-conduction generating small amounts of heat to break the tissue with low risk of massive bleeding.

Under anesthesia with xylazine/midazolam and endotracheal intubation with 2–3% isoflurane in oxygen, the minipig was initially positioned on its lateral right side. Then, the posterior region of the left masseter closer to the mandibular angle was exposed to ablate 2–4% of muscle mass followed by surgical wound closure [22]. The minipig was repositioned on its chest for tongue base volume reduction. After widely opened, the mouth was stabilized by the two mouth openers at each side. The coblation wand reached the area of the implanted ultrasound crystals to ablate the tissue within the space of the cubic-shaped area circumscribed by the eight implanted crystals (**Fig 1D**). The ablated site was irrigated to remove debris, and the wound remained open without suturing. The minipig was fed with slurry chow pellets in water for the next three days after surgery and then with regular chow pellets. Prophylactic antibiotics were administered as prescribed.

### Longitudinal recordings

The longitudinal recordings of ultrasound crystals, EMG, and respiratory airflow were performed in weeks 1, 3, and 5 after the surgical volumetric reduction in normal weighted minipigs. The procedures were the same as for the baseline recording but x-ray video fluoroscopy was only repeated in week 5. The minipigs were euthanized after terminal recordings at week 5 using pentobarbital sodium 15 ml IV (Euthasol, Virbac, USA). The masseter muscles and tongue were harvested (**Fig 1E**) after cardiac perfusion and fixation**.**

The choice of this 5-week experimental period was based upon our previously study on the glossectomy of the tongue body in the same minipig model, in which the complete healing was seen in week 4 [23]. Although a longer experimental period would provide more insight into fibrosis, muscle regeneration and remodeling, as well functional recovery, the limited life span of the implanted SONO system and potential risk of continuous obesity on animal welfare prevented a longer experimental period.

### Data processing and analysis

**Fig 2:**
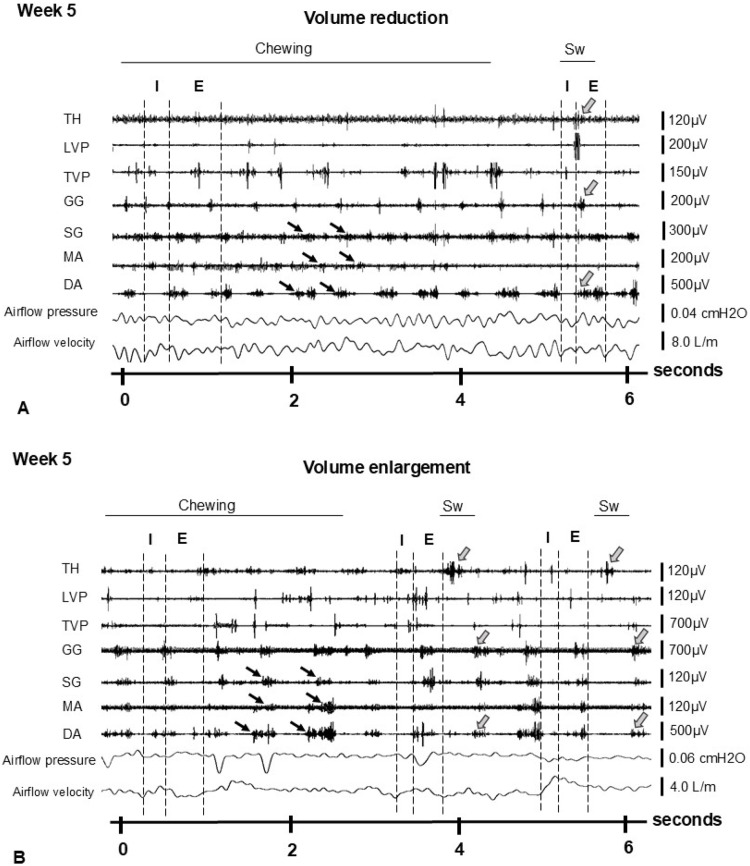
Electromyographic recordings during unrestrained feeding in week 5. **A.** Volume-reduced minipig (1391). **B.** Volume-enlarged minipig (8514). The dotted lines indicate respiratory phases based on the airflow velocity dynamics. I: Inspiration. E: Expiration. The chewing cycle length was defined between the two onsets of DA activity including jaw opening (black arrows in DA) and closing/power stroke (black arrows in SG and MA). The swallowing episodes (gray arrows in TH) appeared either at the post-inspiration (A) or post-expiration (B) followed by jaw opening (arrows in GG and DA). TH: Thyrohyoid. LVP: Levator veli palatini. TVP: Tensor veli palatini. GG: Genioglossus. SG: Styloglossus. MA: Masseter. DA: Digastric.

All EMG and airflow signals were processed and analyzed as previously described [14] and compared between the two groups at each timepoint (baseline, week 1, 3, and 5, **Fig 2A and 2B**), and across the four timepoints in each group. The detailed sample sizes of chewing cycles, swallowing episodes, and respiration cycles for each timepoint and animal are summarized in Tab 1.

SPSS (Ver. 19, IBM) was used for statistical analysis. All data from both groups showed p  <  0.05 on the tests for normality and homogeneity of variances. Therefore, non-parametric Kruskal-Wallis’ test followed by the pair-wise multiplemultiple comparisons (U-tests) were applied. Significance level was set as p  <  0.05.

## Results

### General findings

All minipigs survived the surgeries for ultrasound crystal implantation, and all normal weighted minipigs also survived the surgical volumetric reduction. The BMI at the last timepoint was 37.83 ± 3.54 in the volume-reduced (normal weighted) group, and 60.33 ± 3.93 in the volume-enlarged (obese) minipigs. One of the volume-enlarged minipigs (9822, Table 1) had a weight loss throughout experiments but remained above the threshold (BMI  >  50) in week 5. Table 1 summarizes the BMI for each minipig upon arrival and the terminal at week 5.

**Table 1:** Summaries of the sample sizes and time lengths for each experimental group.

Pig number	Group	Body Mass Index (Kg/m²)			Chewing cycle length (seconds)				Swallowing cycle length (seconds)					Respiratory cycle length chewing (seconds)					Respiratory cycle length swallowing (seconds)			
		Baseline	Week 5	Total	Baseline	Week 1	Week 3	Week 5	Total	Baseline	Week 1	Week 3	Week 5	Total	Baseline	Week 1	Week 3	Week 5	Baseline	Week 1	Week 3	Week 5
7645	Reduction	35	43	11	.	.	0.55	0.49	3	.	.	0.29	0.15	4	.	.	2.13	2.24	.	.	1.32	2.24
8501	Reduction	27	33	19	.	0.67	0.69	.	6	.	0.28	0.27	.	5	.	1.87	1.63	.	.	1.71	1.09	.
9863	Reduction	36	40	34	0.62	0.61	0.61	0.69	11	0.24	0.27	0.26	0.23	12								
1391	Reduction	35	39	20	0.73	0.59	0.55	0.68	6	0.22	0.23	0.21	0.36	7	1.76	1.61	1.51	0.17	1.76	1.66	1.61	1.57
3370	Reduction	33	36	27	0.55	0.53	0.52	0.53	8	0.19	0.21	0.26	.	9	1.29	1.73	1.83	1.60	0.95	1.58	1.27	1.24
4032	Reduction	31	36	45	0.50	0.56	0.51	0.57	12	0.24	0.22	0.19	0.23	15	1.56	1.72	1.34	1.48	1.65	1.11	1.01	1.49
7658	Enlargement	51	58	10	0.68	.	.	.	3	0.35	.	.	.	3	2.26	.	.	.	2.07	.	.	.
8514	Enlargement	53	63	21	.	0.62	0.64	0.61	7	.	0.21	0.22	0.23	7	.	1.77	1.68	1.61	.	1.33	1.17	0.86
9822	Enlargement	69	62	31	0.63	0.63	0.67	0.63	9	0.21	0.24	0.25	0.25	10	1.97	1.76	1.48	1.64	1.43	1.45	1.21	1.51
1384	Enlargement	56	60	19	0.64	0.67	0.63	0.54	7	0.20	0.15	0.14	0.13	6	1.77	1.67	1.42	1.83	1.64	1.38	1.17	1.65
3362	Enlargement	56	65	46	0.48	0.50	0.51	0.48	14	0.26	0.26	0.25	0.23	15	1.59	1.63	1.84	1.78	1.57	1.44	1.51	1.62
4036	Enlargement	50	54	32	0.55	0.49	0.67	0.66	8	0.28	0.23	0.26	0.20	11	1.69	1.50	1.31	1.65	1.65	1.45	1.20	1.34
**Reduction**	**Average**	**32.83**	**37.83**		**0.60**	**0.59**	**0.57**	**0.59**		**0.22**	**0.24**	**0.25**	**0.24**		**1.54**	**1.73**	**1.69**	**1.37**	**1.45**	**1.52**	**1.26**	**1.64**
	**SD**	**3.37**	**3.54**		**0.10**	**0.05**	**0.07**	**0.09**		**0.02**	**0.03**	**0.04**	**0.09**		**0.24**	**0.11**	**0.30**	**0.87**	**0.44**	**0.28**	**0.23**	**0.43**
**Enlargement**	**Average**	**55.83**	**60.33**		**0.60**	**0.58**	**0.62**	**0.58**		**0.26**	**0.22**	**0.22**	**0.21**		**1.86**	**1.67**	**1.55**	**1.70**	**1.67**	**1.41**	**1.25**	**1.40**
	**SD**	**6.91**	**3.93**		**0.08**	**0.08**	**0.07**	**0.07**		**0.06**	**0.04**	**0.05**	**0.05**		**0.27**	**0.11**	**0.21**	**0.10**	**0.24**	**0.05**	**0.15**	**0.32**

The average tongue weights were 107.72 ± 10.40g in the volume-reduced and 125.47 ± 17.21g in the volume-enlarged group. The average volumes of the tongue base were 33.2 ± 4.0 ml and 44.0 ± 4.0 ml in the volume-reduced and enlarged groups respectively. Our previous study of control minipigs with intact tongue base showed a tongue base volume of 33.9 ± 10.7 ml but these controls were 5 weeks younger than the present ones [14], Scar tissue formation and wound contraction around the circumvallate papilla were observed in all tongues related to the volume reduction surgery (**Fig 1E**).

Chewing and swallowing data along with respiration for all time points were retrieved and analyzed. Recordings with unclear activity and noisy signals were excluded. The analyzed chewing cycles, swallowing episodes, and respiratory cycles are summarized in Table 1. Overall, swallowing episodes lasted 0.93 ± 0.54 seconds after the last chewing cycle but not all chewing cycles ended up with swallowing. No significant differences were detected between male and female minipigs in all measured variables.

### Timing of muscle activity

#### Chewing.

As shown in **Fig 2**, the activity bursts of the ipsilateral digastric occurred in jaw opening whereas the activity of the masseter was seen in jaw closing/power stroke.

**Fig 3:**
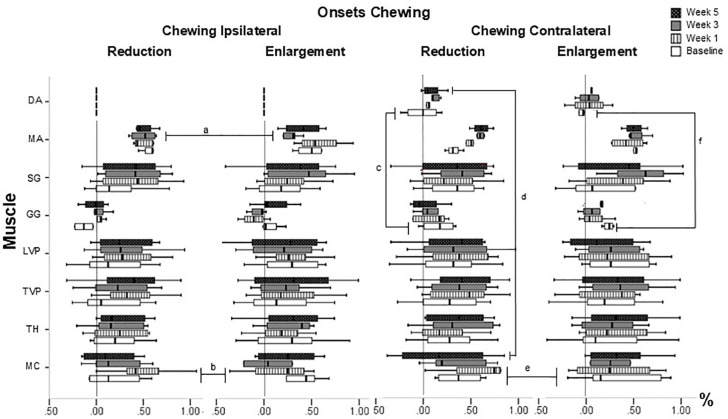
Box-and-whisker plots show the comparisons of burst onsets in chewing at the four timepoints and between the two groups. The vertical lines on each bar, and error sticks indicate median and range. Lines at zero indicate the beginning of the jaw opening phase based on the ipsilateral DA burst onset (time zero). All onsets were converted to the percentage of the total chewing cycle length for standardization. MC: middle pharyngeal constrictor. TH: Thyrohyoid. TVP: Tensor veli palatini. LVP: Levator veli palatini. GG: Genioglossus. SG: Styloglossus. MA: Masseter. DA: Digastric. Superscripts indicate significant differences at week 3 in the ipsilateral MA between volume-reduced and volume-enlarged groups (a); at baseline and week 1 in the ipsilateral MC between groups (b); at baseline between contralateral DA and GG in the volume-reduced group (c); at week 5 between contralateral MC and DA in the volume-reduced group (d); at baseline and week 1 in the contralateral MC between groups (e); and, at baseline between contralateral DA and GG in the volume-enlarged group (f). P < 0.05 by Kruskal-Wallis and U-tests.

To standardize all burst onsets among different chewing cycle lengths, the onset of ipsilateral digastric was taken as a reference (time zero). Then, all other onsets of different muscles and contralateral digastric were converted to the percentage of the corresponding chewing cycle lengths as summarized in **Fig 3**.

At baseline in the volume-reduced group, earlier burst activities predominated in the ipsilateral tongue and palatal muscles (5–13%). In the volume-enlarged group, earlier activities occurred in the ipsilateral genioglossus and tensor veli palatini (4–26%), and the contralateral digastric (1–6%). The onset of the ipsilateral middle pharyngeal constrictor occurred significantly earlier in the volume-reduced compared to the volume-enlarged (p < 0.05). In both groups, the onsets of the contralateral digastric occurred significantly earlier than those of the genioglossus (p < 0.05, **Fig 3**).

At week 1, substantial shifts in timing were observed in the volume-reduced group. The timings of the middle pharyngeal constrictor, tensor veli palatini, and levator veli palatini occurred later in comparison to those at baseline. Significant differences were found in the middle pharyngeal constrictor between the volume-reduced (2.5–41.5% ipsilateral, and 30.7–40.1% contralateral, both later than baseline) and volume-enlarged group (30.3–53.9% ipsilateral – earlier than baseline, and −12.2–30.4% contralateral, p < 0.05).

At week 3, similar EMG activities persisted in the volume-reduced group. The onsets of the masseter occurred significantly later in the volume-reduced group (37.9–62.9% ipsilateral, and 56.6–63.4% contralateral) compared to those of the volume-enlarged group (20.3–38.1% ipsilateral, and 42.6–64.2% contralateral, p < 0.05). In the volume-enlarged group the activities of the contralateral digastric (−8.9–13.5%) and styloglossus (20.6–67.4%) occurred later than their baselines (p < 0.05).

At week 5, in the volume-reduced group the activity of the contralateral middle pharyngeal constrictor (18.5–47.9%) was significantly different than that of the contralateral digastric (−8.5–11.7%, p < 0.05). In the volume-enlarged group the ipsilateral activities of the genioglossus (jaw opening) and the masseter (jaw closing/power stroke) overlapped, and this may be related to the functional adaptations of the enlarged tongue base in this minipig model (**Fig 3**).

The burst durations lasted 15-40% of the chewing cycle length with no significant differences between the two experimental groups across the four timepoints. The ipsilateral activity durations over time for each group are shown on Table 2.

**Table 2:** Percentage of ipsilateral activity durations in chewing and durations in swallowing (Mean ± SD).

Group - Function	Timepoint	Muscle							
		MC	TH	TVP	LVP	GG	SG	MA	DA
Reduction - Chewing	Baseline	0.24 ± 0.05	0.22 ± 0.04	0.21 ± 0.04	0.21 ± 0.03	0.26 ± 0.03	0.21 ± 0.03	0.24 ± 0.04	0.22 ± 0.02
	Week-1	0.25 ± 0.04	0.23 ± 0.02	0.23 ± 0.02	0.24 ± 0.02	0.32 ± 0.14	0.24 ± 0.03	0.25 ± 0.01	0.24 ± 0.02
	Week-3	0.24 ± 0.03	0.22 ± 0.01	0.24 ± 0.03	0.23 ± 0.02	0.24 ± 0.02	0.22 ± 0.02	0.25 ± 0.03	0.23 ± 0.01
	Week-5	0.30 ± 0.05	0.25 ± 0.09	0.23 ± 0.06	0.28 ± 0.07	0.27 ± 0.04	0.25 ± 0.06	0.28 ± 0.04	0.26 ± 0.09
	Baseline	0.23 ± 0.07	0.22 ± 0.04	0.23 ± 0.04	0.23 ± 0.034	0.27 ± 0.02	0.22 ± 0.04	0.26 ± 0.06	0.25 ± 0.03
Enlargement - Chewing	Week-1	0.21 ± 0.04	0.23 ± 0.04	0.25 ± 0.06	0.24 ± 0.06	0.30 ± 0.03	0.23 ± 0.05	0.25 ± 0.05	0.23 ± 0.02
	Week-3	0.21 ± 0.01	0.24 ± 0.02	0.24 ± 0.02	0.23 ± 0.02	0.25 ± 0.04	0.21 ± 0.03	0.26 ± 0.03	0.23 ± 0.01
	Week-5	0.21 ± 0.03	0.24 ± 0.03	0.25 ± 0.04	0.25 ± 0.05	0.26 ± 0.03	0.24 ± 0.03	0.28 ± 0.03	0.22 ± 0.01
Reduction - Swallowing	Baseline	0.78 ± 0.21	0.89 ± 0.08	0.68 ± 0.10	0.73 ± 0.07	.	0.71 ± 0.08	.	0.75 ± 0.15
	Week-1	0.87 ± 0.14	0.82 ± 0.06	0.71 ± 0.09	0.76 ± 0.08	.	0.75 ± 0.13	.	0.75 ± 0.13
	Week-3	0.93 ± 0.06	0.86 ± 0.15	0.71 ± 0.12	0.79 ± 0.14	.	0.79 ± 0.14	.	0.73 ± 0.08
	Week-5	0.93 ± 0.03	0.89 ± 0.05	0.83 ± 0.21	0.77 ± 0.16	.	0.70 ± 0.15	.	0.81 ± 0.21
Enlargement - Swallowing	Baseline	0.83 ± 0.19	0.87 ± 0.15	0.67 ± 0.19	0.61 ± 0.06	.	0.70 ± 0.17	.	0.76 ± 0.24
	Week-1	0.90 ± 0.08	0.91 ± 0.09	0.84 ± 0.15	0.78 ± 0.11	.	0.73 ± 0.09	.	0.84 ± 0.17
	Week-3	0.76 ± 0.28	0.91 ± 0.10	0.77 ± 0.05	0.73 ± 0.09	.	0.77 ± 0.06	.	0.76 ± 0.14
	Week-5	0.94 ± 0.02	0.99 ± 0.06	0.85 ± 0.09	0.87 ± 0.06	.	0.84 ± 0.11	.	0.84 ± 0.07

MC: middle pharyngeal constrictor. TH: Thyrohyoid. TVP: Tensor veli palatini. LVP: Levator veli palatini. GG: Genioglossus. SG: Styloglossus. MA: Masseter. DA: Digastric.

The duration of the chewing phases (jaw opening and jaw closing/power stroke) in relation to the total chewing cycle length massively changed over time. In the volume-reduced group the jaw opening phase was shorter than jaw closing phase at baseline and week 5 whereas in the volume-enlarged group the jaw opening showed increasing durations. These data are summarized in Table 3.

**Table 3:** Percentage of chewing cycle phases for each group and time point.

		Tongue base Reduction	Tongue base Enlargement	Normal tongue base*
**Jaw Opening (%)**	**Baseline**	47.98 ± 16.41%	50.20 ± 19.24%	42.20 ± 19.2%
	**Week-1**	59.69 ± 16.16%	53.47 ± 24.52%	
	**Week-3**	58.50 ± 12.82%	53.28 ± 20.64%	
	**Week-5**	36.36 ± 18.23%	53.24 ± 23.45%	
**Jaw closing/** **power stroke (%)**	**Baseline**	52.02 ± 18.01%	49.80 ± 20.29%	57.80 ± 19.7%
	**Week-1**	40.31 ± 17.16%	46.53 ± 20.45%	
	**Week-3**	41.50 ± 15.62%	46.72 ± 17.31%	
	**Week-5**	63.64 ± 40.23%	46.76 ± 23.01%	

*Rosero Salazar, Honnlee, & Liu, 2024.

#### Swallowing.

Both groups showed major activities of the thyrohyoid muscle during swallowing verified by the simultaneous records of x-ray video fluoroscopy. Thus, this muscle was used as reference for EMG timing analysis of swallowing episodes. Due to swallowing is a propulsive movement, no side was considered for the analysis of these episodes.

**Fig 4:**
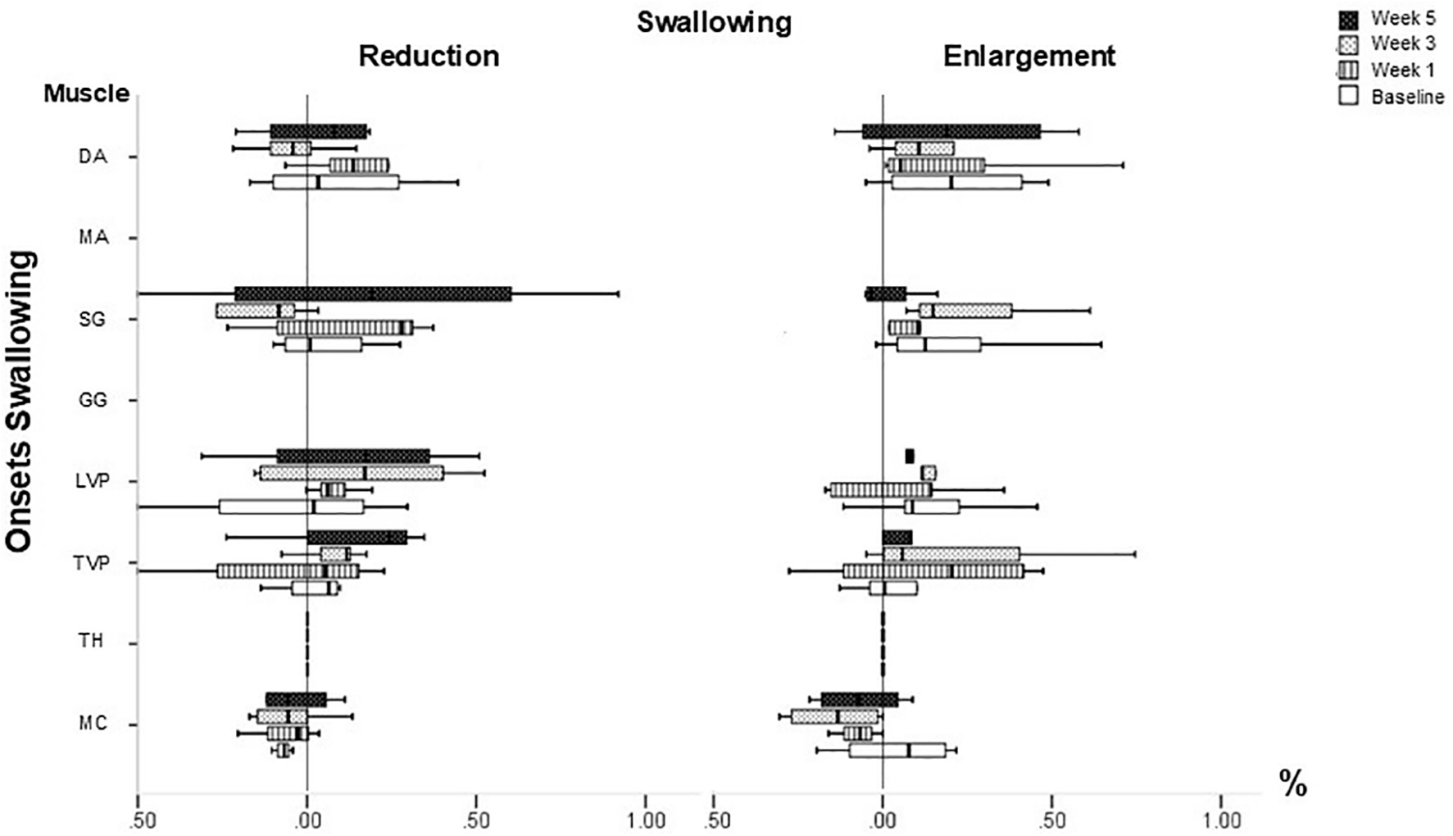
Box-and-whisker plots show the electromyographic activity onsets of swallowing for experimental groups and timepoints. All onsets from each muscle were converted to the percentage of the total swallowing duration for standardization. Lines indicate the zero-point by TH onsets. Bar positions indicate percentages of EMG activity onsets in relation to the total swallowing durations. Refer to **Fig 3** for plot’s muscle’s definitions.

At baseline, the middle pharyngeal constrictor showed earlier onsets in the volume-reduced group 1–15% than the reference. In the volume-enlarged group all activities appeared 5–20% later than those of the thyrohyoid (**Fig 4**).

At week 1, the main shifts were detected in the volume-reduced group on the later activities of the digastric, styloglossus, and levator veli palatini compared to their baselines. In the volume-enlarged group, the onsets of the digastric and middle pharyngeal constrictor occurred 15–20% earlier compared to their baselines.

At week 3, the volume-reduced group showed 5–15% earlier activities of the digastric and styloglossus, and later activities of the palatal muscles compared to their baselines. In the volume-enlarged group the digastric and middle pharyngeal constrictor showed earlier activities than previous timepoints, whereas in the palatal and tongue muscles onsets were delayed but close to the baseline.

At week 5, the main changes in the volume-reduced group were later onsets mainly in the palatal and tongue muscles in comparison to the reference and their baselines. In the volume-enlarged group, the activities of the tongue and pharyngeal muscles occurred 5–10% earlier than the reference and their baselines (**Fig 4**).

No significant differences in activity timings and durations were detected over the four timepoints and between the two experimental groups. Durations over the four timepoints for each group during swallowing are shown on Table 2.

### Amplitudes of muscle activity

**Fig 5:**
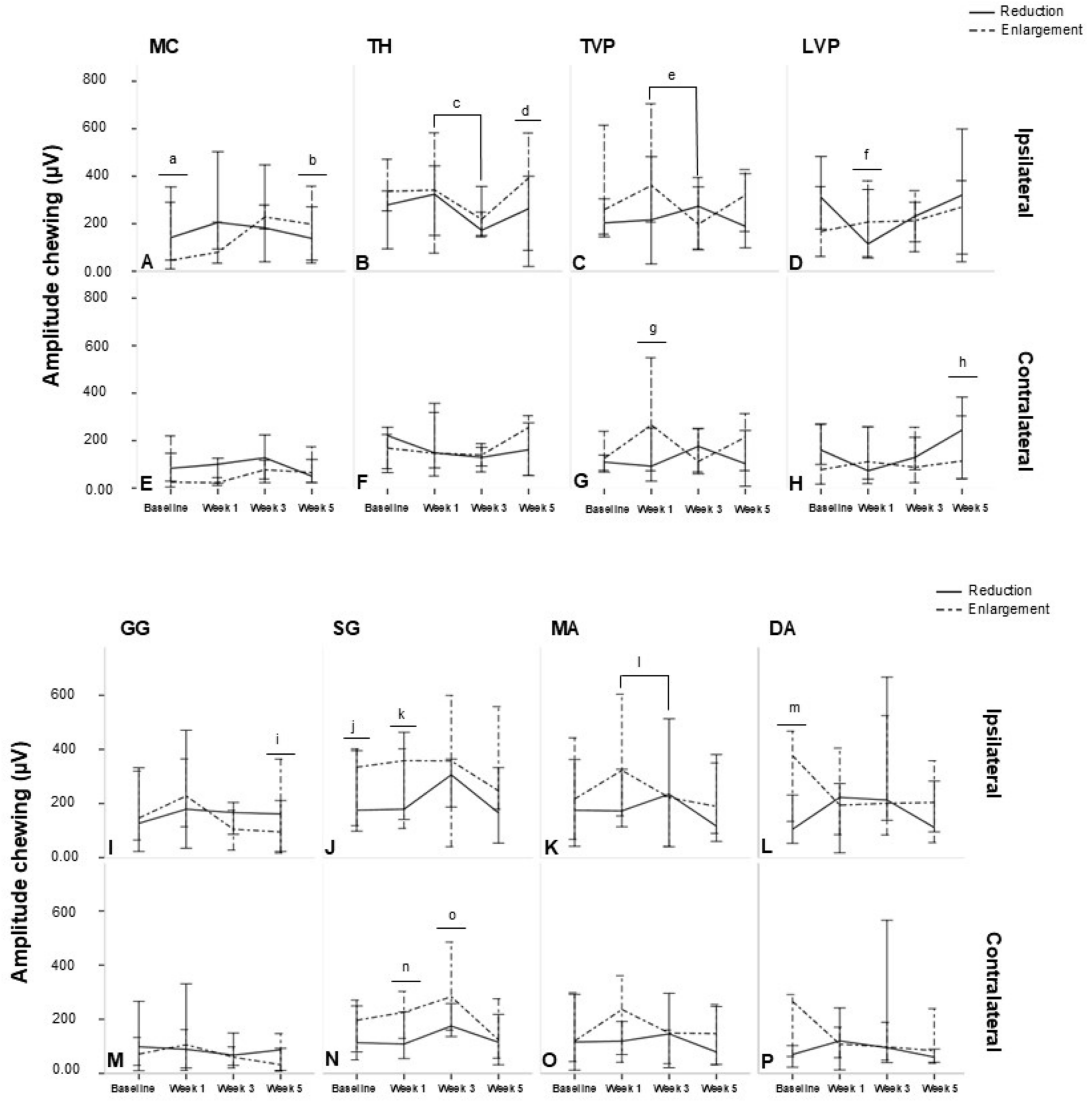
Median amplitudes in microvolts (µV) during chewing. Range of activity for each muscle (**A-L**/ipsilateral and **E-P**/contralateral), timepoints, chewing side, and experimental groups. MC: middle pharyngeal constrictor. TH: Thyrohyoid. TVP: Tensor veli palatini. LVP: Levator veli palatini. GG: Genioglossus. SG: Styloglossus. MA: Masseter. DA: Digastric. Superscript letters indicate significant differences in MC (**A**) between groups at baseline (a) and at week 5 (b); in TH (**B**) between week 1 and week 3 (c) and between groups at week 5 (d); in TVP (**C**) between week 1 and week 3 (e); in LVP (**D**) between experimental groups at week 1 (f); in contralateral TVP (**G**) between groups at week 1 (g); in contralateral LVP (**H**) between groups at week 5 (h);. Superscript letters indicate significant differences in GG (**I**) between groups at week 5 (i); in SG (**J**) between groups at baseline (j) and at week 1 (k); in MA (**K**) between week 1 and week 3 (l); in DA (**L**) between groups at baseline (m); and, in the contralateral SG (**N**) between groups at week 1 (n) and at week 3 (o). p < 0.05 by the non-parametric Kruskal-Wallis and U-tests.

**Fig 6:**
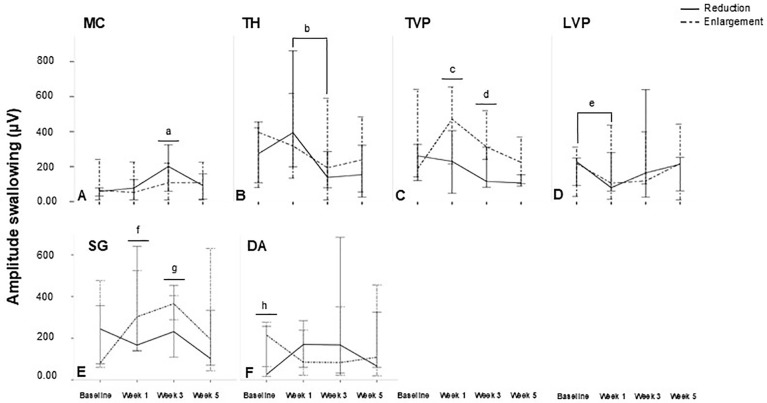
Median amplitudes in microvolts (µV) during swallowing. Range of activity for each muscle (**A-F**), timepoints, chewing side, and experimental groups. MC: middle pharyngeal constrictor. TH: Thyrohyoid. TVP: Tensor veli palatini. LVP: Levator veli palatini. SG: Styloglossus. DA: Digastric. Superscript letters indicate significant differences in MC (**A**) between groups at week 3 (a); in TH (**B**) between week 1 and week 3 (b); in TVP (**C**) between groups at week 1 (c) and week 3 (d); in LVP (**D**) between baseline and week 1 (e); in SG (**E**) between groups at week 1 (f) and week 3 (g); and, in DA (**F**) between groups at baseline (h). p < 0.05 by the non-parametric Kruskal-Wallis and U-tests.

All animals and muscles showed larger ipsilateral amplitudes than their contralateral ones. Significant differences in chewing and swallowing were detected between the two groups and over the four timepoints (**Figs 5** and **6**)**.**

#### Chewing.

The ipsilateral middle pharyngeal constrictor at baseline showed significantly larger amplitudes in the volume-reduced than those of the volume-enlarged group (p < 0.05). Ipsilateral amplitudes in the volume-enlarged group increased overtime being larger at week 5 than those of the volume-reduced group (p < 0.05, **Fig 5A** and **5E**).

The amplitudes in the ipsilateral thyrohyoid muscle were significantly larger in both groups at week 1 compared to those at week 3 (p < 0.05). At week 5, the ipsilateral amplitude of the volume-enlarged was significantly larger than that of the volume-reduced group (p < 0.05, **Fig 5B** and **5F**).

The ipsilateral tensor veli palatini also showed significant differences in both groups between weeks 1 and 3, decreasing in the volume-enlarged and increasing in the volume-reduced group (p < 0.05, **Fig 5C** and **5G**). The contralateral side showed a higher amplitude in the volume-enlarged compared to the volume-reduced groups at week 1 (p < 0.05). The amplitude of the ipsilateral levator veli palatini in week 1 was lower in the volume-reduced compared to the volume-enlarged group (p < 0.05). However, the opposite was observed on the contralateral side at week 5 (p < 0.05, **Fig 5D** and **5H**).

The amplitude of the ipsilateral genioglossus in the volume-reduced was lower at baseline than that of the volume-enlarged groups but gradually increased over time. At week 5 the amplitude of the volume-reduced was larger than that of the volume-enlarged group (p < 0.05, **Fig 5I**).

The styloglossus showed larger amplitudes in the volume-enlarged compared to the volume-reduced groups at baseline and week 1 ipsilaterally (p < 0.05), and at weeks 1 and 3 contralaterally (p < 0.05). These amplitudes in the volume-reduced group remained lower over time on both chewing sides (**Fig 5J**).

The amplitudes of the ipsilateral masseter in the volume-enlarged group were larger in week 1 than those observed at week 3, and the opposite in the volume-reduced group (p < 0.05, **Fig 5K**). Similarly, the amplitudes of the ipsilateral digastric were larger in the volume-enlarged than those in the volume-reduced group at baseline (p < 0.05). These amplitudes were lower in week 5 compared to those at baseline (**Fig 5L**).

Overall, the muscles with the largest amplitudes during chewing were the thyrohyoid, both palatal muscles, and the digastric in both groups.

#### Swallowing.

The amplitudes of the volume-enlarged group were larger than those of the volume-reduced at weeks 1 and 3 in the tensor veli palatini and styloglossus (p < 0.05). The opposite was detected in the middle pharyngeal constrictor at week 3 (p < 0.05). The thyrohyoid amplitudes were larger in week 1 compared to those at week 3 (p < 0.05). Similarly, the levator veli palatini showed larger amplitudes during swallowing at baseline compared to those at week 1 in both groups (p < 0.05) (**Fig 6**).

Overall, the muscles showing the largest amplitudes during swallowing in the volume-enlarged group were the thyrohyoid and tensor veli palatini. In the volume-reduced group these muscles were the thyrohyoid and the levator veli palatini.

It needs to be noted that due to surgical injury by coblation, EMG changes in week 1 might be confounded by the wound healing process.

### Respiration during chewing and swallowing

Respiratory rates at pre-feeding when the minipig was awake, quiet, and not eating were 24–30/minute in the volume-reduced group, and 22–27/minute in the volume-enlarged. During chewing and swallowing the respiratory cycles were mostly shorter with the increased rates of 34–41/minute in the volume-reduced and 32–38/minute in the volume-enlarged group. Swallowing apneas occurred in both groups in about 38.3% of the total number of swallowing episodes. These apneic periods lasted between 0.14–1.40 seconds and mostly occurred during expiratory phases.

Overall, there were 1–2 chewing cycles in each respiratory cycle with 64% and 83% of the chewing cycles that occurred during expiratory phases, and 62% and 75% of swallowing episodes occurred during the expiratory phase in both groups.

#### Duration of Respiratory cycles.

**Fig 7:**
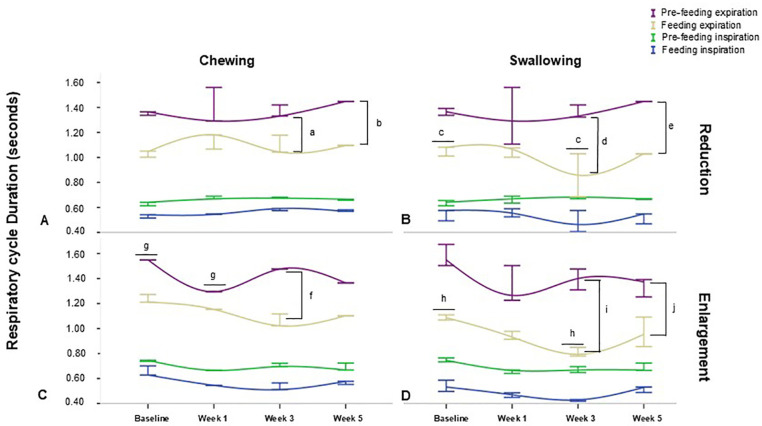
Comparisons (median and range) of respiratory cycle duration in seconds between pre-feeding and chewing (A-C), and pre-feeding and swallowing (B-D) at 4 timepoints for the two groups. Superscript letters indicate significant differences in the volume-reduced group between pre-feeding and chewing (**A**) at week 3 (a) and at week 5 (b); during swallowing (**B**) between baseline and week 3 (c); and, between pre-feeding and swallowing at week 3 (d) and week 5 (e). In the volume-enlarged group differences were detected between pre-feeding and chewing (**C**) at week 3 (f); in pre-feeding between baseline and week 1 (g); in swallowing (**D**) between baseline and week 3 (h); and, between pre-feeding and swallowing at week 3 (i) and at week 5 (j). p < 0.05 by the non-parametric Kruskal-Wallis and U-tests.

Slower respiratory rates during pre-feeding than feeding (chewing and swallowing) were observed in both groups, being significantly different in expiratory phases between different timepoints. In the volume-reduced group expiratory phases were longer in pre-feeding than those during feeding at weeks 3 and 5 (p < 0.05, **Fig 7A** and **7B**). In addition, the duration of expiratory phases during swallowing significantly decreased in week 3 compared to that of baseline (p < 0.05).

In the volume-enlarged group, expiratory phases were significantly shorter during chewing at week 3,and swallowing at weeks 3 and 5 than those of pre-feeding (p < 0.05, **Fig 7C** and **7D**). Additionally, the duration of expiratory phases in pre-feeding at baseline was longer than those at week 1 (p < 0.05), and longer expirations in swallowing were detected at baseline than those of week 3 (p < 0.05).

#### Airflow velocity, tidal volume, and pressure velocity.

**Fig 8:**
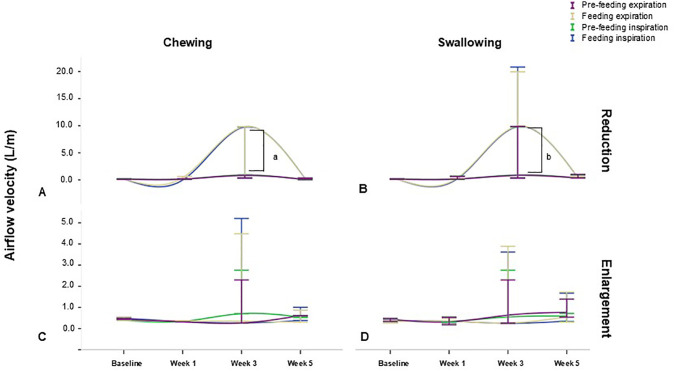
Comparisons (median and range) of airflow velocity in liters per minute (L/m) between pre-feeding and chewing (A-C), and pre-feeding and swallowing (B-D) at 4 timepoints for the two groups. Superscript letters indicate significant differences in the volume-reduced group between pre-feeding and chewing (**A**) at week 3 (a); and, during pre-feeding and swallowing (**B**) at week 3 (b). p < 0.05 by the non-parametric Kruskal-Wallis and U-tests.

The respiratory airflow velocity in the volume-reduced group increased in week 3 in both respiratory phases during feeding compared to those in pre-feeding (p < 0.05, **Fig 8A** and **8B**). In the volume-enlarged group during pre-feeding the inspiratory airflow velocity was higher than that of chewing and swallowing at week 3 without differences during expiration (**Fig 8C** and **8D**).

**Fig 9:**
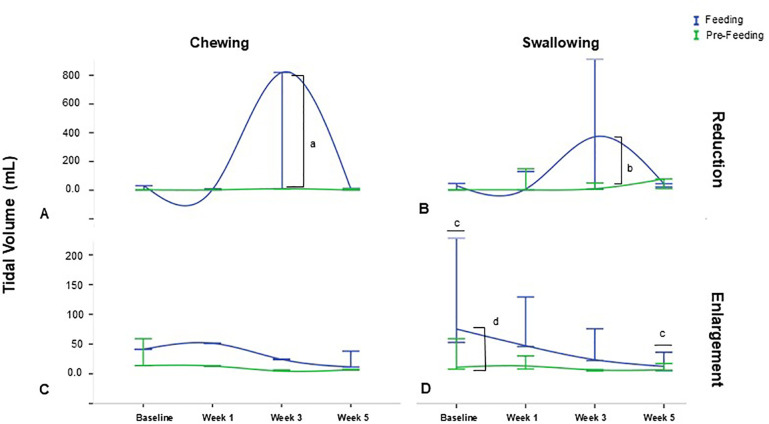
Comparisons (median and range) of tidal volume in milliliters (ml) between pre-feeding and chewing (A-C), and pre-feeding and swallowing (B-D) at 4 timepoints for the two groups. Superscript letters indicate significant differences in the volume-reduced group between pre-feeding and chewing (**A**) at week 3 (a); and, during pre-feeding and swallowing (**B**) at week 3 (b). In the volume-enlarged group differences were detected during swallowing (**D**) between baseline and week 5 (c); and between pre-feeding and swallowing at baseline (d). P < 0.05 by the non-parametric Kruskal-Wallis and U-tests.

The tidal volume, or the amount of air in and out the airway during a respiratory cycle, showed in the volume-reduced group increased volumes during chewing and swallowing at week 3 compared to those of pre-feeding (p < 0.05, Fig 9A and 9B). In the volume-enlarged group the tidal volume was significantly higher at baseline during feeding than that at week 5 (p < 0.05), and significantly higher in feeding than pre-feeding during swallowing at baseline (p < 0.05, **Fig 9C** and **9D**).

**Fig 10:**
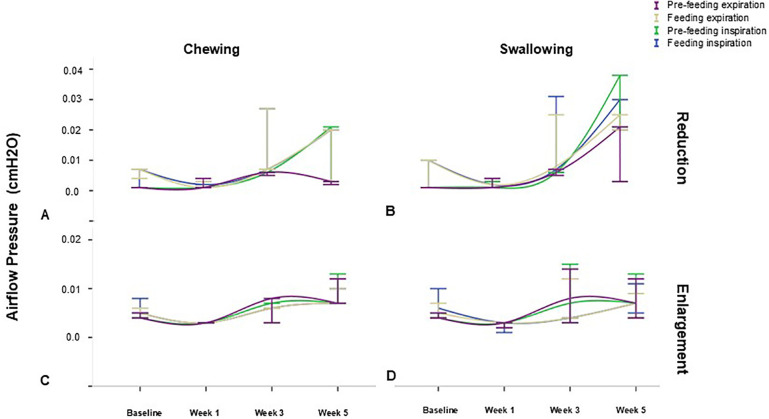
Comparisons (median and range) airflow pressure in centimeters of water (cmH_2_O) between pre-feeding and chewing (A-C), and pre-feeding and swallowing (B-D) at four timepoints for two experimental groups.

Airflow pressure increased over time during chewing and swallowing in the volume-reduced group (Figs 10A and 10B). The expiratory pressure was higher during feeding than that in pre-feeding at baseline and week 5. In the volume-enlarged group the airflow pressure was higher at baseline during feeding than that of pre-feeding. During weeks 3 and 5 the airflow pressure in pre-feeding increased, being higher than those during chewing and swallowing (**Fig 10C** and **10D**). No significant differences were found across the 4 timepoints.

## Discussion

In the present study, volumetric changes of the tongue base were induced by extensive growth over time (gradual and systemic) for enlargement animals, and abrupt surgical intervention (acute and focal) for volume reduction animals. Although these two models look asymmetric, they completely minced the clinical conditions with volume-reduced and enlarged tongue base, representing two clinically relevant pathological models that may explain alterations observed in humans. The process of surgical wound healing might have confounded the observed results explaining changes in the muscle activity often seen in the reduction animals, especially in the early time point. Therefore, this important limitation should be considered when the results of the present study are interpreted. An additional confounding factor of the present study is systemic obesity with volume-enlarged animals. Obesity may not only induce an oversized tongue base, but also enlarged oropharyngeal structures, such as the soft palate, pharyngeal walls, and even epiglottis. Thus, the altered consequences in chewing, swallowing, and respiration may have resulted from overall oropharyngeal soft tissue enlargement as well. This limitation should also be emphasized when the results from the enlarged tongue bases are interpreted.

The minipigs in the present study were all same age/sex sibling pairs suitable for the parallel comparisons. Furthermore, the tongue base of minipigs shares multiple similarities with humans including the histological organization and skeletal muscle arrangement [24]. This makes the minipig model comparable to humans to understand mechanisms of various chewing and swallowing disorders. However, the key differences between the minipig and human include: 1) the larger length of the minipig tongue than that of the human; 2) the orientation of the upper airway, which shows horizontal, rather than descending as in humans; and 3) the overlapped position of the epiglottis and soft palate forming a retroglossal space, which is absent in humans This special structural relationship is considered to facilitate nasal breathing in the minipig model during mastication [24–26]. Overall, the minipig is considered as an ideal non-primate animal model with the appropriate size for bio-electronical/biomechanical instrumentations and biomedical study [27].

### Effects of volumetric changes in chewing

In the present study, the jaw opening phase of chewing lasted longer in the tongue reduction group during wound healing at weeks 1 and 3. In the tongue enlargement group the length of this phase consistently increased over time. These increases may be explained by delayed activity timings of the hyoid/pharyngeal and palatal muscles in the reduction group, and later activity timings of the tongue and jaw muscles in the enlargement group (**Fig 3**).

Our previous findings in normal weighted minipigs without ablation of the tongue base showed duration of the phases between 30–40% for jaw opening and 60–70% for jaw closing including the occlusal or power stroke phase [14]. The volume-reduced tongue base underwent inflammatory and proliferative phases of wound healing mainly during weeks 1 and 3. This healing process involves cell migration, differentiation, angiogenesis, and renewal of the extracellular matrix for tissue repair [28–30]. In addition, muscle damage by surgical reduction stimulates satellite cell proliferation, differentiation, and fusion to form new myofibers in the muscular tongue as occur in other injured skeletal muscles and animal models [31–33]. Similarly, the remodeling of the intramuscular extracellular matrix upon damage regulates muscle repair and mechanical properties that may affect contractility [34,35]. The outcome of this recovery depends on factors such as the extension of the injury and characteristics of the muscle, including the embryonic origin [36–38]. In the present study, the size of the wound in the ablated tongue region was 4–8% (1.20 ± 0.48 cm^3^) of the tongue base volume [39,40]. This is nearly half of the tissue loss in tongue base reduction surgeries performed in adult patients with obstructive sleep apnea (3.42 cm^3^) [41].

As the tongue base is the attachment of all intrinsic and extrinsic muscles, any type of dissecting injury may induce fibrosis and functional impairment [42,43]. In a similar way, the infiltration of adipose tissue in the enlarged tongue may induce decreased areas of skeletal muscle with functional impairment related to the fat accumulation as occurred in patients with obstructive sleep apnea [4,44].

The obese minipigs in the present study showed a shortened jaw closing/power stroke phase than those in the reduction group from this study and those in normal weight minipigs previously reported. The findings in the present study indicated later onsets overtime mainly in the activity of the palatal muscles (tensor veli palatini and levator veli palatini) in relation to the zero-point/digastric muscle in the tongue reduction group. In contrast, earlier onsets occurred in the same muscles for the enlarged-tongue group. The activity of the thyrohyoid muscle in both groups also occurred late over time.

All these adaptations are key aspects for airway protection and bolus processing during chewing in the oropharyngeal region [45]. As observed in healthy humans, the healthy minipig model shows spatial changes in movement direction of the pharyngeal structures that substantially differed during respiration, chewing phases and swallowing [26,46,47]. In the present study, volumetric alterations of the tongue base induced delayed onsets, for instance, of the palatal muscles in the volume-reduced group to adapt to such volumetric change. These adaptations in the activity timing may also have influenced adaptations in movement direction.

Patients recovering from tongue reduction surgery have shown potential risks of swallowing disorders that entail higher susceptibility of airway aspiration and dysphagia [48]. The current findings in EMG activity timings may provide certain clues for the mechanism of these potential swallowing disorders.

The amplitudes of muscle activity in the present study were overall higher in the enlargement than those in the reduction group mostly in the thyrohyoid, styloglossus, and masseter muscles. These amplitudes were also larger than those reported in normal weighted minipigs without volumetric changes. The opposite was observed in the reduced tongue base during healing.

In humans, muscle damage secondary to obesity seems to worsen gradually not only due to age but proportionally to the intramuscular extracellular growth of adipose tissue [49,50]. Initially, an overweight condition induces anabolic responses and increases body surface, strength, remodeling, and muscle mass to bear with all physical and metabolic demands [49]. Later, the growing intramuscular fat infiltration (myosteatosis) deteriorates the ability of skeletal muscles to recover, inducing weakened muscle function and loss of muscle mass. These aspects associated with obesity-related metabolic complications cause further muscle damage and a condition known as sarcopenic obesity [49,50]. Similar events occur in masticatory and oropharyngeal muscles in obese adult humans showing initially higher muscle areas compared with individuals with a normal BMI [51,52]. However, the gradual increase of the subcutaneous fat and the BMI produce a decline of the muscle areas and weakening muscle function [52]. Consequently, obese patients exhibit weak mastication, labored breathing, and longer swallowing episodes that worsen over time [52]. The obese minipigs in the present study were young adults at early stages of obesity, which explains why the larger amplitudes seen in muscles such as the hyoid/pharyngeal and palatal during the chewing cycle.

In summary, volumetric changes of the tongue base affect the dynamics of the chewing cycle featured by the delayed activity onsets in palatal and hyoid/pharyngeal muscles, the increased activity amplitudes in tongue, hyoid/pharyngeal, and jaw muscles, and the increased duration of jaw opening at specific timepoints in both conditions with volumetric changes of the tongue base.

### Effects of volumetric changes on swallowing

In the present study the duration of the swallowing cycle was longer in the reduction compared to the enlargement group. Similarly, in relation to the reference muscle, the activity onsets during the swallowing episodes occurred earlier at baseline in the reduction but delayed in the enlargement group in the tongue, jaw and palatal muscles. The opposite occurred after surgery in the reduction group that partly recovered by week 5. In the enlargement group the tensor veli palatini, styloglossus and digastric muscles also showed delayed activity onsets but the changes in the levator veli palatini were minor over time.

All minipigs in the present study were also healing from surgical implantation of ultrasound crystals. Even though analgesia and anti-inflammatory medications were provided, and no tissue was removed, the healing from this additional surgery might disturb the activity of the pharyngeal and jaw muscles observed in the swallowing episodes.

The amplitudes of muscle activity in the enlargement group were higher in the thyrohyoid, tensor veli palatini, and styloglossus than those of the reduction group and the normal weighted minipig. These amplitudes were only similar in the middle pharyngeal constrictor, levator veli palatini, and digastric between the two groups and the normal weighted minipigs. After reduction surgery, the amplitudes decreased in palatal muscles, styloglossus, and thyrohyoid. Interestingly, the amplitude of the levator veli palatini recovered by week 5 to a value close to its baseline.

Proper swallowing episodes in humans imply elevation of the soft palate and retraction of the tongue base [53]. Even though the model in the present study does not entail injury or reduction of the palatal muscles, a smaller tongue base may show less efficient contact surfaces with the soft palate and epiglottis causing alterations of the bolus propulsion. The levator veli palatini is a large muscle of the soft palate that ensures the nasopharyngeal airway patency during swallowing through velar superior/posterior elevations [26,54,55]. Thus, swallowing dysfunction in humans may occur in the first three weeks after surgery also compromising airway patency at its highest risk in the first two days after surgery mainly due to swelling and potential wound bleeding in the first three weeks after surgery. A major risk of swallowing disorders in the reduction group of the present study may also occur during the inflammatory and proliferative phases of wound healing after surgical tongue reduction, when the activities of the related muscles may be delayed and weakened. Again, these findings correlate with post-operative dysphagia after tongue reduction surgery in adults [56].

In summary, specific effects of volumetric changes in swallowing include delayed onset activities and longer cycle duration after reduction, and higher oropharyngeal activity amplitudes of the palatal, hyoid, and tongue muscles in the enlargement group. The effects observed on the activity onsets after surgical tongue reduction raise potential risks for swallowing impairment compromising airway patency.

### Effects of volumetric changes in respiration

The respiratory rates in both experimental groups in the present study was slower than that in normal weighted minipigs previously reported. This is a key indicator of altered respiratory efforts induced by volumetric alterations of the tongue base mainly at week 3. Additional efforts of the oropharyngeal structures occur to coordinate the faster respiratory rate with the altered chewing and swallowing dynamics already present. These additional efforts ensure synchronization and mostly happen in the expiratory phase that may be explained by the larger number of chewing cycles and swallowing events in expiration. Also, there were shortened inspiratory and expiratory phases during swallowing regardless of the volumetric changes observed in the present study.

In the enlargement group the middle pharyngeal constrictor, levator veli palatini, and genioglossus showed weaker activity amplitudes at weeks 1 and 3. These muscles are crucial for the coordination between chewing, swallowing, and respiration to ensure airway patency during feeding. In obese patients, increasing muscle workload and intramuscular adipose infiltration may gradually cause weakening and lack of coordination leading to possible functional disorders, such as choking during chewing or aspiration during swallowing [52,57].

A higher airflow velocity was found in the reduction than enlargement groups and the similar occurred in airflow pressure as well. At week 3, scar tissue is building up in the reduced tongue that may cause aberrant activity changing the feeding dynamics but also the respiratory function during mastication as observed in week 3. In addition, the airflow pressure in swallowing was higher than that in chewing in the reduction group. Clinically, the effort that the oropharynx must apply to propel the bolus to the esophagus may be higher to clear the airway. Therefore, volume-reduced tongue base is more commonly associated with food-sticking that certainly can affect the respiratory pattern overtime observed in the minipigs of the present study. The total air volume per respiratory cycle or tidal volume was also higher in the reduction than enlargement group. In contrast, the enlargement group showed a gradual decrease in the tidal volume over time. This is likely due to the narrow airway secondary to adipose tissue infiltration that causes enlargement of the soft palate and other pharyngeal structures [58]. All these findings indicate that better airway patency may exist in the reduction than enlargement groups and the enhanced airflow pressure during chewing may be a functional adaptation in the enlarged tongue base.

The surgical volumetric reduction of the tongue in children with macroglossia showed improvements in parameters such as oxygen saturation during deep sleeping, indicating the enhanced respiratory dynamics and perfusion after surgery [59]. Although the reduction surgery in the present study was performed in the normal tongue size, the similar improvements of airflow parameters and airway patency were observed.

In summary, major effects of volumetric reduction of the tongue base on respiration were the decreased respiratory rates and increased airflow velocity and pressure. However, the opposite effects were present in the volumetric enlargement of the tongue base with decreased airflow tidal volumes, indicating a potential risk of breathing disorders.

### Functional coordination in volume enlargement and reduction

The evidence in the present study showed a predisposition to aberrant swallowing in the tongue reduction group with enhanced respiratory dynamics. The opposite was observed in the obese minipigs mainly in the respiratory function. These findings confirm the hypothesis that a volume-reduced tongue base will enhance respiratory dynamics but induces irregular swallowing whereas a volume-enlarged tongue base will lead to the opposite effects.

The soft palate elevates during jaw closing/power stroke which results in narrowing of the velopharyngeal space and further moves downwards and anteriorly during jaw opening to expand the airway space [26,46]. This might explain the longer jaw opening phases in chewing observed in the enlargement group. This also explains the enhanced respiratory dynamics detected in the reduction group that presented a shortened length of the jaw opening phase in chewing at week 5. Therefore, the critical timepoints in the reduction group might be at weeks 1 and 3 when narrowing of the airway could have occurred due to inflammation and tissue proliferation that also induced longer length of the jaw opening phase in chewing.

Interestingly, the longer swallowing episodes in the reduction than the enlargement group were seen in the present study. This may be attributed to the muscle wound and healing in the reduction group whereas the shortened lengths of jaw closing/power stroke in the enlargement group might result from the more efficient food transportation during chewing. On the other hand, the larger muscle mass by infiltration of adipose tissue with the need of ensuring synchronized respirations during feeding may predispose to the shortenings of swallowing episodes as well. Therefore, it may be anticipated that later timepoints beyond week 5, animals with enlarged tongue base may have higher risks of dysphagia and airway aspiration. Early evidence found in the enlargement group is the gradual decreasing of airflow tidal volume during swallowing, which could negatively affect proper gas exchange and perfusion due to potential breathing disorders.

In summary, crucial pathophysiological adaptations with volumetric alterations may ensure coordination of chewing, swallowing, and respiration to maintain masticatory function and airway patency. On the other hand, it could lead to the potential dysfunction on this vital oropharyngeal function, such as various swallowing and breathing disorders

## Conclusions

The tongue base volumetric enlargement and reduction affect the dynamics of chewing, swallowing, and respiration. Adaptations to ensure synchronization and coordination during mastication and respiration with airway patency are present in both types of volumetric changes of the tongue base.

Surgical volumetric reduction of the tongue base enhances the respiratory dynamics; however, it may induce swallowing difficulties during the wound healing process with changes in activity durations and amplitudes in the palatal, hyoid/pharyngeal, and tongue muscles. This entails a risk of developing dysphagia if the condition persists.

On the other hand, volumetric enlargement of the tongue base due to obesity affects the chewing cycle dynamics by altering activity onsets in palatal and hyoid muscles and activity amplitudes in tongue and jaw muscles. The major effects in respiration include increased respiratory rate, reduced airflow velocity and pressure, and gradual decrease of airflow tidal volume. These changes may compromise gas exchange and perfusion with the risk of breathing disorders such as obstructive sleep apnea.

## Supporting information

S1 File All datasets EMG experimental. (PDF)
