# Supplementary material for: Muscle activity and airflow dynamics upon gradual weight-gain volumetric enlargement or acute surgical reduction of the tongue base during chewing and swallowing
Source: PLoS One. 2026 Jul 29;21(7):e0352976. doi: 10.1371/journal.pone.0352976 (PMC13419230; doi:10.1371/journal.pone.0352976)
Supplement: S1 File — (PDF) [file pone.0352976.s001.pdf]

										EMG														Inhale burst				Exhale burst					Onsets timing				
ET pig (code)	ID	Group	Timepoint	Muscle	Burst	Function	chewing site	. phases-activation	phases_sites_function	Onset ref	Duration	Mean	Integral	Interval	muscle cycle duration	Time from last chewing	% onset	% duration	Chewing cycle length	Mean (baseline)	egral (base	Duration	Mean Airflow	Mean Pressure	Mean Volume	Tidal volume	Duration	Mean Airflow	Mean Pressure	Mean Volume	Tidal volume	onset inhale	onset exhale	Location (breathing burst)	Delay (seconds)	Interruption (seconds)
7645	2	1	1	2	1	1	1	1	1	.	.	.	.	.	.	.	.	.	.	.	.	.	.	.	.	.	.	.	.	.	.	.	.	.	.	.
7645	2	1	1	2	2	1	2	1	2	.	.	.	.	.	.	.	.	.	.	.	.	.	.	.	.	.	.	.	.	.	.	.	.	.	.	.
7645	2	1	1	2	3	1	1	2	3	.	.	.	.	.	.	.	.	.	.	.	.	.	.	.	.	.	.	.	.	.	.	.	.	.	.	.
7645	2	1	1	2	4	1	2	2	4	.	.	.	.	.	.	.	.	.	.	.	.	.	.	.	.	.	.	.	.	.	.	.	.	.	.	.
7645	2	1	1	3	5	1	1	1	1	.	.	.	.	.	.	.	.	.	.	.	.	.	.	.	.	.	.	.	.	.	.	.	.	.	.	.
7645	2	1	1	3	6	1	2	1	2	.	.	.	.	.	.	.	.	.	.	.	.	.	.	.	.	.	.	.	.	.	.	.	.	.	.	.
7645	2	1	1	3	7	1	1	2	3	.	.	.	.	.	.	.	.	.	.	.	.	.	.	.	.	.	.	.	.	.	.	.	.	.	.	.
7645	2	1	1	3	8	1	2	2	4	.	.	.	.	.	.	.	.	.	.	.	.	.	.	.	.	.	.	.	.	.	.	.	.	.	.	.
7645	2	1	1	4	9	1	1	1	1	.	.	.	.	.	.	.	.	.	.	.	.	.	.	.	.	.	.	.	.	.	.	.	.	.	.	.
7645	2	1	1	4	10	1	2	1	2	.	.	.	.	.	.	.	.	.	.	.	.	.	.	.	.	.	.	.	.	.	.	.	.	.	.	.
7645	2	1	1	4	11	1	1	2	3	.	.	.	.	.	.	.	.	.	.	.	.	.	.	.	.	.	.	.	.	.	.	.	.	.	.	.
7645	2	1	1	4	12	1	2	2	4	.	.	.	.	.	.	.	.	.	.	.	.	.	.	.	.	.	.	.	.	.	.	.	.	.	.	.
7645	2	1	1	6	13	1	1	1	1	.	.	.	.	.	.	.	.	.	.	.	.	.	.	.	.	.	.	.	.	.	.	.	.	.	.	.
7645	2	1	1	6	14	1	2	1	2	.	.	.	.	.	.	.	.	.	.	.	.	.	.	.	.	.	.	.	.	.	.	.	.	.	.	.
7645	2	1	1	6	15	1	1	2	3	.	.	.	.	.	.	.	.	.	.	.	.	.	.	.	.	.	.	.	.	.	.	.	.	.	.	.
7645	2	1	1	6	16	1	2	2	4	.	.	.	.	.	.	.	.	.	.	.	.	.	.	.	.	.	.	.	.	.	.	.	.	.	.	.
7645	2	1	1	7	17	1	1	1	1	.	.	.	.	.	.	.	.	.	.	.	.	.	.	.	.	.	.	.	.	.	.	.	.	.	.	.
7645	2	1	1	7	18	1	2	1	2	.	.	.	.	.	.	.	.	.	.	.	.	.	.	.	.	.	.	.	.	.	.	.	.	.	.	.
7645	2	1	1	7	19	1	1	2	3	.	.	.	.	.	.	.	.	.	.	.	.	.	.	.	.	.	.	.	.	.	.	.	.	.	.	.
7645	2	1	1	7	20	1	2	2	4	.	.	.	.	.	.	.	.	.	.	.	.	.	.	.	.	.	.	.	.	.	.	.	.	.	.	.
7645	2	1	1	8	21	1	1	1	1	.	.	.	.	.	.	.	.	.	.	.	.	.	.	.	.	.	.	.	.	.	.	.	.	.	.	.
7645	2	1	1	8	22	1	2	1	2	.	.	.	.	.	.	.	.	.	.	.	.	.	.	.	.	.	.	.	.	.	.	.	.	.	.	.
7645	2	1	1	8	23	1	1	2	3	.	.	.	.	.	.	.	.	.	.	.	.	.	.	.	.	.	.	.	.	.	.	.	.	.	.	.
7645	2	1	1	8	24	1	2	2	4	.	.	.	.	.	.	.	.	.	.	.	.	.	.	.	.	.	.	.	.	.	.	.	.	.	.	.
7645	2	1	1	10	25	1	1	1	1	.	.	.	.	.	.	.	.	.	.	.	.	.	.	.	.	.	.	.	.	.	.	.	.	.	.	.
7645	2	1	1	10	26	1	2	1	2	.	.	.	.	.	.	.	.	.	.	.	.	.	.	.	.	.	.	.	.	.	.	.	.	.	.	.
7645	2	1	1	10	27	1	1	2	3	.	.	.	.	.	.	.	.	.	.	.	.	.	.	.	.	.	.	.	.	.	.	.	.	.	.	.
7645	2	1	1	10	28	1	2	2	4	.	.	.	.	.	.	.	.	.	.	.	.	.	.	.	.	.	.	.	.	.	.	.	.	.	.	.
7645	2	1	1	11	29	1	1	1	1	.	.	.	.	.	.	.	.	.	.	.	.	.	.	.	.	.	.	.	.	.	.	.	.	.	.	.
7645	2	1	1	11	30	1	2	1	2	.	.	.	.	.	.	.	.	.	.	.	.	.	.	.	.	.	.	.	.	.	.	.	.	.	.	.
7645	2	1	1	11	31	1	1	2	3	.	.	.	.	.	.	.	.	.	.	.	.	.	.	.	.	.	.	.	.	.	.	.	.	.	.	.
7645	2	1	1	11	32	1	2	2	4	.	.	.	.	.	.	.	.	.	.	.	.	.	.	.	.	.	.	.	.	.	.	.	.	.	.	.
7645	2	1	1	2	33	3	3	3	5	.	.	.	.	.	.	.	.	.	.	.	.	.	.	.	.	.	.	.	.	.	.	.	.	.	.	.
7645	2	1	1	3	34	3	3	3	5	.	.	.	.	.	.	.	.	.	.	.	.	.	.	.	.	.	.	.	.	.	.	.	.	.	.	.
7645	2	1	1	4	35	3	3	3	5	.	.	.	.	.	.	.	.	.	.	.	.	.	.	.	.	.	.	.	.	.	.	.	.	.	.	.
7645	2	1	1	6	36	3	3	3	5	.	.	.	.	.	.	.	.	.	.	.	.	.	.	.	.	.	.	.	.	.	.	.	.	.	.	.
7645	2	1	1	7	37	3	3	3	5	.	.	.	.	.	.	.	.	.	.	.	.	.	.	.	.	.	.	.	.	.	.	.	.	.	.	.
7645	2	1	1	8	38	3	3	3	5	.	.	.	.	.	.	.	.	.	.	.	.	.	.	.	.	.	.	.	.	.	.	.	.	.	.	.
7645	2	1	1	10	39	3	3	3	5	.	.	.	.	.	.	.	.	.	.	.	.	.	.	.	.	.	.	.	.	.	.	.	.	.	.	.
7645	2	1	1	11	40	3	3	3	5	.	.	.	.	.	.	.	.	.	.	.	.	.	.	.	.	.	.	.	.	.	.	.	.	.	.	.
7645	2	1	2	2	1	1	1	1	1	.	.	.	.	.	.	.	.	.	.	.	.	.	.	.	.	.	.	.	.	.	.	.	.	.	.	.
7645	2	1	2	2	2	1	2	1	2	.	.	.	.	.	.	.	.	.	.	.	.	.	.	.	.	.	.	.	.	.	.	.	.	.	.	.
7645	2	1	2	2	3	1	1	2	3	.	.	.	.	.	.	.	.	.	.	.	.	.	.	.	.	.	.	.	.	.	.	.	.	.	.	.
7645	2	1	2	2	4	1	2	2	4	.	.	.	.	.	.	.	.	.	.	.	.	.	.	.	.	.	.	.	.	.	.	.	.	.	.	.
7645	2	1	2	3	5	1	1	1	1	.	.	.	.	.	.	.	.	.	.	.	.	.	.	.	.	.	.	.	.	.	.	.	.	.	.	.
7645	2	1	2	3	6	1	2	1	2	.	.	.	.	.	.	.	.	.	.	.	.	.	.	.	.	.	.	.	.	.	.	.	.	.	.	.
7645	2	1	2	3	7	1	1	2	3	.	.	.	.	.	.	.	.	.	.	.	.	.	.	.	.	.	.	.	.	.	.	.	.	.	.	.
7645	2	1	2	3	8	1	2	2	4	.	.	.	.	.	.	.	.	.	.	.	.	.	.	.	.	.	.	.	.	.	.	.	.	.	.	.
7645	2	1	2	4	9	1	1	1	1	.	.	.	.	.	.	.	.	.	.	.	.	.	.	.	.	.	.	.	.	.	.	.	.	.	.	.
7645	2	1	2	4	10	1	2	1	2	.	.	.	.	.	.	.	.	.	.	.	.	.	.	.	.	.	.	.	.	.	.	.	.	.	.	.
7645	2	1	2	4	11	1	1	2	3	.	.	.	.	.	.	.	.	.	.	.	.	.	.	.	.	.	.	.	.	.	.	.	.	.	.	.
7645	2	1	2	4	12	1	2	2	4	.	.	.	.	.	.	.	.	.	.	.	.	.	.	.	.	.	.	.	.	.	.	.	.	.	.	.
7645	2	1	2	6	13	1	1	1	1	.	.	.	.	.	.	.	.	.	.	.	.	.	.	.	.	.	.	.	.	.	.	.	.	.	.	.
7645	2	1	2	6	14	1	2	1	2	.	.	.	.	.	.	.	.	.	.	.	.	.	.	.	.	.	.	.	.	.	.	.	.	.	.	.
7645	2	1	2	6	15	1	1	2	3	.	.	.	.	.	.	.	.	.	.	.	.	.	.	.	.	.	.	.	.	.	.	.	.	.	.	.
7645	2	1	2	6	16	1	2	2	4	.	.	.	.	.	.	.	.	.	.	.	.	.	.	.	.	.	.	.	.	.	.	.	.	.	.	.
7645	2	1	2	7	17	1	1	1	1	.	.	.	.	.	.	.	.	.	.	.	.	.	.	.	.	.	.	.	.	.	.	.	.	.	.	.
7645	2	1	2	7	18	1	2	1	2	.	.	.	.	.	.	.	.	.	.	.	.	.	.	.	.	.	.	.	.	.	.	.	.	.	.	.
7645	2	1	2	7	19	1	1	2	3	.	.	.	.	.	.	.	.	.	.	.	.	.	.	.	.	.	.	.	.	.	.	.	.	.	.	.
7645	2	1	2	7	20	1	2	2	4	.	.	.	.	.	.	.	.	.	.	.	.	.	.	.	.	.	.	.	.	.	.	.	.	.	.	.
7645	2	1	2	8	21	1	1	1	1	.	.	.	.	.	.	.	.	.	.	.	.	.	.	.	.	.	.	.	.	.	.	.	.	.	.	.
7645	2	1	2	8	22	1	2	1	2	.	.	.	.	.	.	.	.	.	.	.	.	.	.	.	.	.	.	.	.	.	.	.	.	.	.	.
7645	2	1	2	8	23	1	1	2	3	.	.	.	.	.	.	.	.	.	.	.	.	.	.	.	.	.	.	.	.	.	.	.	.	.	.	.
7645	2	1	2	8	24	1	2	2	4	.	.	.	.	.	.	.	.	.	.	.	.	.	.	.	.	.	.	.	.	.	.	.	.	.	.	.
7645	2	1	2	10	25	1	1	1	1	.	.	.	.	.	.	.	.	.	.	.	.	.	.	.	.	.	.	.	.	.	.	.	.	.	.	.
7645	2	1	2	10	26	1	2	1	2	.	.	.	.	.	.	.	.	.	.	.	.	.	.	.	.	.	.	.	.	.	.	.	.	.	.	.
7645	2	1	2	10	27	1	1	2	3	.	.	.	.	.	.	.	.	.	.	.	.	.	.	.	.	.	.	.	.	.	.	.	.	.	.	.
7645	2	1	2	10	28	1	2	2	4	.	.	.	.	.	.	.	.	.	.	.	.	.	.	.	.	.	.	.	.	.	.	.	.	.	.	.
7645	2	1	2	11	29	1	1	1	1	.	.	.	.	.	.	.	.	.	.	.	.	.	.	.	.	.	.	.	.	.	.	.	.	.	.	.
7645	2	1	2	11	30	1	2	1	2	.	.	.	.	.	.	.	.	.	.	.	.	.	.	.	.	.	.	.	.	.	.	.	.	.	.	.
7645	2	1	2	11	31	1	1	2	3	.	.	.	.	.	.	.	.	.	.	.	.	.	.	.	.	.	.	.	.	.	.	.	.	.	.	.
7645	2	1	2	11	32	1	2	2	4	.	.	.	.	.	.	.	.	.	.	.	.	.	.	.	.	.	.	.	.	.	.	.	.	.	.	.
7645	2	1	2	2	33	3	3	3	5	.	.	.	.	.	.	.	.	.	.	.	.	.	.	.	.	.	.	.	.	.	.	.	.	.	.	.
7645	2	1	2	3	34	3	3	3	5	.	.	.	.	.	.	.	.	.	.	.	.	.	.	.	.	.	.	.	.	.	.	.	.	.	.	.
7645	2	1	2	4	35	3	3	3	5	.	.	.	.	.	.	.	.	.	.	.	.	.	.	.	.	.	.	.	.	.	.	.	.	.	.	.
7645	2	1	2	6	36	3	3	3	5	.	.	.	.	.	.	.	.	.	.	.	.	.	.	.	.	.	.	.	.	.	.	.	.	.	.	.
7645	2	1	2	7	37	3	3	3	5	.	.	.	.	.	.	.	.	.	.	.	.	.	.	.	.	.	.	.	.	.	.	.	.	.	.	.
7645	2	1	2	8	38	3	3	3	5	.	.	.	.	.	.	.	.	.	.	.	.	.	.	.	.	.	.	.	.	.	.	.	.	.	.	.
7645	2	1	2	10	39	3	3	3	5	.	.	.	.	.	.	.	.	.	.	.	.	.	.	.	.	.	.	.	.	.	.	.	.	.	.	.
7645	2	1	2	11	40	3	3	3	5	.	.	.	.	.	.	.	.	.	.	.	.	.	.	.	.	.	.	.	.	.	.	.	.	.	.	.
7645	2	1	3	2	1	1	1	1	1	-0.015	0.167	0.040	0.004	0.101	0.268	.	-0.049	0.267	0.554	0.003	0.001	0.658	9.784	0.109	4.392	510.287	1.480	9.861	0.107	4.463	520.326	-0.646	-0.033	2	.	.
7645	2	1	3	2	2	1	2	1	2	0.078	0.142	0.023	0.003	0.020	0.162	.	0.128	0.224	0.554	0.003	0.001	0.658	9.784	0.109	4.392	510.287	1.480	9.861	0.107	4.463	520.326	-0.646	-0.033	2	.	.
7645	2	1	3	2	3	1	1	2	3	0.250	0.129	0.068	0.007	0.012	0.140	.	0.414	0.221	0.554	0.004	0.001	0.658	9.784	0.109	4.392	510.287	1.480	9.861	0.107	4.463	520.326	-0.646	-0.033	2	.	.
7645	2	1	3	2	4	1	2	2	4	0.446	0.104	0.032	0.003	0.052	0.155	.	0.686	0.162	0.554	0.003	0.001	0.658	9.784	0.109	4.392	510.287	1.480	9.861	0.107	4.463	520.326	-0.646	-0.033	2	.	.
7645	2	1	3	3	5	1	1	1	1	0.034	0.123	0.150	0.016	0.048	0.170	.	0.028	0.227	0.554	0.010	0.001	0.658	9.784	0.109	4.392	510.287	1.480	9.861	0.107	4.463	520.326	-0.646	-0.033	2	.	.
7645	2	1	3	3	6	1	2	1	2	0.026	0.126	0.107	0.011	0.022	0.148	.	0.053	0.224	0.554	0.010	0.001	0.658	9.784	0.109	4.392	510.287	1.480	9.861	0.107	4.463	520.326	-0.646	-0.033	2	.	.
7645	2	1	3	3	7	1	1	2	3	0.282	0.133	0.154	0.020	0.010	0.143	.	0.503	0.240	0.554	0.010	0.001	0.658	9.784	0.109	4.392	510.287	1.480	9.861	0.107	4.463	520.326	-0.646	-0.033	2	.	.
7645	2	1	3	3	8	1	2	2	4	0.293	0.132	0.106	0.011	0.063	0.195	.	0.503	0.242	0.554	0.010	0.001	0.658	9.784	0.109	4.392	510.287	1.480	9.861	0.107	4.463	520.326	-0.646	-0.033	2	.	.
7645	2	1	3	4	9	1	1	1	1	-0.008	0.143	0.231	0.025	0.043	0.186	.	-0.007	0.260	0.554	0.033	0.001	0.658	9.784	0.109	4.392	510.287	1.480	9.861	0.107	4.463	520.326	-0.646	-0.033	2	.	.
7645	2	1	3	4	10	1	2	1	2	0.102	0.127	0.159	0.019	0.032	0.159	.	0.176	0.236	0.554	0.033	0.001	0.658	9.784	0.109	4.392	510.287	1.480	9.861	0.107	4.463	520.326	-0.646	-0.033	2	.	.
7645	2	1	3	4	11	1	1	2	3	0.323	0.129	0.317	0.035	0.025	0.155	.	0.571	0.240	0.554	0.033	0.001	0.658	9.784	0.109	4.392	510.287	1.480	9.861	0.107	4.463	520.326	-0.646	-0.033	2	.	.
7645	2	1	3	4	12	1	2	2	4	0.355	0.128	0.171	0.021	0.024	0.152	.	0.662	0.233	0.554	0.033	0.001	0.658	9.784	0.109	4.392	510.287	1.480	9.861	0.107	4.463	520.326	-0.646	-0.033	2	.	.
7645	2	1	3	6	13	1	1	1	1	-0.056	0.133	0.157	0.015	0.042	0.174	.	-0.129	0.249	0.554	0.003	0.001	0.658	9.784	0.109	4.392	510.287	1.480	9.861	0.107	4.463	520.326	-0.646	-0.033	2	.	.
7645	2	1	3	6	14	1	2	1	2	0.030	0.117	0.078	0.008	0.040	0.157	.	0.014	0.218	0.554	0.003	0.001	0.658	9.784	0.109	4.392	510.287	1.480	9.861	0.107	4.463	520.326	-0.646	-0.033	2	.	.
7645	2	1	3	6	15	1	1	2	3	0.163	0.134	0.221	0.022	0.018	0.151	.	0.259	0.246	0.554	0.003	0.001	0.658	9.784	0.109	4.392	510.287	1.480	9.861	0.107	4.463	520.326	-0.646	-0.033	2	.	.
7645	2	1	3	6	16	1	2	2	4	0.316	0.143	0.080	0.008	0.039	0.182	.	0.543	0.270	0.554	0.003	0.001	0.658	9.784	0.109	4.392	510.287	1.480	9.861	0.107	4.463	520.326	-0.646	-0.033	2	.	.
7645	2	1	3	7	17	1	1	1	1	-0.076	0.127	0.086	0.009	0.043	0.170	.	-0.165	0.232	0.554	0.004	0.001	0.658	9.784	0.109	4.392	510.287	1.480	9.861	0.107	4.463	520.326	-0.646	-0.033	2	.	.
7645	2	1	3	7	18	1	2	1	2	-0.041	0.120	0.063	0.007	0.026	0.146	.	-0.105	0.217	0.554	0.004	0.001	0.658	9.784	0.109	4.392	510.287	1.480	9.861	0.107	4.463	520.326	-0.646	-0.033	2	.	.
7645	2	1	3	7	19	1	1	2	3	.	.	.	.	.	.	.	.	.	.	.	.	.	.	.	.	.	.	.	.	.	.	.	.	.	.	.
7645	2	1	3	7	20	1	2	2	4	.	.	.	.	.	.	.	.	.	.	.	.	.	.	.	.	.	.	.	.	.	.	.	.	.	.	.
7645	2	1	3	8	21	1	1	1	1	0.031	0.126	0.299	0.031	0.027	0.153	.	0.043	0.232	0.554	0.034	0.001	0.658	9.784	0.109	4.392	510.287	1.480	9.861	0.107	4.463	520.326	-0.646	-0.033	2	.	.
7645	2	1	3	8	22	1	2	1	2	0.080	0.120	0.148	0.017	0.037	0.157	.	0.122	0.227	0.554	0.034	0.001	0.658	9.784	0.109	4.392	510.287	1.480	9.861	0.107	4.463	520.326	-0.646	-0.033	2	.	.
7645	2	1	3	8	23	1	1	2	3	0.303	0.135	0.198	0.026	0.026	0.161	.	0.550	0.248	0.554	0.034	0.001	0.658	9.784	0.109	4.392	510.287	1.480	9.861	0.107	4.463	520.326	-0.646	-0.033	2	.	.
7645	2	1	3	8	24	1	2	2	4	0.358	0.126	0.141	0.014	0.032	0.158	.	0.639	0.236	0.554	0.034	0.001	0.658	9.784	0.109	4.392	510.287	1.480	9.861	0.107	4.463	520.326	-0.646	-0.033	2	.	.
7645	2	1	3	10	25	1	1	1	1	.	.	.	.	.	.	.	.	.	.	.	.	.	.	.	.	.	.	.	.	.	.	.	.	.	.	.
7645	2	1	3	10	26	1	2	1	2	.	.	.	.	.	.	.	.	.	.	.	.	.	.	.	.	.	.	.	.	.	.	.	.	.	.	.
7645	2	1	3	10	27	1	1	2	3	0.278	0.135	0.143	0.014	0.024	0.159	.	0.464	0.250	0.554	0.007	0.001	0.658	9.784	0.109	4.392	510.287	1.480	9.861	0.107	4.463	520.326	-0.646	-0.033	2	.	.
7645	2	1	3	10	28	1	2	2	4	0.341	0.114	0.076	0.008	0.040	0.155	.	0.611	0.212	0.554	0.007	0.001	0.658	9.784	0.109	4.392	510.287	1.480	9.861	0.107	4.463	520.326	-0.646	-0.033	2	.	.
7645	2	1	3	11	29	1	1	1	1	0.000	0.135	0.265	0.026	0.059	0.194	.	0.001	0.242	0.554	0.005	0.001	0.658	9.784	0.109	4.392	510.287	1.480	9.861	0.107	4.463	520.326	-0.646	-0.033	2	.	.
7645	2	1	3	11	30	1	2	1	2	0.061	0.129	0.118	0.012	0.025	0.154	.	0.101	0.230	0.554	0.005	0.001	0.658	9.784	0.109	4.392	510.287	1.480	9.861	0.107	4.463	520.326	-0.646	-0.033	2	.	.
7645	2	1	3	11	31	1	1	2	3	0.324	0.131	0.287	0.034	0.040	0.171	.	0.563	0.237	0.554	0.005	0.001	0.658	9.784	0.109	4.392	510.287	1.480	9.861	0.107	4.463	520.326	-0.646	-0.033	2	.	.
7645	2	1	3	11	32	1	2	2	4	0.253	0.133	0.123	0.013	0.019	0.152	.	0.456	0.238	0.554	0.005	0.001	0.658	9.784	0.109	4.392	510.287	1.480	9.861	0.107	4.463	520.326	-0.646	-0.033	2	.	.
7645	2	1	3	2	33	3	3	3	5	-0.040	0.262	0.201	0.018	0.000	0.262	.	-0.056	0.868	0.290	0.004	0.001	0.463	9.824	0.108	4.779	503.153	0.858	9.915	0.109	4.712	512.291	-1.286	-0.823	2	.	.
7645	2	1	3	3	34	3	3	3	5	0.000	0.281	0.140	0.007	0.000	0.281	1.495	0.001	0.960	0.290	0.004	0.001	0.463	9.824	0.108	4.779	503.153	0.858	9.915	0.109	4.712	512.291	-1.286	-0.823	2	.	0.845
7645	2	1	3	4	35	3	3	3	5	-0.043	0.201	0.115	0.016	0.000	0.201	.	-0.075	0.709	0.290	0.004	0.001	0.463	9.824	0.108	4.779	503.153	0.858	9.915	0.109	4.712	512.291	-1.286	-0.823	2	.	.
7645	2	1	3	6	36	3	3	3	5	-0.068	0.307	0.123	0.023	0.000	0.307	.	-0.137	1.034	0.290	0.004	0.001	0.463	9.824	0.108	4.779	503.153	0.858	9.915	0.109	4.712	512.291	-1.286	-0.823	2	.	.
7645	2	1	3	7	37	3	3	3	5	.	.	.	.	.	.	.	.	.	.	.	.	.	.	.	.	.	.	.	.	.	.	.	.	.	.	.
7645	2	1	3	8	38	3	3	3	5	-0.059	0.234	0.146	0.017	0.000	0.234	.	0.034	0.803	0.290	0.004	0.001	0.463	9.824	0.108	4.779	503.153	0.858	9.915	0.109	4.712	512.291	-1.286	-0.823	2	.	.
7645	2	1	3	10	39	3	3	3	5	.	.	.	.	.	.	.	.	.	.	.	.	.	.	.	.	.	.	.	.	.	.	.	.	.	.	.
7645	2	1	3	11	40	3	3	3	5	-0.062	0.167	0.124	0.008	0.000	0.167	.	-0.107	0.610	0.290	0.004	0.001	0.463	9.824	0.108	4.779	503.153	0.858	9.915	0.109	4.712	512.291	-1.286	-0.823	2	.	.
7645	2	1	4	2	1	1	1	1	1	-0.061	0.167	-0.047	-0.010	-0.078	-0.245	.	-0.154	-0.345	0.484	-0.003	-0.001	-0.736	-7.375	-0.130	-1.888	-161.386	-1.504	-7.506	-0.129	-2.030	-170.137	0.677	-0.059	2	.	.
7645	2	1	4	2	2	1	2	1	2	-0.243	0.112	-0.023	-0.002	-0.031	-0.142	.	-0.480	-0.226	0.496	-0.003	-0.001	-0.736	-7.375	-0.130	-1.888	-161.386	-1.504	-7.506	-0.129	-2.030	-170.137	0.677	-0.059	2	.	.
7645	2	1	4	2	3	1	1	2	3	-0.130	0.167	-0.090	-0.008	-0.011	-0.178	.	-0.290	-0.349	0.484	-0.003	-0.001	-0.736	-7.375	-0.130	-1.888	-161.386	-1.504	-7.506	-0.129	-2.030	-170.137	0.677	-0.059	2	.	.
7645	2	1	4	2	4	1	2	2	4	-0.413	0.122	-0.040	-0.004	-0.015	-0.137	.	0.853	0.252	0.484	-0.003	-0.001	-0.736	-7.375	-0.130	-1.888	-161.386	-1.504	-7.506	-0.129	-2.030	-170.137	0.677	-0.059	2	.	.
7645	2	1	4	3	5	1	1	1	1	0.000	0.217	-0.020	-0.002	0.000	-0.217	.	-0.001	-0.448	0.484	-0.002	-0.001	-0.736	-7.375	-0.130	-1.888	-161.386	-1.504	-7.506	-0.129	-2.030	-170.137	0.677	-0.059	2	.	.
7645	2	1	4	3	6	1	2	1	2	.	.	.	.	.	.	.	.	.	.	.	.	.	.	.	.	.	.	.	.	.	.	.	.	.	.	.
7645	2	1	4	3	7	1	1	2	3	.	.	.	.	.	.	.	.	.	.	.	.	.	.	.	.	.	.	.	.	.	.	.	.	.	.	.
7645	2	1	4	3	8	1	2	2	4	.	.	.	.	.	.	.	.	.	.	.	.	.	.	.	.	.	.	.	.	.	.	.	.	.	.	.
7645	2	1	4	4	9	1	1	1	1	-0.204	0.128	-0.191	-0.017	-0.064	-0.192	.	-0.415	-0.265	0.490	-0.005	-0.001	-0.736	-7.375	-0.130	-1.888	-161.386	-1.504	-7.506	-0.129	-2.030	-170.137	0.677	-0.059	2	.	.
7645	2	1	4	4	10	1	2	1	2	-0.047	0.148	-0.036	-0.004	-0.042	-0.189	.	-0.130	-0.274	0.507	-0.005	-0.001	-0.736	-7.375	-0.130	-1.888	-161.386	-1.504	-7.506	-0.129	-2.030	-170.137	0.677	-0.059	2	.	.
7645	2	1	4	4	11	1	1	2	3	-0.322	0.160	-0.138	-0.011	-0.013	-0.173	.	-0.653	-0.330	0.490	-0.005	-0.001	-0.736	-7.375	-0.130	-1.888	-161.386	-1.504	-7.506	-0.129	-2.030	-170.137	0.677	-0.059	2	.	.
7645	2	1	4	4	12	1	2	2	4	-0.602	0.100	-0.092	-0.006	-0.024	-0.124	.	-0.548	-0.209	0.507	-0.005	-0.001	-0.736	-7.375	-0.130	-1.888	-161.386	-1.504	-7.506	-0.129	-2.030	-170.137	0.677	-0.059	2	.	.
7645	2	1	4	6	13	1	1	1	1	0.031	0.182	-0.282	-0.061	-0.121	-0.303	.	0.059	-0.371	0.490	-0.004	-0.001	-0.736	-7.375	-0.130	-1.888	-161.386	-1.504	-7.506	-0.129	-2.030	-170.137	0.677	-0.059	2	.	.
7645	2	1	4	6	14	1	2	1	2	-0.173	0.148	-0.250	-0.035	-0.053	-0.200	.	-0.340	-0.288	0.513	-0.004	-0.001	-0.736	-7.375	-0.130	-1.888	-161.386	-1.504	-7.506	-0.129	-2.030	-170.137	0.677	-0.059	2	.	.
7645	2	1	4	6	15	1	1	2	3	-0.281	0.170	-0.285	-0.033	-0.018	-0.188	.	-0.572	-0.351	0.490	-0.004	-0.001	-0.736	-7.375	-0.130	-1.888	-161.386	-1.504	-7.506	-0.129	-2.030	-170.137	0.677	-0.059	2	.	.
7645	2	1	4	6	16	1	2	2	4	-0.335	0.189	-0.062	-0.011	0.000	-0.189	.	-0.647	-0.365	0.518	-0.004	-0.001	-0.736	-7.375	-0.130	-1.888	-161.386	-1.504	-7.506	-0.129	-2.030	-170.137	0.677	-0.059	2	.	.

7645	2	1	4	7	17	1	1	1	1	0.015	0.160	-0.200	-0.020	-0.025	-0.185	.	0.027	-0.326	0.490	-0.002	-0.001	-0.736	-7.375	-0.130	-1.888	-161.386	-1.504	-7.506	-0.129	-2.030	-170.137	0.677	-0.059	2	.	.
7645	2	1	4	7	18	1	2	1	2	-0.065	0.180	-0.093	-0.009	-0.029	-0.209	.	-0.134	-0.367	0.490	-0.002	-0.001	-0.736	-7.375	-0.130	-1.888	-161.386	-1.504	-7.506	-0.129	-2.030	-170.137	0.677	-0.059	2	.	.
7645	2	1	4	7	19	1	1	2	3	.	.	.	.	.	.	.	.	.	.	.	.	.	.	.	.	.	.	.	.	.	.	.	.	.	.	.
7645	2	1	4	7	20	1	2	2	4	.	.	.	.	.	.	.	.	.	.	.	.	.	.	.	.	.	.	.	.	.	.	.	.	.	.	.
7645	2	1	4	8	21	1	1	1	1	-0.077	0.163	-0.168	-0.025	-0.035	-0.198	.	-0.152	-0.334	0.490	-0.013	-0.001	-0.736	-7.375	-0.130	-1.888	-161.386	-1.504	-7.506	-0.129	-2.030	-170.137	0.677	-0.059	2	.	.
7645	2	1	4	8	22	1	2	1	2	-0.176	0.108	-0.167	-0.012	-0.025	-0.133	.	-0.341	-0.211	0.513	-0.013	-0.001	-0.736	-7.375	-0.130	-1.888	-161.386	-1.504	-7.506	-0.129	-2.030	-170.137	0.677	-0.059	2	.	.
7645	2	1	4	8	23	1	1	2	3	-0.361	0.171	-0.223	-0.024	0.000	-0.171	.	-0.726	-0.354	0.490	-0.013	-0.001	-0.736	-7.375	-0.130	-1.888	-161.386	-1.504	-7.506	-0.129	-2.030	-170.137	0.677	-0.059	2	.	.
7645	2	1	4	8	24	1	2	2	4	-0.362	0.117	-0.114	-0.013	-0.051	-0.168	.	-0.714	-0.231	0.507	-0.013	-0.001	-0.736	-7.375	-0.130	-1.888	-161.386	-1.504	-7.506	-0.129	-2.030	-170.137	0.677	-0.059	2	.	.
7645	2	1	4	10	25	1	1	1	1	.	.	.	.	.	.	.	.	.	.	.	.	.	.	.	.	.	.	.	.	.	.	.	.	.	.	.
7645	2	1	4	10	26	1	2	1	2	.	.	.	.	.	.	.	.	.	.	.	.	.	.	.	.	.	.	.	.	.	.	.	.	.	.	.
7645	2	1	4	10	27	1	1	2	3	-0.333	0.167	-0.158	-0.016	0.000	-0.167	.	-0.675	-0.345	0.490	-0.003	-0.001	-0.736	-7.375	-0.130	-1.888	-161.386	-1.504	-7.506	-0.129	-2.030	-170.137	0.677	-0.059	2	.	.
7645	2	1	4	10	28	1	2	2	4	-0.306	0.103	-0.061	-0.006	-0.028	-0.131	.	-0.598	-0.201	0.513	-0.003	-0.001	-0.736	-7.375	-0.130	-1.888	-161.386	-1.504	-7.506	-0.129	-2.030	-170.137	0.677	-0.059	2	.	.
7645	2	1	4	11	29	1	1	1	1	0.000	0.200	-0.283	-0.026	-0.099	-0.299	.	-0.001	-0.408	0.490	-0.002	-0.001	-0.736	-7.375	-0.130	-1.888	-161.386	-1.504	-7.506	-0.129	-2.030	-170.137	0.677	-0.059	2	.	.
7645	2	1	4	11	30	1	2	1	2	.	.	.	.	.	.	.	.	.	.	.	.	.	.	.	.	.	.	.	.	.	.	.	.	.	.	.
7645	2	1	4	11	31	1	1	2	3	-0.268	0.199	-0.027	-0.003	0.000	-0.199	.	-0.552	-0.411	0.484	-0.002	-0.001	-0.736	-7.375	-0.130	-1.888	-161.386	-1.504	-7.506	-0.129	-2.030	-170.137	0.677	-0.059	2	.	.
7645	2	1	4	11	32	1	2	2	4	.	.	.	.	.	.	.	.	.	.	.	.	.	.	.	.	.	.	.	.	.	.	.	.	.	.	.
7645	2	1	4	2	33	3	3	3	5	-0.019	0.162	0.098	0.003	0.000	0.162	0.491	-0.118	1.064	0.151	0.002	0.001	0.736	7.375	0.130	1.888	161.386	1.504	7.506	0.129	2.030	170.137	-1.340	-0.605	2	.	.
7645	2	1	4	3	34	3	3	3	5	0.000	0.135	0.025	0.003	0.000	0.135	0.790	0.001	0.908	0.151	0.002	0.001	0.736	7.375	0.130	1.888	161.386	1.504	7.506	0.129	2.030	170.137	-1.323	-0.588	2	3.888	0.320
7645	2	1	4	4	35	3	3	3	5	-0.045	0.148	0.102	0.008	0.000	0.148	.	-0.238	1.000	0.151	0.002	0.001	0.736	7.375	0.130	1.888	161.386	1.504	7.506	0.129	2.030	170.137	-1.323	-0.588	2	.	.
7645	2	1	4	6	36	3	3	3	5	-0.042	0.134	0.236	0.023	0.000	0.134	.	-0.311	0.919	0.151	0.002	0.001	0.736	7.375	0.130	1.888	161.386	1.504	7.506	0.129	2.030	170.137	-1.323	-0.588	2	.	.
7645	2	1	4	7	37	3	3	3	5	.	.	.	.	.	.	.	.	.	.	.	.	.	.	.	.	.	.	.	.	.	.	.	.	.	.	.
7645	2	1	4	8	38	3	3	3	5	-0.072	0.123	0.334	0.061	0.000	0.123	.	-0.527	0.833	0.151	0.002	0.001	0.736	7.375	0.130	1.888	161.386	1.504	7.506	0.129	2.030	170.137	-1.323	-0.588	2	.	.
7645	2	1	4	10	39	3	3	3	5	.	.	.	.	.	.	.	.	.	.	.	.	.	.	.	.	.	.	.	.	.	.	.	.	.	.	.
7645	2	1	4	11	40	3	3	3	5	0.002	0.159	0.062	0.006	0.000	0.159	.	-0.003	1.061	0.151	0.002	0.001	0.736	7.375	0.130	1.888	161.386	1.504	7.506	0.129	2.030	170.137	-1.323	-0.588	2	.	.
7658	2	2	1	2	1	1	1	1	1	0.184	0.245	0.037	0.002	0.039	0.284	.	0.237	0.316	0.669	0.001	0.001	0.706	6.067	0.008	0.636	207.431	1.562	5.993	0.006	0.716	214.570	-0.694	0.011	2	.	.
7658	2	2	1	2	2	1	2	1	2	0.056	0.145	0.020	0.002	0.033	0.178	.	0.072	0.187	0.669	0.001	0.001	0.706	6.067	0.008	0.636	207.431	1.562	5.993	0.006	0.716	214.570	-0.694	0.011	2	.	.
7658	2	2	1	2	3	1	1	2	3	0.530	0.133	0.010	0.002	0.051	0.184	.	0.684	0.172	0.669	0.001	0.001	0.706	6.067	0.008	0.636	207.431	1.562	5.993	0.006	0.716	214.570	-0.694	0.011	2	.	.
7658	2	2	1	2	4	1	2	2	4	0.691	0.101	0.005	0.002	0.000	0.101	.	0.892	0.130	0.669	0.001	0.001	0.706	6.067	0.008	0.636	207.431	1.562	5.993	0.006	0.716	214.570	-0.694	0.011	2	.	.
7658	2	2	1	3	5	1	1	1	1	0.315	0.184	0.483	0.102	0.058	0.242	.	0.513	0.274	0.669	0.001	0.001	0.706	6.067	0.008	0.636	207.431	1.562	5.993	0.006	0.716	214.570	-0.694	0.011	2	.	.
7658	2	2	1	3	6	1	2	1	2	0.185	0.125	0.082	0.007	0.030	0.155	.	0.313	0.191	0.669	0.001	0.001	0.706	6.067	0.008	0.636	207.431	1.562	5.993	0.006	0.716	214.570	-0.694	0.011	2	.	.
7658	2	2	1	3	7	1	1	2	3	0.579	0.137	0.228	0.042	0.012	0.149	.	0.902	0.207	0.669	0.001	0.001	0.706	6.067	0.008	0.636	207.431	1.562	5.993	0.006	0.716	214.570	-0.694	0.011	2	.	.
7658	2	2	1	3	8	1	2	2	4	0.608	0.117	0.061	0.007	0.019	0.136	.	0.976	0.182	0.669	0.001	0.001	0.706	6.067	0.008	0.636	207.431	1.562	5.993	0.006	0.716	214.570	-0.694	0.011	2	.	.
7658	2	2	1	4	9	1	1	1	1	0.111	0.169	0.290	0.051	0.083	0.252	.	0.165	0.251	0.676	0.010	0.001	0.706	6.067	0.008	0.636	207.431	1.562	5.993	0.006	0.716	214.570	-0.694	0.011	2	.	.
7658	2	2	1	4	10	1	2	1	2	0.064	0.175	0.169	0.029	0.044	0.219	.	0.125	0.256	0.676	0.010	0.001	0.706	6.067	0.008	0.636	207.431	1.562	5.993	0.006	0.716	214.570	-0.694	0.011	2	.	.
7658	2	2	1	4	11	1	1	2	3	0.503	0.145	0.233	0.030	0.023	0.168	.	0.743	0.221	0.676	0.010	0.001	0.706	6.067	0.008	0.636	207.431	1.562	5.993	0.006	0.716	214.570	-0.694	0.011	2	.	.
7658	2	2	1	4	12	1	2	2	4	0.534	0.162	0.178	0.029	0.029	0.191	.	0.807	0.246	0.676	0.009	0.001	0.706	6.067	0.008	0.636	207.431	1.562	5.993	0.006	0.716	214.570	-0.694	0.011	2	.	.
7658	2	2	1	6	13	1	1	1	1	0.009	0.199	0.185	0.025	0.048	0.247	.	0.036	0.294	0.676	0.019	0.001	0.706	6.067	0.008	0.636	207.431	1.562	5.993	0.006	0.716	214.570	-0.694	0.011	2	.	.
7658	2	2	1	6	14	1	2	1	2	0.036	0.152	0.116	0.012	0.043	0.195	.	-0.046	0.229	0.666	0.019	0.001	0.706	6.067	0.008	0.636	207.431	1.562	5.993	0.006	0.716	214.570	-0.694	0.011	2	.	.
7658	2	2	1	6	15	1	1	2	3	0.413	0.140	0.170	0.033	0.024	0.165	.	0.649	0.208	0.676	0.019	0.001	0.706	6.067	0.008	0.636	207.431	1.562	5.993	0.006	0.716	214.570	-0.694	0.011	2	.	.
7658	2	2	1	6	16	1	2	2	4	0.464	0.116	0.105	0.010	0.014	0.130	.	0.737	0.177	0.659	0.019	0.001	0.706	6.067	0.008	0.636	207.431	1.562	5.993	0.006	0.716	214.570	-0.694	0.011	2	.	.
7658	2	2	1	7	17	1	1	1	1	0.024	0.200	0.136	0.016	0.047	0.247	.	0.028	0.295	0.676	0.002	0.001	0.706	6.067	0.008	0.636	207.431	1.562	5.993	0.006	0.716	214.570	-0.694	0.011	2	.	.
7658	2	2	1	7	18	1	2	1	2	0.132	0.163	0.048	0.005	0.054	0.216	.	0.223	0.253	0.676	0.002	0.001	0.706	6.067	0.008	0.636	207.431	1.562	5.993	0.006	0.716	214.570	-0.694	0.011	2	.	.
7658	2	2	1	7	19	1	1	2	3	.	.	.	.	.	.	.	.	.	.	.	.	.	.	.	.	.	.	.	.	.	.	.	.	.	.	.
7658	2	2	1	7	20	1	2	2	4	.	.	.	.	.	.	.	.	.	.	.	.	.	.	.	.	.	.	.	.	.	.	.	.	.	.	.
7658	2	2	1	8	21	1	1	1	1	0.042	0.142	0.036	0.004	0.070	0.212	.	0.054	0.183	0.719	0.001	0.001	0.706	6.067	0.008	0.636	207.431	1.562	5.993	0.006	0.716	214.570	-0.694	0.011	2	.	.
7658	2	2	1	8	22	1	2	1	2	-0.251	0.173	0.050	0.005	0.084	0.257	.	-0.322	0.222	0.701	0.001	0.001	0.706	6.067	0.008	0.636	207.431	1.562	5.993	0.006	0.716	214.570	-0.694	0.011	2	.	.
7658	2	2	1	8	23	1	1	2	3	0.391	0.159	0.118	0.015	0.008	0.168	.	0.589	0.230	0.719	0.001	0.001	0.706	6.067	0.008	0.636	207.431	1.562	5.993	0.006	0.716	214.570	-0.694	0.011	2	.	.
7658	2	2	1	8	24	1	2	2	4	0.277	0.156	0.037	0.003	0.022	0.178	.	0.416	0.223	0.719	0.001	0.001	0.706	6.067	0.008	0.636	207.431	1.562	5.993	0.006	0.716	214.570	-0.694	0.011	2	.	.
7658	2	2	1	10	25	1	1	1	1	.	.	.	.	.	.	.	.	.	.	.	.	.	.	.	.	.	.	.	.	.	.	.	.	.	.	.
7658	2	2	1	10	26	1	2	1	2	.	.	.	.	.	.	.	.	.	.	.	.	.	.	.	.	.	.	.	.	.	.	.	.	.	.	.
7658	2	2	1	10	27	1	1	2	3	0.461	0.132	0.188	0.008	0.061	0.193	.	0.602	0.172	0.699	0.001	0.001	0.706	6.067	0.008	0.636	207.431	1.562	5.993	0.006	0.716	214.570	-0.694	0.011	2	.	.
7658	2	2	1	10	28	1	2	2	4	0.563	0.111	0.120	0.020	0.000	0.111	.	0.726	0.143	0.699	0.001	0.001	0.706	6.067	0.008	0.636	207.431	1.562	5.993	0.006	0.716	214.570	-0.694	0.011	2	.	.
7658	2	2	1	11	29	1	1	1	1	0.000	0.192	0.134	0.018	0.054	0.243	.	0.001	0.283	0.676	0.001	0.001	0.706	6.067	0.008	0.636	207.431	1.562	5.993	0.006	0.716	214.570	-0.694	0.011	2	.	.
7658	2	2	1	11	30	1	2	1	2	-0.033	0.193	0.062	0.004	0.041	0.252	.	-0.020	0.288	0.676	0.001	0.001	0.706	6.067	0.008	0.636	207.431	1.562	5.993	0.006	0.716	214.570	-0.694	0.011	2	.	.
7658	2	2	1	11	31	1	1	2	3	0.515	0.172	0.096	0.011	0.021	0.172	.	0.780	0.259	0.676	0.001	0.001	0.706	6.067	0.008	0.636	207.431	1.562	5.993	0.006	0.716	214.570	-0.694	0.011	2	.	.
7658	2	2	1	11	32	1	2	2	4	0.520	0.175	0.031	0.005	0.011	0.171	.	0.787	0.272	0.676	0.001	0.001	0.706	6.067	0.008	0.636	207.431	1.562	5.993	0.006	0.716	214.570	-0.694	0.011	2	.	.
7658	2	2	1	2	33	3	3	3	5	0.052	0.228	0.241	0.034	0.007	0.234	.	0.154	0.710	0.380	0.001	0.001	0.603	6.171	0.010	0.683	367.549	1.476	6.022	0.007	0.789	374.088	-1.815	-1.199	2	.	.
7658	2	2	1	3	34	3	3	3	5	0.000	0.202	0.080	0.008	0.017	0.219	0.608	0.001	0.681	0.380	0.001	0.001	0.603	6.171	0.010	0.683	367.549	1.476	6.022	0.007	0.789	374.088	-1.815	-1.199	2	1.337	0.423
7658	2	2	1	4	35	3	3	3	5	0.029	0.189	0.191	0.033	0.004	0.194	.	0.101	0.615	0.380	0.017	0.003	0.603	6.171	0.010	0.683	367.549	1.476	6.022	0.007	0.789	374.088	-1.815	-1.199	2	.	.
7658	2	2	1	6	36	3	3	3	5	-0.024	0.217	0.311	0.062	0.005	0.222	.	-0.116	0.624	0.411	0.011	0.002	0.603	6.171	0.010	0.683	367.549	1.476	6.022	0.007	0.789	374.088	-1.815	-1.199	2	.	.
7658	2	2	1	7	37	3	3	3	5	.	.	.	.	.	.	.	.	.	.	.	.	.	.	.	.	.	.	.	.	.	.	.	.	.	.	.
7658	2	2	1	8	38	3	3	3	5	0.009	0.206	0.061	0.006	0.000	0.206	.	0.044	0.776	0.269	0.001	0.001	0.603	6.171	0.010	0.683	367.549	1.476	6.022	0.007	0.789	374.088	-1.815	-1.199	2	.	.
7658	2	2	1	10	39	3	3	3	5	.	.	.	.	.	.	.	.	.	.	.	.	.	.	.	.	.	.	.	.	.	.	.	.	.	.	.
7658	2	2	1	11	40	3	3	3	5	0.049	0.170	0.125	0.018	0.000	0.170	.	0.203	0.547	0.331	0.001	0.001	0.603	6.171	0.010	0.683	367.549	1.476	6.022	0.007	0.789	374.088	-1.815	-1.199	2	.	.
7658	2	2	2	2	1	1	1	1	1	.	.	.	.	.	.	.	.	.	.	.	.	.	.	.	.	.	.	.	.	.	.	.	.	.	.	.
7658	2	2	2	2	2	1	2	1	2	.	.	.	.	.	.	.	.	.	.	.	.	.	.	.	.	.	.	.	.	.	.	.	.	.	.	.
7658	2	2	2	2	3	1	1	2	3	.	.	.	.	.	.	.	.	.	.	.	.	.	.	.	.	.	.	.	.	.	.	.	.	.	.	.
7658	2	2	2	2	4	1	2	2	4	.	.	.	.	.	.	.	.	.	.	.	.	.	.	.	.	.	.	.	.	.	.	.	.	.	.	.
7658	2	2	2	3	5	1	1	1	1	.	.	.	.	.	.	.	.	.	.	.	.	.	.	.	.	.	.	.	.	.	.	.	.	.	.	.
7658	2	2	2	3	6	1	2	1	2	.	.	.	.	.	.	.	.	.	.	.	.	.	.	.	.	.	.	.	.	.	.	.	.	.	.	.
7658	2	2	2	3	7	1	1	2	3	.	.	.	.	.	.	.	.	.	.	.	.	.	.	.	.	.	.	.	.	.	.	.	.	.	.	.
7658	2	2	2	3	8	1	2	2	4	.	.	.	.	.	.	.	.	.	.	.	.	.	.	.	.	.	.	.	.	.	.	.	.	.	.	.
7658	2	2	2	4	9	1	1	1	1	.	.	.	.	.	.	.	.	.	.	.	.	.	.	.	.	.	.	.	.	.	.	.	.	.	.	.
7658	2	2	2	4	10	1	2	1	2	.	.	.	.	.	.	.	.	.	.	.	.	.	.	.	.	.	.	.	.	.	.	.	.	.	.	.
7658	2	2	2	4	11	1	1	2	3	.	.	.	.	.	.	.	.	.	.	.	.	.	.	.	.	.	.	.	.	.	.	.	.	.	.	.
7658	2	2	2	4	12	1	2	2	4	.	.	.	.	.	.	.	.	.	.	.	.	.	.	.	.	.	.	.	.	.	.	.	.	.	.	.
7658	2	2	2	6	13	1	1	1	1	.	.	.	.	.	.	.	.	.	.	.	.	.	.	.	.	.	.	.	.	.	.	.	.	.	.	.
7658	2	2	2	6	14	1	2	1	2	.	.	.	.	.	.	.	.	.	.	.	.	.	.	.	.	.	.	.	.	.	.	.	.	.	.	.
7658	2	2	2	6	15	1	1	2	3	.	.	.	.	.	.	.	.	.	.	.	.	.	.	.	.	.	.	.	.	.	.	.	.	.	.	.
7658	2	2	2	6	16	1	2	2	4	.	.	.	.	.	.	.	.	.	.	.	.	.	.	.	.	.	.	.	.	.	.	.	.	.	.	.
7658	2	2	2	7	17	1	1	1	1	.	.	.	.	.	.	.	.	.	.	.	.	.	.	.	.	.	.	.	.	.	.	.	.	.	.	.
7658	2	2	2	7	18	1	2	1	2	.	.	.	.	.	.	.	.	.	.	.	.	.	.	.	.	.	.	.	.	.	.	.	.	.	.	.
7658	2	2	2	7	19	1	1	2	3	.	.	.	.	.	.	.	.	.	.	.	.	.	.	.	.	.	.	.	.	.	.	.	.	.	.	.
7658	2	2	2	7	20	1	2	2	4	.	.	.	.	.	.	.	.	.	.	.	.	.	.	.	.	.	.	.	.	.	.	.	.	.	.	.
7658	2	2	2	8	21	1	1	1	1	.	.	.	.	.	.	.	.	.	.	.	.	.	.	.	.	.	.	.	.	.	.	.	.	.	.	.
7658	2	2	2	8	22	1	2	1	2	.	.	.	.	.	.	.	.	.	.	.	.	.	.	.	.	.	.	.	.	.	.	.	.	.	.	.
7658	2	2	2	8	23	1	1	2	3	.	.	.	.	.	.	.	.	.	.	.	.	.	.	.	.	.	.	.	.	.	.	.	.	.	.	.
7658	2	2	2	8	24	1	2	2	4	.	.	.	.	.	.	.	.	.	.	.	.	.	.	.	.	.	.	.	.	.	.	.	.	.	.	.
7658	2	2	2	10	25	1	1	1	1	.	.	.	.	.	.	.	.	.	.	.	.	.	.	.	.	.	.	.	.	.	.	.	.	.	.	.
7658	2	2	2	10	26	1	2	1	2	.	.	.	.	.	.	.	.	.	.	.	.	.	.	.	.	.	.	.	.	.	.	.	.	.	.	.
7658	2	2	2	10	27	1	1	2	3	.	.	.	.	.	.	.	.	.	.	.	.	.	.	.	.	.	.	.	.	.	.	.	.	.	.	.
7658	2	2	2	10	28	1	2	2	4	.	.	.	.	.	.	.	.	.	.	.	.	.	.	.	.	.	.	.	.	.	.	.	.	.	.	.
7658	2	2	2	11	29	1	1	1	1	.	.	.	.	.	.	.	.	.	.	.	.	.	.	.	.	.	.	.	.	.	.	.	.	.	.	.
7658	2	2	2	11	30	1	2	1	2	.	.	.	.	.	.	.	.	.	.	.	.	.	.	.	.	.	.	.	.	.	.	.	.	.	.	.
7658	2	2	2	11	31	1	1	2	3	.	.	.	.	.	.	.	.	.	.	.	.	.	.	.	.	.	.	.	.	.	.	.	.	.	.	.
7658	2	2	2	11	32	1	2	2	4	.	.	.	.	.	.	.	.	.	.	.	.	.	.	.	.	.	.	.	.	.	.	.	.	.	.	.
7658	2	2	2	2	33	3	3	3	5	.	.	.	.	.	.	.	.	.	.	.	.	.	.	.	.	.	.	.	.	.	.	.	.	.	.	.
7658	2	2	2	3	34	3	3	3	5	.	.	.	.	.	.	.	.	.	.	.	.	.	.	.	.	.	.	.	.	.	.	.	.	.	.	.
7658	2	2	2	4	35	3	3	3	5	.	.	.	.	.	.	.	.	.	.	.	.	.	.	.	.	.	.	.	.	.	.	.	.	.	.	.
7658	2	2	2	6	36	3	3	3	5	.	.	.	.	.	.	.	.	.	.	.	.	.	.	.	.	.	.	.	.	.	.	.	.	.	.	.
7658	2	2	2	7	37	3	3	3	5	.	.	.	.	.	.	.	.	.	.	.	.	.	.	.	.	.	.	.	.	.	.	.	.	.	.	.
7658	2	2	2	8	38	3	3	3	5	.	.	.	.	.	.	.	.	.	.	.	.	.	.	.	.	.	.	.	.	.	.	.	.	.	.	.
7658	2	2	2	10	39	3	3	3	5	.	.	.	.	.	.	.	.	.	.	.	.	.	.	.	.	.	.	.	.	.	.	.	.	.	.	.
7658	2	2	2	11	40	3	3	3	5	.	.	.	.	.	.	.	.	.	.	.	.	.	.	.	.	.	.	.	.	.	.	.	.	.	.	.
7658	2	2	3	2	1	1	1	1	1	.	.	.	.	.	.	.	.	.	.	.	.	.	.	.	.	.	.	.	.	.	.	.	.	.	.	.
7658	2	2	3	2	2	1	2	1	2	.	.	.	.	.	.	.	.	.	.	.	.	.	.	.	.	.	.	.	.	.	.	.	.	.	.	.
7658	2	2	3	2	3	1	1	2	3	.	.	.	.	.	.	.	.	.	.	.	.	.	.	.	.	.	.	.	.	.	.	.	.	.	.	.
7658	2	2	3	2	4	1	2	2	4	.	.	.	.	.	.	.	.	.	.	.	.	.	.	.	.	.	.	.	.	.	.	.	.	.	.	.
7658	2	2	3	3	5	1	1	1	1	.	.	.	.	.	.	.	.	.	.	.	.	.	.	.	.	.	.	.	.	.	.	.	.	.	.	.
7658	2	2	3	3	6	1	2	1	2	.	.	.	.	.	.	.	.	.	.	.	.	.	.	.	.	.	.	.	.	.	.	.	.	.	.	.
7658	2	2	3	3	7	1	1	2	3	.	.	.	.	.	.	.	.	.	.	.	.	.	.	.	.	.	.	.	.	.	.	.	.	.	.	.
7658	2	2	3	3	8	1	2	2	4	.	.	.	.	.	.	.	.	.	.	.	.	.	.	.	.	.	.	.	.	.	.	.	.	.	.	.
7658	2	2	3	4	9	1	1	1	1	.	.	.	.	.	.	.	.	.	.	.	.	.	.	.	.	.	.	.	.	.	.	.	.	.	.	.
7658	2	2	3	4	10	1	2	1	2	.	.	.	.	.	.	.	.	.	.	.	.	.	.	.	.	.	.	.	.	.	.	.	.	.	.	.
7658	2	2	3	4	11	1	1	2	3	.	.	.	.	.	.	.	.	.	.	.	.	.	.	.	.	.	.	.	.	.	.	.	.	.	.	.
7658	2	2	3	4	12	1	2	2	4	.	.	.	.	.	.	.	.	.	.	.	.	.	.	.	.	.	.	.	.	.	.	.	.	.	.	.
7658	2	2	3	6	13	1	1	1	1	.	.	.	.	.	.	.	.	.	.	.	.	.	.	.	.	.	.	.	.	.	.	.	.	.	.	.
7658	2	2	3	6	14	1	2	1	2	.	.	.	.	.	.	.	.	.	.	.	.	.	.	.	.	.	.	.	.	.	.	.	.	.	.	.
7658	2	2	3	6	15	1	1	2	3	.	.	.	.	.	.	.	.	.	.	.	.	.	.	.	.	.	.	.	.	.	.	.	.	.	.	.
7658	2	2	3	6	16	1	2	2	4	.	.	.	.	.	.	.	.	.	.	.	.	.	.	.	.	.	.	.	.	.	.	.	.	.	.	.
7658	2	2	3	7	17	1	1	1	1	.	.	.	.	.	.	.	.	.	.	.	.	.	.	.	.	.	.	.	.	.	.	.	.	.	.	.
7658	2	2	3	7	18	1	2	1	2	.	.	.	.	.	.	.	.	.	.	.	.	.	.	.	.	.	.	.	.	.	.	.	.	.	.	.
7658	2	2	3	7	19	1	1	2	3	.	.	.	.	.	.	.	.	.	.	.	.	.	.	.	.	.	.	.	.	.	.	.	.	.	.	.
7658	2	2	3	7	20	1	2	2	4	.	.	.	.	.	.	.	.	.	.	.	.	.	.	.	.	.	.	.	.	.	.	.	.	.	.	.
7658	2	2	3	8	21	1	1	1	1	.	.	.	.	.	.	.	.	.	.	.	.	.	.	.	.	.	.	.	.	.	.	.	.	.	.	.
7658	2	2	3	8	22	1	2	1	2	.	.	.	.	.	.	.	.	.	.	.	.	.	.	.	.	.	.	.	.	.	.	.	.	.	.	.
7658	2	2	3	8	23	1	1	2	3	.	.	.	.	.	.	.	.	.	.	.	.	.	.	.	.	.	.	.	.	.	.	.	.	.	.	.
7658	2	2	3	8	24	1	2	2	4	.	.	.	.	.	.	.	.	.	.	.	.	.	.	.	.	.	.	.	.	.	.	.	.	.	.	.
7658	2	2	3	10	25	1	1	1	1	.	.	.	.	.	.	.	.	.	.	.	.	.	.	.	.	.	.	.	.	.	.	.	.	.	.	.
7658	2	2	3	10	26	1	2	1	2	.	.	.	.	.	.	.	.	.	.	.	.	.	.	.	.	.	.	.	.	.	.	.	.	.	.	.
7658	2	2	3	10	27	1	1	2	3	.	.	.	.	.	.	.	.	.	.	.	.	.	.	.	.	.	.	.	.	.	.	.	.	.	.	.
7658	2	2	3	10	28	1	2	2	4	.	.	.	.	.	.	.	.	.	.	.	.	.	.	.	.	.	.	.	.	.	.	.	.	.	.	.
7658	2	2	3	11	29	1	1	1	1	.	.	.	.	.	.	.	.	.	.	.	.	.	.	.	.	.	.	.	.	.	.	.	.	.	.	.
7658	2	2	3	11	30	1	2	1	2	.	.	.	.	.	.	.	.	.	.	.	.	.	.	.	.	.	.	.	.	.	.	.	.	.	.	.
7658	2	2	3	11	31	1	1	2	3	.	.	.	.	.	.	.	.	.	.	.	.	.	.	.	.	.	.	.	.	.	.	.	.	.	.	.
7658	2	2	3	11	32	1	2	2	4	.	.	.	.	.	.	.	.	.	.	.	.	.	.	.	.	.	.	.	.	.	.	.	.	.	.	.
7658	2	2	3	2	33	3	3	3	5	.	.	.	.	.	.	.	.	.	.	.	.	.	.	.	.	.	.	.	.	.	.	.	.	.	.	.
7658	2	2	3	3	34	3	3	3	5	.	.	.	.	.	.	.	.	.	.	.	.	.	.	.	.	.	.	.	.	.	.	.	.	.	.	.
7658	2	2	3	4	35	3	3	3	5	.	.	.	.	.	.	.	.	.	.	.	.	.	.	.	.	.	.	.	.	.	.	.	.	.	.	.

7658	2	2	3	6	36	3	3	3	5	.	.	.	.	.	.	.	.	.	.	.	.	.	.	.	.	.	.	.	.	.	.	.	.	.	.	.
7658	2	2	3	7	37	3	3	3	5	.	.	.	.	.	.	.	.	.	.	.	.	.	.	.	.	.	.	.	.	.	.	.	.	.	.	.
7658	2	2	3	8	38	3	3	3	5	.	.	.	.	.	.	.	.	.	.	.	.	.	.	.	.	.	.	.	.	.	.	.	.	.	.	.
7658	2	2	3	10	39	3	3	3	5	.	.	.	.	.	.	.	.	.	.	.	.	.	.	.	.	.	.	.	.	.	.	.	.	.	.	.
7658	2	2	3	11	40	3	3	3	5	.	.	.	.	.	.	.	.	.	.	.	.	.	.	.	.	.	.	.	.	.	.	.	.	.	.	.
7658	2	2	4	2	1	1	1	1	1	.	.	.	.	.	.	.	.	.	.	.	.	.	.	.	.	.	.	.	.	.	.	.	.	.	.	.
7658	2	2	4	2	2	1	2	1	2	.	.	.	.	.	.	.	.	.	.	.	.	.	.	.	.	.	.	.	.	.	.	.	.	.	.	.
7658	2	2	4	2	3	1	1	2	3	.	.	.	.	.	.	.	.	.	.	.	.	.	.	.	.	.	.	.	.	.	.	.	.	.	.	.
7658	2	2	4	2	4	1	2	2	4	.	.	.	.	.	.	.	.	.	.	.	.	.	.	.	.	.	.	.	.	.	.	.	.	.	.	.
7658	2	2	4	3	5	1	1	1	1	.	.	.	.	.	.	.	.	.	.	.	.	.	.	.	.	.	.	.	.	.	.	.	.	.	.	.
7658	2	2	4	3	6	1	2	1	2	.	.	.	.	.	.	.	.	.	.	.	.	.	.	.	.	.	.	.	.	.	.	.	.	.	.	.
7658	2	2	4	3	7	1	1	2	3	.	.	.	.	.	.	.	.	.	.	.	.	.	.	.	.	.	.	.	.	.	.	.	.	.	.	.
7658	2	2	4	3	8	1	2	2	4	.	.	.	.	.	.	.	.	.	.	.	.	.	.	.	.	.	.	.	.	.	.	.	.	.	.	.
7658	2	2	4	4	9	1	1	1	1	.	.	.	.	.	.	.	.	.	.	.	.	.	.	.	.	.	.	.	.	.	.	.	.	.	.	.
7658	2	2	4	4	10	1	2	1	2	.	.	.	.	.	.	.	.	.	.	.	.	.	.	.	.	.	.	.	.	.	.	.	.	.	.	.
7658	2	2	4	4	11	1	1	2	3	.	.	.	.	.	.	.	.	.	.	.	.	.	.	.	.	.	.	.	.	.	.	.	.	.	.	.
7658	2	2	4	4	12	1	2	2	4	.	.	.	.	.	.	.	.	.	.	.	.	.	.	.	.	.	.	.	.	.	.	.	.	.	.	.
7658	2	2	4	6	13	1	1	1	1	.	.	.	.	.	.	.	.	.	.	.	.	.	.	.	.	.	.	.	.	.	.	.	.	.	.	.
7658	2	2	4	6	14	1	2	1	2	.	.	.	.	.	.	.	.	.	.	.	.	.	.	.	.	.	.	.	.	.	.	.	.	.	.	.
7658	2	2	4	6	15	1	1	2	3	.	.	.	.	.	.	.	.	.	.	.	.	.	.	.	.	.	.	.	.	.	.	.	.	.	.	.
7658	2	2	4	6	16	1	2	2	4	.	.	.	.	.	.	.	.	.	.	.	.	.	.	.	.	.	.	.	.	.	.	.	.	.	.	.
7658	2	2	4	7	17	1	1	1	1	.	.	.	.	.	.	.	.	.	.	.	.	.	.	.	.	.	.	.	.	.	.	.	.	.	.	.
7658	2	2	4	7	18	1	2	1	2	.	.	.	.	.	.	.	.	.	.	.	.	.	.	.	.	.	.	.	.	.	.	.	.	.	.	.
7658	2	2	4	7	19	1	1	2	3	.	.	.	.	.	.	.	.	.	.	.	.	.	.	.	.	.	.	.	.	.	.	.	.	.	.	.
7658	2	2	4	7	20	1	2	2	4	.	.	.	.	.	.	.	.	.	.	.	.	.	.	.	.	.	.	.	.	.	.	.	.	.	.	.
7658	2	2	4	8	21	1	1	1	1	.	.	.	.	.	.	.	.	.	.	.	.	.	.	.	.	.	.	.	.	.	.	.	.	.	.	.
7658	2	2	4	8	22	1	2	1	2	.	.	.	.	.	.	.	.	.	.	.	.	.	.	.	.	.	.	.	.	.	.	.	.	.	.	.
7658	2	2	4	8	23	1	1	2	3	.	.	.	.	.	.	.	.	.	.	.	.	.	.	.	.	.	.	.	.	.	.	.	.	.	.	.
7658	2	2	4	8	24	1	2	2	4	.	.	.	.	.	.	.	.	.	.	.	.	.	.	.	.	.	.	.	.	.	.	.	.	.	.	.
7658	2	2	4	10	25	1	1	1	1	.	.	.	.	.	.	.	.	.	.	.	.	.	.	.	.	.	.	.	.	.	.	.	.	.	.	.
7658	2	2	4	10	26	1	2	1	2	.	.	.	.	.	.	.	.	.	.	.	.	.	.	.	.	.	.	.	.	.	.	.	.	.	.	.
7658	2	2	4	10	27	1	1	2	3	.	.	.	.	.	.	.	.	.	.	.	.	.	.	.	.	.	.	.	.	.	.	.	.	.	.	.
7658	2	2	4	10	28	1	2	2	4	.	.	.	.	.	.	.	.	.	.	.	.	.	.	.	.	.	.	.	.	.	.	.	.	.	.	.
7658	2	2	4	11	29	1	1	1	1	.	.	.	.	.	.	.	.	.	.	.	.	.	.	.	.	.	.	.	.	.	.	.	.	.	.	.
7658	2	2	4	11	30	1	2	1	2	.	.	.	.	.	.	.	.	.	.	.	.	.	.	.	.	.	.	.	.	.	.	.	.	.	.	.
7658	2	2	4	11	31	1	1	2	3	.	.	.	.	.	.	.	.	.	.	.	.	.	.	.	.	.	.	.	.	.	.	.	.	.	.	.
7658	2	2	4	11	32	1	2	2	4	.	.	.	.	.	.	.	.	.	.	.	.	.	.	.	.	.	.	.	.	.	.	.	.	.	.	.
7658	2	2	4	2	33	3	3	3	5	.	.	.	.	.	.	.	.	.	.	.	.	.	.	.	.	.	.	.	.	.	.	.	.	.	.	.
7658	2	2	4	3	34	3	3	3	5	.	.	.	.	.	.	.	.	.	.	.	.	.	.	.	.	.	.	.	.	.	.	.	.	.	.	.
7658	2	2	4	4	35	3	3	3	5	.	.	.	.	.	.	.	.	.	.	.	.	.	.	.	.	.	.	.	.	.	.	.	.	.	.	.
7658	2	2	4	6	36	3	3	3	5	.	.	.	.	.	.	.	.	.	.	.	.	.	.	.	.	.	.	.	.	.	.	.	.	.	.	.
7658	2	2	4	7	37	3	3	3	5	.	.	.	.	.	.	.	.	.	.	.	.	.	.	.	.	.	.	.	.	.	.	.	.	.	.	.
7658	2	2	4	8	38	3	3	3	5	.	.	.	.	.	.	.	.	.	.	.	.	.	.	.	.	.	.	.	.	.	.	.	.	.	.	.
7658	2	2	4	10	39	3	3	3	5	.	.	.	.	.	.	.	.	.	.	.	.	.	.	.	.	.	.	.	.	.	.	.	.	.	.	.
7658	2	2	4	11	40	3	3	3	5	.	.	.	.	.	.	.	.	.	.	.	.	.	.	.	.	.	.	.	.	.	.	.	.	.	.	.
8501	1	1	1	2	1	1	1	1	1	.	.	.	.	.	.	.	.	.	.	.	.	.	.	.	.	.	.	.	.	.	.	.	.	.	.	.
8501	1	1	1	2	2	1	2	1	2	.	.	.	.	.	.	.	.	.	.	.	.	.	.	.	.	.	.	.	.	.	.	.	.	.	.	.
8501	1	1	1	2	3	1	1	2	3	.	.	.	.	.	.	.	.	.	.	.	.	.	.	.	.	.	.	.	.	.	.	.	.	.	.	.
8501	1	1	1	2	4	1	2	2	4	.	.	.	.	.	.	.	.	.	.	.	.	.	.	.	.	.	.	.	.	.	.	.	.	.	.	.
8501	1	1	1	3	5	1	1	1	1	.	.	.	.	.	.	.	.	.	.	.	.	.	.	.	.	.	.	.	.	.	.	.	.	.	.	.
8501	1	1	1	3	6	1	2	1	2	.	.	.	.	.	.	.	.	.	.	.	.	.	.	.	.	.	.	.	.	.	.	.	.	.	.	.
8501	1	1	1	3	7	1	1	2	3	.	.	.	.	.	.	.	.	.	.	.	.	.	.	.	.	.	.	.	.	.	.	.	.	.	.	.
8501	1	1	1	3	8	1	2	2	4	.	.	.	.	.	.	.	.	.	.	.	.	.	.	.	.	.	.	.	.	.	.	.	.	.	.	.
8501	1	1	1	4	9	1	1	1	1	.	.	.	.	.	.	.	.	.	.	.	.	.	.	.	.	.	.	.	.	.	.	.	.	.	.	.
8501	1	1	1	4	10	1	2	1	2	.	.	.	.	.	.	.	.	.	.	.	.	.	.	.	.	.	.	.	.	.	.	.	.	.	.	.
8501	1	1	1	4	11	1	1	2	3	.	.	.	.	.	.	.	.	.	.	.	.	.	.	.	.	.	.	.	.	.	.	.	.	.	.	.
8501	1	1	1	4	12	1	2	2	4	.	.	.	.	.	.	.	.	.	.	.	.	.	.	.	.	.	.	.	.	.	.	.	.	.	.	.
8501	1	1	1	6	13	1	1	1	1	.	.	.	.	.	.	.	.	.	.	.	.	.	.	.	.	.	.	.	.	.	.	.	.	.	.	.
8501	1	1	1	6	14	1	2	1	2	.	.	.	.	.	.	.	.	.	.	.	.	.	.	.	.	.	.	.	.	.	.	.	.	.	.	.
8501	1	1	1	6	15	1	1	2	3	.	.	.	.	.	.	.	.	.	.	.	.	.	.	.	.	.	.	.	.	.	.	.	.	.	.	.
8501	1	1	1	6	16	1	2	2	4	.	.	.	.	.	.	.	.	.	.	.	.	.	.	.	.	.	.	.	.	.	.	.	.	.	.	.
8501	1	1	1	7	17	1	1	1	1	.	.	.	.	.	.	.	.	.	.	.	.	.	.	.	.	.	.	.	.	.	.	.	.	.	.	.
8501	1	1	1	7	18	1	2	1	2	.	.	.	.	.	.	.	.	.	.	.	.	.	.	.	.	.	.	.	.	.	.	.	.	.	.	.
8501	1	1	1	7	19	1	1	2	3	.	.	.	.	.	.	.	.	.	.	.	.	.	.	.	.	.	.	.	.	.	.	.	.	.	.	.
8501	1	1	1	7	20	1	2	2	4	.	.	.	.	.	.	.	.	.	.	.	.	.	.	.	.	.	.	.	.	.	.	.	.	.	.	.
8501	1	1	1	8	21	1	1	1	1	.	.	.	.	.	.	.	.	.	.	.	.	.	.	.	.	.	.	.	.	.	.	.	.	.	.	.
8501	1	1	1	8	22	1	2	1	2	.	.	.	.	.	.	.	.	.	.	.	.	.	.	.	.	.	.	.	.	.	.	.	.	.	.	.
8501	1	1	1	8	23	1	1	2	3	.	.	.	.	.	.	.	.	.	.	.	.	.	.	.	.	.	.	.	.	.	.	.	.	.	.	.
8501	1	1	1	8	24	1	2	2	4	.	.	.	.	.	.	.	.	.	.	.	.	.	.	.	.	.	.	.	.	.	.	.	.	.	.	.
8501	1	1	1	10	25	1	1	1	1	.	.	.	.	.	.	.	.	.	.	.	.	.	.	.	.	.	.	.	.	.	.	.	.	.	.	.
8501	1	1	1	10	26	1	2	1	2	.	.	.	.	.	.	.	.	.	.	.	.	.	.	.	.	.	.	.	.	.	.	.	.	.	.	.
8501	1	1	1	10	27	1	1	2	3	.	.	.	.	.	.	.	.	.	.	.	.	.	.	.	.	.	.	.	.	.	.	.	.	.	.	.
8501	1	1	1	10	28	1	2	2	4	.	.	.	.	.	.	.	.	.	.	.	.	.	.	.	.	.	.	.	.	.	.	.	.	.	.	.
8501	1	1	1	11	29	1	1	1	1	.	.	.	.	.	.	.	.	.	.	.	.	.	.	.	.	.	.	.	.	.	.	.	.	.	.	.
8501	1	1	1	11	30	1	2	1	2	.	.	.	.	.	.	.	.	.	.	.	.	.	.	.	.	.	.	.	.	.	.	.	.	.	.	.
8501	1	1	1	11	31	1	1	2	3	.	.	.	.	.	.	.	.	.	.	.	.	.	.	.	.	.	.	.	.	.	.	.	.	.	.	.
8501	1	1	1	11	32	1	2	2	4	.	.	.	.	.	.	.	.	.	.	.	.	.	.	.	.	.	.	.	.	.	.	.	.	.	.	.
8501	1	1	1	2	33	3	3	3	5	.	.	.	.	.	.	.	.	.	.	.	.	.	.	.	.	.	.	.	.	.	.	.	.	.	.	.
8501	1	1	1	3	34	3	3	3	5	.	.	.	.	.	.	.	.	.	.	.	.	.	.	.	.	.	.	.	.	.	.	.	.	.	.	.
8501	1	1	1	4	35	3	3	3	5	.	.	.	.	.	.	.	.	.	.	.	.	.	.	.	.	.	.	.	.	.	.	.	.	.	.	.
8501	1	1	1	6	36	3	3	3	5	.	.	.	.	.	.	.	.	.	.	.	.	.	.	.	.	.	.	.	.	.	.	.	.	.	.	.
8501	1	1	1	7	37	3	3	3	5	.	.	.	.	.	.	.	.	.	.	.	.	.	.	.	.	.	.	.	.	.	.	.	.	.	.	.
8501	1	1	1	8	38	3	3	3	5	.	.	.	.	.	.	.	.	.	.	.	.	.	.	.	.	.	.	.	.	.	.	.	.	.	.	.
8501	1	1	1	10	39	3	3	3	5	.	.	.	.	.	.	.	.	.	.	.	.	.	.	.	.	.	.	.	.	.	.	.	.	.	.	.
8501	1	1	1	11	40	3	3	3	5	.	.	.	.	.	.	.	.	.	.	.	.	.	.	.	.	.	.	.	.	.	.	.	.	.	.	.
8501	1	1	2	2	1	1	1	1	1	0.004	0.122	0.093	0.009	0.048	0.171	.	-0.001	0.192	0.636	0.001	0.001	0.675	25.810	0.906	3.408	1752.525	1.196	25.787	0.908	3.347	1778.228	-0.809	-0.070	2	.	.
8501	1	1	2	2	2	1	2	1	2	.	.	.	.	.	.	.	.	.	.	.	.	.	.	.	.	.	.	.	.	.	.	.	.	.	.	.
8501	1	1	2	2	3	1	1	2	3	0.312	0.140	0.190	0.021	0.000	0.140	.	0.488	0.219	0.639	0.001	0.001	0.675	25.810	0.906	3.408	1752.525	1.196	25.787	0.908	3.347	1778.228	-0.809	-0.070	2	.	.
8501	1	1	2	2	4	1	2	2	4	.	.	.	.	.	.	.	.	.	.	.	.	.	.	.	.	.	.	.	.	.	.	.	.	.	.	.
8501	1	1	2	3	5	1	1	1	1	0.028	0.156	0.168	0.012	0.054	0.210	.	0.036	0.233	0.674	0.038	0.005	0.675	25.810	0.906	3.408	1752.525	1.196	25.787	0.908	3.347	1778.228	-0.809	-0.070	2	.	.
8501	1	1	2	3	6	1	2	1	2	-0.057	0.132	0.101	0.006	0.041	0.173	.	-0.084	0.198	0.674	0.038	0.005	0.675	25.810	0.906	3.408	1752.525	1.196	25.787	0.908	3.347	1778.228	-0.809	-0.070	2	.	.
8501	1	1	2	3	7	1	1	2	3	0.402	0.140	0.151	0.012	0.016	0.159	.	0.566	0.214	0.674	0.038	0.005	0.675	25.810	0.906	3.408	1752.525	1.196	25.787	0.908	3.347	1778.228	-0.809	-0.070	2	.	.
8501	1	1	2	3	8	1	2	2	4	0.380	0.140	0.085	0.008	0.038	0.175	.	0.468	0.203	0.674	0.038	0.005	0.675	25.810	0.906	3.408	1752.525	1.196	25.787	0.908	3.347	1778.228	-0.809	-0.070	2	.	.
8501	1	1	2	4	9	1	1	1	1	0.028	0.143	0.226	0.050	0.085	0.226	.	0.168	0.211	0.678	0.001	0.001	0.675	25.810	0.906	3.408	1752.525	1.196	25.787	0.908	3.347	1778.228	-0.809	-0.070	2	.	.
8501	1	1	2	4	10	1	2	1	2	0.234	0.139	0.252	0.034	0.034	0.173	.	0.440	0.216	0.647	0.001	0.001	0.675	25.810	0.906	3.408	1752.525	1.196	25.787	0.908	3.347	1778.228	-0.809	-0.070	2	.	.
8501	1	1	2	4	11	1	1	2	3	0.565	0.123	0.224	0.034	0.040	0.170	.	0.903	0.192	0.679	0.001	0.001	0.675	25.810	0.906	3.408	1752.525	1.196	25.787	0.908	3.347	1778.228	-0.809	-0.070	2	.	.
8501	1	1	2	4	12	1	2	2	4	0.639	0.113	0.170	0.021	0.000	0.113	.	0.657	0.167	0.678	0.001	0.001	0.675	25.810	0.906	3.408	1752.525	1.196	25.787	0.908	3.347	1778.228	-0.809	-0.070	2	.	.
8501	1	1	2	6	13	1	1	1	1	0.063	0.179	0.356	0.167	0.036	0.215	.	0.098	0.269	0.674	0.023	0.003	0.675	25.810	0.906	3.408	1752.525	1.196	25.787	0.908	3.347	1778.228	-0.809	-0.070	2	.	.
8501	1	1	2	6	14	1	2	1	2	0.071	0.137	0.297	0.047	0.068	0.204	.	0.103	0.206	0.674	0.023	0.002	0.675	25.810	0.906	3.408	1752.525	1.196	25.787	0.908	3.347	1778.228	-0.809	-0.070	2	.	.
8501	1	1	2	6	15	1	1	2	3	0.356	0.147	0.344	0.069	0.038	0.185	.	0.533	0.220	0.674	0.023	0.002	0.675	25.810	0.906	3.408	1752.525	1.196	25.787	0.908	3.347	1778.228	-0.809	-0.070	2	.	.
8501	1	1	2	6	16	1	2	2	4	0.345	0.147	0.258	0.060	0.015	0.162	.	0.500	0.219	0.674	0.023	0.002	0.675	25.810	0.906	3.408	1752.525	1.196	25.787	0.908	3.347	1778.228	-0.809	-0.070	2	.	.
8501	1	1	2	7	17	1	1	1	1	0.035	0.134	0.117	0.012	0.079	0.201	.	0.052	0.199	0.674	0.001	0.001	0.675	25.810	0.906	3.408	1752.525	1.196	25.787	0.908	3.347	1778.228	-0.809	-0.070	2	.	.
8501	1	1	2	7	18	1	2	1	2	-0.072	0.135	0.074	0.009	0.071	0.206	.	-0.107	0.206	0.661	0.001	0.001	0.675	25.810	0.906	3.408	1752.525	1.196	25.787	0.908	3.347	1778.228	-0.809	-0.070	2	.	.
8501	1	1	2	7	19	1	1	2	3	.	.	.	.	.	.	.	.	.	.	.	.	.	.	.	.	.	.	.	.	.	.	.	.	.	.	.
8501	1	1	2	7	20	1	2	2	4	.	.	.	.	.	.	.	.	.	.	.	.	.	.	.	.	.	.	.	.	.	.	.	.	.	.	.
8501	1	1	2	8	21	1	1	1	1	0.093	0.158	0.161	0.037	0.043	0.201	.	0.155	0.234	0.674	0.031	0.004	0.675	25.810	0.906	3.408	1752.525	1.196	25.787	0.908	3.347	1778.228	-0.809	-0.070	2	.	.
8501	1	1	2	8	22	1	2	1	2	-0.090	0.142	0.099	0.007	0.027	0.169	.	-0.136	0.211	0.674	0.031	0.004	0.675	25.810	0.906	3.408	1752.525	1.196	25.787	0.908	3.347	1778.228	-0.809	-0.070	2	.	.
8501	1	1	2	8	23	1	1	2	3	0.441	0.159	0.163	0.014	0.008	0.172	.	0.657	0.237	0.674	0.031	0.004	0.675	25.810	0.906	3.408	1752.525	1.196	25.787	0.908	3.347	1778.228	-0.809	-0.070	2	.	.
8501	1	1	2	8	24	1	2	2	4	0.326	0.161	0.116	0.010	0.025	0.186	.	0.488	0.238	0.674	0.031	0.004	0.675	25.810	0.906	3.408	1752.525	1.196	25.787	0.908	3.347	1778.228	-0.809	-0.070	2	.	.
8501	1	1	2	10	25	1	1	1	1	.	.	.	.	.	.	.	.	.	.	.	.	.	.	.	.	.	.	.	.	.	.	.	.	.	.	.
8501	1	1	2	10	26	1	2	1	2	.	.	.	.	.	.	.	.	.	.	.	.	.	.	.	.	.	.	.	.	.	.	.	.	.	.	.
8501	1	1	2	10	27	1	1	2	3	0.289	0.154	0.150	0.011	0.055	0.209	.	0.434	0.232	0.674	0.002	0.001	0.675	25.810	0.906	3.408	1752.525	1.196	25.787	0.908	3.347	1778.228	-0.809	-0.070	2	.	.
8501	1	1	2	10	28	1	2	2	4	0.195	0.160	0.083	0.006	0.062	0.221	.	0.282	0.237	0.674	0.002	0.001	0.675	25.810	0.906	3.408	1752.525	1.196	25.787	0.908	3.347	1778.228	-0.809	-0.070	2	.	.
8501	1	1	2	11	29	1	1	1	1	0.000	0.152	0.162	0.011	0.048	0.200	.	0.001	0.226	0.674	0.004	0.001	0.675	25.810	0.906	3.408	1752.525	1.196	25.787	0.908	3.347	1778.228	-0.809	-0.070	2	.	.
8501	1	1	2	11	30	1	2	1	2	0.000	0.149	0.105	0.008	0.050	0.199	.	0.060	0.223	0.674	0.004	0.001	0.675	25.810	0.906	3.408	1752.525	1.196	25.787	0.908	3.347	1778.228	-0.809	-0.070	2	.	.
8501	1	1	2	11	31	1	1	2	3	0.334	0.170	0.169	0.014	0.033	0.202	.	0.497	0.252	0.674	0.004	0.001	0.675	25.810	0.906	3.408	1752.525	1.196	25.787	0.908	3.347	1778.228	-0.809	-0.070	2	.	.
8501	1	1	2	11	32	1	2	2	4	0.509	0.130	0.099	0.008	0.006	0.136	.	0.752	0.194	0.674	0.004	0.001	0.675	25.810	0.906	3.408	1752.525	1.196	25.787	0.908	3.347	1778.228	-0.809	-0.070	2	.	.
8501	1	1	2	2	33	3	3	3	5	-0.003	0.161	0.077	0.008	0.000	0.161	.	-0.027	0.710	0.288	0.001	0.001	0.635	25.841	0.897	2.666	2131.912	1.076	25.789	0.895	2.625	2155.093	-1.484	-0.983	2	.	.
8501	1	1	2	3	34	3	3	3	5	0.000	0.155	0.199	0.022	0.000	0.155	1.174	0.001	0.728	0.288	0.001	0.001	0.635	25.841	0.897	2.666	2131.912	1.076	25.789	0.895	2.625	2155.093	-1.484	-0.983	2	1.030	0.435
8501	1	1	2	4	35	3	3	3	5	0.050	0.147	0.266	0.042	0.000	0.147	.	0.228	0.664	0.288	0.007	0.002	0.635	25.841	0.897	2.666	2131.912	1.076	25.789	0.895	2.625	2155.093	-1.484	-0.983	2	.	.
8501	1	1	2	6	36	3	3	3	5	0.039	0.157	0.280	0.050	0.000	0.157	.	0.113	0.769	0.288	0.007	0.002	0.635	25.841	0.897	2.666	2131.912	1.076	25.789	0.895	2.625	2155.093	-1.484	-0.983	2	.	.
8501	1	1	2	7	37	3	3	3	5	.	.	.	.	.	.	.	.	.	.	.	.	.	.	.	.	.	.	.	.	.	.	.	.	.	.	.
8501	1	1	2	8	38	3	3	3	5	-0.013	0.161	0.167	0.023	0.000	0.161	.	-0.087	0.798	0.288	0.035	0.005	0.635	25.841	0.897	2.666	2131.912	1.076	25.789	0.895	2.625	2155.093	-1.484	-0.983	2	.	.
8501	1	1	2	10	39	3	3	3	5	.	.	.	.	.	.	.	.	.	.	.	.	.	.	.	.	.	.	.	.	.	.	.	.	.	.	.
8501	1	1	2	11	40	3	3	3	5	-0.009	0.135	0.127	0.010	0.000	0.135	.	-0.064	0.636	0.288	0.029	0.004	0.635	25.841	0.897	2.666	2131.912	1.076	25.789	0.895	2.625	2155.093	-1.484	-0.983	2	.	.
8501	1	1	3	2	1	1	1	1	1	-0.109	0.117	0.139	0.005	0.067	0.183	.	-0.159	0.164	0.710	0.002	0.001	0.593	29.182	.	11.056	393.963	1.043	44.583	.	11.412	425.885	-0.757	-0.164	2	.	.
8501	1	1	3	2	2	1	2	1	2	-0.028	0.105	0.128	0.006	0.089	0.194	.	-0.041	0.154	0.680	0.002	0.001	0.593	29.182	.	11.056	393.963	1.043	44.583	.	11.412	425.885	-0.757	-0.164	2	.	.
8501	1	1	3	2	3	1	1	2	3	0.108	0.181	0.180	0.024	0.051	0.231	.	0.158	0.252	0.710	0.002	0.001	0.593	29.182	.	11.056	393.963	1.043	44.583	.	11.412	425.885	-0.757	-0.164	2	.	.
8501	1	1	3	2	4	1	2	2	4	.	.	.	.	.	.	.	.	.	.	.	.	.	.	.	.	.	.	.	.	.	.	.	.	.	.	.
8501	1	1	3	3	5	1	1	1	1	-0.125	0.149	0.170	0.025	0.041	0.190	.	-0.177	0.216	0.690	0.022	0.003	0.593	29.182	.	11.056	393.963	1.043	44.583	.	11.412	425.885	-0.757	-0.164	2	.	.
8501	1	1	3	3	6	1	2	1	2	-0.111	0.152	0.135	0.033	0.104	0.256	.	-0.160	0.220	0.690	0.022	0.003	0.593	29.182	.	11.056	393.963	1.043	44.583	.	11.412	425.885	-0.757	-0.164	2	.	.
8501	1	1	3	3	7	1	1	2	3	0.348	0.158	0.209	0.034	0.059	0.217	.	0.505	0.229	0.690	0.022	0.003	0.593	29.182	.	11.056	393.963	1.043	44.583	.	11.412	425.885	-0.757	-0.164	2	.	.
8501	1	1	3	3	8	1	2	2	4	0.508	0.156	0.172	0.025	0.012	0.168	.	0.734	0.224	0.690	0.022	0.003	0.593	29.182	.	11.056	393.963	1.043	44.583	.	11.412	425.885	-0.757	-0.164	2	.	.
8501	1	1	3	4	9	1	1	1	1	-0.168	0.146	0.287	0.039	0.074	0.220	.	-0.248	0.212	0.690	0.044	0.006	0.593	29.182	.	11.056	393.963	1.043	44.583	.	11.412	425.885	-0.757	-0.164	2	.	.
8501	1	1	3	4	10	1	2	1	2	-0.045	0.169	0.189	0.032	0.052	0.221	.	-0.063	0.246	0.690	0.044	0.006	0.593	29.182	.	11.056	393.963	1.043	44.583	.	11.412	425.885	-0.757	-0.164	2	.	.
8501	1	1	3	4	11	1	1	2	3	0.369	0.169	0.260	0.046	0.026	0.195	.	0.541	0.243	0.690	0.044	0.006	0.593	29.182	.	11.056	393.963	1.043	44.583	.	11.412	425.885	-0.757	-0.164	2	.	.
8501	1	1	3	4	12	1	2	2	4	0.435	0.178	0.181	0.034	0.022	0.200	.	0.625	0.257	0.690	0.044	0.006	0.593	29.182	.	11.056	393.963	1.043	44.583	.	11.412	425.885	-0.757	-0.164	2	.	.
8501	1	1	3	6	13	1	1	1	1	0.000	0.147	0.242	0.042	0.104	0.251	.	0.000	0.215	0.690	0.034	0.005	0.593	29.182	.	11.056	393.963	1.043	44.583	.	11.412	425.885	-0.757	-0.164	2	.	.
8501	1	1	3	6	14	1	2	1	2	0.023	0.150	0.174	0.037	0.067	0.217	.	0.041	0.219	0.690	0.034	0.005	0.593	29.182	.	11.056	393.963	1.043	44.583	.	11.412	425.885	-0.757	-0.164	2	.	.

8501	1	1	3	6	15	1	1	2	3	0.419	0.189	0.275	0.069	0.056	0.245	.	0.610	0.275	0.690	0.034	0.005	0.593	29.182	.	11.056	393.963	1.043	44.583	.	11.412	425.885	-0.757	-0.164	2	.	.
8501	1	1	3	6	16	1	2	2	4	0.667	0.148	0.214	0.047	0.010	0.158	.	0.968	0.216	0.690	0.034	0.005	0.593	29.182	.	11.056	393.963	1.043	44.583	.	11.412	425.885	-0.757	-0.164	2	.	.
8501	1	1	3	7	17	1	1	1	1	0.011	0.154	0.204	0.030	0.064	0.219	.	0.011	0.227	0.689	0.002	0.001	0.593	29.182	.	11.056	393.963	1.043	44.583	.	11.412	425.885	-0.757	-0.164	2	.	.
8501	1	1	3	7	18	1	2	1	2	0.103	0.145	0.149	0.021	0.099	0.243	.	0.157	0.213	0.689	0.002	0.001	0.593	29.182	.	11.056	393.963	1.043	44.583	.	11.412	425.885	-0.757	-0.164	2	.	.
8501	1	1	3	7	19	1	1	2	3	.	.	.	.	.	.	.	.	.	.	.	.	.	.	.	.	.	.	.	.	.	.	.	.	.	.	.
8501	1	1	3	7	20	1	2	2	4	.	.	.	.	.	.	.	.	.	.	.	.	.	.	.	.	.	.	.	.	.	.	.	.	.	.	.
8501	1	1	3	8	21	1	1	1	1	0.012	0.158	0.187	0.020	0.096	0.254	.	0.016	0.229	0.690	0.001	0.001	0.593	29.182	.	11.056	393.963	1.043	44.583	.	11.412	425.885	-0.757	-0.164	2	.	.
8501	1	1	3	8	22	1	2	1	2	-0.007	0.153	0.136	0.015	0.106	0.258	.	-0.016	0.222	0.690	0.001	0.001	0.593	29.182	.	11.056	393.963	1.043	44.583	.	11.412	425.885	-0.757	-0.164	2	.	.
8501	1	1	3	8	23	1	1	2	3	0.537	0.143	0.134	0.011	0.025	0.169	.	0.774	0.210	0.690	0.001	0.001	0.593	29.182	.	11.056	393.963	1.043	44.583	.	11.412	425.885	-0.757	-0.164	2	.	.
8501	1	1	3	8	24	1	2	2	4	0.423	0.134	0.086	0.013	0.007	0.140	.	0.634	0.194	0.690	0.001	0.001	0.593	29.182	.	11.056	393.963	1.043	44.583	.	11.412	425.885	-0.757	-0.164	2	.	.
8501	1	1	3	10	25	1	1	1	1	.	.	.	.	.	.	.	.	.	.	.	.	.	.	.	.	.	.	.	.	.	.	.	.	.	.	.
8501	1	1	3	10	26	1	2	1	2	.	.	.	.	.	.	.	.	.	.	.	.	.	.	.	.	.	.	.	.	.	.	.	.	.	.	.
8501	1	1	3	10	27	1	1	2	3	0.424	0.164	0.147	0.016	0.045	0.208	.	0.623	0.236	0.690	0.001	0.001	0.593	29.182	.	11.056	393.963	1.043	44.583	.	11.412	425.885	-0.757	-0.164	2	.	.
8501	1	1	3	10	28	1	2	2	4	0.446	0.158	0.080	0.007	0.094	0.252	.	0.643	0.228	0.690	0.001	0.001	0.593	29.182	.	11.056	393.963	1.043	44.583	.	11.412	425.885	-0.757	-0.164	2	.	.
8501	1	1	3	11	29	1	1	1	1	0.000	0.144	0.161	0.016	0.096	0.240	.	0.001	0.210	0.690	0.007	0.001	0.593	29.182	.	11.056	393.963	1.043	44.583	.	11.412	425.885	-0.757	-0.164	2	.	.
8501	1	1	3	11	30	1	2	1	2	-0.004	0.129	0.106	0.014	0.073	0.202	.	0.095	0.187	0.690	0.007	0.001	0.593	29.182	.	11.056	393.963	1.043	44.583	.	11.412	425.885	-0.757	-0.164	2	.	.
8501	1	1	3	11	31	1	1	2	3	0.549	0.134	0.165	0.019	0.021	0.155	.	0.795	0.194	0.690	0.007	0.001	0.593	29.182	.	11.056	393.963	1.043	44.583	.	11.412	425.885	-0.757	-0.164	2	.	.
8501	1	1	3	11	32	1	2	2	4	0.387	0.144	0.122	0.012	0.028	0.172	.	0.574	0.209	0.690	0.007	0.001	0.593	29.182	.	11.056	393.963	1.043	44.583	.	11.412	425.885	-0.757	-0.164	2	.	.
8501	1	1	3	2	33	3	3	3	5	-0.046	0.271	0.218	0.020	0.000	0.271	.	-0.170	1.006	0.269	0.002	0.001	0.406	23.143	.	6.343	245.829	0.686	36.026	.	10.696	126.075	-0.460	-0.054	2	.	.
8501	1	1	3	3	34	3	3	3	5	0.000	0.194	0.286	0.046	0.000	0.194	0.754	0.001	0.724	0.269	0.019	0.004	0.406	23.143	.	6.343	245.829	0.686	36.026	.	10.696	126.075	-0.460	-0.054	2	0.733	0.569
8501	1	1	3	4	35	3	3	3	5	0.009	0.169	0.301	0.047	0.000	0.169	.	0.041	0.633	0.269	0.009	0.002	0.406	23.143	.	6.343	245.829	0.686	36.026	.	10.696	126.075	-0.460	-0.054	2	.	.
8501	1	1	3	6	36	3	3	3	5	-0.042	0.193	0.324	0.052	0.000	0.193	.	-0.155	0.723	0.269	0.035	0.006	0.406	23.143	.	6.343	245.829	0.686	36.026	.	10.696	126.075	-0.460	-0.054	2	.	.
8501	1	1	3	7	37	3	3	3	5	.	.	.	.	.	.	.	.	.	.	.	.	.	.	.	.	.	.	.	.	.	.	.	.	.	.	.
8501	1	1	3	8	38	3	3	3	5	-0.016	0.207	0.250	0.039	0.000	0.207	.	-0.037	0.779	0.269	0.002	0.001	0.406	23.143	.	6.343	245.829	0.686	36.026	.	10.696	126.075	-0.460	-0.054	2	.	.
8501	1	1	3	10	39	3	3	3	5	.	.	.	.	.	.	.	.	.	.	.	.	.	.	.	.	.	.	.	.	.	.	.	.	.	.	.
8501	1	1	3	11	40	3	3	3	5	0.004	0.218	0.156	0.026	0.000	0.218	.	0.011	0.812	0.269	0.008	0.001	0.406	23.143	.	6.343	245.829	0.686	36.026	.	10.696	126.075	-0.460	-0.054	2	.	.
8501	1	1	4	2	1	1	1	1	1	.	.	.	.	.	.	.	.	.	.	.	.	.	.	.	.	.	.	.	.	.	.	.	.	.	.	.
8501	1	1	4	2	2	1	2	1	2	.	.	.	.	.	.	.	.	.	.	.	.	.	.	.	.	.	.	.	.	.	.	.	.	.	.	.
8501	1	1	4	2	3	1	1	2	3	.	.	.	.	.	.	.	.	.	.	.	.	.	.	.	.	.	.	.	.	.	.	.	.	.	.	.
8501	1	1	4	2	4	1	2	2	4	.	.	.	.	.	.	.	.	.	.	.	.	.	.	.	.	.	.	.	.	.	.	.	.	.	.	.
8501	1	1	4	3	5	1	1	1	1	.	.	.	.	.	.	.	.	.	.	.	.	.	.	.	.	.	.	.	.	.	.	.	.	.	.	.
8501	1	1	4	3	6	1	2	1	2	.	.	.	.	.	.	.	.	.	.	.	.	.	.	.	.	.	.	.	.	.	.	.	.	.	.	.
8501	1	1	4	3	7	1	1	2	3	.	.	.	.	.	.	.	.	.	.	.	.	.	.	.	.	.	.	.	.	.	.	.	.	.	.	.
8501	1	1	4	3	8	1	2	2	4	.	.	.	.	.	.	.	.	.	.	.	.	.	.	.	.	.	.	.	.	.	.	.	.	.	.	.
8501	1	1	4	4	9	1	1	1	1	.	.	.	.	.	.	.	.	.	.	.	.	.	.	.	.	.	.	.	.	.	.	.	.	.	.	.
8501	1	1	4	4	10	1	2	1	2	.	.	.	.	.	.	.	.	.	.	.	.	.	.	.	.	.	.	.	.	.	.	.	.	.	.	.
8501	1	1	4	4	11	1	1	2	3	.	.	.	.	.	.	.	.	.	.	.	.	.	.	.	.	.	.	.	.	.	.	.	.	.	.	.
8501	1	1	4	4	12	1	2	2	4	.	.	.	.	.	.	.	.	.	.	.	.	.	.	.	.	.	.	.	.	.	.	.	.	.	.	.
8501	1	1	4	6	13	1	1	1	1	.	.	.	.	.	.	.	.	.	.	.	.	.	.	.	.	.	.	.	.	.	.	.	.	.	.	.
8501	1	1	4	6	14	1	2	1	2	.	.	.	.	.	.	.	.	.	.	.	.	.	.	.	.	.	.	.	.	.	.	.	.	.	.	.
8501	1	1	4	6	15	1	1	2	3	.	.	.	.	.	.	.	.	.	.	.	.	.	.	.	.	.	.	.	.	.	.	.	.	.	.	.
8501	1	1	4	6	16	1	2	2	4	.	.	.	.	.	.	.	.	.	.	.	.	.	.	.	.	.	.	.	.	.	.	.	.	.	.	.
8501	1	1	4	7	17	1	1	1	1	.	.	.	.	.	.	.	.	.	.	.	.	.	.	.	.	.	.	.	.	.	.	.	.	.	.	.
8501	1	1	4	7	18	1	2	1	2	.	.	.	.	.	.	.	.	.	.	.	.	.	.	.	.	.	.	.	.	.	.	.	.	.	.	.
8501	1	1	4	7	19	1	1	2	3	.	.	.	.	.	.	.	.	.	.	.	.	.	.	.	.	.	.	.	.	.	.	.	.	.	.	.
8501	1	1	4	7	20	1	2	2	4	.	.	.	.	.	.	.	.	.	.	.	.	.	.	.	.	.	.	.	.	.	.	.	.	.	.	.
8501	1	1	4	8	21	1	1	1	1	.	.	.	.	.	.	.	.	.	.	.	.	.	.	.	.	.	.	.	.	.	.	.	.	.	.	.
8501	1	1	4	8	22	1	2	1	2	.	.	.	.	.	.	.	.	.	.	.	.	.	.	.	.	.	.	.	.	.	.	.	.	.	.	.
8501	1	1	4	8	23	1	1	2	3	.	.	.	.	.	.	.	.	.	.	.	.	.	.	.	.	.	.	.	.	.	.	.	.	.	.	.
8501	1	1	4	8	24	1	2	2	4	.	.	.	.	.	.	.	.	.	.	.	.	.	.	.	.	.	.	.	.	.	.	.	.	.	.	.
8501	1	1	4	10	25	1	1	1	1	.	.	.	.	.	.	.	.	.	.	.	.	.	.	.	.	.	.	.	.	.	.	.	.	.	.	.
8501	1	1	4	10	26	1	2	1	2	.	.	.	.	.	.	.	.	.	.	.	.	.	.	.	.	.	.	.	.	.	.	.	.	.	.	.
8501	1	1	4	10	27	1	1	2	3	.	.	.	.	.	.	.	.	.	.	.	.	.	.	.	.	.	.	.	.	.	.	.	.	.	.	.
8501	1	1	4	10	28	1	2	2	4	.	.	.	.	.	.	.	.	.	.	.	.	.	.	.	.	.	.	.	.	.	.	.	.	.	.	.
8501	1	1	4	11	29	1	1	1	1	.	.	.	.	.	.	.	.	.	.	.	.	.	.	.	.	.	.	.	.	.	.	.	.	.	.	.
8501	1	1	4	11	30	1	2	1	2	.	.	.	.	.	.	.	.	.	.	.	.	.	.	.	.	.	.	.	.	.	.	.	.	.	.	.
8501	1	1	4	11	31	1	1	2	3	.	.	.	.	.	.	.	.	.	.	.	.	.	.	.	.	.	.	.	.	.	.	.	.	.	.	.
8501	1	1	4	11	32	1	2	2	4	.	.	.	.	.	.	.	.	.	.	.	.	.	.	.	.	.	.	.	.	.	.	.	.	.	.	.
8501	1	1	4	2	33	3	3	3	5	.	.	.	.	.	.	.	.	.	.	.	.	.	.	.	.	.	.	.	.	.	.	.	.	.	.	.
8501	1	1	4	3	34	3	3	3	5	.	.	.	.	.	.	.	.	.	.	.	.	.	.	.	.	.	.	.	.	.	.	.	.	.	.	.
8501	1	1	4	4	35	3	3	3	5	.	.	.	.	.	.	.	.	.	.	.	.	.	.	.	.	.	.	.	.	.	.	.	.	.	.	.
8501	1	1	4	6	36	3	3	3	5	.	.	.	.	.	.	.	.	.	.	.	.	.	.	.	.	.	.	.	.	.	.	.	.	.	.	.
8501	1	1	4	7	37	3	3	3	5	.	.	.	.	.	.	.	.	.	.	.	.	.	.	.	.	.	.	.	.	.	.	.	.	.	.	.
8501	1	1	4	8	38	3	3	3	5	.	.	.	.	.	.	.	.	.	.	.	.	.	.	.	.	.	.	.	.	.	.	.	.	.	.	.
8501	1	1	4	10	39	3	3	3	5	.	.	.	.	.	.	.	.	.	.	.	.	.	.	.	.	.	.	.	.	.	.	.	.	.	.	.
8501	1	1	4	11	40	3	3	3	5	.	.	.	.	.	.	.	.	.	.	.	.	.	.	.	.	.	.	.	.	.	.	.	.	.	.	.
8514	1	2	1	2	1	1	1	1	1	.	.	.	.	.	.	.	.	.	.	.	.	.	.	.	.	.	.	.	.	.	.	.	.	.	.	.
8514	1	2	1	2	2	1	2	1	2	.	.	.	.	.	.	.	.	.	.	.	.	.	.	.	.	.	.	.	.	.	.	.	.	.	.	.
8514	1	2	1	2	3	1	1	2	3	.	.	.	.	.	.	.	.	.	.	.	.	.	.	.	.	.	.	.	.	.	.	.	.	.	.	.
8514	1	2	1	2	4	1	2	2	4	.	.	.	.	.	.	.	.	.	.	.	.	.	.	.	.	.	.	.	.	.	.	.	.	.	.	.
8514	1	2	1	3	5	1	1	1	1	.	.	.	.	.	.	.	.	.	.	.	.	.	.	.	.	.	.	.	.	.	.	.	.	.	.	.
8514	1	2	1	3	6	1	2	1	2	.	.	.	.	.	.	.	.	.	.	.	.	.	.	.	.	.	.	.	.	.	.	.	.	.	.	.
8514	1	2	1	3	7	1	1	2	3	.	.	.	.	.	.	.	.	.	.	.	.	.	.	.	.	.	.	.	.	.	.	.	.	.	.	.
8514	1	2	1	3	8	1	2	2	4	.	.	.	.	.	.	.	.	.	.	.	.	.	.	.	.	.	.	.	.	.	.	.	.	.	.	.
8514	1	2	1	4	9	1	1	1	1	.	.	.	.	.	.	.	.	.	.	.	.	.	.	.	.	.	.	.	.	.	.	.	.	.	.	.
8514	1	2	1	4	10	1	2	1	2	.	.	.	.	.	.	.	.	.	.	.	.	.	.	.	.	.	.	.	.	.	.	.	.	.	.	.
8514	1	2	1	4	11	1	1	2	3	.	.	.	.	.	.	.	.	.	.	.	.	.	.	.	.	.	.	.	.	.	.	.	.	.	.	.
8514	1	2	1	4	12	1	2	2	4	.	.	.	.	.	.	.	.	.	.	.	.	.	.	.	.	.	.	.	.	.	.	.	.	.	.	.
8514	1	2	1	6	13	1	1	1	1	.	.	.	.	.	.	.	.	.	.	.	.	.	.	.	.	.	.	.	.	.	.	.	.	.	.	.
8514	1	2	1	6	14	1	2	1	2	.	.	.	.	.	.	.	.	.	.	.	.	.	.	.	.	.	.	.	.	.	.	.	.	.	.	.
8514	1	2	1	6	15	1	1	2	3	.	.	.	.	.	.	.	.	.	.	.	.	.	.	.	.	.	.	.	.	.	.	.	.	.	.	.
8514	1	2	1	6	16	1	2	2	4	.	.	.	.	.	.	.	.	.	.	.	.	.	.	.	.	.	.	.	.	.	.	.	.	.	.	.
8514	1	2	1	7	17	1	1	1	1	.	.	.	.	.	.	.	.	.	.	.	.	.	.	.	.	.	.	.	.	.	.	.	.	.	.	.
8514	1	2	1	7	18	1	2	1	2	.	.	.	.	.	.	.	.	.	.	.	.	.	.	.	.	.	.	.	.	.	.	.	.	.	.	.
8514	1	2	1	7	19	1	1	2	3	.	.	.	.	.	.	.	.	.	.	.	.	.	.	.	.	.	.	.	.	.	.	.	.	.	.	.
8514	1	2	1	7	20	1	2	2	4	.	.	.	.	.	.	.	.	.	.	.	.	.	.	.	.	.	.	.	.	.	.	.	.	.	.	.
8514	1	2	1	8	21	1	1	1	1	.	.	.	.	.	.	.	.	.	.	.	.	.	.	.	.	.	.	.	.	.	.	.	.	.	.	.
8514	1	2	1	8	22	1	2	1	2	.	.	.	.	.	.	.	.	.	.	.	.	.	.	.	.	.	.	.	.	.	.	.	.	.	.	.
8514	1	2	1	8	23	1	1	2	3	.	.	.	.	.	.	.	.	.	.	.	.	.	.	.	.	.	.	.	.	.	.	.	.	.	.	.
8514	1	2	1	8	24	1	2	2	4	.	.	.	.	.	.	.	.	.	.	.	.	.	.	.	.	.	.	.	.	.	.	.	.	.	.	.
8514	1	2	1	10	25	1	1	1	1	.	.	.	.	.	.	.	.	.	.	.	.	.	.	.	.	.	.	.	.	.	.	.	.	.	.	.
8514	1	2	1	10	26	1	2	1	2	.	.	.	.	.	.	.	.	.	.	.	.	.	.	.	.	.	.	.	.	.	.	.	.	.	.	.
8514	1	2	1	10	27	1	1	2	3	.	.	.	.	.	.	.	.	.	.	.	.	.	.	.	.	.	.	.	.	.	.	.	.	.	.	.
8514	1	2	1	10	28	1	2	2	4	.	.	.	.	.	.	.	.	.	.	.	.	.	.	.	.	.	.	.	.	.	.	.	.	.	.	.
8514	1	2	1	11	29	1	1	1	1	.	.	.	.	.	.	.	.	.	.	.	.	.	.	.	.	.	.	.	.	.	.	.	.	.	.	.
8514	1	2	1	11	30	1	2	1	2	.	.	.	.	.	.	.	.	.	.	.	.	.	.	.	.	.	.	.	.	.	.	.	.	.	.	.
8514	1	2	1	11	31	1	1	2	3	.	.	.	.	.	.	.	.	.	.	.	.	.	.	.	.	.	.	.	.	.	.	.	.	.	.	.
8514	1	2	1	11	32	1	2	2	4	.	.	.	.	.	.	.	.	.	.	.	.	.	.	.	.	.	.	.	.	.	.	.	.	.	.	.
8514	1	2	1	2	33	3	3	3	5	.	.	.	.	.	.	.	.	.	.	.	.	.	.	.	.	.	.	.	.	.	.	.	.	.	.	.
8514	1	2	1	3	34	3	3	3	5	.	.	.	.	.	.	.	.	.	.	.	.	.	.	.	.	.	.	.	.	.	.	.	.	.	.	.
8514	1	2	1	4	35	3	3	3	5	.	.	.	.	.	.	.	.	.	.	.	.	.	.	.	.	.	.	.	.	.	.	.	.	.	.	.
8514	1	2	1	6	36	3	3	3	5	.	.	.	.	.	.	.	.	.	.	.	.	.	.	.	.	.	.	.	.	.	.	.	.	.	.	.
8514	1	2	1	7	37	3	3	3	5	.	.	.	.	.	.	.	.	.	.	.	.	.	.	.	.	.	.	.	.	.	.	.	.	.	.	.
8514	1	2	1	8	38	3	3	3	5	.	.	.	.	.	.	.	.	.	.	.	.	.	.	.	.	.	.	.	.	.	.	.	.	.	.	.
8514	1	2	1	10	39	3	3	3	5	.	.	.	.	.	.	.	.	.	.	.	.	.	.	.	.	.	.	.	.	.	.	.	.	.	.	.
8514	1	2	1	11	40	3	3	3	5	.	.	.	.	.	.	.	.	.	.	.	.	.	.	.	.	.	.	.	.	.	.	.	.	.	.	.
8514	1	2	2	2	1	1	1	1	1	-0.861	0.145	0.119	0.015	0.026	0.170	.	-1.358	0.213	0.696	0.001	0.001	0.610	26.018	0.168	7.200	6277.220	1.166	25.923	0.168	7.117	6300.921	-0.618	-0.009	2	.	.
8514	1	2	2	2	2	1	2	1	2	-0.117	0.101	0.044	0.003	0.028	0.129	.	-0.183	0.158	0.640	0.001	0.001	0.610	26.018	0.168	7.200	6277.220	1.166	25.923	0.168	7.117	6300.921	-0.618	-0.009	2	.	.
8514	1	2	2	2	3	1	1	2	3	0.139	0.112	0.207	0.018	0.039	0.151	.	0.184	0.161	0.696	0.001	0.001	0.610	26.018	0.168	7.200	6277.220	1.166	25.923	0.168	7.117	6300.921	-0.618	-0.009	2	.	.
8514	1	2	2	2	4	1	2	2	4	0.318	0.161	0.025	0.003	0.000	0.161	.	0.497	0.252	0.640	0.001	0.001	0.610	26.018	0.168	7.200	6277.220	1.166	25.923	0.168	7.117	6300.921	-0.618	-0.009	2	.	.
8514	1	2	2	3	5	1	1	1	1	-0.022	0.134	0.076	0.003	0.031	0.164	.	-0.043	0.190	0.696	0.001	0.001	0.610	26.018	0.168	7.200	6277.220	1.166	25.923	0.168	7.117	6300.921	-0.618	-0.009	2	.	.
8514	1	2	2	3	6	1	2	1	2	0.000	0.083	0.051	0.003	0.025	0.108	.	0.001	0.130	0.640	0.001	0.001	0.610	26.018	0.168	7.200	6277.220	1.166	25.923	0.168	7.117	6300.921	-0.618	-0.009	2	.	.
8514	1	2	2	3	7	1	1	2	3	0.203	0.159	0.053	0.003	0.008	0.167	.	0.285	0.231	0.696	0.001	0.001	0.610	26.018	0.168	7.200	6277.220	1.166	25.923	0.168	7.117	6300.921	-0.618	-0.009	2	.	.
8514	1	2	2	3	8	1	2	2	4	0.334	0.122	0.028	0.003	0.000	0.122	.	0.522	0.191	0.640	0.001	0.001	0.610	26.018	0.168	7.200	6277.220	1.166	25.923	0.168	7.117	6300.921	-0.618	-0.009	2	.	.
8514	1	2	2	4	9	1	1	1	1	0.091	0.100	0.290	0.026	0.069	0.169	.	0.143	0.164	0.635	0.006	0.003	0.610	26.018	0.168	7.200	6277.220	1.166	25.923	0.168	7.117	6300.921	-0.618	-0.009	2	.	.
8514	1	2	2	4	10	1	2	1	2	-0.073	0.112	0.244	0.028	0.020	0.131	.	-0.119	0.195	0.576	0.006	0.003	0.610	26.018	0.168	7.200	6277.220	1.166	25.923	0.168	7.117	6300.921	-0.618	-0.009	2	.	.
8514	1	2	2	4	11	1	1	2	3	0.325	0.126	0.236	0.026	0.028	0.154	.	0.518	0.207	0.635	0.006	0.003	0.610	26.018	0.168	7.200	6277.220	1.166	25.923	0.168	7.117	6300.921	-0.618	-0.009	2	.	.
8514	1	2	2	4	12	1	2	2	4	0.292	0.114	0.165	0.014	0.010	0.124	.	0.503	0.196	0.576	0.006	0.003	0.610	26.018	0.168	7.200	6277.220	1.166	25.923	0.168	7.117	6300.921	-0.618	-0.009	2	.	.
8514	1	2	2	6	13	1	1	1	1	-0.048	0.118	0.383	0.024	0.047	0.165	.	-0.084	0.198	0.607	0.007	0.003	0.610	26.018	0.168	7.200	6277.220	1.166	25.923	0.168	7.117	6300.921	-0.618	-0.009	2	.	.
8514	1	2	2	6	14	1	2	1	2	-0.057	0.110	0.258	0.018	0.041	0.150	.	-0.073	0.182	0.607	0.007	0.003	0.610	26.018	0.168	7.200	6277.220	1.166	25.923	0.168	7.117	6300.921	-0.618	-0.009	2	.	.
8514	1	2	2	6	15	1	1	2	3	0.238	0.093	0.380	0.021	0.033	0.126	.	0.388	0.153	0.607	0.007	0.003	0.610	26.018	0.168	7.200	6277.220	1.166	25.923	0.168	7.117	6300.921	-0.618	-0.009	2	.	.
8514	1	2	2	6	16	1	2	2	4	0.365	0.107	0.327	0.020	0.000	0.107	.	0.591	0.181	0.607	0.007	0.003	0.610	26.018	0.168	7.200	6277.220	1.166	25.923	0.168	7.117	6300.921	-0.618	-0.009	2	.	.
8514	1	2	2	7	17	1	1	1	1	.	.	.	.	.	.	.	.	.	.	.	.	.	.	.	.	.	.	.	.	.	.	.	.	.	.	.
8514	1	2	2	7	18	1	2	1	2	.	.	.	.	.	.	.	.	.	.	.	.	.	.	.	.	.	.	.	.	.	.	.	.	.	.	.
8514	1	2	2	7	19	1	1	2	3	.	.	.	.	.	.	.	.	.	.	.	.	.	.	.	.	.	.	.	.	.	.	.	.	.	.	.
8514	1	2	2	7	20	1	2	2	4	.	.	.	.	.	.	.	.	.	.	.	.	.	.	.	.	.	.	.	.	.	.	.	.	.	.	.
8514	1	2	2	8	21	1	1	1	1	-0.043	0.129	0.402	0.032	0.046	0.175	.	-0.074	0.217	0.607	0.009	0.002	0.610	26.018	0.168	7.200	6277.220	1.166	25.923	0.168	7.117	6300.921	-0.618	-0.009	2	.	.
8514	1	2	2	8	22	1	2	1	2	-0.042	0.115	0.278	0.017	0.033	0.149	.	0.008	0.193	0.607	0.009	0.002	0.610	26.018	0.168	7.200	6277.220	1.166	25.923	0.168	7.117	6300.921	-0.618	-0.009	2	.	.
8514	1	2	2	8	23	1	1	2	3	0.242	0.111	0.377	0.016	0.027	0.138	.	0.391	0.185	0.607	0.009	0.002	0.610	26.018	0.168	7.200	6277.220	1.166	25.923	0.168	7.117	6300.921	-0.618	-0.009	2	.	.
8514	1	2	2	8	24	1	2	2	4	0.370	0.126	0.303	0.052	0.000	0.126	.	0.603	0.212	0.607	0.009	0.002	0.610	26.018	0.168	7.200	6277.220	1.166	25.923	0.168	7.117	6300.921	-0.618	-0.009	2	.	.
8514	1	2	2	10	25	1	1	1	1	.	.	.	.	.	.	.	.	.	.	.	.	.	.	.	.	.	.	.	.	.	.	.	.	.	.	.
8514	1	2	2	10	26	1	2	1	2	.	.	.	.	.	.	.	.	.	.	.	.	.	.	.	.	.	.	.	.	.	.	.	.	.	.	.
8514	1	2	2	10	27	1	1	2	3	.	.	.	.	.	.	.	.	.	.	.	.	.	.	.	.	.	.	.	.	.	.	.	.	.	.	.
8514	1	2	2	10	28	1	2	2	4	.	.	.	.	.	.	.	.	.	.	.	.	.	.	.	.	.	.	.	.	.	.	.	.	.	.	.
8514	1	2	2	11	29	1	1	1	1	0.000	0.123	0.329	0.045	0.046	0.169	.	0.001	0.204	0.607	0.001	0.001	0.610	26.018	0.168	7.200	6277.220	1.166	25.923	0.168	7.117	6300.921	-0.618	-0.009	2	.	.
8514	1	2	2	11	30	1	2	1	2	-0.084	0.121	0.167	0.020	0.034	0.154	.	-0.114	0.202	0.607	0.001	0.001	0.610	26.018	0.168	7.200	6277.220	1.166	25.923	0.168	7.117	6300.921	-0.618	-0.009	2	.	.
8514	1	2	2	11	31	1	1	2	3	0.242	0.120	0.261	0.029	0.032	0.152	.	0.418	0.205	0.607	0.001	0.001	0.610	26.018	0.168	7.200	6277.220	1.166	25.923	0.168	7.117	6300.921	-0.618	-0.009	2	.	.
8514	1	2	2	11	32	1	2	2	4	0.273	0.128	0.166	0.019	0.011	0.139	.	0.436	0.210	0.607	0.001	0.001	0.610	26.018	0.168	7.200	6277.220	1.166	25.923	0.168	7.117	6300.921	-0.618	-0.009	2	.	.
8514	1	2	2	2	33	3	3	3	5	-0.015	0.229	0.226	0.026	0.000	0.229	.	-0.067	1.088	0.210	0.001	0.001	0.486	25.985	0.168	3.765	6857.936	0.851	25.988	0.168	3.709	6875.628	-0.264	0.226	1	.	.

8514	1	2	2	3	34	3	3	3	5	0.000	0.213	0.272	0.025	0.000	0.213	1.051	0.001	1.020	0.210	0.001	0.001	0.486	25.985	0.168	3.765	6857.936	0.851	25.988	0.168	3.709	6875.628	-0.264	0.226	1	0.408	0.334
8514	1	2	2	4	35	3	3	3	5	0.011	0.161	0.474	0.055	0.000	0.161	.	0.047	0.779	0.210	0.006	0.003	0.486	25.985	0.168	3.765	6857.936	0.851	25.988	0.168	3.709	6875.628	-0.264	0.226	1	.	.
8514	1	2	2	6	36	3	3	3	5	0.036	0.181	0.353	0.055	0.000	0.181	.	0.144	0.850	0.210	0.007	0.003	0.486	25.985	0.168	3.765	6857.936	0.851	25.988	0.168	3.709	6875.628	-0.264	0.226	1	.	.
8514	1	2	2	7	37	3	3	3	5	.	.	.	.	.	.	.	.	.	.	.	.	.	.	.	.	.	.	.	.	.	.	.	.	.	.	.
8514	1	2	2	8	38	3	3	3	5	-0.064	0.177	0.421	0.040	0.000	0.177	.	-0.298	0.846	0.210	0.009	0.002	0.486	25.985	0.168	3.765	6857.936	0.851	25.988	0.168	3.709	6875.628	-0.264	0.226	1	.	.
8514	1	2	2	10	39	3	3	3	5	.	.	.	.	.	.	.	.	.	.	.	.	.	.	.	.	.	.	.	.	.	.	.	.	.	.	.
8514	1	2	2	11	40	3	3	3	5	0.006	0.205	0.238	0.049	0.000	0.205	.	0.054	0.969	0.210	0.001	0.001	0.486	25.985	0.168	3.765	6857.936	0.851	25.988	0.168	3.709	6875.628	-0.264	0.226	1	.	.
8514	1	2	3	2	1	1	1	1	1	-0.128	0.137	0.279	0.034	0.024	0.161	.	-0.212	0.209	0.675	0.004	0.001	0.565	8.312	.	0.962	18.553	1.117	6.677	.	0.961	5.694	-0.498	0.066	1	.	.
8514	1	2	3	2	2	1	2	1	2	0.047	0.125	0.117	0.020	0.022	0.147	.	0.046	0.192	0.654	0.004	0.001	0.565	8.312	.	0.962	18.553	1.117	6.677	.	1.161	5.694	-0.498	0.066	1	.	.
8514	1	2	3	2	3	1	1	2	3	0.212	0.152	0.178	0.012	0.013	0.165	.	0.297	0.224	0.675	0.004	0.001	0.565	8.312	.	0.962	18.553	1.117	6.677	.	1.361	5.694	-0.498	0.066	1	.	.
8514	1	2	3	2	4	1	2	2	4	0.323	0.184	0.038	0.003	0.000	0.184	.	0.466	0.285	0.654	0.004	0.001	0.565	8.312	.	0.962	18.553	1.117	6.677	.	1.561	5.694	-0.498	0.066	1	.	.
8514	1	2	3	3	5	1	1	1	1	0.039	0.128	0.249	0.029	0.024	0.152	.	0.034	0.205	0.635	0.006	0.003	0.565	8.312	.	0.962	18.553	1.117	6.677	.	1.961	5.694	-0.498	0.066	1	.	.
8514	1	2	3	3	6	1	2	1	2	0.044	0.130	0.159	0.016	0.031	0.160	.	0.081	0.204	0.635	0.006	0.003	0.565	8.312	.	0.962	18.553	1.117	6.677	.	1.761	5.694	-0.498	0.066	1	.	.
8514	1	2	3	3	7	1	1	2	3	0.312	0.150	0.240	0.025	0.023	0.173	.	0.479	0.234	0.642	0.005	0.003	0.565	8.312	.	0.962	18.553	1.117	6.677	.	2.161	5.694	-0.498	0.066	1	.	.
8514	1	2	3	3	8	1	2	2	4	0.433	0.147	0.120	0.024	0.006	0.153	.	0.662	0.236	0.635	0.006	0.003	0.565	8.312	.	0.962	18.553	1.117	6.677	.	2.361	5.694	-0.498	0.066	1	.	.
8514	1	2	3	4	9	1	1	1	1	-0.004	0.152	0.355	0.049	0.027	0.179	.	-0.016	0.237	0.635	0.003	0.001	0.565	8.312	.	0.962	18.553	1.117	6.677	.	2.561	5.694	-0.498	0.066	1	.	.
8514	1	2	3	4	10	1	2	1	2	0.032	0.138	0.250	0.032	0.023	0.161	.	0.045	0.216	0.635	0.003	0.001	0.565	8.312	.	0.962	18.553	1.117	6.677	.	2.561	5.694	-0.498	0.066	1	.	.
8514	1	2	3	4	11	1	1	2	3	0.257	0.149	0.395	0.051	0.022	0.171	.	0.392	0.231	0.635	0.003	0.001	0.565	8.312	.	0.962	18.553	1.117	6.677	.	2.561	5.694	-0.498	0.066	1	.	.
8514	1	2	3	4	12	1	2	2	4	0.428	0.149	0.220	0.021	0.000	0.149	.	0.658	0.235	0.635	0.003	0.001	0.565	8.312	.	0.962	18.553	1.117	6.677	.	2.561	5.694	-0.498	0.066	1	.	.
8514	1	2	3	6	13	1	1	1	1	0.057	0.126	0.331	0.043	0.021	0.147	.	0.072	0.204	0.635	0.015	0.003	0.565	8.312	.	0.962	18.553	1.117	6.677	.	2.561	5.694	-0.498	0.066	1	.	.
8514	1	2	3	6	14	1	2	1	2	0.016	0.127	0.256	0.031	0.024	0.151	.	0.028	0.198	0.635	0.015	0.003	0.565	8.312	.	0.962	18.553	1.117	6.677	.	2.561	5.694	-0.498	0.066	1	.	.
8514	1	2	3	6	15	1	1	2	3	0.328	0.130	0.416	0.055	0.021	0.151	.	0.501	0.208	0.635	0.015	0.003	0.565	8.312	.	0.962	18.553	1.117	6.677	.	2.561	5.694	-0.498	0.066	1	.	.
8514	1	2	3	6	16	1	2	2	4	0.375	0.136	0.257	0.038	0.006	0.141	.	0.594	0.215	0.635	0.015	0.003	0.565	8.312	.	0.962	18.553	1.117	6.677	.	2.561	5.694	-0.498	0.066	1	.	.
8514	1	2	3	7	17	1	1	1	1	0.004	0.200	0.175	0.021	0.024	0.225	.	0.023	0.313	0.635	0.013	0.003	0.565	8.312	.	0.962	18.553	1.117	6.677	.	2.561	5.694	-0.498	0.066	1	.	.
8514	1	2	3	7	18	1	2	1	2	0.061	0.166	0.098	0.008	0.025	0.191	.	0.062	0.265	0.635	0.013	0.003	0.565	8.312	.	0.962	18.553	1.117	6.677	.	2.561	5.694	-0.498	0.066	1	.	.
8514	1	2	3	7	19	1	1	2	3	.	.	.	.	.	.	.	.	.	.	.	.	.	.	.	.	.	.	.	.	.	.	.	.	.	.	.
8514	1	2	3	7	20	1	2	2	4	.	.	.	.	.	.	.	.	.	.	.	.	.	.	.	.	.	.	.	.	.	.	.	.	.	.	.
8514	1	2	3	8	21	1	1	1	1	0.042	0.131	0.184	0.023	0.024	0.156	.	0.052	0.212	0.635	0.020	0.003	0.565	8.312	.	0.962	18.553	1.117	6.677	.	2.561	5.694	-0.498	0.066	1	.	.
8514	1	2	3	8	22	1	2	1	2	0.035	0.129	0.160	0.020	0.023	0.152	.	0.110	0.205	0.635	0.020	0.003	0.565	8.312	.	0.962	18.553	1.117	6.677	.	2.561	5.694	-0.498	0.066	1	.	.
8514	1	2	3	8	23	1	1	2	3	0.292	0.115	0.237	0.027	0.022	0.136	.	0.447	0.183	0.635	0.020	0.003	0.565	8.312	.	0.962	18.553	1.117	6.677	.	2.561	5.694	-0.498	0.066	1	.	.
8514	1	2	3	8	24	1	2	2	4	0.436	0.126	0.190	0.018	0.000	0.126	.	0.674	0.200	0.635	0.020	0.003	0.565	8.312	.	0.962	18.553	1.117	6.677	.	2.561	5.694	-0.498	0.066	1	.	.
8514	1	2	3	10	25	1	1	1	1	.	.	.	.	.	.	.	.	.	.	.	.	.	.	.	.	.	.	.	.	.	.	.	.	.	.	.
8514	1	2	3	10	26	1	2	1	2	.	.	.	.	.	.	.	.	.	.	.	.	.	.	.	.	.	.	.	.	.	.	.	.	.	.	.
8514	1	2	3	10	27	1	1	2	3	0.220	0.177	0.226	0.044	0.045	0.222	.	0.326	0.285	0.635	0.002	0.001	0.565	8.312	.	0.962	18.553	1.117	6.677	.	2.561	5.694	-0.498	0.066	1	.	.
8514	1	2	3	10	28	1	2	2	4	0.454	0.117	0.160	0.011	0.007	0.124	.	0.701	0.187	0.635	0.002	0.001	0.565	8.312	.	0.962	18.553	1.117	6.677	.	2.561	5.694	-0.498	0.066	1	.	.
8514	1	2	3	11	29	1	1	1	1	0.000	0.145	0.259	0.027	0.021	0.166	.	0.001	0.228	0.635	0.003	0.001	0.565	8.312	.	0.962	18.553	1.117	6.677	.	2.561	5.694	-0.498	0.066	1	.	.
8514	1	2	3	11	30	1	2	1	2	0.097	0.149	0.189	0.020	0.022	0.171	.	0.134	0.240	0.635	0.003	0.001	0.565	8.312	.	0.962	18.553	1.117	6.677	.	2.561	5.694	-0.498	0.066	1	.	.
8514	1	2	3	11	31	1	1	2	3	0.398	0.150	0.180	0.016	0.007	0.157	.	0.636	0.235	0.635	0.003	0.002	0.565	8.312	.	0.962	18.553	1.117	6.677	.	2.561	5.694	-0.498	0.066	1	.	.
8514	1	2	3	11	32	1	2	2	4	0.364	0.146	0.158	0.018	0.013	0.159	.	0.559	0.235	0.635	0.003	0.001	0.565	8.312	.	0.962	18.553	1.117	6.677	.	2.561	5.694	-0.498	0.066	1	.	.
8514	1	2	3	2	33	3	3	3	5	-0.051	0.199	0.202	0.035	0.000	0.199	.	-0.234	0.899	0.225	0.006	0.001	0.429	11.691	.	0.921	15.836	0.743	10.362	.	2.806	7.911	-0.836	-0.414	2	.	.
8514	1	2	3	3	34	3	3	3	5	0.000	0.176	0.252	0.029	0.000	0.176	1.153	0.001	0.777	0.225	0.005	0.002	0.429	11.691	.	0.921	15.836	0.743	10.362	.	2.806	7.911	-0.836	-0.414	2	0.988	0.419
8514	1	2	3	4	35	3	3	3	5	-0.001	0.182	0.368	0.070	0.000	0.182	.	0.054	0.807	0.225	0.004	0.001	0.429	11.691	.	0.921	15.836	0.743	10.362	.	2.806	7.911	-0.836	-0.414	2	.	.
8514	1	2	3	6	36	3	3	3	5	0.021	0.172	0.399	0.070	0.000	0.172	.	0.116	0.789	0.225	0.014	0.004	0.429	11.691	.	0.921	15.836	0.743	10.362	.	2.806	7.911	-0.836	-0.414	2	.	.
8514	1	2	3	7	37	3	3	3	5	.	.	.	.	.	.	.	.	.	.	.	.	.	.	.	.	.	.	.	.	.	.	.	.	.	.	.
8514	1	2	3	8	38	3	3	3	5	0.041	0.179	0.289	0.047	0.000	0.179	.	0.149	0.787	0.225	0.020	0.003	0.429	11.691	.	0.921	15.836	0.743	10.362	.	2.806	7.911	-0.836	-0.414	2	.	.
8514	1	2	3	10	39	3	3	3	5	.	.	.	.	.	.	.	.	.	.	.	.	.	.	.	.	.	.	.	.	.	.	.	.	.	.	.
8514	1	2	3	11	40	3	3	3	5	0.051	0.173	0.188	0.020	0.000	0.173	.	0.210	0.777	0.225	0.008	0.002	0.429	11.691	.	0.921	15.836	0.743	10.362	.	2.806	7.911	-0.836	-0.414	2	.	.
8514	1	2	4	2	1	1	1	1	1	-0.060	0.118	0.270	0.024	0.020	0.138	.	-0.092	0.196	0.610	0.005	0.002	0.578	7.876	0.010	2.170	19.612	1.029	6.445	0.010	1.602	18.550	-0.872	-0.294	2	.	.
8514	1	2	4	2	2	1	2	1	2	-0.001	0.135	0.174	0.022	0.015	0.150	.	-0.011	0.227	0.610	0.005	0.002	0.578	7.876	0.010	2.170	19.612	1.029	6.445	0.010	1.602	18.550	-0.872	-0.294	2	.	.
8514	1	2	4	2	3	1	1	2	3	0.232	0.134	0.125	0.008	0.009	0.143	.	0.373	0.216	0.610	0.005	0.002	0.578	7.876	0.010	2.170	19.612	1.029	6.445	0.010	1.602	18.550	-0.872	-0.294	2	.	.
8514	1	2	4	2	4	1	2	2	4	0.288	0.161	0.066	0.008	0.004	0.165	.	0.483	0.267	0.610	0.005	0.002	0.578	7.876	0.010	2.170	19.612	1.029	6.445	0.010	1.602	18.550	-0.872	-0.294	2	.	.
8514	1	2	4	3	5	1	1	1	1	0.042	0.153	0.482	0.079	0.015	0.169	.	0.051	0.259	0.610	0.011	0.002	0.578	7.876	0.010	2.170	19.612	1.029	6.445	0.010	1.602	18.550	-0.872	-0.294	2	.	.
8514	1	2	4	3	6	1	2	1	2	0.037	0.155	0.278	0.040	0.024	0.178	.	0.056	0.258	0.610	0.011	0.002	0.578	7.876	0.010	2.170	19.612	1.029	6.445	0.010	1.602	18.550	-0.872	-0.294	2	.	.
8514	1	2	4	3	7	1	1	2	3	0.344	0.148	0.592	0.091	0.018	0.166	.	0.562	0.245	0.610	0.011	0.002	0.578	7.876	0.010	2.170	19.612	1.029	6.445	0.010	1.602	18.550	-0.872	-0.294	2	.	.
8514	1	2	4	3	8	1	2	2	4	0.414	0.159	0.305	0.046	0.004	0.163	.	0.691	0.262	0.610	0.011	0.002	0.578	7.876	0.010	2.170	19.612	1.029	6.445	0.010	1.602	18.550	-0.872	-0.294	2	.	.
8514	1	2	4	4	9	1	1	1	1	-0.113	0.186	0.331	0.069	0.023	0.209	.	-0.227	0.314	0.604	0.014	0.002	0.578	7.876	0.010	2.170	19.612	1.029	6.445	0.010	1.602	18.550	-0.872	-0.294	2	.	.
8514	1	2	4	4	10	1	2	1	2	0.004	0.138	0.313	0.044	0.024	0.162	.	-0.036	0.229	0.604	0.014	0.002	0.578	7.876	0.010	2.170	19.612	1.029	6.445	0.010	1.602	18.550	-0.872	-0.294	2	.	.
8514	1	2	4	4	11	1	1	2	3	0.203	0.135	0.428	0.059	0.022	0.158	.	0.298	0.230	0.604	0.014	0.002	0.578	7.876	0.010	2.170	19.612	1.029	6.445	0.010	1.602	18.550	-0.872	-0.294	2	.	.
8514	1	2	4	4	12	1	2	2	4	0.383	0.136	0.345	0.046	0.000	0.136	.	0.607	0.228	0.604	0.014	0.002	0.578	7.876	0.010	2.170	19.612	1.029	6.445	0.010	1.602	18.550	-0.872	-0.294	2	.	.
8514	1	2	4	6	13	1	1	1	1	-0.082	0.145	0.381	0.052	0.023	0.159	.	0.150	0.228	0.604	0.003	0.002	0.578	7.876	0.010	2.170	19.612	1.029	6.445	0.010	1.602	18.550	-0.872	-0.294	2	.	.
8514	1	2	4	6	14	1	2	1	2	0.024	0.134	0.304	0.036	0.022	0.156	.	0.030	0.222	0.604	0.003	0.002	0.578	7.876	0.010	2.170	19.612	1.029	6.445	0.010	1.602	18.550	-0.872	-0.294	2	.	.
8514	1	2	4	6	15	1	1	2	3	0.475	0.134	0.333	0.037	0.005	0.161	.	0.656	0.246	0.604	0.003	0.002	0.578	7.876	0.010	2.170	19.612	1.029	6.445	0.010	1.602	18.550	-0.872	-0.294	2	.	.
8514	1	2	4	6	16	1	2	2	4	0.345	0.139	0.347	0.041	0.021	0.157	.	0.674	0.243	0.604	0.003	0.002	0.578	7.876	0.010	2.170	19.612	1.029	6.445	0.010	1.602	18.550	-0.872	-0.294	2	.	.
8514	1	2	4	7	17	1	1	1	1	0.154	0.172	0.095	0.016	0.033	0.205	.	0.239	0.289	0.604	0.004	0.002	0.578	7.876	0.010	2.170	19.612	1.029	6.445	0.010	1.602	18.550	-0.872	-0.294	2	.	.
8514	1	2	4	7	18	1	2	1	2	0.108	0.151	0.048	0.010	0.025	0.176	.	0.152	0.248	0.604	0.004	0.002	0.578	7.876	0.010	2.170	19.612	1.029	6.445	0.010	1.602	18.550	-0.872	-0.294	2	.	.
8514	1	2	4	7	19	1	1	2	3	.	.	.	.	.	.	.	.	.	.	.	.	.	.	.	.	.	.	.	.	.	.	.	.	.	.	.
8514	1	2	4	7	20	1	2	2	4	.	.	.	.	.	.	.	.	.	.	.	.	.	.	.	.	.	.	.	.	.	.	.	.	.	.	.
8514	1	2	4	8	21	1	1	1	1	0.018	0.181	0.181	0.030	0.026	0.207	.	0.028	0.290	0.618	0.004	0.001	0.578	7.876	0.010	2.170	19.612	1.029	6.445	0.010	1.602	18.550	-0.872	-0.294	2	.	.
8514	1	2	4	8	22	1	2	1	2	-0.053	0.160	0.152	0.011	0.029	0.189	.	-0.111	0.259	0.610	0.004	0.001	0.578	7.876	0.010	2.170	19.612	1.029	6.445	0.010	1.602	18.550	-0.872	-0.294	2	.	.
8514	1	2	4	8	23	1	1	2	3	0.339	0.134	0.215	0.030	0.013	0.148	.	0.520	0.202	0.610	0.004	0.001	0.578	7.876	0.010	2.170	19.612	1.029	6.445	0.010	1.602	18.550	-0.872	-0.294	2	.	.
8514	1	2	4	8	24	1	2	2	4	0.402	0.152	0.056	0.004	0.009	0.161	.	0.651	0.249	0.610	0.004	0.001	0.578	7.876	0.010	2.170	19.612	1.029	6.445	0.010	1.602	18.550	-0.872	-0.294	2	.	.
8514	1	2	4	10	25	1	1	1	1	.	.	.	.	.	.	.	.	.	.	.	.	.	.	.	.	.	.	.	.	.	.	.	.	.	.	.
8514	1	2	4	10	26	1	2	1	2	.	.	.	.	.	.	.	.	.	.	.	.	.	.	.	.	.	.	.	.	.	.	.	.	.	.	.
8514	1	2	4	10	27	1	1	2	3	0.312	0.147	0.219	0.026	0.019	0.165	.	0.504	0.245	0.604	0.006	0.002	0.578	7.876	0.010	2.170	19.612	1.029	6.445	0.010	1.602	18.550	-0.872	-0.294	2	.	.
8514	1	2	4	10	28	1	2	2	4	0.311	0.154	0.214	0.013	0.012	0.166	.	0.515	0.255	0.604	0.006	0.002	0.578	7.876	0.010	2.170	19.612	1.029	6.445	0.010	1.602	18.550	-0.872	-0.294	2	.	.
8514	1	2	4	11	29	1	1	1	1	0.000	0.142	0.204	0.019	0.017	0.159	.	0.001	0.234	0.604	0.002	0.001	0.578	7.876	0.010	2.170	19.612	1.029	6.445	0.010	1.602	18.550	-0.872	-0.294	2	.	0.259
8514	1	2	4	11	30	1	2	1	2	0.044	0.152	0.084	0.005	0.026	0.178	.	0.065	0.254	0.604	0.002	0.001	0.578	7.876	0.010	2.170	19.612	1.029	6.445	0.010	1.602	18.550	-0.872	-0.294	2	.	.
8514	1	2	4	11	31	1	1	2	3	0.319	0.123	0.180	0.018	0.012	0.135	.	0.519	0.202	0.604	0.002	0.001	0.578	7.876	0.010	2.170	19.612	1.029	6.445	0.010	1.602	18.550	-0.872	-0.294	2	.	.
8514	1	2	4	11	32	1	2	2	4	0.356	0.134	0.152	0.021	0.006	0.140	.	0.591	0.228	0.604	0.002	0.001	0.578	7.876	0.010	2.170	19.612	1.029	6.445	0.010	1.602	18.550	-0.872	-0.294	2	.	.
8514	1	2	4	2	33	3	3	3	5	0.026	0.219	0.225	0.043	0.000	0.219	.	0.089	0.985	0.226	0.005	0.002	0.366	7.670	0.011	1.543	17.854	0.499	8.558	0.009	0.924	18.391	-0.664	-0.297	2	.	.
8514	1	2	4	3	34	3	3	3	5	0.000	0.203	0.239	0.051	0.000	0.203	0.883	0.001	0.890	0.226	0.011	0.002	0.366	7.670	0.011	1.543	17.854	0.499	8.558	0.009	0.924	18.391	-0.664	-0.297	2	0.356	0.392
8514	1	2	4	4	35	3	3	3	5	0.011	0.189	0.369	0.073	0.000	0.199	.	0.085	0.878	0.226	0.013	0.002	0.366	7.670	0.011	1.543	17.854	0.499	8.558	0.009	0.924	18.391	-0.664	-0.297	2	.	.
8514	1	2	4	6	36	3	3	3	5	0.024	0.192	0.340	0.057	0.000	0.192	.	0.091	0.860	0.226	0.004	0.002	0.366	7.670	0.011	1.543	17.854	0.499	8.558	0.009	0.924	18.391	-0.664	-0.297	2	.	.
8514	1	2	4	7	37	3	3	3	5	.	.	.	.	.	.	.	.	.	.	.	.	.	.	.	.	.	.	.	.	.	.	.	.	.	.	.
8514	1	2	4	8	38	3	3	3	5	0.039	0.183	0.223	0.036	0.000	0.183	.	0.162	0.813	0.226	0.006	0.002	0.366	7.670	0.011	1.543	17.854	0.499	8.558	0.009	0.924	18.391	-0.664	-0.297	2	.	.
8514	1	2	4	10	39	3	3	3	5	.	.	.	.	.	.	.	.	.	.	.	.	.	.	.	.	.	.	.	.	.	.	.	.	.	.	.
8514	1	2	4	11	40	3	3	3	5	0.086	0.186	0.177	0.009	0.000	0.186	.	0.351	0.834	0.226	0.004	0.001	0.366	7.670	0.011	1.543	17.854	0.499	8.558	0.009	0.924	18.391	-0.664	-0.297	2	.	.
9863	2	1	1	2	1	1	1	1	1	-0.038	0.128	0.233	0.028	0.025	0.153	.	-0.073	0.215	0.605	0.009	0.003	0.541	0.226	0.007	0.899	15.448	1.002	0.225	0.007	0.905	15.620	-0.349	0.193	1	.	.
9863	2	1	1	2	2	1	2	1	2	0.126	0.124	0.147	0.016	0.018	0.143	.	0.198	0.208	0.605	0.009	0.003	0.541	0.226	0.007	0.899	15.448	1.002	0.225	0.007	0.905	15.620	-0.349	0.193	1	.	.
9863	2	1	1	2	3	1	1	2	3	0.364	0.122	0.355	0.026	0.015	0.137	.	0.591	0.203	0.605	0.010	0.003	0.541	0.226	0.007	0.899	15.448	1.002	0.225	0.007	0.905	15.620	-0.349	0.193	2	.	.
9863	2	1	1	2	4	1	2	2	4	0.395	0.135	0.120	0.013	0.011	0.146	.	0.659	0.221	0.605	0.010	0.003	0.541	0.226	0.007	0.899	15.448	1.002	0.225	0.007	0.905	15.620	-0.349	0.193	1	.	.
9863	2	1	1	3	5	1	1	1	1	-0.004	0.147	0.338	0.048	0.015	0.163	.	-0.010	0.236	0.629	0.013	0.002	0.541	0.226	0.007	0.899	15.448	1.002	0.225	0.007	0.905	15.620	-0.349	0.193	1	.	.
9863	2	1	1	3	6	1	2	1	2	0.028	0.127	0.239	0.029	0.027	0.155	.	0.051	0.206	0.617	0.013	0.002	0.541	0.226	0.007	0.899	15.448	1.002	0.225	0.007	0.905	15.620	-0.349	0.193	1	.	.
9863	2	1	1	3	7	1	1	2	3	0.168	0.129	0.330	0.049	0.009	0.138	.	0.294	0.204	0.629	0.008	0.002	0.541	0.226	0.007	0.899	15.448	1.002	0.225	0.007	0.905	15.620	-0.349	0.193	1	.	.
9863	2	1	1	3	8	1	2	2	4	0.273	0.130	0.246	0.034	0.021	0.151	.	0.440	0.209	0.617	0.008	0.002	0.541	0.226	0.007	0.899	15.448	1.002	0.225	0.007	0.905	15.620	-0.349	0.193	1	.	.
9863	2	1	1	4	9	1	1	1	1	0.002	0.153	0.161	0.010	0.017	0.170	.	0.004	0.254	0.591	0.002	0.001	0.541	0.226	0.007	0.899	15.448	1.002	0.225	0.007	0.905	15.620	-0.349	0.193	1	.	.
9863	2	1	1	4	10	1	2	1	2	0.036	0.162	0.139	0.023	0.017	0.178	.	0.024	0.279	0.591	0.002	0.001	0.541	0.226	0.007	0.899	15.448	1.002	0.225	0.007	0.905	15.620	-0.349	0.193	1	.	.
9863	2	1	1	4	11	1	1	2	3	0.388	0.122	0.145	0.018	0.007	0.130	.	0.632	0.203	0.609	0.004	0.002	0.541	0.226	0.007	0.899	15.448	1.002	0.225	0.007	0.905	15.620	-0.349	0.193	1	.	.
9863	2	1	1	4	12	1	2	2	4	0.368	0.116	0.110	0.013	0.011	0.127	.	0.590	0.197	0.591	0.010	0.003	0.541	0.226	0.007	0.899	15.448	1.002	0.225	0.007	0.905	15.620	-0.349	0.193	1	.	.
9863	2	1	1	6	13	1	1	1	1	0.094	0.132	0.447	0.058	0.025	0.157	.	0.146	0.211	0.629	0.014	0.004	0.541	0.226	0.007	0.899	15.448	1.002	0.225	0.007	0.905	15.620	-0.349	0.193	1	.	.
9863	2	1	1	6	14	1	2	1	2	0.199	0.111	0.230	0.026	0.023	0.134	.	0.321	0.179	0.629	0.014	0.004	0.541	0.226	0.007	0.899	15.448	1.002	0.225	0.007	0.905	15.620	-0.349	0.193	1	.	.
9863	2	1	1	6	15	1	1	2	3	0.394	0.147	0.395	0.058	0.030	0.177	.	0.635	0.235	0.629	0.011	0.003	0.541	0.226	0.007	0.899	15.448	1.002	0.225	0.007	0.905	15.620	-0.349	0.193	1	.	.
9863	2	1	1	6	16	1	2	2	4	0.518	0.112	0.263	0.030	0.015	0.127	.	0.835	0.180	0.629	0.011	0.003	0.541	0.226	0.007	0.899	15.448	1.002	0.225	0.007	0.905	15.620	-0.349	0.193	2	.	.
9863	2	1	1	7	17	1	1	1	1	-0.022	0.167	0.114	0.012	0.024	0.191	.	-0.033	0.254	0.624	0.006	0.002	0.541	0.226	0.007	0.899	15.448	1.002	0.225	0.007	0.905	15.620	-0.349	0.193	1	.	.
9863	2	1	1	7	18	1	2	1	2	0.184	0.126	0.092	0.012	0.024	0.150	.	0.281	0.192	0.624	0.005	0.002	0.541	0.226	0.007	0.899	15.448	1.002	0.225	0.007	0.905	15.620	-0.349	0.193	1	.	.
9863	2	1	1	7	19	1	1	2	3	.	.	.	.	.	.	.	.	.	.	.	.	.	.	.	.	.	.	.	.	.	.	.	.	.	.	.
9863	2	1	1	7	20	1	2	2	4	.	.	.	.	.	.	.	.	.	.	.	.	.	.	.	.	.	.	.	.	.	.	.	.	.	.	.
9863	2	1	1	8	21	1	1	1	1	0.070	0.126	0.395	0.051	0.043	0.168	.	0.100	0.205	0.617	0.005	0.002	0.541	0.226	0.007	0.899	15.448	1.002	0.225	0.007	0.905	15.620	-0.349	0.193	1	.	.
9863	2	1	1	8	22	1	2	1	2	0.185	0.125	0.249	0.030	0.047	0.172	.	0.298	0.201	0.617	0.005	0.002	0.541	0.226	0.007	0.899	15.448	1.002	0.225	0.007	0.905	15.620	-0.349	0.193	1	.	.
9863	2	1	1	8	23	1	1	2	3	0.497	0.121	0.197	0.020	0.007	0.128	.	0.778	0.193	0.629	0.007	0.002	0.541	0.226	0.007	0.899	15.448	1.002	0.225	0.007	0.905	15.620	-0.349	0.193	2	.	.
9863	2	1	1	8	24	1	2	2	4	0.397	0.132	0.127	0.016	0.020	0.152	.	0.636	0.209	0.629	0.006	0.002	0.541	0.226	0.007	0.899	15.448	1.002	0.225	0.007	0.905	15.620	-0.349	0.193	1	.	.
9863	2	1	1	10	25	1	1	1	1	.	.	.	.	.	.	.	.	.	.	.	.	.	.	.	.	.	.	.	.	.	.	.	.	.	.	.
9863	2	1	1	10	26	1	2	1	2	.	.	.	.	.	.	.	.	.	.	.	.	.	.	.	.	.	.	.	.	.	.	.	.	.	.	.
9863	2	1	1	10	27	1	1	2	3	0.385	0.137	0.175	0.023	0.003	0.140	.	0.606	0.219	0.629	0.006	0.002	0.541	0.226	0.007	0.899	15.448	1.002	0.225	0.007	0.905	15.620	-0.349	0.193	1	.	.
9863	2	1	1	10	28	1	2	2	4	0.150	0.150	0.116	0.014	0.027	0.178	.	0.230	0.242	0.629	0.004	0.001	0.541	0.226	0.007	0.899	15.448	1.002	0.225	0.007	0.905	15.620	-0.349	0.193	1	.	.
9863	2	1	1	11	29	1	1	1	1	0.000	0.140	0.144	0.019	0.020	0.160	.	0.001	0.223	0.629	0.005	0.002	0.541	0.226	0.007	0.899	15.448	1.002	0.225	0.007	0.905	15.620	-0.349	0.193	1	.	.
9863	2	1	1	11	30	1	2	1	2	0.135	0.140	0.103	0.011	0.016	0.156	.	0.196	0.225	0.629	0.005	0.002	0.541	0.226	0.007	0.899	15.448	1.002	0.225	0.007	0.905	15.620	-0.349	0.193	2	.	.
9863	2	1	1	11	31	1	1	2	3	0.333	0.149	0.218	0.033	0.013	0.162	.	0.519	0.237	0.629	0.007	0.002	0.541	0.226	0.007	0.899	15.448	1.002	0.225	0.007	0.905	15.620	-0.349	0.193	2	.	.
9863	2	1	1	11	32	1	2	2	4	0.372	0.130	0.094	0.011	0.012	0.142	.	0.586	0.210	0.629	0.011	0.003	0.541	0.226	0.007	0.899	15.448	1.002	0.225	0.007	0.905	15.620	-0.349	0.193	2	.	.
9863	2	1	1	2	33	3	3	3	5	-0.017	0.249	0.078	0.012	0.000	0.249	.	-0.067	0.783	0.092	0.012	0.003	0.493	0.235	0.010	0.587	23.378	1.011	0.352	0.010	0.592	23.548	-0.873	-0.380	2	.	.
9863	2	1	1	3	34	3	3	3	5	0.000	0.227	0.455	0.107	0.000	0.227	1.213	0.001	0.905	0.259	0.009	0.002	0.493	0.235	0.010	0.587	23.378	1.011	0.352	0.010	0.592	23.548	-0.873	-0.380	2	0.308	0.316
9863	2	1	1	4	35	3	3	3	5	0.021	0.206	0.241	0.052	0.000	0.206	.	0.081	0.803	0.259	0.008	0.002	0.493	0.235	0.010	0.587	23.378	1.011	0.352	0.010	0.592	23.548	-0.873	-0.380	2	.	.
9863	2	1	1	6	36	3	3	3	5	0.063	0.212	0.249	0.051	0.000	0.212	.	0.297	0.817	0.259	0.005	0.002	0.493	0.235	0.010	0.587	23.378	1.011	0.352	0.010	0.592	23.548	-0.873	-0.380	2	.	.
9863	2	1	1	7	37	3	3	3	5	.	.	.	.	.	.	.	.	.	.	.	.	.	.	.	.	.	.	.	.	.	.	.	.	.	.	.
9863	2	1	1	8	38	3	3	3	5	-0.025	0.214	0.357	0.070	0.000	0.214	.	-0.099	0.790	0.270	0.004	0.001	0.493	0.235	0.010	0.587	23.378	1.011	0.352	0.010	0.592	23.548	-0.873	-0.380	2	.	.
9863	2	1	1	10	39	3	3	3	5	.	.	.	.	.	.	.	.	.	.	.	.	.	.	.	.	.	.	.	.	.	.	.	.	.	.	.
9863	2	1	1	11	40	3	3	3	5	-0.020	0.261	0.257	0.074	0.000	0.261	.	-0.029	0.897	0.312	0.008	0.002	0.493	0.235	0.010	0.587	23.378	1.011	0.352	0.010	0.592	23.548	-0.873	-0.380	2	.	.
9863	2	1	2	2	1	1	1	1	1	0.220	0.172	0.212	0.032	0.029	0.201	.	0.327	0.283	0.618	0.001	0.001	0.507	0.184	0.003	0.379	4.841	1.058	0.637	0.003	0.378	4.983	-0.627	-0.120	2	.	.
9863	2	1	2	2	2	1	2	1	2	0.045	0.151	0.116	0.012	0.017	0.168	.	0.020	0.276	0.587	0.001	0.001	0.507	0.184	0.003	0.379	4.841	1.058	0.637	0.003	0.378	4.983	-0.627	-0.120	2	.	.
9863	2	1	2	2	3	1	1	2	3	0.436	0.141	0.199	0.026	0.018	0.159	.	0.665	0.231	0.631	0.001	0.001	0.507	0.184	0.003	0.379	4.841	1.058	0.637	0.003	0.378	4.983	-0.627	-0.120	2	.	.
9863	2	1	2	2	4	1	2	2	4	0.525	0.132	0.126	0.013	0.026	0.157	.	0.816	0.222	0.631	0.001	0.001	0.507	0.184	0.003	0.379	4.841	1.058	0.637	0.003	0.378	4.983	-0.627	-0.120	2	.	.
9863	2	1	2	3	5	1	1	1	1	0.055	0.143	0.332	0.044	0.020	0.163	.	0.095	0.237	0.631	0.005	0.001	0.507	0.184	0.003	0.379	4.841	1.058	0.637	0.003	0.378	4.983	-0.627	-0.120	2	.	.
9863	2	1	2	3	6	1	2	1	2	0.078	0.143	0.228	0.028	0.020	0.163	.	0.072	0.245	0.631	0.005	0.001	0.507	0.184	0.003	0.379	4.841	1.058	0.637	0.003	0.378	4.983	-0.627	-0.120	2	.	.
9863	2	1	2	3	7	1	1	2	3	0.371	0.163	0.444	0.081	0.007	0.170	.	0.567	0.267	0.631	0.005	0.001	0.507	0.184	0.003	0.379	4.841	1.058	0.637	0.003	0.378	4.983	-0.627	-0.120	2	.	.
9863	2	1	2	3	8	1	2	2	4	0.428	0.154	0.125	0.017	0.028	0.182	.	0.710	0.266	0.618	0.005	0.001	0.507	0.184	0.003	0.379	4.841	1.058	0.637	0.003	0.378	4.983	-0.627	-0.120	2	.	.
9863	2	1	2	4	9	1	1	1	1	0.105	0.127	0.235	0.027	0.036	0.164	.	0.136	0.217	0.602	0.002	0.001	0.507	0.184	0.003	0.379	4.841	1.058	0.637	0.003	0.378	4.983	-0.627	-0.120	2	.	.
9863	2	1	2	4	10	1	2	1	2	0.052	0.162	0.103	0.019	0.029	0.190	.	0.065	0.224	0.602	0.002	0.001	0.507	0.184	0.003	0.379	4.841	1.058	0.637	0.003	0.378	4.983	-0.627	-0.120	2	.	.
9863	2	1	2	4	11	1	1	2	3	0.345	0.148	0.482	0.082	0.013	0.161	.	0.500	0.231	0.631	0.004	0.001	0.507	0.184	0.003	0.379	4.841	1.058	0.637	0.003	0.378	4.983	-0.627	-0.120	2	.	.
9863	2	1	2	4	12	1	2	2	4	0.451	0.151	0.199	0.030	0.013	0.164	.	0.621	0.207	0.602	0.002	0.001	0.507	0.184	0.003	0.379	4.841	1.058	0.637	0.003	0.378	4.983	-0.627	-0.120	2	.	.

9863	2	1	2	6	13	1	1	1	1	0.072	0.164	0.214	0.034	0.017	0.181	.	0.134	0.276	0.615	0.009	0.003	0.507	0.184	0.003	0.379	4.841	1.058	0.637	0.003	0.378	4.983	-0.627	-0.120	2	.	.
9863	2	1	2	6	14	1	2	1	2	0.120	0.148	0.186	0.028	0.031	0.178	.	0.258	0.251	0.600	0.009	0.003	0.507	0.184	0.003	0.379	4.841	1.058	0.637	0.003	0.378	4.983	-0.627	-0.120	2	.	.
9863	2	1	2	6	15	1	1	2	3	0.479	0.150	0.178	0.025	0.016	0.166	.	0.814	0.250	0.624	0.009	0.003	0.507	0.184	0.003	0.379	4.841	1.058	0.637	0.003	0.378	4.983	-0.627	-0.120	2	.	.
9863	2	1	2	6	16	1	2	2	4	0.455	0.149	0.125	0.014	0.005	0.153	.	0.743	0.249	0.618	0.009	0.003	0.507	0.184	0.003	0.379	4.841	1.058	0.637	0.003	0.378	4.983	-0.627	-0.120	2	.	.
9863	2	1	2	7	17	1	1	1	1	-0.067	0.131	0.035	0.004	0.043	0.174	.	-0.161	0.316	0.415	0.001	0.001	0.507	0.184	0.003	0.379	4.841	1.058	0.637	0.003	0.378	4.983	-0.627	-0.120	2	.	.
9863	2	1	2	7	18	1	2	1	2	0.117	0.101	0.020	0.002	0.015	0.116	.	0.270	0.233	0.415	0.001	0.001	0.507	0.184	0.003	0.379	4.841	1.058	0.637	0.003	0.378	4.983	-0.627	-0.120	2	.	.
9863	2	1	2	7	19	1	1	2	3	.	.	.	.	.	.	.	.	.	.	.	.	.	.	.	.	.	.	.	.	.	.	.	.	.	.	.
9863	2	1	2	7	20	1	2	2	4	.	.	.	.	.	.	.	.	.	.	.	.	.	.	.	.	.	.	.	.	.	.	.	.	.	.	.
9863	2	1	2	8	21	1	1	1	1	0.042	0.162	0.170	0.029	0.021	0.183	.	0.073	0.268	0.634	0.005	0.002	0.507	0.184	0.003	0.379	4.841	1.058	0.637	0.003	0.378	4.983	-0.627	-0.120	2	.	.
9863	2	1	2	8	22	1	2	1	2	0.076	0.145	0.101	0.014	0.020	0.165	.	0.123	0.229	0.634	0.005	0.002	0.507	0.184	0.003	0.379	4.841	1.058	0.637	0.003	0.378	4.983	-0.627	-0.120	2	.	.
9863	2	1	2	8	23	1	1	2	3	0.358	0.136	0.188	0.024	0.012	0.148	.	0.568	0.224	0.634	0.005	0.002	0.507	0.184	0.003	0.379	4.841	1.058	0.637	0.003	0.378	4.983	-0.627	-0.120	2	.	.
9863	2	1	2	8	24	1	2	2	4	0.425	0.149	0.090	0.013	0.006	0.155	.	0.695	0.242	0.634	0.005	0.002	0.507	0.184	0.003	0.379	4.841	1.058	0.637	0.003	0.378	4.983	-0.627	-0.120	2	.	.
9863	2	1	2	10	25	1	1	1	1	.	.	.	.	.	.	.	.	.	.	.	.	.	.	.	.	.	.	.	.	.	.	.	.	.	.	.
9863	2	1	2	10	26	1	2	1	2	.	.	.	.	.	.	.	.	.	.	.	.	.	.	.	.	.	.	.	.	.	.	.	.	.	.	.
9863	2	1	2	10	27	1	1	2	3	0.273	0.173	0.302	0.053	0.012	0.185	.	0.399	0.276	0.634	0.004	0.001	0.507	0.184	0.003	0.379	4.841	1.058	0.637	0.003	0.378	4.983	-0.627	-0.120	2	.	.
9863	2	1	2	10	28	1	2	2	4	0.338	0.167	0.167	0.028	0.008	0.176	.	0.526	0.274	0.634	0.004	0.001	0.507	0.184	0.003	0.379	4.841	1.058	0.637	0.003	0.378	4.983	-0.627	-0.120	2	.	.
9863	2	1	2	11	29	1	1	1	1	0.000	0.153	0.227	0.030	0.022	0.175	.	0.001	0.245	0.634	0.001	0.001	0.507	0.184	0.003	0.379	4.841	1.058	0.637	0.003	0.378	4.983	-0.627	-0.120	2	.	.
9863	2	1	2	11	30	1	2	1	2	0.046	0.132	0.242	0.032	0.015	0.146	.	0.063	0.216	0.634	0.001	0.001	0.507	0.184	0.003	0.379	4.841	1.058	0.637	0.003	0.378	4.983	-0.627	-0.120	2	.	.
9863	2	1	2	11	31	1	1	2	3	0.288	0.138	0.368	0.048	0.013	0.151	.	0.452	0.224	0.634	0.001	0.001	0.507	0.184	0.003	0.379	4.841	1.058	0.637	0.003	0.378	4.983	-0.627	-0.112	2	.	.
9863	2	1	2	11	32	1	2	2	4	0.406	0.143	0.220	0.026	0.008	0.151	.	0.647	0.228	0.634	0.001	0.001	0.507	0.184	0.003	0.379	4.841	1.058	0.637	0.003	0.378	4.983	-0.627	-0.120	2	.	.
9863	2	1	2	2	33	3	3	3	5	-0.055	0.235	0.126	0.017	0.000	0.235	.	-0.204	0.912	0.257	0.001	0.001	0.554	0.165	0.004	0.412	4.125	1.002	0.385	0.004	0.428	4.250	-0.215	0.339	1	.	.
9863	2	1	2	3	34	3	3	3	5	0.000	0.204	0.339	0.072	0.000	0.204	1.189	0.001	0.802	0.257	0.005	0.001	0.554	0.165	0.004	0.412	4.125	1.002	0.385	0.004	0.428	4.250	-0.215	0.339	1	1.118	0.426
9863	2	1	2	4	35	3	3	3	5	-0.126	0.193	0.194	0.037	0.000	0.193	.	-0.558	0.854	0.266	0.002	0.001	0.554	0.165	0.004	0.412	4.125	1.002	0.385	0.004	0.428	4.250	-0.215	0.339	1	.	.
9863	2	1	2	6	36	3	3	3	5	0.013	0.266	0.079	0.013	0.000	0.266	.	0.042	0.869	0.306	0.002	0.001	0.554	0.165	0.004	0.412	4.125	1.002	0.385	0.004	0.428	4.250	-0.215	0.339	1	.	.
9863	2	1	2	7	37	3	3	3	5	.	.	.	.	.	.	.	.	.	.	.	.	.	.	.	.	.	.	.	.	.	.	.	.	.	.	.
9863	2	1	2	8	38	3	3	3	5	0.073	0.253	0.177	0.041	0.000	0.253	.	0.280	0.984	0.257	0.005	0.002	0.554	0.165	0.004	0.412	4.125	1.002	0.385	0.004	0.428	4.250	-0.215	0.339	1	.	.
9863	2	1	2	10	39	3	3	3	5	.	.	.	.	.	.	.	.	.	.	.	.	.	.	.	.	.	.	.	.	.	.	.	.	.	.	.
9863	2	1	2	11	40	3	3	3	5	0.047	0.235	0.284	0.064	0.000	0.235	.	0.137	0.929	0.257	0.001	0.001	0.554	0.165	0.004	0.412	4.125	1.002	0.385	0.004	0.428	4.250	-0.215	0.339	1	.	.
9863	2	1	3	2	1	1	1	1	1	0.013	0.149	0.448	0.058	0.017	0.166	.	0.046	0.255	0.585	0.002	0.001	0.574	18.293	0.027	76.381	776.247	1.178	18.120	0.027	75.196	790.947	-0.379	0.192	1	.	.
9863	2	1	3	2	2	1	2	1	2	0.087	0.118	0.224	0.023	0.023	0.141	.	0.195	0.203	0.585	0.002	0.001	0.574	18.293	0.027	76.381	776.247	1.178	18.120	0.027	75.196	790.947	-0.379	0.192	1	.	.
9863	2	1	3	2	3	1	1	2	3	0.285	0.157	0.185	0.024	0.043	0.201	.	0.519	0.265	0.585	0.002	0.001	0.574	18.293	0.027	76.381	776.247	1.178	18.120	0.027	75.196	790.947	-0.379	0.192	1	.	.
9863	2	1	3	2	4	1	2	2	4	0.427	0.121	0.087	0.009	0.052	0.173	.	0.780	0.226	0.557	0.002	0.001	0.574	18.293	0.027	76.381	776.247	1.178	18.120	0.027	75.196	790.947	-0.379	0.192	1	.	.
9863	2	1	3	3	5	1	1	1	1	0.017	0.144	0.147	0.021	0.018	0.162	.	0.047	0.235	0.618	0.006	0.001	0.574	18.293	0.027	76.381	776.247	1.178	18.120	0.027	75.196	790.947	-0.379	0.192	1	.	.
9863	2	1	3	3	6	1	2	1	2	0.112	0.149	0.124	0.016	0.015	0.164	.	0.213	0.235	0.618	0.006	0.001	0.574	18.293	0.027	76.381	776.247	1.178	18.120	0.027	75.196	790.947	-0.379	0.192	1	.	.
9863	2	1	3	3	7	1	1	2	3	0.319	0.126	0.283	0.034	0.012	0.138	.	0.538	0.209	0.618	0.006	0.001	0.574	18.293	0.027	76.381	776.247	1.178	18.120	0.027	75.196	790.947	-0.379	0.192	1	.	.
9863	2	1	3	3	8	1	2	2	4	0.482	0.128	0.141	0.016	0.012	0.140	.	0.803	0.213	0.618	0.006	0.001	0.574	18.293	0.027	76.381	776.247	1.178	18.120	0.027	75.196	790.947	-0.379	0.192	1	.	.
9863	2	1	3	4	9	1	1	1	1	.	.	.	.	.	.	.	.	.	.	.	.	.	.	.	.	.	.	.	.	.	.	.	.	.	.	.
9863	2	1	3	4	10	1	2	1	2	.	.	.	.	.	.	.	.	.	.	.	.	.	.	.	.	.	.	.	.	.	.	.	.	.	.	.
9863	2	1	3	4	11	1	1	2	3	.	.	.	.	.	.	.	.	.	.	.	.	.	.	.	.	.	.	.	.	.	.	.	.	.	.	.
9863	2	1	3	4	12	1	2	2	4	.	.	.	.	.	.	.	.	.	.	.	.	.	.	.	.	.	.	.	.	.	.	.	.	.	.	.
9863	2	1	3	6	13	1	1	1	1	-0.004	0.146	0.144	0.018	0.016	0.163	.	0.005	0.240	0.611	0.003	0.001	0.574	18.293	0.027	76.381	776.247	1.178	18.120	0.027	75.196	790.947	-0.379	0.192	1	.	.
9863	2	1	3	6	14	1	2	1	2	-0.011	0.123	0.085	0.009	0.025	0.147	.	0.001	0.206	0.611	0.003	0.001	0.574	18.293	0.027	76.381	776.247	1.178	18.120	0.027	75.196	790.947	-0.379	0.192	1	.	.
9863	2	1	3	6	15	1	1	2	3	0.294	0.150	0.088	0.013	0.025	0.175	.	0.493	0.246	0.611	0.003	0.001	0.574	18.293	0.027	76.381	776.247	1.178	18.120	0.027	75.196	790.947	-0.379	0.192	1	.	.
9863	2	1	3	6	16	1	2	2	4	0.420	0.137	0.060	0.007	0.021	0.158	.	0.712	0.228	0.611	0.003	0.001	0.574	18.293	0.027	76.381	776.247	1.178	18.120	0.027	75.196	790.947	-0.379	0.192	1	.	.
9863	2	1	3	7	17	1	1	1	1	0.036	0.130	0.170	0.018	0.052	0.182	.	0.072	0.213	0.611	0.001	0.001	0.574	18.293	0.027	76.381	776.247	1.178	18.120	0.027	75.196	790.947	-0.379	0.192	1	.	.
9863	2	1	3	7	18	1	2	1	2	0.013	0.129	0.071	0.007	0.027	0.156	.	-0.007	0.215	0.611	0.001	0.001	0.574	18.293	0.027	76.381	776.247	1.178	18.120	0.027	75.196	790.947	-0.379	0.192	1	.	.
9863	2	1	3	7	19	1	1	2	3	.	.	.	.	.	.	.	.	.	.	.	.	.	.	.	.	.	.	.	.	.	.	.	.	.	.	.
9863	2	1	3	7	20	1	2	2	4	.	.	.	.	.	.	.	.	.	.	.	.	.	.	.	.	.	.	.	.	.	.	.	.	.	.	.
9863	2	1	3	8	21	1	1	1	1	0.056	0.128	0.377	0.048	0.016	0.144	.	0.099	0.210	0.614	0.003	0.001	0.574	18.293	0.027	76.381	776.247	1.178	18.120	0.027	75.196	790.947	-0.379	0.192	1	.	.
9863	2	1	3	8	22	1	2	1	2	0.123	0.127	0.258	0.028	0.015	0.141	.	0.213	0.209	0.614	0.003	0.001	0.574	18.293	0.027	76.381	776.247	1.178	18.120	0.027	75.196	790.947	-0.379	0.192	1	.	.
9863	2	1	3	8	23	1	1	2	3	0.433	0.132	0.355	0.042	0.013	0.145	.	0.681	0.220	0.632	0.003	0.001	0.574	18.293	0.027	76.381	776.247	1.178	18.120	0.027	75.196	790.947	-0.379	0.192	1	.	.
9863	2	1	3	8	24	1	2	2	4	0.451	0.121	0.185	0.021	0.016	0.138	.	0.717	0.199	0.632	0.003	0.001	0.574	18.293	0.027	76.381	776.247	1.178	18.120	0.027	75.196	790.947	-0.379	0.192	1	.	.
9863	2	1	3	10	25	1	1	1	1	.	.	.	.	.	.	.	.	.	.	.	.	.	.	.	.	.	.	.	.	.	.	.	.	.	.	.
9863	2	1	3	10	26	1	2	1	2	.	.	.	.	.	.	.	.	.	.	.	.	.	.	.	.	.	.	.	.	.	.	.	.	.	.	.
9863	2	1	3	10	27	1	1	2	3	0.237	0.161	0.419	0.073	0.013	0.174	.	0.347	0.258	0.632	0.003	0.001	0.574	18.293	0.027	76.381	776.247	1.178	18.120	0.027	75.196	790.947	-0.379	0.192	1	.	.
9863	2	1	3	10	28	1	2	2	4	0.365	0.150	0.264	0.063	0.012	0.162	.	0.567	0.237	0.632	0.003	0.001	0.574	18.293	0.027	76.381	776.247	1.178	18.120	0.027	75.196	790.947	-0.379	0.192	1	.	.
9863	2	1	3	11	29	1	1	1	1	0.000	0.146	0.270	0.030	0.678	0.824	.	0.001	0.237	0.632	0.003	0.001	0.574	18.293	0.027	76.381	776.247	1.178	18.120	0.027	75.196	790.947	-0.379	0.192	1	.	.
9863	2	1	3	11	30	1	2	1	2	0.058	0.144	0.080	0.009	0.034	0.178	.	0.093	0.224	0.632	0.003	0.001	0.574	18.293	0.027	76.381	776.247	1.178	18.120	0.027	75.196	790.947	-0.379	0.192	1	.	.
9863	2	1	3	11	31	1	1	2	3	0.338	0.145	0.243	0.028	0.017	0.162	.	0.542	0.234	0.632	0.003	0.001	0.574	18.293	0.027	76.381	776.247	1.178	18.120	0.027	75.196	790.947	-0.379	0.192	1	.	.
9863	2	1	3	11	32	1	2	2	4	0.433	0.141	0.168	0.018	0.019	0.160	.	0.669	0.228	0.632	0.003	0.001	0.574	18.293	0.027	76.381	776.247	1.178	18.120	0.027	75.196	790.947	-0.379	0.192	1	.	.
9863	2	1	3	2	33	3	3	3	5	0.018	0.209	0.164	0.025	0.000	0.209	.	0.134	0.931	0.228	0.003	0.001	0.576	20.829	0.031	33.536	478.790	1.167	19.938	0.025	35.438	495.824	-1.185	-0.611	2	.	.
9863	2	1	3	3	34	3	3	3	5	0.000	0.203	0.108	0.014	0.000	0.203	1.012	0.001	0.919	0.228	0.009	0.001	0.576	20.829	0.031	33.536	478.790	1.167	19.938	0.025	35.438	495.824	-1.185	-0.611	2	0.974	0.627
9863	2	1	3	4	35	3	3	3	5	.	.	.	.	.	.	.	.	.	.	.	.	.	.	.	.	.	.	.	.	.	.	.	.	.	.	.
9863	2	1	3	6	36	3	3	3	5	0.092	0.204	0.205	0.051	0.000	0.204	.	0.402	0.899	0.228	0.003	0.001	0.576	20.829	0.031	33.536	478.790	1.167	19.938	0.025	35.438	495.824	-1.185	-0.611	2	.	.
9863	2	1	3	7	37	3	3	3	5	.	.	.	.	.	.	.	.	.	.	.	.	.	.	.	.	.	.	.	.	.	.	.	.	.	.	.
9863	2	1	3	8	38	3	3	3	5	-0.065	0.187	0.232	0.055	0.000	0.187	.	-0.267	0.892	0.217	0.005	0.001	0.576	20.829	0.031	33.536	478.790	1.167	19.938	0.025	35.438	495.824	-1.185	-0.611	2	.	.
9863	2	1	3	10	39	3	3	3	5	.	.	.	.	.	.	.	.	.	.	.	.	.	.	.	.	.	.	.	.	.	.	.	.	.	.	.
9863	2	1	3	11	40	3	3	3	5	0.006	0.194	0.180	0.018	0.000	0.194	.	-0.028	0.765	0.428	0.003	0.001	0.576	20.829	0.031	33.536	478.790	1.167	19.938	0.025	35.438	495.824	-1.185	-0.611	2	.	.
9863	2	1	4	2	1	1	1	1	1	.	.	.	.	.	.	.	.	.	.	.	.	.	.	.	.	.	.	.	.	.	.	.	.	.	.	.
9863	2	1	4	2	2	1	2	1	2	.	.	.	.	.	.	.	.	.	.	.	.	.	.	.	.	.	.	.	.	.	.	.	.	.	.	.
9863	2	1	4	2	3	1	1	2	3	.	.	.	.	.	.	.	.	.	.	.	.	.	.	.	.	.	.	.	.	.	.	.	.	.	.	.
9863	2	1	4	2	4	1	2	2	4	.	.	.	.	.	.	.	.	.	.	.	.	.	.	.	.	.	.	.	.	.	.	.	.	.	.	.
9863	2	1	4	3	5	1	1	1	1	0.056	0.146	0.328	0.048	0.020	0.166	.	0.090	0.207	0.700	0.005	0.002	0.581	0.494	0.002	0.506	6.870	1.095	0.518	0.002	0.518	6.870	-0.382	0.199	1	.	.
9863	2	1	4	3	6	1	2	1	2	0.018	0.155	0.194	0.029	0.012	0.167	.	0.035	0.221	0.700	0.005	0.002	0.581	0.494	0.002	0.506	6.870	1.095	0.518	0.002	0.518	6.870	-0.382	0.199	1	.	.
9863	2	1	4	3	7	1	1	2	3	0.354	0.142	0.324	0.043	0.014	0.156	.	0.512	0.201	0.700	0.005	0.002	0.581	0.494	0.002	0.506	6.870	1.095	0.518	0.002	0.518	6.870	-0.382	0.199	1	.	.
9863	2	1	4	3	8	1	2	2	4	0.437	0.133	0.181	0.021	0.006	0.139	.	0.631	0.189	0.700	0.005	0.002	0.581	0.494	0.002	0.506	6.870	1.095	0.518	0.002	0.518	6.870	-0.382	0.199	1	.	.
9863	2	1	4	4	9	1	1	1	1	0.151	0.100	0.104	0.010	0.028	0.128	.	0.247	0.158	0.638	0.001	0.001	0.581	0.494	0.002	0.506	6.870	1.095	0.518	0.002	0.518	6.870	-0.382	0.199	1	.	.
9863	2	1	4	4	10	1	2	1	2	0.154	0.122	0.020	0.002	0.012	0.134	.	0.258	0.193	0.638	0.001	0.001	0.581	0.494	0.002	0.506	6.870	1.095	0.518	0.002	0.518	6.870	-0.382	0.199	1	.	.
9863	2	1	4	4	11	1	1	2	3	0.613	0.111	0.098	0.010	0.011	0.122	.	1.000	0.181	0.613	0.001	0.001	0.581	0.494	0.002	0.506	6.870	1.095	0.518	0.002	0.518	6.870	-0.382	0.199	1	.	.
9863	2	1	4	4	12	1	2	2	4	0.719	0.122	0.008	0.002	0.000	0.122	.	1.173	0.199	0.613	0.001	0.001	0.581	0.494	0.002	0.506	6.870	1.095	0.518	0.002	0.518	6.870	-0.382	0.199	1	.	.
9863	2	1	4	6	13	1	1	1	1	0.012	0.199	0.483	0.092	0.039	0.238	.	0.028	0.286	0.700	0.008	0.002	0.581	0.494	0.002	0.506	6.870	1.095	0.518	0.002	0.518	6.870	-0.382	0.199	1	.	.
9863	2	1	4	6	14	1	2	1	2	-0.025	0.173	0.322	0.058	0.022	0.195	.	-0.046	0.250	0.700	0.008	0.002	0.581	0.494	0.002	0.506	6.870	1.095	0.518	0.002	0.518	6.870	-0.382	0.199	1	.	.
9863	2	1	4	6	15	1	1	2	3	0.431	0.150	0.599	0.098	0.009	0.159	.	0.616	0.217	0.700	0.008	0.002	0.581	0.494	0.002	0.506	6.870	1.095	0.518	0.002	0.518	6.870	-0.382	0.199	1	.	.
9863	2	1	4	6	16	1	2	2	4	0.417	0.147	0.383	0.056	0.014	0.160	.	0.609	0.209	0.700	0.008	0.002	0.581	0.494	0.002	0.506	6.870	1.095	0.518	0.002	0.518	6.870	-0.382	0.199	1	.	.
9863	2	1	4	7	17	1	1	1	1	-0.020	0.166	0.023	0.004	0.044	0.210	.	-0.036	0.234	0.713	0.006	0.002	0.581	0.494	0.002	0.506	6.870	1.095	0.518	0.002	0.518	6.870	-0.382	0.199	1	.	.
9863	2	1	4	7	18	1	2	1	2	-0.023	0.150	0.011	0.002	0.051	0.202	.	-0.019	0.217	0.713	0.002	0.001	0.581	0.494	0.002	0.506	6.870	1.095	0.518	0.002	0.518	6.870	-0.382	0.199	1	.	.
9863	2	1	4	7	19	1	1	2	3	.	.	.	.	.	.	.	.	.	.	.	.	.	.	.	.	.	.	.	.	.	.	.	.	.	.	.
9863	2	1	4	7	20	1	2	2	4	.	.	.	.	.	.	.	.	.	.	.	.	.	.	.	.	.	.	.	.	.	.	.	.	.	.	.
9863	2	1	4	8	21	1	1	1	1	-0.008	0.152	0.333	0.050	0.029	0.181	.	-0.015	0.220	0.700	0.004	0.001	0.581	0.494	0.002	0.506	6.870	1.095	0.518	0.002	0.518	6.870	-0.382	0.199	1	.	.
9863	2	1	4	8	22	1	2	1	2	-0.024	0.141	0.147	0.021	0.027	0.168	.	-0.034	0.203	0.700	0.005	0.001	0.581	0.494	0.002	0.506	6.870	1.095	0.518	0.002	0.518	6.870	-0.382	0.199	1	.	.
9863	2	1	4	8	23	1	1	2	3	0.341	0.146	0.312	0.050	0.008	0.155	.	0.493	0.212	0.700	0.005	0.001	0.581	0.494	0.002	0.506	6.870	1.095	0.518	0.002	0.518	6.870	-0.382	0.199	1	.	.
9863	2	1	4	8	24	1	2	2	4	0.439	0.148	0.218	0.035	0.012	0.160	.	0.623	0.214	0.700	0.005	0.001	0.581	0.494	0.002	0.506	6.870	1.095	0.518	0.002	0.518	6.870	-0.382	0.199	1	.	.
9863	2	1	4	10	25	1	1	1	1	.	.	.	.	.	.	.	.	.	.	.	.	.	.	.	.	.	.	.	.	.	.	.	.	.	.	.
9863	2	1	4	10	26	1	2	1	2	.	.	.	.	.	.	.	.	.	.	.	.	.	.	.	.	.	.	.	.	.	.	.	.	.	.	.
9863	2	1	4	10	27	1	1	2	3	0.300	0.200	0.381	0.089	0.018	0.218	.	0.425	0.288	0.700	0.004	0.001	0.581	0.494	0.002	0.506	6.870	1.095	0.518	0.002	0.518	6.870	-0.382	0.199	1	.	.
9863	2	1	4	10	28	1	2	2	4	0.431	0.190	0.247	0.054	0.005	0.195	.	0.617	0.274	0.700	0.003	0.001	0.581	0.494	0.002	0.506	6.870	1.095	0.518	0.002	0.518	6.870	-0.382	0.199	1	.	.
9863	2	1	4	11	29	1	1	1	1	0.000	0.152	0.117	0.016	0.032	0.183	.	0.001	0.219	0.700	0.005	0.001	0.581	0.494	0.002	0.506	6.870	1.095	0.518	0.002	0.518	6.870	-0.382	0.199	1	.	.
9863	2	1	4	11	30	1	2	1	2	0.038	0.179	0.060	0.010	0.041	0.220	.	0.047	0.258	0.700	0.005	0.001	0.581	0.494	0.002	0.506	6.870	1.095	0.518	0.002	0.518	6.870	-0.382	0.199	1	.	.
9863	2	1	4	11	31	1	1	2	3	0.416	0.156	0.111	0.016	0.008	0.165	.	0.594	0.226	0.700	0.005	0.001	0.581	0.494	0.002	0.506	6.870	1.095	0.518	0.002	0.518	6.870	-0.382	0.199	1	.	.
9863	2	1	4	11	32	1	2	2	4	0.427	0.145	0.052	0.007	0.009	0.153	.	0.610	0.208	0.700	0.005	0.001	0.581	0.494	0.002	0.506	6.870	1.095	0.518	0.002	0.518	6.870	-0.382	0.199	1	.	.
9863	2	1	4	2	33	3	3	3	5	-0.045	0.237	0.158	0.041	0.000	0.237	.	0.001	1.029	0.230	0.004	0.002	0.428	0.422	0.002	0.422	11.073	0.843	0.658	0.002	0.394	11.197	-0.691	-0.263	2	.	.
9863	2	1	4	3	34	3	3	3	5	0.000	0.211	0.229	0.052	0.000	0.211	0.875	0.001	0.924	0.230	0.009	0.003	0.428	0.422	0.002	0.422	11.073	0.843	0.658	0.002	0.394	11.197	-0.691	-0.263	2	1.372	0.411
9863	2	1	4	4	35	3	3	3	5	.	.	.	.	.	.	.	.	.	.	.	.	.	.	.	.	.	.	.	.	.	.	.	.	.	.	.
9863	2	1	4	6	36	3	3	3	5	0.030	0.167	0.253	0.038	0.000	0.167	.	0.136	0.722	0.230	0.006	0.002	0.428	0.422	0.002	0.422	11.073	0.843	0.658	0.002	0.394	11.197	-0.691	-0.263	2	.	.
9863	2	1	4	7	37	3	3	3	5	.	.	.	.	.	.	.	.	.	.	.	.	.	.	.	.	.	.	.	.	.	.	.	.	.	.	.
9863	2	1	4	8	38	3	3	3	5	0.068	0.162	0.112	0.018	0.000	0.162	.	0.285	0.707	0.230	0.007	0.002	0.428	0.422	0.002	0.422	11.073	0.843	0.658	0.002	0.394	11.197	-0.691	-0.263	2	.	.
9863	2	1	4	10	39	3	3	3	5	.	.	.	.	.	.	.	.	.	.	.	.	.	.	.	.	.	.	.	.	.	.	.	.	.	.	.
9863	2	1	4	11	40	3	3	3	5	0.044	0.156	0.061	0.008	0.000	0.156	.	0.185	0.680	0.230	0.008	0.002	0.428	0.422	0.002	0.422	11.073	0.843	0.658	0.002	0.394	11.197	-0.691	-0.263	2	.	.
9822	2	2	1	2	1	1	1	1	1	0.229	0.094	0.270	0.020	0.016	0.110	.	0.364	0.149	0.629	0.001	0.001	0.701	0.487	0.010	0.043	2.401	1.272	0.544	0.008	0.085	2.118	-0.393	0.308	2	.	.
9822	2	2	1	2	2	1	2	1	2	0.056	0.139	0.220	0.030	0.038	0.177	.	0.089	0.221	0.629	0.001	0.001	0.701	0.487	0.010	0.043	2.401	1.272	0.544	0.008	0.085	2.118	-0.393	0.308	2	.	.
9822	2	2	1	2	3	1	1	2	3	0.329	0.111	0.290	0.030	0.011	0.122	.	0.523	0.176	0.629	0.001	0.001	0.701	0.487	0.010	0.043	2.401	1.272	0.544	0.008	0.085	2.118	-0.393	0.308	2	.	.
9822	2	2	1	2	4	1	2	2	4	0.496	0.117	0.030	0.010	0.000	0.117	.	0.789	0.186	0.629	0.001	0.001	0.701	0.487	0.010	0.043	2.401	1.272	0.544	0.008	0.085	2.118	-0.393	0.308	2	.	.
9822	2	2	1	3	5	1	1	1	1	-0.041	0.115	0.337	0.037	0.023	0.139	.	-0.061	0.182	0.637	0.012	0.005	0.701	0.487	0.010	0.043	2.401	1.272	0.544	0.008	0.085	2.118	-0.393	0.308	2	.	.
9822	2	2	1	3	6	1	2	1	2	-0.010	0.150	0.140	0.021	0.018	0.168	.	-0.006	0.239	0.637	0.012	0.005	0.701	0.487	0.010	0.043	2.401	1.272	0.544	0.008	0.085	2.118	-0.393	0.308	2	.	.
9822	2	2	1	3	7	1	1	2	3	0.314	0.120	0.254	0.029	0.012	0.132	.	0.503	0.190	0.637	0.012	0.005	0.701	0.487	0.010	0.043	2.401	1.272	0.544	0.008	0.085	2.118	-0.393	0.308	2	.	.
9822	2	2	1	3	8	1	2	2	4	0.121	0.140	0.160	0.020	0.007	0.147	.	0.167	0.222	0.637	0.012	0.005	0.701	0.487	0.010	0.043	2.401	1.272	0.544	0.008	0.085	2.118	-0.393	0.308	2	.	.
9822	2	2	1	4	9	1	1	1	1	-0.005	0.135	0.827	0.117	0.026	0.161	.	-0.011	0.214	0.637	0.003	0.001	0.701	0.487	0.010	0.043	2.401	1.272	0.544	0.008	0.085	2.118	-0.393	0.308	2	.	.
9822	2	2	1	4	10	1	2	1	2	0.071	0.114	0.432	0.050	0.055	0.169	.	0.111	0.179	0.637	0.003	0.001	0.701	0.487	0.010	0.043	2.401	1.272	0.544	0.008	0.085	2.118	-0.393	0.308	2	.	.
9822	2	2	1	4	11	1	1	2	3	0.311	0.115	0.615	0.072	0.017	0.132	.	0.486	0.181	0.637	0.003	0.001	0.701	0.487	0.010	0.043	2.401	1.272	0.544	0.008	0.085	2.118	-0.393	0.308	2	.	.
9822	2	2	1	4	12	1	2	2	4	0.407	0.139	0.239	0.034	0.003	0.142	.	0.646	0.218	0.637	0.003	0.001	0.701	0.487	0.010	0.043	2.401	1.272	0.544	0.008	0.085	2.118	-0.393	0.308	2	.	.
9822	2	2	1	6	13	1	1	1	1	0.076	0.148	0.440	0.073	0.019	0.167	.	0.120	0.232	0.637	0.005	0.001	0.701	0.487	0.010	0.043	2.401	1.272	0.544	0.008	0.085	2.118	-0.393	0.308	2	.	.
9822	2	2	1	6	14	1	2	1	2	0.007	0.148	0.312	0.051	0.023	0.171	.	0.017	0.233	0.637	0.005	0.001	0.701	0.487	0.010	0.043	2.401	1.272	0.544	0.008	0.085	2.118	-0.393	0.308	2	.	.
9822	2	2	1	6	15	1	1	2	3	0.365	0.117	0.356	0.039	0.012	0.128	.	0.579	0.185	0.637	0.005	0.001	0.701	0.487	0.010	0.043	2.401	1.272	0.544	0.008	0.085	2.118	-0.393	0.308	2	.	.
9822	2	2	1	6	16	1	2	2	4	0.404	0.135	0.268	0.037	0.004	0.138	.	0.639	0.212	0.637	0.005	0.001	0.701	0.487	0.010	0.043	2.401	1.272	0.544	0.008	0.085	2.118	-0.393	0.308	2	.	.
9822	2	2	1	7	17	1	1	1	1	.	.	.	.	.	.	.	.	.	.	.	.	.	.	.	.	.	.	.	.	.	.	.	.	.	.	.
9822	2	2	1	7	18	1	2	1	2	.	.	.	.	.	.	.	.	.	.	.	.	.	.	.	.	.	.	.	.	.	.	.	.	.	.	.
9822	2	2	1	7	19	1	1	2	3	.	.	.	.	.	.	.	.	.	.	.	.	.	.	.	.	.	.	.	.	.	.	.	.	.	.	.
9822	2	2	1	7	20	1	2	2	4	.	.	.	.	.	.	.	.	.	.	.	.	.	.	.	.	.	.	.	.	.	.	.	.	.	.	.
9822	2	2	1	8	21	1	1	1	1	-0.028	0.128	0.346	0.049	0.024	0.152	.	-0.048	0.201	0.637	0.009	0.003	0.701	0.487	0.010	0.043	2.401	1.272	0.544	0.008	0.085	2.118	-0.393	0.308	2	.	.
9822	2	2	1	8	22	1	2	1	2	-0.064	0.143	0.162	0.025	0.022	0.164	.	-0.085	0.222	0.637	0.009	0.003	0.701	0.487	0.010	0.043	2.401	1.272	0.544	0.008	0.085	2.118	-0.393	0.308	2	.	.
9822	2	2	1	8	23	1	1	2	3	0.255	0.146	0.295	0.046	0.015	0.161	.	0.405	0.228	0.637	0.009	0.003	0.701	0.487	0.010	0.043	2.401	1.272	0.544	0.008	0.085	2.118	-0.393	0.308	2	.	.
9822	2	2	1	8	24	1	2	2	4	0.328	0.140	0.231	0.032	0.006	0.146	.	0.522	0.220	0.637	0.009	0.003	0.701	0.487	0.010	0.043	2.401	1.272	0.544	0.008	0.085	2.118	-0.393	0.308	2	.	.
9822	2	2	1	10	25	1	1	1	1	.	.	.	.	.	.	.	.	.	.	.	.	.	.	.	.	.	.	.	.	.	.	.	.	.	.	.
9822	2	2	1	10	26	1	2	1	2	.	.	.	.	.	.	.	.	.	.	.	.	.	.	.	.	.	.	.	.	.	.	.	.	.	.	.
9822	2	2	1	10	27	1	1	2	3	0.189	0.175	0.308	0.058	0.017	0.192	.	0.302	0.276	0.637	0.015	0.006	0.701	0.487	0.010	0.043	2.401	1.272	0.544	0.008	0.085	2.118	-0.393	0.308	2	.	.
9822	2	2	1	10	28	1	2	2	4	0.333	0.168	0.123	0.023	0.005	0.173	.	0.526	0.264	0.637	0.015	0.006	0.701	0.487	0.010	0.043	2.401	1.272	0.544	0.008	0.085	2.118	-0.393	0.308	2	.	.
9822	2	2	1	11	29	1	1	1	1	0.000	0.141	0.202	0.030	0.024	0.165	.	0.001	0.221	0.637	0.002	0.001	0.701	0.487	0.010	0.043	2.401	1.272	0.544	0.008	0.085	2.118	-0.393	0.308	2	0.863	0.613
9822	2	2	1	11	30	1	2	1	2	-0.053	0.160	0.098	0.015	0.020	0.180	.	-0.075	0.251	0.637	0.002	0.001	0.701	0.487	0.010	0.043	2.401	1.272	0.544	0.008	0.085	2.118	-0.393	0.308	2	.	.
9822	2	2	1	11	31	1	1	2	3	0.236	0.157	0.259	0.039	0.020	0.176	.	0.375	0.245	0.637	0.002	0.001	0.701	0.487	0.010	0.043	2.401	1.272	0.544	0.008	0.085	2.118	-0.393	0.308	2	.	.

9822	2	2	1	11	32	1	2	2	4	0.355	0.139	0.125	0.016	0.006	0.145	.	0.559	0.219	0.637	0.002	0.001	0.701	0.487	0.010	0.043	2.401	1.272	0.544	0.008	0.085	2.118	-0.393	0.308	2	.	.
9822	2	2	1	2	33	3	3	3	5	-0.039	0.212	0.039	0.010	0.000	0.212	.	-0.195	1.000	0.212	0.001	0.001	0.496	0.358	0.013	0.505	142.635	0.942	0.259	0.015	0.431	142.605	-1.875	-1.384	2	.	.
9822	2	2	1	3	34	3	3	3	5	0.000	0.132	0.396	0.065	0.000	0.132	2.731	0.001	0.623	0.212	0.012	0.005	0.496	0.358	0.013	0.505	142.635	0.942	0.259	0.015	0.431	142.605	-1.875	-1.379	2	.	.
9822	2	2	1	4	35	3	3	3	5	-0.009	0.128	0.642	0.085	0.000	0.128	.	-0.127	0.383	0.212	0.003	0.001	0.496	0.358	0.013	0.505	142.635	0.942	0.259	0.015	0.431	142.605	-1.875	-1.379	2	.	.
9822	2	2	1	6	36	3	3	3	5	0.047	0.141	0.274	0.039	0.000	0.141	.	0.226	0.680	0.212	0.005	0.001	0.496	0.358	0.013	0.505	142.635	0.942	0.259	0.015	0.431	142.605	-1.875	-1.379	2	.	.
9822	2	2	1	7	37	3	3	3	5	.	.	.	.	.	.	.	.	.	.	.	.	.	.	.	.	.	.	.	.	.	.	.	.	.	.	.
9822	2	2	1	8	38	3	3	3	5	0.030	0.184	0.348	0.082	0.000	0.184	.	0.126	0.861	0.212	0.009	0.003	0.496	0.358	0.013	0.505	142.635	0.942	0.259	0.015	0.431	142.605	-1.875	-1.379	2	.	.
9822	2	2	1	10	39	3	3	3	5	.	.	.	.	.	.	.	.	.	.	.	.	.	.	.	.	.	.	.	.	.	.	.	.	.	.	.
9822	2	2	1	11	40	3	3	3	5	0.000	0.259	0.215	0.056	0.000	0.259	.	0.028	1.131	0.234	0.002	0.001	0.496	0.358	0.013	0.505	142.635	0.942	0.259	0.015	0.431	142.605	-1.875	-1.379	2	.	.
9822	2	2	2	2	1	1	1	1	1	0.200	0.134	0.034	0.004	0.011	0.145	.	0.315	0.211	0.633	0.003	0.001	0.568	0.433	0.003	0.291	62.583	1.198	0.445	0.003	0.311	62.967	-0.234	0.334	1	.	.
9822	2	2	2	2	2	1	2	1	2	0.000	0.139	0.011	0.002	0.012	0.151	.	0.001	0.219	0.633	0.003	0.001	0.568	0.433	0.003	0.291	62.583	1.198	0.445	0.003	0.311	62.967	-0.234	0.334	1	.	.
9822	2	2	2	2	3	1	1	2	3	0.334	0.172	0.041	0.007	0.022	0.194	.	0.526	0.271	0.633	0.003	0.001	0.568	0.433	0.003	0.291	62.583	1.198	0.445	0.003	0.311	62.967	-0.234	0.334	1	.	.
9822	2	2	2	2	4	1	2	2	4	0.535	0.122	0.021	0.002	0.000	0.122	.	0.843	0.192	0.633	0.003	0.001	0.568	0.433	0.003	0.291	62.583	1.198	0.445	0.003	0.311	62.967	-0.234	0.334	1	.	.
9822	2	2	2	3	5	1	1	1	1	0.278	0.145	0.280	0.039	0.023	0.168	.	0.441	0.230	0.628	0.018	0.002	0.568	0.433	0.003	0.291	62.583	1.198	0.445	0.003	0.311	62.967	-0.234	0.334	1	.	.
9822	2	2	2	3	6	1	2	1	2	0.086	0.149	0.110	0.016	0.029	0.178	.	0.136	0.238	0.628	0.018	0.002	0.568	0.433	0.003	0.291	62.583	1.198	0.445	0.003	0.311	62.967	-0.234	0.334	1	.	.
9822	2	2	2	3	7	1	1	2	3	0.573	0.167	0.404	0.080	0.006	0.172	.	0.910	0.265	0.628	0.018	0.002	0.568	0.433	0.003	0.291	62.583	1.198	0.445	0.003	0.311	62.967	-0.234	0.334	1	.	.
9822	2	2	2	3	8	1	2	2	4	0.527	0.141	0.184	0.027	0.015	0.156	.	0.837	0.224	0.628	0.018	0.002	0.568	0.433	0.003	0.291	62.583	1.198	0.445	0.003	0.311	62.967	-0.234	0.334	1	.	.
9822	2	2	2	4	9	1	1	1	1	.	.	.	.	.	.	.	.	.	.	.	.	.	.	.	.	.	.	.	.	.	.	.	.	.	.	.
9822	2	2	2	4	10	1	2	1	2	.	.	.	.	.	.	.	.	.	.	.	.	.	.	.	.	.	.	.	.	.	.	.	.	.	.	.
9822	2	2	2	4	11	1	1	2	3	.	.	.	.	.	.	.	.	.	.	.	.	.	.	.	.	.	.	.	.	.	.	.	.	.	.	.
9822	2	2	2	4	12	1	2	2	4	.	.	.	.	.	.	.	.	.	.	.	.	.	.	.	.	.	.	.	.	.	.	.	.	.	.	.
9822	2	2	2	6	13	1	1	1	1	0.182	0.145	0.227	0.040	0.023	0.168	.	0.288	0.231	0.628	0.009	0.001	0.568	0.433	0.003	0.291	62.583	1.198	0.445	0.003	0.311	62.967	-0.234	0.334	1	.	.
9822	2	2	2	6	14	1	2	1	2	0.179	0.121	0.154	0.020	0.016	0.138	.	0.282	0.193	0.628	0.009	0.001	0.568	0.433	0.003	0.291	62.583	1.198	0.445	0.003	0.311	62.967	-0.234	0.334	1	.	.
9822	2	2	2	6	15	1	1	2	3	0.455	0.130	0.340	0.041	0.014	0.144	.	0.725	0.207	0.628	0.009	0.001	0.568	0.433	0.003	0.291	62.583	1.198	0.445	0.003	0.311	62.967	-0.234	0.334	1	.	.
9822	2	2	2	6	16	1	2	2	4	0.557	0.128	0.129	0.015	0.003	0.131	.	0.883	0.204	0.628	0.009	0.001	0.568	0.433	0.003	0.291	62.583	1.198	0.445	0.003	0.311	62.967	-0.234	0.334	1	.	.
9822	2	2	2	7	17	1	1	1	1	-0.050	0.206	0.114	0.023	0.028	0.234	.	-0.079	0.324	0.622	0.001	0.001	0.568	0.433	0.003	0.291	62.583	1.198	0.445	0.003	0.311	62.967	-0.234	0.334	1	.	.
9822	2	2	2	7	18	1	2	1	2	0.195	0.128	0.011	0.001	0.039	0.167	.	0.307	0.202	0.622	0.001	0.001	0.568	0.433	0.003	0.291	62.583	1.198	0.445	0.003	0.311	62.967	-0.234	0.334	1	.	.
9822	2	2	2	7	19	1	1	2	3	.	.	.	.	.	.	.	.	.	.	.	.	.	.	.	.	.	.	.	.	.	.	.	.	.	.	.
9822	2	2	2	7	20	1	2	2	4	.	.	.	.	.	.	.	.	.	.	.	.	.	.	.	.	.	.	.	.	.	.	.	.	.	.	.
9822	2	2	2	8	21	1	1	1	1	0.112	0.137	0.163	0.019	0.025	0.162	.	0.180	0.219	0.628	0.009	0.003	0.568	0.433	0.003	0.291	62.583	1.198	0.445	0.003	0.311	62.967	-0.234	0.334	1	.	.
9822	2	2	2	8	22	1	2	1	2	0.179	0.121	0.154	0.020	0.016	0.138	.	0.282	0.193	0.628	0.009	0.001	0.568	0.433	0.003	0.291	62.583	1.198	0.445	0.003	0.311	62.967	-0.234	0.334	1	.	.
9822	2	2	2	8	23	1	1	2	3	0.455	0.130	0.340	0.041	0.014	0.144	.	0.725	0.207	0.628	0.009	0.001	0.568	0.433	0.003	0.291	62.583	1.198	0.445	0.003	0.311	62.967	-0.234	0.334	1	.	.
9822	2	2	2	8	24	1	2	2	4	0.557	0.128	0.129	0.015	0.003	0.131	.	0.883	0.204	0.628	0.009	0.001	0.568	0.433	0.003	0.291	62.583	1.198	0.445	0.003	0.311	62.967	-0.234	0.334	1	.	.
9822	2	2	2	10	25	1	1	1	1	.	.	.	.	.	.	.	.	.	.	.	.	.	.	.	.	.	.	.	.	.	.	.	.	.	.	.
9822	2	2	2	10	26	1	2	1	2	.	.	.	.	.	.	.	.	.	.	.	.	.	.	.	.	.	.	.	.	.	.	.	.	.	.	.
9822	2	2	2	10	27	1	1	2	3	0.364	0.136	0.259	0.035	0.013	0.149	.	0.582	0.216	0.628	0.009	0.003	0.568	0.433	0.003	0.291	62.583	1.198	0.445	0.003	0.311	62.967	-0.234	0.334	1	.	.
9822	2	2	2	10	28	1	2	2	4	0.167	0.161	0.215	0.035	0.012	0.174	.	0.269	0.256	0.628	0.009	0.003	0.568	0.433	0.003	0.291	62.583	1.198	0.445	0.003	0.311	62.967	-0.234	0.334	1	.	.
9822	2	2	2	11	29	1	1	1	1	0.000	0.141	0.085	0.012	0.022	0.164	.	0.001	0.225	0.628	0.003	0.001	0.568	0.433	0.003	0.291	62.583	1.198	0.445	0.003	0.311	62.967	-0.234	0.334	1	.	.
9822	2	2	2	11	30	1	2	1	2	0.111	0.137	0.058	0.007	0.014	0.151	.	0.178	0.218	0.628	0.003	0.001	0.568	0.433	0.003	0.291	62.583	1.198	0.445	0.003	0.311	62.967	-0.234	0.334	1	.	.
9822	2	2	2	11	31	1	1	2	3	0.443	0.128	0.094	0.012	0.003	0.131	.	0.704	0.203	0.628	0.003	0.001	0.568	0.433	0.003	0.291	62.583	1.198	0.445	0.003	0.311	62.967	-0.234	0.334	1	.	.
9822	2	2	2	11	32	1	2	2	4	0.320	0.140	0.058	0.008	0.037	0.177	.	0.511	0.223	0.628	0.003	0.001	0.568	0.433	0.003	0.291	62.583	1.198	0.445	0.003	0.311	62.967	-0.234	0.334	1	.	.
9822	2	2	2	2	33	3	3	3	5	-0.045	0.235	0.085	0.013	0.000	0.235	.	-0.160	0.958	0.246	0.003	0.001	0.445	0.502	0.001	0.169	64.335	1.010	0.473	0.003	0.180	64.751	-0.744	-0.299	2	.	.
9822	2	2	2	3	34	3	3	3	5	0.000	0.195	0.433	0.081	0.000	0.195	0.872	0.000	0.810	0.246	0.018	0.002	0.445	0.502	0.001	0.169	64.335	1.010	0.473	0.003	0.180	64.751	-0.744	-0.299	2	1.364	0.293
9822	2	2	2	4	35	3	3	3	5	.	.	.	.	.	.	.	.	.	.	.	.	.	.	.	.	.	.	.	.	.	.	.	.	.	.	.
9822	2	2	2	6	36	3	3	3	5	0.040	0.188	0.437	0.156	0.000	0.188	.	0.144	0.748	0.246	0.009	0.001	0.445	0.502	0.001	0.169	64.335	1.010	0.473	0.003	0.180	64.751	-0.744	-0.299	2	.	.
9822	2	2	2	7	37	3	3	3	5	.	.	.	.	.	.	.	.	.	.	.	.	.	.	.	.	.	.	.	.	.	.	.	.	.	.	.
9822	2	2	2	8	38	3	3	3	5	0.026	0.164	0.265	0.047	0.000	0.164	.	0.111	0.668	0.246	0.009	0.003	0.445	0.502	0.001	0.169	64.335	1.010	0.473	0.003	0.180	64.751	-0.744	-0.299	2	.	.
9822	2	2	2	10	39	3	3	3	5	.	.	.	.	.	.	.	.	.	.	.	.	.	.	.	.	.	.	.	.	.	.	.	.	.	.	.
9822	2	2	2	11	40	3	3	3	5	0.001	0.205	0.097	0.021	0.000	0.205	.	0.019	0.841	0.246	0.003	0.001	0.445	0.502	0.001	0.169	64.335	1.010	0.473	0.003	0.180	64.751	-0.744	-0.299	2	.	.
9822	2	2	3	2	1	1	1	1	1	.	.	.	.	.	.	.	.	.	.	.	.	.	.	.	.	.	.	.	.	.	.	.	.	.	.	.
9822	2	2	3	2	2	1	2	1	2	.	.	.	.	.	.	.	.	.	.	.	.	.	.	.	.	.	.	.	.	.	.	.	.	.	.	.
9822	2	2	3	2	3	1	1	2	3	.	.	.	.	.	.	.	.	.	.	.	.	.	.	.	.	.	.	.	.	.	.	.	.	.	.	.
9822	2	2	3	2	4	1	2	2	4	.	.	.	.	.	.	.	.	.	.	.	.	.	.	.	.	.	.	.	.	.	.	.	.	.	.	.
9822	2	2	3	3	5	1	1	1	1	-0.087	0.168	0.180	0.031	0.023	0.191	.	-0.115	0.255	0.668	0.008	0.001	0.468	5.207	0.006	27.301	200.529	1.022	4.481	0.006	27.165	203.974	-1.231	-0.764	2	.	.
9822	2	2	3	3	6	1	2	1	2	-0.032	0.161	0.068	0.012	0.020	0.180	.	-0.044	0.238	0.668	0.008	0.001	0.468	5.207	0.006	27.301	200.529	1.022	4.481	0.006	27.165	203.974	-1.231	-0.764	2	.	.
9822	2	2	3	3	7	1	1	2	3	0.349	0.151	0.145	0.022	0.010	0.161	.	0.525	0.227	0.668	0.008	0.001	0.468	5.207	0.006	27.301	200.529	1.022	4.481	0.006	27.165	203.974	-1.231	-0.764	2	.	.
9822	2	2	3	3	8	1	2	2	4	0.319	0.147	0.081	0.013	0.009	0.156	.	0.493	0.219	0.668	0.008	0.001	0.468	5.207	0.006	27.301	200.529	1.022	4.481	0.006	27.165	203.974	-1.231	-0.764	2	.	.
9822	2	2	3	4	9	1	1	1	1	.	.	.	.	.	.	.	.	.	.	.	.	.	.	.	.	.	.	.	.	.	.	.	.	.	.	.
9822	2	2	3	4	10	1	2	1	2	.	.	.	.	.	.	.	.	.	.	.	.	.	.	.	.	.	.	.	.	.	.	.	.	.	.	.
9822	2	2	3	4	11	1	1	2	3	.	.	.	.	.	.	.	.	.	.	.	.	.	.	.	.	.	.	.	.	.	.	.	.	.	.	.
9822	2	2	3	4	12	1	2	2	4	.	.	.	.	.	.	.	.	.	.	.	.	.	.	.	.	.	.	.	.	.	.	.	.	.	.	.
9822	2	2	3	6	13	1	1	1	1	-0.080	0.156	0.339	0.053	0.027	0.183	.	-0.112	0.231	0.668	0.011	0.002	0.468	5.207	0.006	27.301	200.529	1.022	4.481	0.006	27.165	203.974	-1.231	-0.764	2	.	.
9822	2	2	3	6	14	1	2	1	2	0.117	0.136	0.141	0.020	0.025	0.161	.	0.179	0.202	0.668	0.011	0.002	0.468	5.207	0.006	27.301	200.529	1.022	4.481	0.006	27.165	203.974	-1.231	-0.764	2	.	.
9822	2	2	3	6	15	1	1	2	3	0.395	0.136	0.212	0.029	0.016	0.151	.	0.599	0.205	0.668	0.011	0.002	0.468	5.207	0.006	27.301	200.529	1.022	4.481	0.006	27.165	203.974	-1.231	-0.764	2	.	.
9822	2	2	3	6	16	1	2	2	4	0.404	0.170	0.086	0.014	0.007	0.177	.	0.605	0.258	0.668	0.011	0.002	0.468	5.207	0.006	27.301	200.529	1.022	4.481	0.006	27.165	203.974	-1.231	-0.764	2	.	.
9822	2	2	3	7	17	1	1	1	1	-0.123	0.175	0.105	0.016	0.028	0.203	.	-0.182	0.263	0.668	0.002	0.001	0.468	5.207	0.006	27.301	200.529	1.022	4.481	0.006	27.165	203.974	-1.231	-0.764	2	.	.
9822	2	2	3	7	18	1	2	1	2	0.096	0.131	0.075	0.008	0.021	0.153	.	0.147	0.199	0.668	0.002	0.001	0.468	5.207	0.006	27.301	200.529	1.022	4.481	0.006	27.165	203.974	-1.231	-0.764	2	.	.
9822	2	2	3	7	19	1	1	2	3	.	.	.	.	.	.	.	.	.	.	.	.	.	.	.	.	.	.	.	.	.	.	.	.	.	.	.
9822	2	2	3	7	20	1	2	2	4	.	.	.	.	.	.	.	.	.	.	.	.	.	.	.	.	.	.	.	.	.	.	.	.	.	.	.
9822	2	2	3	8	21	1	1	1	1	0.002	0.194	0.549	0.099	0.016	0.209	.	0.001	0.288	0.668	0.014	0.003	0.468	5.207	0.006	27.301	200.529	1.022	4.481	0.006	27.165	203.974	-1.231	-0.764	2	.	.
9822	2	2	3	8	22	1	2	1	2	0.225	0.168	0.356	0.060	0.017	0.185	.	0.331	0.255	0.668	0.014	0.003	0.468	5.207	0.006	27.301	200.529	1.022	4.481	0.006	27.165	203.974	-1.231	-0.764	2	.	.
9822	2	2	3	8	23	1	1	2	3	0.476	0.140	0.547	0.083	0.018	0.157	.	0.706	0.213	0.668	0.014	0.003	0.468	5.207	0.006	27.301	200.529	1.022	4.481	0.006	27.165	203.974	-1.231	-0.764	2	.	.
9822	2	2	3	8	24	1	2	2	4	0.541	0.128	0.230	0.030	0.007	0.135	.	0.816	0.195	0.668	0.014	0.003	0.468	5.207	0.006	27.301	200.529	1.022	4.481	0.006	27.165	203.974	-1.231	-0.764	2	.	.
9822	2	2	3	10	25	1	1	1	1	.	.	.	.	.	.	.	.	.	.	.	.	.	.	.	.	.	.	.	.	.	.	.	.	.	.	.
9822	2	2	3	10	26	1	2	1	2	.	.	.	.	.	.	.	.	.	.	.	.	.	.	.	.	.	.	.	.	.	.	.	.	.	.	.
9822	2	2	3	10	27	1	1	2	3	0.275	0.184	0.208	0.035	0.014	0.198	.	0.416	0.270	0.668	0.009	0.001	0.468	5.207	0.006	27.301	200.529	1.022	4.481	0.006	27.165	203.974	-1.231	-0.764	2	.	.
9822	2	2	3	10	28	1	2	2	4	0.305	0.182	0.149	0.025	0.008	0.190	.	0.469	0.271	0.668	0.009	0.001	0.468	5.207	0.006	27.301	200.529	1.022	4.481	0.006	27.165	203.974	-1.231	-0.764	2	.	.
9822	2	2	3	11	29	1	1	1	1	0.000	0.168	0.123	0.023	0.026	0.194	.	0.001	0.249	0.668	0.007	0.002	0.468	5.207	0.006	27.301	200.529	1.022	4.481	0.006	27.165	203.974	-1.231	-0.764	2	.	.
9822	2	2	3	11	30	1	2	1	2	0.013	0.136	0.058	0.007	0.028	0.164	.	0.029	0.204	0.668	0.007	0.002	0.468	5.207	0.006	27.301	200.529	1.022	4.481	0.006	27.165	203.974	-1.231	-0.764	2	.	.
9822	2	2	3	11	31	1	1	2	3	0.339	0.145	0.113	0.018	0.013	0.158	.	0.510	0.215	0.668	0.007	0.002	0.468	5.207	0.006	27.301	200.529	1.022	4.481	0.006	27.165	203.974	-1.231	-0.764	2	.	.
9822	2	2	3	11	32	1	2	2	4	0.447	0.114	0.064	0.007	0.002	0.117	.	0.675	0.170	0.668	0.007	0.002	0.468	5.207	0.006	27.301	200.529	1.022	4.481	0.006	27.165	203.974	-1.231	-0.764	2	.	.
9822	2	2	3	2	33	3	3	3	5	-0.015	0.224	0.010	0.002	0.000	0.224	.	-0.030	0.822	0.330	0.001	0.001	0.418	3.619	0.004	11.909	340.279	0.794	3.889	0.004	11.501	342.420	0.082	0.500	1	.	.
9822	2	2	3	3	34	3	3	3	5	0.000	0.196	0.080	0.017	0.000	0.196	1.005	0.000	0.833	0.236	0.008	0.001	0.418	3.619	0.004	11.909	340.279	0.794	3.889	0.004	11.501	342.420	0.082	0.500	1	4.368	0.877
9822	2	2	3	4	35	3	3	3	5	.	.	.	.	.	.	.	.	.	.	.	.	.	.	.	.	.	.	.	.	.	.	.	.	.	.	.
9822	2	2	3	6	36	3	3	3	5	0.029	0.167	0.118	0.019	0.000	0.167	.	0.117	0.707	0.236	0.011	0.002	0.418	3.619	0.004	11.909	340.279	0.794	3.889	0.004	11.501	342.420	0.082	0.500	1	.	.
9822	2	2	3	7	37	3	3	3	5	.	.	.	.	.	.	.	.	.	.	.	.	.	.	.	.	.	.	.	.	.	.	.	.	.	.	.
9822	2	2	3	8	38	3	3	3	5	0.017	0.164	0.366	0.058	0.000	0.164	.	0.070	0.699	0.236	0.014	0.003	0.418	3.619	0.004	11.909	340.279	0.794	3.889	0.004	11.501	342.420	0.082	0.500	1	.	.
9822	2	2	3	10	39	3	3	3	5	.	.	.	.	.	.	.	.	.	.	.	.	.	.	.	.	.	.	.	.	.	.	.	.	.	.	.
9822	2	2	3	11	40	3	3	3	5	-0.010	0.165	0.083	0.013	0.000	0.165	.	-0.039	0.691	0.236	0.007	0.002	0.418	3.619	0.004	11.909	340.279	0.794	3.889	0.004	11.501	342.420	0.082	0.500	1	.	.
9822	2	2	4	2	1	1	1	1	1	-0.032	0.140	0.040	0.006	0.031	0.171	.	-0.057	0.218	0.639	0.004	0.001	0.549	0.303	0.017	0.162	5.620	1.102	0.304	0.017	0.164	5.852	-0.455	0.094	2	.	.
9822	2	2	4	2	2	1	2	1	2	0.107	0.122	0.030	0.003	0.022	0.144	.	0.150	0.186	0.657	0.004	0.001	0.549	0.303	0.017	0.162	5.620	1.102	0.304	0.017	0.164	5.852	-0.455	0.094	2	.	.
9822	2	2	4	2	3	1	1	2	3	0.369	0.121	0.035	0.004	0.013	0.134	.	0.526	0.177	0.687	0.004	0.001	0.549	0.303	0.017	0.162	5.620	1.102	0.304	0.017	0.164	5.852	-0.455	0.094	2	.	.
9822	2	2	4	2	4	1	2	2	4	0.400	0.113	0.025	0.003	0.004	0.117	.	0.571	0.166	0.687	0.004	0.001	0.549	0.303	0.017	0.162	5.620	1.102	0.304	0.017	0.164	5.852	-0.455	0.094	2	.	.
9822	2	2	4	3	5	1	1	1	1	0.101	0.158	0.552	0.083	0.027	0.184	.	0.164	0.239	0.650	0.015	0.003	0.549	0.303	0.017	0.162	5.620	1.102	0.304	0.017	0.164	5.852	-0.455	0.094	2	.	.
9822	2	2	4	3	6	1	2	1	2	0.059	0.127	0.320	0.041	0.020	0.147	.	0.103	0.196	0.650	0.015	0.003	0.549	0.303	0.017	0.162	5.620	1.102	0.304	0.017	0.164	5.852	-0.455	0.094	2	.	.
9822	2	2	4	3	7	1	1	2	3	0.414	0.127	0.402	0.051	0.010	0.137	.	0.649	0.195	0.650	0.015	0.003	0.549	0.303	0.017	0.162	5.620	1.102	0.304	0.017	0.164	5.852	-0.455	0.094	2	.	.
9822	2	2	4	3	8	1	2	2	4	0.422	0.129	0.265	0.036	0.012	0.141	.	0.646	0.194	0.650	0.015	0.003	0.549	0.303	0.017	0.162	5.620	1.102	0.304	0.017	0.164	5.852	-0.455	0.094	2	.	.
9822	2	2	4	4	9	1	1	1	1	0.382	0.112	0.362	0.039	0.019	0.131	.	0.664	0.197	0.571	0.002	0.001	0.549	0.303	0.017	0.162	5.620	1.102	0.304	0.017	0.164	5.852	-0.455	0.094	2	.	.
9822	2	2	4	4	10	1	2	1	2	0.189	0.159	0.259	0.041	0.022	0.181	.	0.328	0.278	0.571	0.002	0.001	0.549	0.303	0.017	0.162	5.620	1.102	0.304	0.017	0.164	5.852	-0.455	0.094	2	.	.
9822	2	2	4	4	11	1	1	2	3	0.566	0.126	0.274	0.034	0.006	0.131	.	0.992	0.220	0.571	0.002	0.001	0.549	0.303	0.017	0.162	5.620	1.102	0.304	0.017	0.164	5.852	-0.455	0.094	2	.	.
9822	2	2	4	4	12	1	2	2	4	0.569	0.139	0.303	0.042	0.014	0.153	.	0.990	0.243	0.571	0.002	0.001	0.549	0.303	0.017	0.162	5.620	1.102	0.304	0.017	0.164	5.852	-0.455	0.094	2	.	.
9822	2	2	4	6	13	1	1	1	1	-0.097	0.120	0.298	0.032	0.034	0.154	.	-0.156	0.186	0.650	0.003	0.001	0.549	0.303	0.017	0.162	5.620	1.102	0.304	0.017	0.164	5.852	-0.455	0.094	2	.	.
9822	2	2	4	6	14	1	2	1	2	-0.112	0.139	0.180	0.023	0.034	0.173	.	-0.164	0.216	0.650	0.003	0.001	0.549	0.303	0.017	0.162	5.620	1.102	0.304	0.017	0.164	5.852	-0.455	0.094	2	.	.
9822	2	2	4	6	15	1	1	2	3	0.222	0.128	0.267	0.034	0.015	0.143	.	0.343	0.203	0.650	0.003	0.001	0.549	0.303	0.017	0.162	5.620	1.102	0.304	0.017	0.164	5.852	-0.455	0.094	2	.	.
9822	2	2	4	6	16	1	2	2	4	0.137	0.127	0.136	0.015	0.011	0.138	.	0.184	0.193	0.668	0.003	0.001	0.549	0.303	0.017	0.162	5.620	1.102	0.304	0.017	0.164	5.852	-0.455	0.094	2	.	.
9822	2	2	4	7	17	1	1	1	1	0.022	0.184	0.066	0.012	0.031	0.215	.	0.033	0.278	0.657	0.002	0.001	0.549	0.303	0.017	0.162	5.620	1.102	0.304	0.017	0.164	5.852	-0.455	0.094	2	.	.
9822	2	2	4	7	18	1	2	1	2	0.126	0.167	0.032	0.005	0.028	0.195	.	0.176	0.252	0.657	0.002	0.001	0.549	0.303	0.017	0.162	5.620	1.102	0.304	0.017	0.164	5.852	-0.455	0.094	2	.	.
9822	2	2	4	7	19	1	1	2	3	.	.	.	.	.	.	.	.	.	.	.	.	.	.	.	.	.	.	.	.	.	.	.	.	.	.	.
9822	2	2	4	7	20	1	2	2	4	.	.	.	.	.	.	.	.	.	.	.	.	.	.	.	.	.	.	.	.	.	.	.	.	.	.	.
9822	2	2	4	8	21	1	1	1	1	0.033	0.135	0.558	0.091	0.019	0.154	.	0.056	0.208	0.559	0.005	0.001	0.549	0.303	0.017	0.162	5.620	1.102	0.304	0.017	0.164	5.852	-0.455	0.094	2	.	.
9822	2	2	4	8	22	1	2	1	2	0.010	0.151	0.276	0.045	0.029	0.180	.	0.014	0.229	0.652	0.005	0.001	0.549	0.303	0.017	0.162	5.620	1.102	0.304	0.017	0.164	5.852	-0.455	0.094	2	.	.
9822	2	2	4	8	23	1	1	2	3	0.459	0.134	0.612	0.084	0.002	0.135	.	0.704	0.209	0.652	0.005	0.001	0.549	0.303	0.017	0.162	5.620	1.102	0.304	0.017	0.164	5.852	-0.455	0.094	2	.	.
9822	2	2	4	8	24	1	2	2	4	0.375	0.136	0.403	0.053	0.013	0.149	.	0.568	0.210	0.652	0.005	0.001	0.549	0.303	0.017	0.162	5.620	1.102	0.304	0.017	0.164	5.852	-0.455	0.094	2	.	.
9822	2	2	4	10	25	1	1	1	1	.	.	.	.	.	.	.	.	.	.	.	.	.	.	.	.	.	.	.	.	.	.	.	.	.	.	.
9822	2	2	4	10	26	1	2	1	2	.	.	.	.	.	.	.	.	.	.	.	.	.	.	.	.	.	.	.	.	.	.	.	.	.	.	.
9822	2	2	4	10	27	1	1	2	3	0.084	0.161	0.090	0.010	0.022	0.183	.	0.146	0.280	0.574	0.005	0.001	0.549	0.303	0.017	0.162	5.620	1.102	0.304	0.017	0.164	5.852	-0.455	0.094	2	.	.
9822	2	2	4	10	28	1	2	2	4	0.279	0.150	0.080	0.010	0.000	0.150	.	0.486	0.261	0.574	0.005	0.001	0.549	0.303	0.017	0.162	5.620	1.102	0.304	0.017	0.164	5.852	-0.455	0.094	2	.	.
9822	2	2	4	11	29	1	1	1	1	0.000	0.138	0.358	0.048	0.018	0.156	.	0.001	0.217	0.641	0.004	0.001	0.549	0.303	0.017	0.162	5.620	1.102	0.304	0.017	0.164	5.852	-0.455	0.094	2	.	.
9822	2	2	4	11	30	1	2	1	2	0.041	0.115	0.157	0.018	0.020	0.135	.	0.051	0.183	0.641	0.004	0.001	0.549	0.303	0.017	0.162	5.620	1.102	0.304	0.017	0.164	5.852	-0.455	0.094	2	.	.
9822	2	2	4	11	31	1	1	2	3	0.322	0.133	0.310	0.043	0.017	0.149	.	0.503	0.210	0.641	0.004	0.001	0.549	0.303	0.017	0.162	5.620	1.102	0.304	0.017	0.164	5.852	-0.455	0.094	2	.	.
9822	2	2	4	11	32	1	2	2	4	0.329	0.137	0.189	0.027	0.014	0.150	.	0.504	0.216	0.641	0.004	0.001	0.549	0.303	0.017	0.162	5.620	1.102	0.304	0.017	0.164	5.852	-0.455	0.094	2	.	.
9822	2	2	4	2	33	3	3	3	5	-0.035	0.239	0.065	0.011	0.000	0.239	.	-0.145	0.968	0.249	0.004	0.001	0.557	0.333	0.017	0.042	6.151	0.954	0.304	0.018	0.044	6.371	-0.547	0.010	2	.	.
9822	2	2	4	3	34	3	3	3	5	0.000	0.238	0.425	0.098	0.000	0.238	0.875	0.000	0.968	0.249	0.015	0.003	0.557	0.333	0.017	0.042	6.151	0.954	0.304	0.018	0.044	6.371	-0.547	0.010	2	.	0.237
9822	2	2	4	4	35	3	3	3	5	0.078	0.175	0.224	0.029	0.000	0.175	.	0.293	0.763	0.226	0.002	0.001	0.557	0.333	0.017	0.042	6.151	0.954	0.304	0.018	0.044	6.371	-0.547	0.010	2	.	.
9822	2	2	4	6	36	3	3	3	5	0.060	0.234	0.103	0.024	0.000	0.234	.	0.209	0.850	0.274	0.003	0.001	0.557	0.333	0.017	0.042	6.151	0.954	0.304	0.018	0.044	6.371	-0.547	0.010	2	.	.
9822	2	2	4	7	37	3	3	3	5	.	.	.	.	.	.	.	.	.	.	.	.	.	.	.	.	.	.	.	.	.	.	.	.	.	.	.
9822	2	2	4	8	38	3	3	3	5	-0.011	0.228	0.631	0.134	0.000	0.228	.	-0.051	0.901	0.249	0.005	0.001	0.557	0.333	0.017	0.042	6.151	0.954	0.304	0.018	0.044	6.371	-0.547	0.010	2	.	.
9822	2	2	4	10	39	3	3	3	5	.	.	.	.	.	.	.	.	.	.	.	.	.	.	.	.	.	.	.	.	.	.	.	.	.	.	.
9822	2	2	4	11	40	3	3	3	5	-0.033	0.249	0.456	0.141	0.000	0.249	.	-0.141	0.926	0.274	0.005	0.001	0.557	0.333	0.017	0.042	6.151	0.954	0.304	0.018	0.044	6.371	-0.547	0.010	2	.	.
1391	1	1	1	2	1	1	1	1	1	.	.	.	.	.	.	.	.	.	.	.	.	.	.	.	.	.	.	.	.	.	.	.	.	.	.	.
1391	1	1	1	2	2	1	2	1	2	.	.	.	.	.	.	.	.	.	.	.	.	.	.	.	.	.	.	.	.	.	.	.	.	.	.	.
1391	1	1	1	2	3	1	1	2	3	.	.	.	.	.	.	.	.	.	.	.	.	.	.	.	.	.	.	.	.	.	.	.	.	.	.	.
1391	1	1	1	2	4	1	2	2	4	.	.	.	.	.	.	.	.	.	.	.	.	.	.	.	.	.	.	.	.	.	.	.	.	.	.	.
1391	1	1	1	3	5	1	1	1	1	0.067	0.120	0.185	0.021	0.015	0.135	.	0.108	0.165	0.731	0.023	0.004	0.594	0.150	0.020	0.529	29.182	1.170	0.158	0.021	0.534	29.312	-0.044	0.549	1	.	.
1391	1	1	1	3	6	1	2	1	2	0.134	0.117	0.119	0.013	0.080	0.197	.	0.180	0.161	0.731	0.023	0.004	0.594	0.150	0.020	0.529	29.182	1.170	0.158	0.021	0.534	29.312	-0.044	0.549	1	.	.
1391	1	1	1	3	7	1	1	2	3	0.468	0.136	0.094	0.013	0.026	0.161	.	0.639	0.184	0.731	0.023	0.004	0.594	0.150	0.020	0.529	29.182	1.170	0.158	0.021	0.534	29.312	-0.044	0.549	1	.	.
1391	1	1	1	3	8	1	2	2	4	0.567	0.117	0.065	0.008	0.004	0.121	.	0.784	0.163	0.731	0.023	0.004	0.594	0.150	0.020	0.529	29.182	1.170	0.158	0.021	0.534	29.312	-0.044	0.549	1	.	.
1391	1	1	1	4	9	1	1	1	1	0.045	0.124	0.207	0.027	0.014	0.138	.	0.056	0.170	0.731	0.020	0.003	0.594	0.150	0.020	0.529	29.182	1.170	0.158	0.021	0.534	29.312	-0.044	0.549	1	.	.
1391	1	1	1	4	10	1	2	1	2	0.134	0.117	0.119	0.013	0.080	0.197	.	0.180	0.161	0.731	0.023	0.004	0.594	0.150	0.020	0.529	29.182	1.170	0.158	0.021	0.534	29.312	-0.044	0.549	1	.	.

1391	1	1	1	4	11	1	1	2	3	0.437	0.115	0.305	0.037	0.013	0.128	.	0.583	0.159	0.731	0.020	0.003	0.594	0.150	0.020	0.529	29.182	1.170	0.158	0.021	0.534	29.312	-0.044	0.549	1	.	.
1391	1	1	1	4	12	1	2	2	4	0.518	0.132	0.094	0.012	0.002	0.134	.	0.706	0.180	0.731	0.020	0.003	0.594	0.150	0.020	0.529	29.182	1.170	0.158	0.021	0.534	29.312	-0.044	0.549	1	.	.
1391	1	1	1	6	13	1	1	1	1	0.049	0.132	0.483	0.064	0.022	0.154	.	0.080	0.182	0.731	0.001	0.001	0.594	0.150	0.020	0.529	29.182	1.170	0.158	0.021	0.534	29.312	-0.044	0.549	1	.	.
1391	1	1	1	6	14	1	2	1	2	0.102	0.135	0.271	0.038	0.039	0.174	.	0.133	0.187	0.731	0.001	0.001	0.594	0.150	0.020	0.529	29.182	1.170	0.158	0.021	0.534	29.312	-0.044	0.549	1	.	.
1391	1	1	1	6	15	1	1	2	3	0.499	0.106	0.308	0.032	0.009	0.115	.	0.679	0.144	0.731	0.001	0.001	0.594	0.150	0.020	0.529	29.182	1.170	0.158	0.021	0.534	29.312	-0.044	0.549	1	.	.
1391	1	1	1	6	16	1	2	2	4	0.448	0.122	0.167	0.020	0.009	0.131	.	0.626	0.168	0.731	0.001	0.001	0.594	0.150	0.020	0.529	29.182	1.170	0.158	0.021	0.534	29.312	-0.044	0.549	1	.	.
1391	1	1	1	7	17	1	1	1	1	-0.030	0.214	0.023	0.010	0.035	0.249	.	-0.039	0.299	0.731	0.001	0.001	0.594	0.150	0.020	0.529	29.182	1.170	0.158	0.021	0.534	29.312	-0.044	0.549	1	.	.
1391	1	1	1	7	18	1	2	1	2	0.234	0.112	0.010	0.010	0.000	0.112	.	0.343	0.160	0.731	0.001	0.001	0.594	0.150	0.020	0.529	29.182	1.170	0.158	0.021	0.534	29.312	-0.044	0.549	1	.	.
1391	1	1	1	7	19	1	1	2	3	.	.	.	.	.	.	.	.	.	.	.	.	.	.	.	.	.	.	.	.	.	.	.	.	.	.	.
1391	1	1	1	7	20	1	2	2	4	.	.	.	.	.	.	.	.	.	.	.	.	.	.	.	.	.	.	.	.	.	.	.	.	.	.	.
1391	1	1	1	8	21	1	1	1	1	-0.039	0.138	0.098	0.013	0.024	0.162	.	-0.056	0.188	0.731	0.018	0.003	0.594	0.150	0.020	0.529	29.182	1.170	0.158	0.021	0.534	29.312	-0.044	0.549	1	.	.
1391	1	1	1	8	22	1	2	1	2	0.022	0.128	0.084	0.009	0.061	0.189	.	0.007	0.177	0.731	0.018	0.003	0.594	0.150	0.020	0.529	29.182	1.170	0.158	0.021	0.534	29.312	-0.044	0.549	1	.	.
1391	1	1	1	8	23	1	1	2	3	0.125	0.119	0.154	0.018	0.000	0.119	.	0.175	0.164	0.731	0.018	0.003	0.594	0.150	0.020	0.529	29.182	1.170	0.158	0.021	0.534	29.312	-0.044	0.549	1	.	.
1391	1	1	1	8	24	1	2	2	4	0.317	0.126	0.100	0.012	0.011	0.137	.	0.417	0.173	0.731	0.018	0.003	0.594	0.150	0.020	0.529	29.182	1.170	0.158	0.021	0.534	29.312	-0.044	0.549	1	.	.
1391	1	1	1	10	25	1	1	1	1	.	.	.	.	.	.	.	.	.	.	.	.	.	.	.	.	.	.	.	.	.	.	.	.	.	.	.
1391	1	1	1	10	26	1	2	1	2	.	.	.	.	.	.	.	.	.	.	.	.	.	.	.	.	.	.	.	.	.	.	.	.	.	.	.
1391	1	1	1	10	27	1	1	2	3	0.438	0.154	0.042	0.005	0.022	0.176	.	0.598	0.215	0.731	0.001	0.001	0.594	0.150	0.020	0.529	29.182	1.170	0.158	0.021	0.534	29.312	-0.044	0.549	1	.	.
1391	1	1	1	10	28	1	2	2	4	0.245	0.150	0.012	0.001	0.172	0.322	.	0.310	0.190	0.791	0.001	0.001	0.594	0.150	0.020	0.529	29.182	1.170	0.158	0.021	0.534	29.312	-0.044	0.549	1	.	.
1391	1	1	1	11	29	1	1	1	1	0.000	0.134	0.053	0.010	0.037	0.171	.	0.001	0.185	0.731	0.001	0.001	0.594	0.150	0.020	0.529	29.182	1.170	0.158	0.021	0.534	29.312	-0.044	0.549	1	.	.
1391	1	1	1	11	30	1	2	1	2	0.050	0.149	0.023	0.010	0.021	0.170	.	0.077	0.205	0.731	0.001	0.001	0.594	0.150	0.020	0.529	29.182	1.170	0.158	0.021	0.534	29.312	-0.044	0.549	1	.	.
1391	1	1	1	11	31	1	1	2	3	0.353	0.150	0.037	0.010	0.011	0.161	.	0.496	0.205	0.731	0.001	0.001	0.594	0.150	0.020	0.529	29.182	1.170	0.158	0.021	0.534	29.312	-0.044	0.549	1	.	.
1391	1	1	1	11	32	1	2	2	4	0.485	0.120	0.020	0.010	0.006	0.125	.	0.666	0.168	0.716	0.001	0.001	0.594	0.150	0.020	0.529	29.182	1.170	0.158	0.021	0.534	29.312	-0.044	0.549	1	.	.
1391	1	1	1	2	33	3	3	3	5	-0.025	0.220	0.058	0.005	0.000	0.220	.	-0.104	1.000	0.220	0.001	0.001	0.613	0.162	0.020	0.540	29.572	1.156	0.175	0.021	0.529	29.707	0.017	0.629	1	.	.
1391	1	1	1	3	34	3	3	3	5	0.000	0.176	0.107	0.019	0.000	0.176	0.502	0.001	0.818	0.220	0.023	0.004	0.613	0.162	0.020	0.540	29.572	1.156	0.175	0.021	0.529	29.707	0.017	0.629	1	.	.
1391	1	1	1	4	35	3	3	3	5	0.011	0.151	0.327	0.046	0.000	0.151	.	0.048	0.705	0.220	0.020	0.003	0.613	0.162	0.020	0.540	29.572	1.156	0.175	0.021	0.529	29.707	0.017	0.629	1	.	.
1391	1	1	1	6	36	3	3	3	5	0.000	0.140	0.220	0.031	0.000	0.140	.	0.003	0.645	0.220	0.001	0.001	0.613	0.162	0.020	0.540	29.572	1.156	0.175	0.021	0.529	29.707	0.017	0.629	1	.	.
1391	1	1	1	7	37	3	3	3	5	.	.	.	.	.	.	.	.	.	.	.	.	.	.	.	.	.	.	.	.	.	.	.	.	.	.	.
1391	1	1	1	8	38	3	3	3	5	0.070	0.136	0.206	0.028	0.000	0.136	.	0.275	0.617	0.229	0.015	0.017	0.585	0.136	0.011	0.265	15.283	1.142	0.147	0.011	0.259	15.399	-0.565	0.019	2	.	.
1391	1	1	1	10	39	3	3	3	5	.	.	.	.	.	.	.	.	.	.	.	.	.	.	.	.	.	.	.	.	.	.	.	.	.	.	.
1391	1	1	1	11	40	3	3	3	5	0.095	0.181	0.025	0.010	0.000	0.181	.	0.446	0.806	0.220	0.001	0.001	0.613	0.162	0.020	0.540	29.572	1.156	0.175	0.021	0.529	29.707	0.017	0.629	1	.	.
1391	1	1	2	2	1	1	1	1	1	.	.	.	.	.	.	.	.	.	.	.	.	.	.	.	.	.	.	.	.	.	.	.	.	.	.	.
1391	1	1	2	2	2	1	2	1	2	.	.	.	.	.	.	.	.	.	.	.	.	.	.	.	.	.	.	.	.	.	.	.	.	.	.	.
1391	1	1	2	2	3	1	1	2	3	.	.	.	.	.	.	.	.	.	.	.	.	.	.	.	.	.	.	.	.	.	.	.	.	.	.	.
1391	1	1	2	2	4	1	2	2	4	.	.	.	.	.	.	.	.	.	.	.	.	.	.	.	.	.	.	.	.	.	.	.	.	.	.	.
1391	1	1	2	3	5	1	1	1	1	-0.074	0.119	0.394	0.042	0.018	0.137	.	-0.141	0.200	0.596	0.015	0.003	0.548	0.196	0.002	0.005	0.258	1.067	0.129	0.001	0.007	0.345	-0.706	-0.159	2	.	.
1391	1	1	2	3	6	1	2	1	2	-0.045	0.132	0.357	0.061	0.021	0.153	.	-0.127	0.233	0.596	0.015	0.003	0.548	0.196	0.002	0.005	0.258	1.067	0.129	0.001	0.007	0.345	-0.706	-0.159	2	.	.
1391	1	1	2	3	7	1	1	2	3	0.266	0.128	0.420	0.059	0.004	0.132	.	0.403	0.224	0.596	0.001	0.001	0.548	0.196	0.002	0.005	0.258	1.067	0.129	0.001	0.007	0.345	-0.706	-0.159	2	.	.
1391	1	1	2	3	8	1	2	2	4	0.217	0.113	0.312	0.034	0.016	0.129	.	0.354	0.186	0.596	0.006	0.002	0.548	0.196	0.002	0.005	0.258	1.067	0.129	0.001	0.007	0.345	-0.706	-0.159	2	.	.
1391	1	1	2	4	9	1	1	1	1	0.176	0.131	0.080	0.010	0.024	0.155	.	0.273	0.236	0.568	0.001	0.001	0.548	0.196	0.002	0.005	0.258	1.067	0.129	0.001	0.007	0.345	-0.706	-0.159	2	.	.
1391	1	1	2	4	10	1	2	1	2	0.014	0.136	0.055	0.010	0.034	0.170	.	-0.013	0.243	0.568	0.001	0.001	0.548	0.196	0.002	0.005	0.258	1.067	0.129	0.001	0.007	0.345	-0.706	-0.159	2	.	.
1391	1	1	2	4	11	1	1	2	3	0.376	0.154	0.031	0.008	0.037	0.190	.	0.636	0.284	0.568	0.001	0.001	0.548	0.196	0.002	0.005	0.258	1.067	0.129	0.001	0.007	0.345	-0.706	-0.159	2	.	.
1391	1	1	2	4	12	1	2	2	4	0.579	0.128	0.030	0.010	0.000	0.128	.	1.013	0.231	0.568	0.001	0.001	0.548	0.196	0.002	0.005	0.258	1.067	0.129	0.001	0.007	0.345	-0.706	-0.159	2	.	.
1391	1	1	2	6	13	1	1	1	1	0.067	0.144	0.053	0.006	0.037	0.181	.	0.087	0.257	0.568	0.001	0.001	0.548	0.196	0.002	0.005	0.258	1.067	0.129	0.001	0.007	0.345	-0.706	-0.159	2	.	.
1391	1	1	2	6	14	1	2	1	2	0.034	0.156	0.038	0.006	0.027	0.183	.	0.073	0.276	0.568	0.001	0.001	0.548	0.196	0.002	0.005	0.258	1.067	0.129	0.001	0.007	0.345	-0.706	-0.159	2	.	.
1391	1	1	2	6	15	1	1	2	3	0.426	0.125	0.056	0.006	0.011	0.136	.	0.753	0.220	0.568	0.001	0.001	0.548	0.196	0.002	0.005	0.258	1.067	0.129	0.001	0.007	0.345	-0.706	-0.159	2	.	.
1391	1	1	2	6	16	1	2	2	4	0.463	0.112	0.033	0.006	0.008	0.120	.	0.787	0.196	0.568	0.001	0.001	0.548	0.196	0.002	0.005	0.258	1.067	0.129	0.001	0.007	0.345	-0.706	-0.159	2	.	.
1391	1	1	2	7	17	1	1	1	1	0.048	0.345	0.410	0.060	0.041	0.386	.	0.106	0.561	0.596	0.001	0.001	0.548	0.196	0.002	0.005	0.258	1.067	0.129	0.001	0.007	0.345	-0.706	-0.159	2	.	.
1391	1	1	2	7	18	1	2	1	2	0.075	0.178	0.233	0.041	0.017	0.195	.	0.172	0.294	0.596	0.001	0.001	0.548	0.196	0.002	0.005	0.258	1.067	0.129	0.001	0.007	0.345	-0.706	-0.159	2	.	.
1391	1	1	2	7	19	1	1	2	3	.	.	.	.	.	.	.	.	.	.	.	.	.	.	.	.	.	.	.	.	.	.	.	.	.	.	.
1391	1	1	2	7	20	1	2	2	4	.	.	.	.	.	.	.	.	.	.	.	.	.	.	.	.	.	.	.	.	.	.	.	.	.	.	.
1391	1	1	2	8	21	1	1	1	1	0.247	0.104	0.513	0.053	0.026	0.130	.	0.402	0.177	0.596	0.012	0.030	0.548	0.196	0.002	0.005	0.258	1.067	0.129	0.001	0.007	0.345	-0.706	-0.159	2	.	.
1391	1	1	2	8	22	1	2	1	2	0.028	0.122	0.341	0.043	0.015	0.137	.	0.008	0.211	0.596	0.012	0.030	0.548	0.196	0.002	0.005	0.258	1.067	0.129	0.001	0.007	0.345	-0.706	-0.159	2	.	.
1391	1	1	2	8	23	1	1	2	3	0.561	0.154	0.463	0.076	0.011	0.165	.	0.932	0.255	0.596	0.012	0.030	0.548	0.196	0.002	0.005	0.258	1.067	0.129	0.001	0.007	0.345	-0.706	-0.159	2	.	.
1391	1	1	2	8	24	1	2	2	4	0.492	0.113	0.227	0.028	0.033	0.146	.	0.846	0.190	0.596	0.012	0.030	0.548	0.196	0.002	0.005	0.258	1.067	0.129	0.001	0.007	0.345	-0.706	-0.159	2	.	.
1391	1	1	2	10	25	1	1	1	1	.	.	.	.	.	.	.	.	.	.	.	.	.	.	.	.	.	.	.	.	.	.	.	.	.	.	.
1391	1	1	2	10	26	1	2	1	2	.	.	.	.	.	.	.	.	.	.	.	.	.	.	.	.	.	.	.	.	.	.	.	.	.	.	.
1391	1	1	2	10	27	1	1	2	3	0.351	0.150	0.114	0.015	0.018	0.168	.	0.604	0.254	0.596	0.003	0.001	0.548	0.196	0.002	0.005	0.258	1.067	0.129	0.001	0.007	0.345	-0.706	-0.159	2	.	.
1391	1	1	2	10	28	1	2	2	4	0.426	0.165	0.070	0.012	0.011	0.176	.	0.694	0.280	0.596	0.003	0.001	0.548	0.196	0.002	0.005	0.258	1.067	0.129	0.001	0.007	0.345	-0.706	-0.159	2	.	.
1391	1	1	2	11	29	1	1	1	1	0.000	0.156	0.018	0.002	0.050	0.205	.	0.001	0.268	0.596	0.001	0.001	0.548	0.196	0.002	0.005	0.258	1.067	0.129	0.001	0.007	0.345	-0.706	-0.159	2	.	.
1391	1	1	2	11	30	1	2	1	2	0.189	0.123	0.013	0.001	0.064	0.187	.	0.293	0.190	0.596	0.001	0.001	0.548	0.196	0.002	0.005	0.258	1.067	0.129	0.001	0.007	0.345	-0.706	-0.159	2	.	.
1391	1	1	2	11	31	1	1	2	3	0.412	0.117	0.127	0.014	0.009	0.126	.	0.681	0.203	0.596	0.001	0.001	0.548	0.196	0.002	0.005	0.258	1.067	0.129	0.001	0.007	0.345	-0.706	-0.159	2	.	.
1391	1	1	2	11	32	1	2	2	4	0.449	0.145	0.153	0.020	0.011	0.156	.	0.695	0.225	0.644	0.001	0.001	0.548	0.196	0.002	0.005	0.258	1.067	0.129	0.001	0.007	0.345	-0.706	-0.159	2	.	.
1391	1	1	2	2	33	3	3	3	5	0.011	0.230	0.010	0.010	0.000	0.230	.	0.036	1.000	0.230	0.001	0.001	0.589	0.184	0.002	0.004	0.347	1.077	0.133	0.002	0.001	0.473	-1.100	-0.511	2	.	.
1391	1	1	2	3	34	3	3	3	5	0.000	0.202	0.862	0.175	0.000	0.202	1.335	0.001	0.887	0.230	0.015	0.003	0.589	0.184	0.002	0.004	0.347	1.077	0.133	0.002	0.001	0.473	-1.100	-0.511	2	.	0.248
1391	1	1	2	4	35	3	3	3	5	0.018	0.152	0.047	0.010	0.000	0.152	.	0.078	0.661	0.230	0.001	0.001	0.589	0.184	0.002	0.004	0.347	1.077	0.133	0.002	0.001	0.473	-1.100	-0.511	2	.	.
1391	1	1	2	6	36	3	3	3	5	0.046	0.145	0.060	0.007	0.000	0.145	.	0.193	0.641	0.230	0.001	0.001	0.589	0.184	0.002	0.004	0.347	1.077	0.133	0.002	0.001	0.473	-1.100	-0.511	2	.	.
1391	1	1	2	7	37	3	3	3	5	.	.	.	.	.	.	.	.	.	.	.	.	.	.	.	.	.	.	.	.	.	.	.	.	.	.	.
1391	1	1	2	8	38	3	3	3	5	0.089	0.152	0.641	0.106	0.000	0.152	.	0.373	0.674	0.230	0.012	0.030	0.589	0.184	0.002	0.004	0.347	1.077	0.133	0.002	0.001	0.473	-1.100	-0.511	2	.	.
1391	1	1	2	10	39	3	3	3	5	.	.	.	.	.	.	.	.	.	.	.	.	.	.	.	.	.	.	.	.	.	.	.	.	.	.	.
1391	1	1	2	11	40	3	3	3	5	0.056	0.173	0.060	0.007	0.000	0.173	.	0.240	0.759	0.230	0.001	0.001	0.589	0.184	0.002	0.004	0.347	1.077	0.133	0.002	0.001	0.473	-1.100	-0.511	2	.	.
1391	1	1	3	2	1	1	1	1	1	.	.	.	.	.	.	.	.	.	.	.	.	.	.	.	.	.	.	.	.	.	.	.	.	.	.	.
1391	1	1	3	2	2	1	2	1	2	.	.	.	.	.	.	.	.	.	.	.	.	.	.	.	.	.	.	.	.	.	.	.	.	.	.	.
1391	1	1	3	2	3	1	1	2	3	.	.	.	.	.	.	.	.	.	.	.	.	.	.	.	.	.	.	.	.	.	.	.	.	.	.	.
1391	1	1	3	2	4	1	2	2	4	.	.	.	.	.	.	.	.	.	.	.	.	.	.	.	.	.	.	.	.	.	.	.	.	.	.	.
1391	1	1	3	3	5	1	1	1	1	.	.	.	.	.	.	.	.	.	.	.	.	.	.	.	.	.	.	.	.	.	.	.	.	.	.	.
1391	1	1	3	3	6	1	2	1	2	.	.	.	.	.	.	.	.	.	.	.	.	.	.	.	.	.	.	.	.	.	.	.	.	.	.	.
1391	1	1	3	3	7	1	1	2	3	.	.	.	.	.	.	.	.	.	.	.	.	.	.	.	.	.	.	.	.	.	.	.	.	.	.	.
1391	1	1	3	3	8	1	2	2	4	.	.	.	.	.	.	.	.	.	.	.	.	.	.	.	.	.	.	.	.	.	.	.	.	.	.	.
1391	1	1	3	4	9	1	1	1	1	0.062	0.112	0.090	0.010	0.019	0.131	.	0.092	0.215	0.524	0.001	0.001	0.543	0.264	0.007	0.050	1.292	0.972	0.359	0.006	0.071	1.448	-0.272	0.271	1	.	.
1391	1	1	3	4	10	1	2	1	2	0.095	0.131	0.060	0.010	0.028	0.158	.	0.182	0.251	0.524	0.001	0.001	0.543	0.264	0.007	0.050	1.292	0.972	0.359	0.006	0.071	1.448	-0.272	0.271	1	.	.
1391	1	1	3	4	11	1	1	2	3	0.371	0.145	0.070	0.010	0.006	0.150	.	0.696	0.281	0.524	0.001	0.001	0.543	0.264	0.007	0.050	1.292	0.972	0.359	0.006	0.071	1.448	-0.272	0.271	1	.	.
1391	1	1	3	4	12	1	2	2	4	0.404	0.114	0.050	0.010	0.008	0.122	.	0.782	0.221	0.524	0.001	0.001	0.543	0.264	0.007	0.050	1.292	0.972	0.359	0.006	0.071	1.448	-0.272	0.271	1	.	.
1391	1	1	3	6	13	1	1	1	1	0.053	0.111	0.290	0.032	0.081	0.192	.	0.090	0.213	0.524	0.001	0.001	0.543	0.264	0.007	0.050	1.292	0.972	0.359	0.006	0.071	1.448	-0.272	0.271	1	.	.
1391	1	1	3	6	14	1	2	1	2	-0.081	0.114	0.227	0.026	0.011	0.124	.	-0.168	0.219	0.524	0.001	0.001	0.543	0.264	0.007	0.050	1.292	0.972	0.359	0.006	0.071	1.448	-0.272	0.271	1	.	.
1391	1	1	3	6	15	1	1	2	3	0.510	0.098	0.392	0.038	0.006	0.103	.	0.940	0.188	0.524	0.001	0.001	0.543	0.264	0.007	0.050	1.292	0.972	0.359	0.006	0.071	1.448	-0.272	0.271	1	.	.
1391	1	1	3	6	16	1	2	2	4	0.462	0.123	0.265	0.031	0.003	0.126	.	0.856	0.239	0.524	0.001	0.001	0.543	0.264	0.007	0.050	1.292	0.972	0.359	0.006	0.071	1.448	-0.272	0.271	1	.	.
1391	1	1	3	7	17	1	1	1	1	0.097	0.131	0.096	0.013	0.023	0.154	.	0.181	0.234	0.568	0.001	0.001	0.543	0.264	0.007	0.050	1.292	0.972	0.359	0.006	0.071	1.448	-0.272	0.271	1	.	.
1391	1	1	3	7	18	1	2	1	2	0.279	0.132	0.064	0.011	0.017	0.149	.	0.507	0.234	0.564	0.001	0.001	0.543	0.264	0.007	0.050	1.292	0.972	0.359	0.006	0.071	1.448	-0.272	0.271	1	.	.
1391	1	1	3	7	19	1	1	2	3	.	.	.	.	.	.	.	.	.	.	.	.	.	.	.	.	.	.	.	.	.	.	.	.	.	.	.
1391	1	1	3	7	20	1	2	2	4	.	.	.	.	.	.	.	.	.	.	.	.	.	.	.	.	.	.	.	.	.	.	.	.	.	.	.
1391	1	1	3	8	21	1	1	1	1	0.062	0.133	0.313	0.044	0.013	0.146	.	0.104	0.235	0.568	0.010	0.002	0.543	0.264	0.007	0.050	1.292	0.972	0.359	0.006	0.071	1.448	-0.272	0.271	1	.	.
1391	1	1	3	8	22	1	2	1	2	0.151	0.127	0.165	0.020	0.016	0.143	.	0.266	0.223	0.568	0.008	0.002	0.543	0.264	0.007	0.050	1.292	0.972	0.359	0.006	0.071	1.448	-0.272	0.271	1	.	.
1391	1	1	3	8	23	1	1	2	3	0.457	0.095	0.343	0.032	0.000	0.095	.	0.809	0.170	0.568	0.010	0.002	0.543	0.264	0.007	0.050	1.292	0.972	0.359	0.006	0.071	1.448	-0.272	0.271	1	.	.
1391	1	1	3	8	24	1	2	2	4	0.312	0.110	0.255	0.027	0.014	0.124	.	0.552	0.195	0.568	0.010	0.002	0.543	0.264	0.007	0.050	1.292	0.972	0.359	0.006	0.071	1.448	-0.272	0.271	1	.	.
1391	1	1	3	10	25	1	1	1	1	.	.	.	.	.	.	.	.	.	.	.	.	.	.	.	.	.	.	.	.	.	.	.	.	.	.	.
1391	1	1	3	10	26	1	2	1	2	.	.	.	.	.	.	.	.	.	.	.	.	.	.	.	.	.	.	.	.	.	.	.	.	.	.	.
1391	1	1	3	10	27	1	1	2	3	0.366	0.123	0.040	0.005	0.005	0.128	.	0.637	0.218	0.570	0.001	0.001	0.543	0.264	0.007	0.050	1.292	0.972	0.359	0.006	0.071	1.448	-0.272	0.271	1	.	.
1391	1	1	3	10	28	1	2	2	4	0.346	0.132	0.021	0.003	0.008	0.140	.	0.581	0.234	0.570	0.001	0.001	0.543	0.264	0.007	0.050	1.292	0.972	0.359	0.006	0.071	1.448	-0.272	0.271	1	.	.
1391	1	1	3	11	29	1	1	1	1	0.000	0.142	0.667	0.094	0.015	0.157	.	0.001	0.250	0.568	0.028	0.003	0.543	0.264	0.007	0.050	1.292	0.972	0.359	0.006	0.071	1.448	-0.272	0.271	1	.	.
1391	1	1	3	11	30	1	2	1	2	0.108	0.107	0.566	0.060	0.017	0.124	.	0.188	0.189	0.568	0.028	0.003	0.543	0.264	0.007	0.050	1.292	0.972	0.359	0.006	0.071	1.448	-0.272	0.271	1	.	.
1391	1	1	3	11	31	1	1	2	3	0.354	0.117	0.779	0.090	0.008	0.125	.	0.611	0.209	0.568	0.028	0.003	0.543	0.264	0.007	0.050	1.292	0.972	0.359	0.006	0.071	1.448	-0.272	0.271	1	.	.
1391	1	1	3	11	32	1	2	2	4	0.362	0.126	0.631	0.081	0.006	0.131	.	0.645	0.223	0.568	0.028	0.003	0.543	0.264	0.007	0.050	1.292	0.972	0.359	0.006	0.071	1.448	-0.272	0.271	1	.	.
1391	1	1	3	2	33	3	3	3	5	0.000	0.208	0.059	0.020	0.000	0.208	.	0.001	1.000	0.208	0.001	0.001	0.585	0.314	0.007	0.058	3.170	1.030	0.283	0.008	0.031	3.366	-0.236	0.349	1	.	.
1391	1	1	3	3	34	3	3	3	5	0.000	0.208	0.079	0.020	0.000	0.208	0.755	0.001	1.000	0.208	0.001	0.001	0.585	0.314	0.007	0.058	3.170	1.030	0.283	0.008	0.031	3.366	-0.236	0.349	1	0.624	0.362
1391	1	1	3	4	35	3	3	3	5	0.024	0.173	0.082	0.014	0.000	0.173	.	0.117	0.869	0.208	0.001	0.001	0.585	0.314	0.007	0.058	3.170	1.030	0.283	0.008	0.031	3.366	-0.236	0.349	1	.	.
1391	1	1	3	6	36	3	3	3	5	0.110	0.133	0.025	0.002	0.000	0.133	.	0.525	0.646	0.208	0.001	0.001	0.585	0.314	0.007	0.058	3.170	1.030	0.283	0.008	0.031	3.366	-0.236	0.349	1	.	.
1391	1	1	3	7	37	3	3	3	5	.	.	.	.	.	.	.	.	.	.	.	.	.	.	.	.	.	.	.	.	.	.	.	.	.	.	.
1391	1	1	3	8	38	3	3	3	5	-0.014	0.182	0.454	0.076	0.000	0.182	.	-0.083	0.938	0.208	0.010	0.002	0.585	0.314	0.007	0.058	3.170	1.030	0.283	0.008	0.031	3.366	-0.236	0.349	1	.	.
1391	1	1	3	10	39	3	3	3	5	.	.	.	.	.	.	.	.	.	.	.	.	.	.	.	.	.	.	.	.	.	.	.	.	.	.	.
1391	1	1	3	11	40	3	3	3	5	-0.052	0.145	0.685	0.100	0.000	0.145	.	-0.218	0.727	0.208	0.028	0.003	0.585	0.314	0.007	0.058	3.170	1.030	0.283	0.008	0.031	3.366	-0.236	0.349	1	.	.
1391	1	1	4	2	1	1	1	1	1	.	.	.	.	.	.	.	.	.	.	.	.	.	.	.	.	.	.	.	.	.	.	.	.	.	.	.
1391	1	1	4	2	2	1	2	1	2	.	.	.	.	.	.	.	.	.	.	.	.	.	.	.	.	.	.	.	.	.	.	.	.	.	.	.
1391	1	1	4	2	3	1	1	2	3	.	.	.	.	.	.	.	.	.	.	.	.	.	.	.	.	.	.	.	.	.	.	.	.	.	.	.
1391	1	1	4	2	4	1	2	2	4	.	.	.	.	.	.	.	.	.	.	.	.	.	.	.	.	.	.	.	.	.	.	.	.	.	.	.
1391	1	1	4	3	5	1	1	1	1	0.105	0.139	0.134	0.018	0.020	0.159	.	0.162	0.205	0.678	0.020	0.002	0.568	0.365	0.020	0.806	5.950	1.097	0.367	0.020	0.800	6.223	-0.655	-0.087	1	.	.
1391	1	1	4	3	6	1	2	1	2	0.189	0.112	0.088	0.009	0.017	0.128	.	0.277	0.164	0.678	0.020	0.002	0.568	0.365	0.020	0.806	5.950	1.097	0.367	0.020	0.800	6.223	-0.655	-0.087	1	.	.
1391	1	1	4	3	7	1	1	2	3	0.426	0.120	0.076	0.009	0.006	0.127	.	0.623	0.178	0.678	0.020	0.002	0.568	0.365	0.020	0.806	5.950	1.097	0.367	0.020	0.800	6.223	-0.655	-0.087	1	.	.
1391	1	1	4	3	8	1	2	2	4	0.502	0.117	0.053	0.006	0.002	0.119	.	0.746	0.172	0.678	0.020	0.002	0.568	0.365	0.020	0.806	5.950	1.097	0.367	0.020	0.800	6.223	-0.655	-0.087	1	.	.
1391	1	1	4	4	9	1	1	1	1	0.119	0.124	0.411	0.050	0.034	0.158	.	0.184	0.182	0.678	0.003	0.001	0.568	0.365	0.020	0.806	5.950	1.097	0.367	0.020	0.800	6.223	-0.655	-0.087	1	.	.
1391	1	1	4	4	10	1	2	1	2	0.113	0.127	0.243	0.030	0.024	0.151	.	0.170	0.187	0.678	0.003	0.001	0.568	0.365	0.020	0.806	5.950	1.097	0.367	0.020	0.800	6.223	-0.655	-0.087	1	.	.
1391	1	1	4	4	11	1	1	2	3	0.379	0.154	0.366	0.058	0.015	0.169	.	0.560	0.226	0.678	0.003	0.001	0.568	0.365	0.020	0.806	5.950	1.097	0.367	0.020	0.800	6.223	-0.655	-0.087	1	.	.
1391	1	1	4	4	12	1	2	2	4	0.487	0.124	0.173	0.020	0.002	0.127	.	0.730	0.184	0.678	0.003	0.001	0.568	0.365	0.020	0.806	5.950	1.097	0.367	0.020	0.800	6.223	-0.655	-0.087	1	.	.
1391	1	1	4	6	13	1	1	1	1	0.056	0.137	0.509	0.072	0.035	0.172	.	0.096	0.202	0.678	0.005	0.001	0.568	0.365	0.020	0.806	5.950	1.097	0.367	0.020	0.800	6.223	-0.655	-0.087	1	.	.
1391	1	1	4	6	14	1	2	1	2	0.113	0.131	0.292	0.038	0.030	0.161	.	0.169	0.193	0.678	0.005	0.001	0.568	0.365	0.020	0.806	5.950	1.097	0.367	0.020	0.800	6.223	-0.655	-0.087	1	.	.
1391	1	1	4	6	15	1	1	2	3	0.455	0.127	0.356	0.046	0.005	0.132	.	0.674	0.187	0.678	0.005	0.001	0.568	0.365	0.020	0.806	5.950	1.097	0.367	0.020	0.800	6.223	-0.655	-0.087	1	.	.
1391	1	1	4	6	16	1	2	2	4	0.407	0.133	0.239	0.033	0.008	0.140	.	0.612	0.195	0.678	0.005	0.001	0.568	0.365	0.020	0.806	5.950	1.097	0.367	0.020	0.800	6.223	-0.655	-0.087	1	.	.
1391	1	1	4	7	17	1	1	1	1	0.083	0.157	0.123	0.021	0.023	0.180	.	0.127	0.240	0.678	0.024	0.002	0.568	0.365	0.020	0.806	5.950	1.097	0.367	0.020	0.800	6.223	-0.655	-0.087	1	.	.
1391	1	1	4	7	18	1	2	1	2	0.194	0.127	0.081	0.010	0.023	0.150	.	0.296	0.193	0.678	0.024	0.002	0.568	0.365	0.020	0.806	5.950	1.097	0.367	0.020	0.800	6.223	-0.655	-0.087	1	.	.
1391	1	1	4	7	19	1	1	2	3	.	.	.	.	.	.	.	.	.	.	.	.	.	.	.	.	.	.	.	.	.	.	.	.	.	.	.
1391	1	1	4	7	20	1	2	2	4	.	.	.	.	.	.	.	.	.	.	.	.	.	.	.	.	.	.	.	.	.	.	.	.	.	.	.
1391	1	1	4	8	21	1	1	1	1	0.219	0.120	0.163	0.019	0.030	0.150	.	0.346	0.178	0.683	0.001	0.001	0.568	0.365	0.020	0.806	5.950	1.097	0.367	0.020	0.800	6.223	-0.655	-0.087	1	.	.
1391	1	1	4	8	22	1	2	1	2	0.217	0.122	0.115	0.014	0.013	0.135	.	0.355	0.180	0.683	0.001	0.001	0.568	0.365	0.020	0.806	5.950	1.097	0.367	0.020	0.800	6.223	-0.655	-0.087	1	.	.
1391	1	1	4	8	23	1	1	2	3	0.520	0.128	0.132	0.016	0.005	0.134	.	0.797	0.187	0.683	0.001	0.001	0.568	0.365	0.020	0.806	5.950	1.097	0.367	0.020	0.800	6.223	-0.655	-0.087	1	.	.
1391	1	1	4	8	24	1	2	2	4	0.492	0.110	0.097	0.010	0.013	0.123	.	0.738	0.163	0.683	0.001	0.001	0.568	0.365	0.020	0.806	5.950	1.097	0.367	0.020	0.800	6.223	-0.655	-0.087	1	.	.
1391	1	1	4	10	25	1	1	1	1	.	.	.	.	.	.	.	.	.	.	.	.	.	.	.	.	.	.	.	.	.	.	.	.	.	.	.
1391	1	1	4	10	26	1	2	1	2	.	.	.	.	.	.	.	.	.	.	.	.	.	.	.	.	.	.	.	.	.	.	.	.	.	.	.
1391	1	1	4	10	27	1	1	2	3	0.288	0.151	0.075	0.010	0.021	0.172	.	0.441	0.231	0.678	0.002	0.001	0.568	0.365	0.020	0.806	5.950	1.097	0.367	0.020	0.800	6.223	-0.655	-0.087	1	.	.
1391	1	1	4	10	28	1	2	2	4	0.492	0.110	0.097	0.010	0.013	0.123	.	0.738	0.163	0.683	0.001	0.001	0.568	0.365	0.020	0.806	5.950	1.097	0.367	0.020	0.800	6.223	-0.655	-0.087	1	.	.
1391	1	1	4	11	29	1	1	1	1	0.000	0.155	0.106	0.016	0.013	0.168	.	0.001	0.227	0.678	0.001	0.001	0.568	0.365	0.020	0.806	5.950	1.097	0.367	0.020	0.800	6.223	-0.655	-0.087	1	.	.

1391	1	1	4	11	30	1	2	1	2	0.176	0.103	0.090	0.010	0.079	0.182	.	0.260	0.152	0.678	0.001	0.001	0.568	0.365	0.020	0.806	5.950	1.097	0.367	0.020	0.800	6.223	-0.655	-0.087	1	.	.
1391	1	1	4	11	31	1	1	2	3	0.563	0.111	0.147	0.016	0.000	0.111	.	0.826	0.165	0.678	0.001	0.001	0.568	0.365	0.020	0.806	5.950	1.097	0.367	0.020	0.800	6.223	-0.655	-0.087	1	.	.
1391	1	1	4	11	32	1	2	2	4	0.387	0.151	0.074	0.011	0.043	0.194	.	0.566	0.219	0.689	0.001	0.001	0.568	0.365	0.020	0.806	5.950	1.097	0.367	0.020	0.800	6.223	-0.655	-0.087	1	.	.
1391	1	1	4	2	33	3	3	3	5	0.020	0.200	0.014	0.005	0.000	0.200	.	0.112	1.054	0.191	0.001	0.001	0.548	0.405	0.020	1.231	21.901	1.030	0.358	0.020	1.225	22.160	-0.385	0.163	1	.	.
1391	1	1	4	3	34	3	3	3	5	0.000	0.220	0.078	0.017	0.000	0.220	.	0.001	0.811	0.470	0.020	0.002	0.548	0.405	0.020	1.231	21.901	1.030	0.358	0.020	1.225	22.160	-0.385	0.163	1	.	0.512
1391	1	1	4	4	35	3	3	3	5	0.071	0.152	0.153	0.024	0.000	0.152	.	0.347	0.593	0.374	0.003	0.001	0.548	0.405	0.020	1.231	21.901	1.030	0.358	0.020	1.225	22.160	-0.385	0.163	1	.	.
1391	1	1	4	6	36	3	3	3	5	0.094	0.147	0.191	0.032	0.000	0.147	.	0.509	0.564	0.374	0.005	0.001	0.548	0.405	0.020	1.231	21.901	1.030	0.358	0.020	1.225	22.160	-0.385	0.163	1	.	.
1391	1	1	4	7	37	3	3	3	5	.	.	.	.	.	.	.	.	.	.	.	.	.	.	.	.	.	.	.	.	.	.	.	.	.	.	.
1391	1	1	4	8	38	3	3	3	5	-0.017	0.124	0.071	0.008	0.000	0.124	.	0.102	0.494	0.374	0.001	0.001	0.548	0.405	0.020	1.231	21.901	1.030	0.358	0.020	1.225	22.160	-0.385	0.163	1	.	.
1391	1	1	4	10	39	3	3	3	5	.	.	.	.	.	.	.	.	.	.	.	.	.	.	.	.	.	.	.	.	.	.	.	.	.	.	.
1391	1	1	4	11	40	3	3	3	5	-0.061	0.137	0.070	0.009	0.000	0.137	.	-0.210	0.604	0.374	0.001	0.001	0.548	0.405	0.020	1.231	21.901	1.030	0.358	0.020	1.225	22.160	-0.385	0.163	1	.	.
1384	1	1	1	2	1	1	1	1	1	.	.	.	.	.	.	.	.	.	.	.	.	.	.	.	.	.	.	.	.	.	.	.	.	.	.	.
1384	1	2	1	2	2	1	2	1	2	.	.	.	.	.	.	.	.	.	.	.	.	.	.	.	.	.	.	.	.	.	.	.	.	.	.	.
1384	1	2	1	2	3	1	1	2	3	.	.	.	.	.	.	.	.	.	.	.	.	.	.	.	.	.	.	.	.	.	.	.	.	.	.	.
1384	1	2	1	2	4	1	2	2	4	.	.	.	.	.	.	.	.	.	.	.	.	.	.	.	.	.	.	.	.	.	.	.	.	.	.	.
1384	1	2	1	3	5	1	1	1	1	-0.169	0.104	0.411	0.043	0.014	0.118	.	-0.294	0.168	0.627	0.014	0.002	0.565	0.250	0.005	0.358	31.588	1.211	0.234	0.005	0.342	17.282	-0.929	-0.364	2	.	.
1384	1	2	1	3	6	1	2	1	2	-0.238	0.119	0.177	0.021	-0.039	0.081	.	-0.412	0.194	0.627	0.014	0.002	0.565	0.250	0.005	0.358	31.588	1.211	0.234	0.005	0.342	17.282	-0.929	-0.364	1	.	.
1384	1	2	1	3	7	1	1	2	3	0.156	0.111	0.472	0.050	0.000	0.111	.	0.227	0.177	0.627	0.014	0.002	0.565	0.250	0.005	0.358	31.588	1.211	0.234	0.005	0.342	17.282	-0.929	-0.364	2	.	.
1384	1	2	1	3	8	1	2	2	4	-0.003	0.102	0.250	0.023	0.042	0.144	.	-0.030	0.166	0.627	0.014	0.002	0.565	0.250	0.005	0.358	31.588	1.211	0.234	0.005	0.342	17.282	-0.929	-0.364	2	.	.
1384	1	2	1	4	9	1	1	1	1	-0.069	0.141	0.332	0.045	0.016	0.157	.	-0.106	0.226	0.627	0.002	0.001	0.565	0.250	0.005	0.358	31.588	1.211	0.234	0.005	0.342	17.282	-0.929	-0.364	2	.	.
1384	1	2	1	4	10	1	2	1	2	-0.089	0.126	0.067	0.008	0.029	0.155	.	-0.148	0.201	0.627	0.002	0.001	0.565	0.250	0.005	0.358	31.588	1.211	0.234	0.005	0.342	17.282	-0.929	-0.364	2	.	.
1384	1	2	1	4	11	1	1	2	3	0.207	0.109	0.283	0.032	0.003	0.112	.	0.327	0.174	0.627	0.002	0.001	0.565	0.250	0.005	0.358	31.588	1.211	0.234	0.005	0.342	17.282	-0.929	-0.364	2	.	.
1384	1	2	1	4	12	1	2	2	4	0.251	0.117	0.111	0.012	0.008	0.125	.	0.390	0.180	0.662	0.002	0.001	0.565	0.250	0.005	0.358	31.588	1.211	0.234	0.005	0.342	17.282	-0.929	-0.364	1	.	.
1384	1	2	1	6	13	1	1	1	1	0.043	0.116	0.062	0.012	0.023	0.139	.	0.080	0.179	0.670	0.001	0.001	0.565	0.250	0.005	0.358	31.588	1.211	0.234	0.005	0.342	17.282	-0.929	-0.364	1	.	.
1384	1	2	1	6	14	1	2	1	2	0.030	0.150	0.017	0.007	0.020	0.170	.	0.042	0.231	0.670	0.001	0.001	0.565	0.250	0.005	0.358	31.588	1.211	0.234	0.005	0.342	17.282	-0.929	-0.364	1	.	.
1384	1	2	1	6	15	1	1	2	3	0.303	0.120	0.018	0.007	0.037	0.157	.	0.470	0.186	0.670	0.001	0.001	0.565	0.250	0.005	0.358	31.588	1.211	0.234	0.005	0.342	17.282	-0.929	-0.364	1	.	.
1384	1	2	1	6	16	1	2	2	4	0.436	0.150	0.016	0.007	0.000	0.150	.	0.680	0.230	0.670	0.001	0.001	0.565	0.250	0.005	0.358	31.588	1.211	0.234	0.005	0.342	17.282	-0.929	-0.364	1	.	.
1384	1	2	1	7	17	1	1	1	1	0.005	0.150	0.157	0.026	0.033	0.183	.	-0.012	0.249	0.627	0.009	0.003	0.565	0.250	0.005	0.358	31.588	1.211	0.234	0.005	0.342	17.282	-0.929	-0.364	2	.	.
1384	1	2	1	7	18	1	2	1	2	0.172	0.157	0.095	0.013	0.019	0.176	.	0.291	0.257	0.627	0.009	0.003	0.565	0.250	0.005	0.358	31.588	1.211	0.234	0.005	0.342	17.282	-0.929	-0.364	2	.	.
1384	1	2	1	7	19	1	1	2	3	.	.	.	.	.	.	.	.	.	.	.	.	.	.	.	.	.	.	.	.	.	.	.	.	.	.	.
1384	1	2	1	7	20	1	2	2	4	.	.	.	.	.	.	.	.	.	.	.	.	.	.	.	.	.	.	.	.	.	.	.	.	.	.	.
1384	1	2	1	8	21	1	1	1	1	0.028	0.115	0.341	0.041	0.016	0.131	.	0.076	0.191	0.627	0.022	0.003	0.565	0.250	0.005	0.358	31.588	1.211	0.234	0.005	0.342	17.282	-0.929	-0.364	2	.	.
1384	1	2	1	8	22	1	2	1	2	-0.058	0.118	0.237	0.029	0.017	0.135	.	-0.072	0.196	0.627	0.022	0.003	0.565	0.250	0.005	0.358	31.588	1.211	0.234	0.005	0.342	17.282	-0.929	-0.364	2	.	.
1384	1	2	1	8	23	1	1	2	3	0.220	0.113	0.328	0.035	0.016	0.129	.	0.381	0.181	0.627	0.022	0.003	0.565	0.250	0.005	0.358	31.588	1.211	0.234	0.005	0.342	17.282	-0.929	-0.364	2	.	.
1384	1	2	1	8	24	1	2	2	4	0.297	0.106	0.270	0.030	0.005	0.115	.	0.516	0.170	0.627	0.022	0.003	0.565	0.250	0.005	0.358	31.588	1.211	0.234	0.005	0.342	17.282	-0.929	-0.364	2	.	.
1384	1	2	1	10	25	1	1	1	1	.	.	.	.	.	.	.	.	.	.	.	.	.	.	.	.	.	.	.	.	.	.	.	.	.	.	.
1384	1	2	1	10	26	1	2	1	2	.	.	.	.	.	.	.	.	.	.	.	.	.	.	.	.	.	.	.	.	.	.	.	.	.	.	.
1384	1	2	1	10	27	1	1	2	3	0.248	0.151	0.217	0.033	0.025	0.177	.	0.366	0.247	0.627	0.008	0.001	0.565	0.250	0.005	0.358	31.588	1.211	0.234	0.005	0.342	17.282	-0.929	-0.364	2	.	.
1384	1	2	1	10	28	1	2	2	4	0.341	0.135	0.092	0.012	0.004	0.139	.	0.537	0.227	0.627	0.008	0.001	0.565	0.250	0.005	0.358	31.588	1.211	0.234	0.005	0.342	17.282	-0.929	-0.364	2	.	.
1384	1	2	1	11	29	1	1	1	1	0.000	0.187	0.417	0.096	0.029	0.216	.	0.001	0.296	0.627	0.019	0.003	0.565	0.250	0.005	0.358	31.588	1.211	0.234	0.005	0.342	17.282	-0.929	-0.364	2	.	.
1384	1	2	1	11	30	1	2	1	2	-0.015	0.137	0.272	0.036	0.036	0.173	.	-0.031	0.220	0.627	0.019	0.003	0.565	0.250	0.005	0.358	31.588	1.211	0.234	0.005	0.342	17.282	-0.929	-0.364	2	.	.
1384	1	2	1	11	31	1	1	2	3	0.390	0.121	0.517	0.063	0.004	0.125	.	0.606	0.188	0.648	0.019	0.003	0.565	0.250	0.005	0.358	31.588	1.211	0.234	0.005	0.342	17.282	-0.929	-0.364	2	.	.
1384	1	2	1	11	32	1	2	2	4	0.370	0.111	0.335	0.037	0.005	0.116	.	0.579	0.178	0.627	0.019	0.003	0.565	0.250	0.005	0.358	31.588	1.211	0.234	0.005	0.342	17.282	-0.929	-0.364	2	.	.
1384	1	2	1	2	33	3	3	3	5	0.000	0.204	0.010	0.010	0.000	0.204	.	0.001	1.000	0.204	0.001	0.001	0.532	0.260	0.006	0.141	26.277	1.109	0.233	0.005	0.118	26.468	-0.894	-0.362	2	.	.
1384	1	2	1	3	34	3	3	3	5	0.000	0.204	0.399	0.097	0.000	0.204	0.482	0.001	1.000	0.204	0.014	0.002	0.532	0.260	0.006	0.141	26.277	1.109	0.233	0.005	0.118	26.468	-0.894	-0.362	2	.	0.582
1384	1	2	1	4	35	3	3	3	5	0.106	0.166	0.141	0.024	0.000	0.206	.	0.614	0.887	0.204	0.002	0.001	0.532	0.260	0.006	0.141	26.277	1.109	0.233	0.005	0.118	26.468	-0.894	-0.362	2	.	.
1384	1	2	1	6	36	3	3	3	5	0.106	0.117	0.029	0.003	0.000	0.206	.	0.088	0.675	0.209	0.001	0.001	0.532	0.260	0.006	0.141	26.277	1.109	0.233	0.005	0.118	26.468	-0.894	-0.362	2	.	.
1384	1	2	1	7	37	3	3	3	5	.	.	.	.	.	.	.	.	.	.	.	.	.	.	.	.	.	.	.	.	.	.	.	.	.	.	.
1384	1	2	1	8	38	3	3	3	5	0.122	0.161	0.476	0.071	0.000	0.195	.	0.646	0.849	0.204	0.022	0.003	0.532	0.260	0.006	0.141	26.277	1.109	0.233	0.005	0.118	26.468	-0.894	-0.362	2	.	.
1384	1	2	1	10	39	3	3	3	5	.	.	.	.	.	.	.	.	.	.	.	.	.	.	.	.	.	.	.	.	.	.	.	.	.	.	.
1384	1	2	1	11	40	3	3	3	5	0.080	0.153	0.273	0.041	0.000	0.208	.	0.411	0.876	0.204	0.019	0.003	0.532	0.260	0.006	0.141	26.277	1.109	0.233	0.005	0.118	26.468	-0.894	-0.362	2	.	.
1384	1	2	2	2	1	1	1	1	1	.	.	.	.	.	.	.	.	.	.	.	.	.	.	.	.	.	.	.	.	.	.	.	.	.	.	.
1384	1	2	2	2	2	1	2	1	2	.	.	.	.	.	.	.	.	.	.	.	.	.	.	.	.	.	.	.	.	.	.	.	.	.	.	.
1384	1	2	2	2	3	1	1	2	3	.	.	.	.	.	.	.	.	.	.	.	.	.	.	.	.	.	.	.	.	.	.	.	.	.	.	.
1384	1	2	2	2	4	1	2	2	4	.	.	.	.	.	.	.	.	.	.	.	.	.	.	.	.	.	.	.	.	.	.	.	.	.	.	.
1384	1	2	2	3	5	1	1	1	1	0.040	0.133	0.583	0.077	0.040	0.172	.	0.033	0.202	0.668	0.024	0.002	0.521	0.335	0.003	0.283	20.252	1.153	0.363	0.003	0.292	20.577	-0.482	0.040	1	.	.
1384	1	2	2	3	6	1	2	1	2	0.004	0.148	0.319	0.043	0.022	0.170	.	0.009	0.222	0.668	0.024	0.002	0.521	0.335	0.003	0.283	20.252	1.153	0.363	0.003	0.292	20.577	-0.482	0.040	1	.	.
1384	1	2	2	3	7	1	1	2	3	0.364	0.115	0.712	0.084	0.027	0.142	.	0.547	0.169	0.668	0.024	0.002	0.521	0.335	0.003	0.283	20.252	1.153	0.363	0.003	0.292	20.577	-0.482	0.040	2	.	.
1384	1	2	2	3	8	1	2	2	4	0.446	0.126	0.503	0.062	0.004	0.131	.	0.642	0.193	0.668	0.025	0.004	0.521	0.335	0.003	0.283	20.252	1.153	0.363	0.003	0.292	20.577	-0.482	0.040	2	.	.
1384	1	2	2	4	9	1	1	1	1	0.007	0.153	0.483	0.076	0.024	0.177	.	-0.013	0.229	0.668	0.025	0.005	0.521	0.335	0.003	0.283	20.252	1.153	0.363	0.003	0.292	20.577	-0.482	0.040	1	.	.
1384	1	2	2	4	10	1	2	1	2	0.189	0.151	0.308	0.048	0.022	0.172	.	0.260	0.227	0.668	0.025	0.005	0.521	0.335	0.003	0.283	20.252	1.153	0.363	0.003	0.292	20.577	-0.482	0.040	1	.	.
1384	1	2	2	4	11	1	1	2	3	0.590	0.130	0.705	0.093	0.004	0.135	.	0.867	0.196	0.668	0.010	0.002	0.521	0.335	0.003	0.283	20.252	1.153	0.363	0.003	0.292	20.577	-0.482	0.040	2	.	.
1384	1	2	2	4	12	1	2	2	4	0.368	0.142	0.549	0.078	0.020	0.161	.	0.527	0.221	0.668	0.025	0.005	0.521	0.335	0.003	0.283	20.252	1.153	0.363	0.003	0.292	20.577	-0.482	0.040	2	.	.
1384	1	2	2	6	13	1	1	1	1	0.135	0.128	0.061	0.007	0.020	0.148	.	0.227	0.196	0.668	0.002	0.001	0.521	0.335	0.003	0.283	20.252	1.153	0.363	0.003	0.292	20.577	-0.482	0.040	1	.	.
1384	1	2	2	6	14	1	2	1	2	0.050	0.131	0.016	0.002	0.037	0.168	.	0.094	0.195	0.668	0.002	0.001	0.521	0.335	0.003	0.283	20.252	1.153	0.363	0.003	0.292	20.577	-0.482	0.040	1	.	.
1384	1	2	2	6	15	1	1	2	3	0.475	0.148	0.033	0.005	0.007	0.155	.	0.742	0.218	0.668	0.002	0.001	0.521	0.335	0.003	0.283	20.252	1.153	0.363	0.003	0.292	20.577	-0.482	0.040	1	.	.
1384	1	2	2	6	16	1	2	2	4	0.428	0.113	0.019	0.002	0.024	0.137	.	0.662	0.171	0.668	0.002	0.001	0.521	0.335	0.003	0.283	20.252	1.153	0.363	0.003	0.292	20.577	-0.482	0.040	1	.	.
1384	1	2	2	7	17	1	1	1	1	-0.173	0.177	0.365	0.072	0.031	0.208	.	-0.273	0.268	0.668	0.003	0.001	0.521	0.335	0.003	0.283	20.252	1.153	0.363	0.003	0.292	20.577	-0.482	0.040	1	.	.
1384	1	2	2	7	18	1	2	1	2	0.029	0.144	0.091	0.010	0.022	0.166	.	0.035	0.221	0.668	0.003	0.001	0.521	0.335	0.003	0.283	20.252	1.153	0.363	0.003	0.292	20.577	-0.482	0.040	1	.	.
1384	1	2	2	7	19	1	1	2	3	.	.	.	.	.	.	.	.	.	.	.	.	.	.	.	.	.	.	.	.	.	.	.	.	.	.	.
1384	1	2	2	7	20	1	2	2	4	.	.	.	.	.	.	.	.	.	.	.	.	.	.	.	.	.	.	.	.	.	.	.	.	.	.	.
1384	1	2	2	8	21	1	1	1	1	0.022	0.142	0.389	0.057	0.022	0.163	.	0.010	0.220	0.668	0.003	0.001	0.521	0.335	0.003	0.283	20.252	1.153	0.363	0.003	0.292	20.577	-0.482	0.040	1	.	.
1384	1	2	2	8	22	1	2	1	2	0.100	0.131	0.255	0.033	0.020	0.150	.	0.151	0.198	0.668	0.003	0.001	0.521	0.335	0.003	0.283	20.252	1.153	0.363	0.003	0.292	20.577	-0.482	0.040	1	.	.
1384	1	2	2	8	23	1	1	2	3	0.382	0.091	0.653	0.059	0.015	0.106	.	0.567	0.138	0.668	0.003	0.001	0.521	0.335	0.003	0.283	20.252	1.153	0.363	0.003	0.292	20.577	-0.482	0.040	2	.	.
1384	1	2	2	8	24	1	2	2	4	0.388	0.117	0.566	0.065	0.011	0.128	.	0.582	0.179	0.668	0.003	0.001	0.521	0.335	0.003	0.283	20.252	1.153	0.363	0.003	0.292	20.577	-0.482	0.040	2	.	.
1384	1	2	2	10	25	1	1	1	1	.	.	.	.	.	.	.	.	.	.	.	.	.	.	.	.	.	.	.	.	.	.	.	.	.	.	.
1384	1	2	2	10	26	1	2	1	2	.	.	.	.	.	.	.	.	.	.	.	.	.	.	.	.	.	.	.	.	.	.	.	.	.	.	.
1384	1	2	2	10	27	1	1	2	3	0.630	0.142	0.154	0.021	0.000	0.142	.	0.938	0.215	0.668	0.003	0.001	0.521	0.335	0.003	0.283	20.252	1.153	0.363	0.003	0.292	20.577	-0.482	0.040	2	.	.
1384	1	2	2	10	28	1	2	2	4	0.373	0.199	0.041	0.007	0.026	0.226	.	0.556	0.298	0.668	0.003	0.001	0.521	0.335	0.003	0.283	20.252	1.153	0.363	0.003	0.292	20.577	-0.482	0.040	2	.	.
1384	1	2	2	11	29	1	1	1	1	0.000	0.144	0.129	0.016	0.013	0.157	.	0.001	0.221	0.668	0.003	0.001	0.521	0.335	0.003	0.283	20.252	1.153	0.363	0.003	0.292	20.577	-0.482	0.040	1	.	.
1384	1	2	2	11	30	1	2	1	2	0.182	0.117	0.066	0.008	0.042	0.159	.	0.278	0.177	0.668	0.003	0.001	0.521	0.335	0.003	0.283	20.252	1.153	0.363	0.003	0.292	20.577	-0.482	0.040	1	.	.
1384	1	2	2	11	31	1	1	2	3	0.517	0.139	0.228	0.029	0.004	0.144	.	0.785	0.209	0.668	0.010	0.001	0.521	0.335	0.003	0.283	20.252	1.153	0.363	0.003	0.292	20.577	-0.482	0.040	2	.	.
1384	1	2	2	11	32	1	2	2	4	0.418	0.126	0.181	0.024	0.013	0.139	.	0.622	0.187	0.668	0.010	0.001	0.521	0.335	0.003	0.283	20.252	1.153	0.363	0.003	0.292	20.577	-0.482	0.661	2	.	.
1384	1	2	2	2	33	3	3	3	5	0.000	0.140	0.010	0.010	0.000	0.140	.	0.001	0.935	0.150	0.002	0.001	0.449	0.346	0.003	0.257	22.760	0.932	0.347	0.003	0.257	22.974	-0.430	0.020	2	.	.
1384	1	2	2	3	34	3	3	3	5	0.000	0.140	0.618	0.087	0.000	0.140	0.685	0.001	0.935	0.150	0.002	0.001	0.449	0.346	0.003	0.257	22.760	0.932	0.347	0.003	0.257	22.974	-0.430	0.020	2	.	.
1384	1	2	2	4	35	3	3	3	5	0.070	0.133	0.655	0.088	0.000	0.133	.	0.474	0.885	0.150	0.003	0.001	0.449	0.346	0.003	0.257	22.760	0.932	0.347	0.003	0.257	22.974	-0.430	0.020	2	.	.
1384	1	2	2	6	36	3	3	3	5	-0.023	0.123	0.013	0.001	0.000	0.123	.	-0.152	0.817	0.150	0.003	0.001	0.449	0.346	0.003	0.257	22.760	0.932	0.347	0.003	0.257	22.974	-0.430	0.020	2	.	.
1384	1	2	2	7	37	3	3	3	5	.	.	.	.	.	.	.	.	.	.	.	.	.	.	.	.	.	.	.	.	.	.	.	.	.	.	.
1384	1	2	2	8	38	3	3	3	5	0.005	0.113	0.526	0.052	0.000	0.113	.	0.020	0.753	0.150	0.003	0.001	0.449	0.346	0.003	0.257	22.760	0.932	0.347	0.003	0.257	22.974	-0.430	0.020	2	.	.
1384	1	2	2	10	39	3	3	3	5	.	.	.	.	.	.	.	.	.	.	.	.	.	.	.	.	.	.	.	.	.	.	.	.	.	.	.
1384	1	2	2	11	40	3	3	3	5	0.106	0.153	0.055	0.010	0.000	0.153	.	0.711	1.022	0.150	0.003	0.001	0.449	0.346	0.003	0.257	22.760	0.932	0.347	0.003	0.257	22.974	-0.430	0.020	2	.	.
1384	1	2	3	2	1	1	1	1	1	.	.	.	.	.	.	.	.	.	.	.	.	.	.	.	.	.	.	.	.	.	.	.	.	.	.	.
1384	1	2	3	2	2	1	2	1	2	.	.	.	.	.	.	.	.	.	.	.	.	.	.	.	.	.	.	.	.	.	.	.	.	.	.	.
1384	1	2	3	2	3	1	1	2	3	.	.	.	.	.	.	.	.	.	.	.	.	.	.	.	.	.	.	.	.	.	.	.	.	.	.	.
1384	1	2	3	2	4	1	2	2	4	.	.	.	.	.	.	.	.	.	.	.	.	.	.	.	.	.	.	.	.	.	.	.	.	.	.	.
1384	1	2	3	3	5	1	1	1	1	.	.	.	.	.	.	.	.	.	.	.	.	.	.	.	.	.	.	.	.	.	.	.	.	.	.	.
1384	1	2	3	3	6	1	2	1	2	.	.	.	.	.	.	.	.	.	.	.	.	.	.	.	.	.	.	.	.	.	.	.	.	.	.	.
1384	1	2	3	3	7	1	1	2	3	.	.	.	.	.	.	.	.	.	.	.	.	.	.	.	.	.	.	.	.	.	.	.	.	.	.	.
1384	1	2	3	3	8	1	2	2	4	.	.	.	.	.	.	.	.	.	.	.	.	.	.	.	.	.	.	.	.	.	.	.	.	.	.	.
1384	1	2	3	4	9	1	1	1	1	0.089	0.148	0.190	0.024	0.042	0.190	.	0.136	0.235	0.627	0.007	0.001	0.510	0.260	0.012	0.378	36.170	0.919	0.343	0.013	0.322	36.406	-0.494	0.016	2	.	.
1384	1	2	3	4	10	1	2	1	2	0.065	0.134	0.088	0.010	0.095	0.228	.	0.093	0.212	0.627	0.007	0.001	0.510	0.260	0.012	0.378	36.170	0.919	0.343	0.013	0.322	36.406	-0.494	0.016	2	.	.
1384	1	2	3	4	11	1	1	2	3	0.450	0.120	0.122	0.013	0.024	0.144	.	0.700	0.191	0.627	0.007	0.001	0.510	0.260	0.012	0.378	36.170	0.919	0.343	0.013	0.322	36.406	-0.494	0.016	2	.	.
1384	1	2	3	4	12	1	2	2	4	0.600	0.141	0.091	0.012	0.000	0.141	.	0.940	0.224	0.627	0.007	0.001	0.510	0.260	0.012	0.378	36.170	0.919	0.343	0.013	0.322	36.406	-0.494	0.016	2	.	.
1384	1	2	3	6	13	1	1	1	1	-0.187	0.163	0.113	0.020	0.039	0.202	.	-0.296	0.264	0.627	0.003	0.001	0.510	0.260	0.012	0.378	36.170	0.919	0.343	0.013	0.322	36.406	-0.494	0.016	1	.	.
1384	1	2	3	6	14	1	2	1	2	-0.080	0.141	0.018	0.002	0.030	0.171	.	-0.111	0.228	0.627	0.003	0.001	0.510	0.260	0.012	0.378	36.170	0.919	0.343	0.013	0.322	36.406	-0.494	0.016	2	.	.
1384	1	2	3	6	15	1	1	2	3	0.260	0.120	0.058	0.007	0.013	0.133	.	0.434	0.192	0.627	0.003	0.001	0.510	0.260	0.012	0.378	36.170	0.919	0.343	0.013	0.322	36.406	-0.494	0.016	2	.	.
1384	1	2	3	6	16	1	2	2	4	0.208	0.119	0.023	0.002	0.027	0.146	.	0.341	0.190	0.627	0.003	0.001	0.510	0.260	0.012	0.378	36.170	0.919	0.343	0.013	0.322	36.406	-0.494	0.016	2	.	.
1384	1	2	3	7	17	1	1	1	1	-0.003	0.144	0.034	0.011	0.095	0.239	.	-0.007	0.233	0.627	0.001	0.001	0.510	0.260	0.012	0.378	36.170	0.919	0.343	0.013	0.322	36.406	-0.494	0.016	1	.	.
1384	1	2	3	7	18	1	2	1	2	0.286	0.124	0.025	0.009	0.015	0.139	.	0.452	0.199	0.627	0.001	0.001	0.510	0.260	0.012	0.378	36.170	0.919	0.343	0.013	0.322	36.406	-0.494	0.016	2	.	.
1384	1	2	3	7	19	1	1	2	3	.	.	.	.	.	.	.	.	.	.	.	.	.	.	.	.	.	.	.	.	.	.	.	.	.	.	.
1384	1	2	3	7	20	1	2	2	4	.	.	.	.	.	.	.	.	.	.	.	.	.	.	.	.	.	.	.	.	.	.	.	.	.	.	.
1384	1	2	3	8	21	1	1	1	1	0.323	0.114	0.599	0.068	0.054	0.167	.	0.495	0.182	0.627	0.007	0.001	0.510	0.260	0.012	0.378	36.170	0.919	0.343	0.013	0.322	36.406	-0.494	0.016	2	.	.
1384	1	2	3	8	22	1	2	1	2	0.372	0.100	0.485	0.047	0.030	0.130	.	0.579	0.161	0.627	0.007	0.001	0.510	0.260	0.012	0.378	36.170	0.919	0.343	0.013	0.322	36.406	-0.494	0.016	2	.	.
1384	1	2	3	8	23	1	1	2	3	0.608	0.115	0.478	0.056	0.011	0.126	.	0.951	0.185	0.627	0.007	0.001	0.510	0.260	0.012	0.378	36.170	0.919	0.343	0.013	0.322	36.406	-0.494	0.016	2	.	.
1384	1	2	3	8	24	1	2	2	4	0.647	0.098	0.337	0.034	0.002	0.100	.	1.024	0.156	0.627	0.007	0.001	0.510	0.260	0.012	0.378	36.170	0.919	0.343	0.013	0.322	36.406	-0.494	0.016	2	.	.
1384	1	2	3	10	25	1	1	1	1	.	.	.	.	.	.	.	.	.	.	.	.	.	.	.	.	.	.	.	.	.	.	.	.	.	.	.
1384	1	2	3	10	26	1	2	1	2	.	.	.	.	.	.	.	.	.	.	.	.	.	.	.	.	.	.	.	.	.	.	.	.	.	.	.
1384	1	2	3	10	27	1	1	2	3	0.134	0.145	0.042	0.006	0.016	0.161	.	0.203	0.230	0.627	0.002	0.001	0.510	0.260	0.012	0.378	36.170	0.919	0.343	0.013	0.322	36.406	-0.494	0.016	2	.	.
1384	1	2	3	10	28	1	2	2	4	0.377	0.169	0.036	0.006	0.000	0.169	.	0.588	0.273	0.627	0.002	0.001	0.510	0.260	0.012	0.378	36.170	0.919	0.343	0.013	0.322	36.406	-0.494	0.016	2	.	.
1384	1	2	3	11	29	1	1	1	1	0.000	0.141	0.201	0.024	0.043	0.183	.	0.001	0.224	0.627	0.001	0.001	0.510	0.260	0.012	0.378	36.170	0.919	0.343	0.013	0.322	36.406	-0.494	0.016	2	.	.
1384	1	2	3	11	30	1	2	1	2	0.093	0.135	0.098	0.014	0.013	0.148	.	0.132	0.217	0.627	0.001	0.001	0.510	0.260	0.012	0.378	36.170	0.919	0.343	0.013	0.322	36.406	-0.494	0.016	2	.	.
1384	1	2	3	11	31	1	1	2	3	0.444	0.121	0.136	0.019	0.000	0.121	.	0.702	0.196	0.627	0.001	0.001	0.510	0.260	0.012	0.378	36.170	0.919	0.343	0.013	0.322	36.406	-0.494	0.016	2	.	.
1384	1	2	3	11	32	1	2	2	4	0.307	0.111	0.106	0.012	0.016	0.127	.	0.483	0.178	0.627	0.001	0.001	0.510	0.260	0.012	0.378	36.170	0.919	0.343	0.013	0.322	36.406	-0.494	0.016	2	.	.
1384	1	2	3	2	33	3	3	3	5	0.000	0.138	0.220	0.027	0.000	0.138	.	0.001	1.000	0.138	0.001	0.001	0.399	0.247	0.012	0.212	37.721	0.780	0.261	0.012	0.215	37.862	-0.364	0.035	1	.	.
1384	1	2	3	3	34	3	3	3	5	0.000	0.139	0.010	0.010	0.000	0.139	0.687	0.001	1.007	0.138	0.001	0.001	0.399	0.247	0.012	0.212	37.721	0.780	0.261	0.012	0.215	37.862	-0.364	0.035	1	.	0.256
1384	1	2	3	4	35	3	3	3	5	0.104	0.108	0.241	0.025	0.000	0.108	.	0.746	0.779	0.138	0.012	0.001	0.399	0.247	0.012	0.212	37.721	0.780	0.261	0.012	0.215	37.862	-0.364	0.035	1	.	.
1384	1	2	3	6	36	3	3	3	5	0.067	0.120	0.113	0.010	0.000	0.120	.	0.491	0.872	0.138	0.005	0.001	0.399	0.247	0.012	0.212	37.721	0.780	0.261	0.012	0.215	37.862	-0.364	0.035	1	.	.
1384	1	2	3	7	37	3	3	3	5	.	.	.	.	.	.	.	.	.	.	.	.	.	.	.	.	.	.	.	.	.	.	.	.	.	.	.
1384	1	2	3	8	38	3	3	3	5	0.085	0.115	0.405	0.047	0.000	0.115	.	0.613	0.831	0.138	0.013	0.001	0.399	0.247	0.012	0.212	37.721	0.780	0.261	0.012	0.215	37.862	-0.364	0.035	1	.	.
1384	1	2	3	10	39	3	3	3	5	.	.	.	.	.	.	.	.	.	.	.	.	.	.	.	.	.	.	.	.	.	.	.	.	.	.	.
1384	1	2	3	11	40	3	3	3	5	0.151	0.137	0.022	0.003	0.000	0.137	.	1.068	0.970	0.141	0.002	0.001	0.399	0.247	0.012	0.212	37.721	0.780	0.261	0.012	0.215	37.862	-0.364	0.035	1	.	.
1384	1	2	4	2	1	1	1	1	1	.	.	.	.	.	.	.	.	.	.	.	.	.	.	.	.	.	.	.	.	.	.	.	.	.	.	.
1384	1	2	4	2	2	1	2	1	2	.	.	.	.	.	.	.	.	.	.	.	.	.	.	.	.	.	.	.	.	.	.	.	.	.	.	.
1384	1	2	4	2	3	1	1	2	3	.	.	.	.	.	.	.	.	.	.	.	.	.	.	.	.	.	.	.	.	.	.	.	.	.	.	.
1384	1	2	4	2	4	1	2	2	4	.	.	.	.	.	.	.	.	.	.	.	.	.	.	.	.	.	.	.	.	.	.	.	.	.	.	.
1384	1	2	4	3	5	1	1	1	1	-0.230	0.157	0.067	0.010	0.053	0.192	.	-0.440	0.307	0.536	0.006	0.001	0.627	0.099	0.007	0.002	0.039	1.205	0.154	0.007	0.008	0.170	-0.992	-0.365	1	.	.
1384	1	2	4	3	6	1	2	1	2	-0.013	0.126	0.054	0.004	0.024	0.145	.	0.002	0.240	0.536	0.006	0.001	0.627	0.099	0.007	0.002	0.039	1.205	0.154	0.007	0.008	0.170	-0.992	-0.365	1	.	.
1384	1	2	4	3	7	1	1	2	3	0.191	0.122	0.088	0.009	0.024	0.152	.	0.394	0.234	0.536	0.006	0.001	0.627	0.099	0.007	0.002	0.039	1.205	0.154	0.007	0.008	0.170	-0.992	-0.365	2	.	.
1384	1	2	4	3	8	1	2	2	4	0.271	0.109	0.024	0.004	0.002	0.117	.	0.556	0.211	0.536	0.006	0.001	0.627	0.099	0.007	0.002	0.039	1.205	0.154	0.007	0.008	0.170	-0.992	-0.365	2	.	.

1384	1	2	4	4	9	1	1	1	1	-0.091	0.158	0.135	0.021	0.024	0.163	.	-0.097	0.292	0.536	0.007	0.001	0.627	0.099	0.007	0.002	0.039	1.205	0.154	0.007	0.008	0.170	-0.992	-0.365	2	.	.
1384	1	2	4	4	10	1	2	1	2	-0.041	0.134	0.074	0.010	0.051	0.170	.	-0.059	0.259	0.536	0.007	0.001	0.627	0.099	0.007	0.002	0.039	1.205	0.154	0.007	0.008	0.170	-0.992	-0.365	1	.	.
1384	1	2	4	4	11	1	1	2	3	0.315	0.115	0.167	0.019	0.005	0.124	.	0.677	0.219	0.536	0.007	0.001	0.627	0.099	0.007	0.002	0.039	1.205	0.154	0.007	0.008	0.170	-0.992	-0.365	2	.	.
1384	1	2	4	4	12	1	2	2	4	0.268	0.120	0.041	0.004	0.009	0.135	.	0.544	0.236	0.536	0.007	0.001	0.627	0.099	0.007	0.002	0.039	1.205	0.154	0.007	0.008	0.170	-0.992	-0.365	2	.	.
1384	1	2	4	6	13	1	1	1	1	-0.093	0.139	0.397	0.054	0.024	0.141	.	-0.124	0.274	0.536	0.003	0.001	0.627	0.099	0.007	0.002	0.039	1.205	0.154	0.007	0.008	0.170	-0.992	-0.365	2	.	.
1384	1	2	4	6	14	1	2	1	2	-0.156	0.154	0.255	0.040	0.045	0.167	.	-0.245	0.288	0.536	0.003	0.001	0.627	0.099	0.007	0.002	0.039	1.205	0.154	0.007	0.008	0.170	-0.992	-0.365	1	.	.
1384	1	2	4	6	15	1	1	2	3	0.290	0.130	0.274	0.035	0.013	0.126	.	0.626	0.249	0.536	0.003	0.001	0.627	0.099	0.007	0.002	0.039	1.205	0.154	0.007	0.008	0.170	-0.992	-0.365	2	.	.
1384	1	2	4	6	16	1	2	2	4	0.226	0.137	0.093	0.013	0.029	0.168	.	0.494	0.264	0.536	0.003	0.001	0.627	0.099	0.007	0.002	0.039	1.205	0.154	0.007	0.008	0.170	-0.992	-0.365	2	.	.
1384	1	2	4	7	17	1	1	1	1	0.002	0.122	0.017	0.002	0.056	0.145	.	0.006	0.234	0.536	0.001	0.001	0.627	0.099	0.007	0.002	0.039	1.205	0.154	0.007	0.008	0.170	-0.992	-0.365	1	.	.
1384	1	2	4	7	18	1	2	1	2	0.080	0.135	0.008	0.004	0.013	0.143	.	0.160	0.259	0.536	0.001	0.001	0.627	0.099	0.007	0.002	0.039	1.205	0.154	0.007	0.008	0.170	-0.992	-0.365	2	.	.
1384	1	2	4	7	19	1	1	2	3	.	.	.	.	.	.	.	.	.	.	.	.	.	.	.	.	.	.	.	.	.	.	.	.	.	.	.
1384	1	2	4	7	20	1	2	2	4	.	.	.	.	.	.	.	.	.	.	.	.	.	.	.	.	.	.	.	.	.	.	.	.	.	.	.
1384	1	2	4	8	21	1	1	1	1	-0.215	0.128	0.279	0.035	0.035	0.148	.	-0.404	0.245	0.536	0.017	0.001	0.627	0.099	0.007	0.002	0.039	1.205	0.154	0.007	0.008	0.170	-0.992	-0.365	1	.	.
1384	1	2	4	8	22	1	2	1	2	-0.052	0.116	0.095	0.011	0.065	0.170	.	-0.081	0.224	0.536	0.017	0.001	0.627	0.099	0.007	0.002	0.039	1.205	0.154	0.007	0.008	0.170	-0.992	-0.365	1	.	.
1384	1	2	4	8	23	1	1	2	3	0.260	0.125	0.179	0.022	0.005	0.123	.	0.575	0.242	0.536	0.017	0.001	0.627	0.099	0.007	0.002	0.039	1.205	0.154	0.007	0.008	0.170	-0.992	-0.365	2	.	.
1384	1	2	4	8	24	1	2	2	4	0.234	0.145	0.074	0.011	0.028	0.176	.	0.466	0.289	0.536	0.017	0.001	0.627	0.099	0.007	0.002	0.039	1.205	0.154	0.007	0.008	0.170	-0.992	-0.365	2	.	.
1384	1	2	4	10	25	1	1	1	1	.	.	.	.	.	.	.	.	.	.	.	.	.	.	.	.	.	.	.	.	.	.	.	.	.	.	.
1384	1	2	4	10	26	1	2	1	2	.	.	.	.	.	.	.	.	.	.	.	.	.	.	.	.	.	.	.	.	.	.	.	.	.	.	.
1384	1	2	4	10	27	1	1	2	3	.	.	.	.	.	.	.	.	.	.	.	.	.	.	.	.	.	.	.	.	.	.	.	.	.	.	.
1384	1	2	4	10	28	1	2	2	4	.	.	.	.	.	.	.	.	.	.	.	.	.	.	.	.	.	.	.	.	.	.	.	.	.	.	.
1384	1	2	4	11	29	1	1	1	1	0.000	0.112	0.056	0.006	0.014	0.126	.	0.001	0.210	0.536	0.001	0.001	0.627	0.099	0.007	0.002	0.039	1.205	0.154	0.007	0.008	0.170	-0.992	-0.365	1	.	.
1384	1	2	4	11	30	1	2	1	2	0.028	0.095	0.038	0.003	0.035	0.123	.	0.053	0.177	0.536	0.001	0.001	0.627	0.099	0.007	0.002	0.039	1.205	0.154	0.007	0.008	0.170	-0.992	-0.365	1	.	.
1384	1	2	4	11	31	1	1	2	3	0.336	0.092	0.031	0.002	0.003	0.103	.	0.602	0.170	0.536	0.001	0.001	0.627	0.099	0.007	0.002	0.039	1.205	0.154	0.007	0.008	0.170	-0.992	-0.365	1	.	.
1384	1	2	4	11	32	1	2	2	4	0.266	0.104	0.017	0.002	0.015	0.126	.	0.495	0.194	0.536	0.001	0.001	0.627	0.099	0.007	0.002	0.039	1.205	0.154	0.007	0.008	0.170	-0.992	-0.365	1	.	.
1384	1	2	4	2	33	3	3	3	5	0.000	0.132	0.010	0.010	0.000	0.134	.	0.001	1.000	0.132	0.001	0.001	0.529	0.078	0.007	0.007	0.056	1.128	0.303	0.007	0.035	0.159	-0.758	-0.233	2	.	.
1384	1	2	4	3	34	3	3	3	5	0.000	0.132	0.055	0.007	0.000	0.134	0.586	0.001	1.000	0.132	0.006	0.001	0.529	0.078	0.007	0.007	0.056	1.128	0.303	0.007	0.035	0.159	-0.758	-0.226	2	0.780	1.400
1384	1	2	4	4	35	3	3	3	5	0.000	0.126	0.246	0.031	0.000	0.128	.	0.001	0.955	0.132	0.007	0.001	0.529	0.078	0.007	0.007	0.056	1.128	0.303	0.007	0.035	0.159	-0.758	-0.226	2	.	.
1384	1	2	4	6	36	3	3	3	5	0.010	0.125	0.443	0.054	0.000	0.124	.	0.069	0.955	0.132	0.003	0.001	0.529	0.078	0.007	0.007	0.056	1.128	0.303	0.007	0.035	0.159	-0.758	-0.226	2	.	.
1384	1	2	4	7	37	3	3	3	5	.	.	.	.	.	.	.	.	.	.	.	.	.	.	.	.	.	.	.	.	.	.	.	.	.	.	.
1384	1	2	4	8	38	3	3	3	5	-0.005	0.121	0.044	0.005	0.000	0.126	.	-0.041	0.960	0.126	0.017	0.001	0.529	0.078	0.007	0.007	0.056	1.128	0.303	0.007	0.035	0.159	-0.758	-0.226	2	.	.
1384	1	2	4	10	39	3	3	3	5	.	.	.	.	.	.	.	.	.	.	.	.	.	.	.	.	.	.	.	.	.	.	.	.	.	.	.
1384	1	2	4	11	40	3	3	3	5	0.078	0.117	0.041	0.006	0.000	0.128	.	0.580	0.875	0.135	0.001	0.001	0.529	0.078	0.007	0.007	0.056	1.128	0.303	0.007	0.035	0.159	-0.758	-0.226	2	.	.
3370	2	1	1	2	1	1	1	1	1	.	.	.	.	.	.	.	.	.	.	.	.	.	.	.	.	.	.	.	.	.	.	.	.	.	.	.
3370	2	1	1	2	2	1	2	1	2	.	.	.	.	.	.	.	.	.	.	.	.	.	.	.	.	.	.	.	.	.	.	.	.	.	.	.
3370	2	1	1	2	3	1	1	2	3	.	.	.	.	.	.	.	.	.	.	.	.	.	.	.	.	.	.	.	.	.	.	.	.	.	.	.
3370	2	1	1	2	4	1	2	2	4	.	.	.	.	.	.	.	.	.	.	.	.	.	.	.	.	.	.	.	.	.	.	.	.	.	.	.
3370	2	1	1	3	5	1	1	1	1	-0.018	0.111	0.287	0.029	0.017	0.129	.	-0.033	0.200	0.549	0.005	0.001	0.373	0.134	0.001	0.003	0.124	0.917	0.102	0.002	0.003	0.197	-0.675	-0.303	2	.	.
3370	2	1	1	3	6	1	2	1	2	-0.098	0.108	0.162	0.015	0.016	0.124	.	-0.181	0.194	0.549	0.005	0.001	0.373	0.134	0.001	0.003	0.124	0.917	0.102	0.002	0.003	0.197	-0.675	-0.303	2	.	.
3370	2	1	1	3	7	1	1	2	3	0.166	0.127	0.230	0.029	0.026	0.153	.	0.293	0.228	0.549	0.005	0.001	0.373	0.134	0.001	0.003	0.124	0.917	0.102	0.002	0.003	0.197	-0.675	-0.303	2	.	.
3370	2	1	1	3	8	1	2	2	4	0.225	0.134	0.203	0.026	0.106	0.229	.	0.372	0.235	0.549	0.005	0.001	0.373	0.134	0.001	0.003	0.124	0.917	0.102	0.002	0.003	0.197	-0.675	-0.303	2	.	.
3370	2	1	1	4	9	1	1	1	1	-0.139	0.102	0.201	0.020	0.016	0.118	.	-0.255	0.188	0.549	0.007	0.001	0.373	0.134	0.001	0.003	0.124	0.917	0.102	0.002	0.003	0.197	-0.675	-0.303	2	.	.
3370	2	1	1	4	10	1	2	1	2	-0.143	0.115	0.126	0.013	0.015	0.130	.	-0.271	0.214	0.549	0.007	0.001	0.373	0.134	0.001	0.003	0.124	0.917	0.102	0.002	0.003	0.197	-0.675	-0.303	2	.	.
3370	2	1	1	4	11	1	1	2	3	0.048	0.120	0.241	0.023	0.033	0.150	.	0.047	0.210	0.549	0.007	0.001	0.373	0.134	0.001	0.003	0.124	0.917	0.102	0.002	0.003	0.197	-0.675	-0.303	2	.	.
3370	2	1	1	4	12	1	2	2	4	0.224	0.121	0.109	0.012	0.133	0.255	.	0.373	0.231	0.549	0.007	0.001	0.373	0.134	0.001	0.003	0.124	0.917	0.102	0.002	0.003	0.197	-0.675	-0.303	2	.	.
3370	2	1	1	6	13	1	1	1	1	-0.171	0.121	0.178	0.018	0.025	0.146	.	-0.317	0.222	0.549	0.009	0.001	0.373	0.134	0.001	0.003	0.124	0.917	0.102	0.002	0.003	0.197	-0.675	-0.303	2	.	.
3370	2	1	1	6	14	1	2	1	2	-0.195	0.108	0.127	0.012	0.013	0.121	.	-0.356	0.197	0.549	0.009	0.001	0.373	0.134	0.001	0.003	0.124	0.917	0.102	0.002	0.003	0.197	-0.675	-0.303	2	.	.
3370	2	1	1	6	15	1	1	2	3	0.064	0.144	0.184	0.022	0.049	0.193	.	0.108	0.255	0.549	0.009	0.001	0.373	0.134	0.001	0.003	0.124	0.917	0.102	0.002	0.003	0.197	-0.675	-0.303	2	.	.
3370	2	1	1	6	16	1	2	2	4	0.173	0.126	0.154	0.018	0.055	0.181	.	0.311	0.228	0.549	0.009	0.001	0.373	0.134	0.001	0.003	0.124	0.917	0.102	0.002	0.003	0.197	-0.675	-0.303	2	.	.
3370	2	1	1	7	17	1	1	1	1	-0.120	0.124	0.332	0.037	0.019	0.142	.	-0.226	0.224	0.549	0.002	0.001	0.373	0.134	0.001	0.003	0.124	0.917	0.102	0.002	0.003	0.197	-0.675	-0.303	2	.	.
3370	2	1	1	7	18	1	2	1	2	-0.029	0.113	0.266	0.030	0.088	0.203	.	-0.051	0.205	0.549	0.002	0.001	0.373	0.134	0.001	0.003	0.124	0.917	0.102	0.002	0.003	0.197	-0.675	-0.303	2	.	.
3370	2	1	1	7	19	1	1	2	3	.	.	.	.	.	.	.	.	.	.	.	.	.	.	.	.	.	.	.	.	.	.	.	.	.	.	.
3370	2	1	1	7	20	1	2	2	4	.	.	.	.	.	.	.	.	.	.	.	.	.	.	.	.	.	.	.	.	.	.	.	.	.	.	.
3370	2	1	1	8	21	1	1	1	1	-0.066	0.114	0.291	0.031	0.029	0.143	.	-0.124	0.208	0.549	0.007	0.002	0.373	0.134	0.001	0.003	0.124	0.917	0.102	0.002	0.003	0.197	-0.675	-0.303	2	.	.
3370	2	1	1	8	22	1	2	1	2	-0.054	0.107	0.231	0.023	0.015	0.123	.	-0.103	0.195	0.549	0.007	0.002	0.373	0.134	0.001	0.003	0.124	0.917	0.102	0.002	0.003	0.197	-0.675	-0.303	2	.	.
3370	2	1	1	8	23	1	1	2	3	0.166	0.132	0.278	0.037	0.027	0.153	.	0.249	0.232	0.549	0.007	0.002	0.373	0.134	0.001	0.003	0.124	0.917	0.102	0.002	0.003	0.197	-0.675	-0.303	2	.	.
3370	2	1	1	8	24	1	2	2	4	0.256	0.115	0.212	0.024	0.127	0.239	.	0.500	0.204	0.549	0.007	0.002	0.373	0.134	0.001	0.003	0.124	0.917	0.102	0.002	0.003	0.197	-0.675	-0.303	2	.	.
3370	2	1	1	10	25	1	1	1	1	.	.	.	.	.	.	.	.	.	.	.	.	.	.	.	.	.	.	.	.	.	.	.	.	.	.	.
3370	2	1	1	10	26	1	2	1	2	.	.	.	.	.	.	.	.	.	.	.	.	.	.	.	.	.	.	.	.	.	.	.	.	.	.	.
3370	2	1	1	10	27	1	1	2	3	.	.	.	.	.	.	.	.	.	.	.	.	.	.	.	.	.	.	.	.	.	.	.	.	.	.	.
3370	2	1	1	10	28	1	2	2	4	.	.	.	.	.	.	.	.	.	.	.	.	.	.	.	.	.	.	.	.	.	.	.	.	.	.	.
3370	2	1	1	11	29	1	1	1	1	0.000	0.129	0.231	0.023	0.021	0.150	.	0.001	0.235	0.549	0.001	0.001	0.373	0.134	0.001	0.003	0.124	0.917	0.102	0.002	0.003	0.197	-0.675	-0.303	2	.	.
3370	2	1	1	11	30	1	2	1	2	-0.132	0.120	0.098	0.011	0.016	0.135	.	-0.239	0.218	0.549	0.001	0.001	0.373	0.134	0.001	0.003	0.124	0.917	0.102	0.002	0.003	0.197	-0.675	-0.303	2	.	.
3370	2	1	1	11	31	1	1	2	3	0.081	0.138	0.233	0.032	0.013	0.151	.	0.153	0.253	0.549	0.001	0.001	0.373	0.134	0.001	0.003	0.124	0.917	0.102	0.002	0.003	0.197	-0.675	-0.303	2	.	.
3370	2	1	1	11	32	1	2	2	4	0.308	0.149	0.077	0.009	0.085	0.234	.	0.562	0.273	0.549	0.001	0.001	0.373	0.134	0.001	0.003	0.124	0.917	0.102	0.002	0.003	0.197	-0.675	-0.303	2	.	.
3370	2	1	1	2	33	3	3	3	5	.	.	.	.	.	.	.	.	.	.	.	.	.	.	.	.	.	.	.	.	.	.	.	.	.	.	.
3370	2	1	1	3	34	3	3	3	5	0.000	0.137	0.321	0.044	0.000	0.137	0.585	0.001	0.710	0.193	0.001	0.001	0.281	0.141	0.001	0.002	0.130	0.668	0.094	0.001	0.004	0.190	-0.474	-0.193	2	0.172	0.136
3370	2	1	1	4	35	3	3	3	5	0.012	0.111	0.120	0.010	0.000	0.111	.	0.098	0.576	0.193	0.001	0.001	0.281	0.141	0.001	0.002	0.130	0.668	0.094	0.001	0.004	0.190	-0.474	-0.193	2	.	.
3370	2	1	1	6	36	3	3	3	5	0.006	0.143	0.236	0.031	0.000	0.143	.	0.037	0.731	0.193	0.001	0.001	0.281	0.141	0.001	0.002	0.130	0.668	0.094	0.001	0.004	0.190	-0.474	-0.193	2	.	.
3370	2	1	1	7	37	3	3	3	5	.	.	.	.	.	.	.	.	.	.	.	.	.	.	.	.	.	.	.	.	.	.	.	.	.	.	.
3370	2	1	1	8	38	3	3	3	5	0.009	0.130	0.283	0.035	0.000	0.130	.	0.048	0.664	0.193	0.001	0.001	0.281	0.141	0.001	0.002	0.130	0.668	0.094	0.001	0.004	0.190	-0.474	-0.193	2	.	.
3370	2	1	1	10	39	3	3	3	5	.	.	.	.	.	.	.	.	.	.	.	.	.	.	.	.	.	.	.	.	.	.	.	.	.	.	.
3370	2	1	1	11	40	3	3	3	5	0.019	0.098	0.017	0.004	0.000	0.098	.	0.096	0.543	0.193	0.001	0.001	0.281	0.141	0.001	0.002	0.130	0.668	0.094	0.001	0.004	0.190	-0.474	-0.193	2	.	.
3370	2	1	2	2	1	1	1	1	1	.	.	.	.	.	.	.	.	.	.	.	.	.	.	.	.	.	.	.	.	.	.	.	.	.	.	.
3370	2	1	2	2	2	1	2	1	2	.	.	.	.	.	.	.	.	.	.	.	.	.	.	.	.	.	.	.	.	.	.	.	.	.	.	.
3370	2	1	2	2	3	1	1	2	3	.	.	.	.	.	.	.	.	.	.	.	.	.	.	.	.	.	.	.	.	.	.	.	.	.	.	.
3370	2	1	2	2	4	1	2	2	4	.	.	.	.	.	.	.	.	.	.	.	.	.	.	.	.	.	.	.	.	.	.	.	.	.	.	.
3370	2	1	2	3	5	1	1	1	1	.	.	.	.	.	.	.	.	.	.	.	.	.	.	.	.	.	.	.	.	.	.	.	.	.	.	.
3370	2	1	2	3	6	1	2	1	2	.	.	.	.	.	.	.	.	.	.	.	.	.	.	.	.	.	.	.	.	.	.	.	.	.	.	.
3370	2	1	2	3	7	1	1	2	3	.	.	.	.	.	.	.	.	.	.	.	.	.	.	.	.	.	.	.	.	.	.	.	.	.	.	.
3370	2	1	2	3	8	1	2	2	4	.	.	.	.	.	.	.	.	.	.	.	.	.	.	.	.	.	.	.	.	.	.	.	.	.	.	.
3370	2	1	2	4	9	1	1	1	1	.	.	.	.	.	.	.	.	.	.	.	.	.	.	.	.	.	.	.	.	.	.	.	.	.	.	.
3370	2	1	2	4	10	1	2	1	2	.	.	.	.	.	.	.	.	.	.	.	.	.	.	.	.	.	.	.	.	.	.	.	.	.	.	.
3370	2	1	2	4	11	1	1	2	3	.	.	.	.	.	.	.	.	.	.	.	.	.	.	.	.	.	.	.	.	.	.	.	.	.	.	.
3370	2	1	2	4	12	1	2	2	4	.	.	.	.	.	.	.	.	.	.	.	.	.	.	.	.	.	.	.	.	.	.	.	.	.	.	.
3370	2	1	2	6	13	1	1	1	1	-0.020	0.156	0.109	0.012	0.010	0.166	.	-0.040	0.275	0.526	0.001	0.001	0.527	0.631	0.001	1.141	48.294	1.205	0.636	0.001	1.133	48.547	-0.567	-0.039	2	.	.
3370	2	1	2	6	14	1	2	1	2	-0.166	0.179	0.040	0.010	0.046	0.225	.	-0.284	0.306	0.526	0.001	0.001	0.527	0.631	0.001	1.141	48.294	1.205	0.636	0.001	1.133	48.547	-0.567	-0.039	2	.	.
3370	2	1	2	6	15	1	1	2	3	0.246	0.153	0.056	0.008	0.010	0.163	.	0.421	0.262	0.526	0.001	0.001	0.527	0.631	0.001	1.141	48.294	1.205	0.636	0.001	1.133	48.547	-0.567	-0.039	2	.	.
3370	2	1	2	6	16	1	2	2	4	0.399	0.133	0.054	0.008	0.000	0.133	.	0.682	0.227	0.526	0.001	0.001	0.527	0.631	0.001	1.141	48.294	1.205	0.636	0.001	1.133	48.547	-0.567	-0.039	2	.	.
3370	2	1	2	7	17	1	1	1	1	-0.011	0.135	0.179	0.017	0.009	0.134	.	0.057	0.247	0.526	0.001	0.001	0.527	0.631	0.001	1.141	48.294	1.205	0.636	0.001	1.133	48.547	-0.567	-0.039	2	.	.
3370	2	1	2	7	18	1	2	1	2	0.107	0.127	0.089	0.011	0.001	0.128	.	0.220	0.246	0.526	0.001	0.001	0.527	0.631	0.001	1.141	48.294	1.205	0.636	0.001	1.133	48.547	-0.567	-0.039	2	.	.
3370	2	1	2	7	19	1	1	2	3	.	.	.	.	.	.	.	.	.	.	.	.	.	.	.	.	.	.	.	.	.	.	.	.	.	.	.
3370	2	1	2	7	20	1	2	2	4	.	.	.	.	.	.	.	.	.	.	.	.	.	.	.	.	.	.	.	.	.	.	.	.	.	.	.
3370	2	1	2	8	21	1	1	1	1	0.005	0.125	0.319	0.039	0.013	0.138	.	0.013	0.241	0.526	0.001	0.001	0.527	0.631	0.001	1.141	48.294	1.205	0.636	0.001	1.133	48.547	-0.567	-0.039	2	.	.
3370	2	1	2	8	22	1	2	1	2	0.025	0.133	0.152	0.018	0.018	0.151	.	0.059	0.263	0.526	0.001	0.001	0.527	0.631	0.001	1.141	48.294	1.205	0.636	0.001	1.133	48.547	-0.567	-0.039	2	.	.
3370	2	1	2	8	23	1	1	2	3	0.333	0.121	0.296	0.033	0.010	0.136	.	0.481	0.240	0.526	0.001	0.001	0.527	0.631	0.001	1.141	48.294	1.205	0.636	0.001	1.133	48.547	-0.567	-0.039	2	.	.
3370	2	1	2	8	24	1	2	2	4	0.354	0.135	0.194	0.019	0.004	0.134	.	0.519	0.247	0.526	0.001	0.001	0.527	0.631	0.001	1.141	48.294	1.205	0.636	0.001	1.133	48.547	-0.567	-0.039	2	.	.
3370	2	1	2	10	25	1	1	1	1	.	.	.	.	.	.	.	.	.	.	.	.	.	.	.	.	.	.	.	.	.	.	.	.	.	.	.
3370	2	1	2	10	26	1	2	1	2	.	.	.	.	.	.	.	.	.	.	.	.	.	.	.	.	.	.	.	.	.	.	.	.	.	.	.
3370	2	1	2	10	27	1	1	2	3	0.225	0.126	0.327	0.025	0.010	0.140	.	0.416	0.241	0.526	0.001	0.001	0.527	0.631	0.001	1.141	48.294	1.205	0.636	0.001	1.133	48.547	-0.567	-0.039	2	.	.
3370	2	1	2	10	28	1	2	2	4	0.290	0.123	0.192	0.022	0.007	0.127	.	0.504	0.224	0.526	0.001	0.001	0.527	0.631	0.001	1.141	48.294	1.205	0.636	0.001	1.133	48.547	-0.567	-0.039	2	.	.
3370	2	1	2	11	29	1	1	1	1	0.000	0.137	0.274	0.025	0.013	0.150	.	0.001	0.260	0.526	0.001	0.001	0.527	0.631	0.001	1.141	48.294	1.205	0.636	0.001	1.133	48.547	-0.567	-0.039	2	.	.
3370	2	1	2	11	30	1	2	1	2	0.024	0.125	0.120	0.011	0.013	0.138	.	0.034	0.239	0.526	0.001	0.001	0.527	0.631	0.001	1.141	48.294	1.205	0.636	0.001	1.133	48.547	-0.567	-0.039	2	.	.
3370	2	1	2	11	31	1	1	2	3	0.279	0.141	0.271	0.022	0.010	0.152	.	0.521	0.270	0.526	0.001	0.001	0.527	0.631	0.001	1.141	48.294	1.205	0.636	0.001	1.133	48.547	-0.567	-0.039	2	.	.
3370	2	1	2	11	32	1	2	2	4	0.359	0.125	0.141	0.015	0.001	0.127	.	0.678	0.238	0.526	0.001	0.001	0.527	0.631	0.001	1.141	48.294	1.205	0.636	0.001	1.133	48.547	-0.567	-0.039	2	.	.
3370	2	1	2	2	33	3	3	3	5	.	.	.	.	.	.	.	.	.	.	.	.	.	.	.	.	.	.	.	.	.	.	.	.	.	.	.
3370	2	1	2	4	34	3	3	3	5	.	.	.	.	.	.	.	.	.	.	.	.	.	.	.	.	.	.	.	.	.	.	.	.	.	.	.
3370	2	1	2	3	35	3	3	3	5	0.000	0.180	0.437	0.014	0.000	0.180	0.113	0.001	0.865	0.209	0.001	0.001	0.524	0.620	0.001	1.181	64.368	1.065	0.615	0.001	1.172	64.895	-0.494	0.030	2	0.366	0.233
3370	2	1	2	6	36	3	3	3	5	0.015	0.167	0.121	0.014	0.000	0.167	.	0.062	0.792	0.209	0.001	0.001	0.524	0.620	0.001	1.181	64.368	1.065	0.615	0.001	1.172	64.895	-0.494	0.030	2	.	.
3370	2	1	2	7	37	3	3	3	5	.	.	.	.	.	.	.	.	.	.	.	.	.	.	.	.	.	.	.	.	.	.	.	.	.	.	.
3370	2	1	2	8	38	3	3	3	5	-0.050	0.140	0.139	0.014	0.000	0.140	.	-0.235	0.669	0.209	0.001	0.001	0.524	0.620	0.001	1.181	64.368	1.065	0.615	0.001	1.172	64.895	-0.494	0.030	2	.	.
3370	2	1	2	10	39	3	3	3	5	.	.	.	.	.	.	.	.	.	.	.	.	.	.	.	.	.	.	.	.	.	.	.	.	.	.	.
3370	2	1	2	11	40	3	3	3	5	0.017	0.127	0.234	0.010	0.000	0.127	.	0.069	0.610	0.209	0.001	0.001	0.524	0.620	0.001	1.181	64.368	1.065	0.615	0.001	1.172	64.895	-0.494	0.030	2	.	.
3370	2	1	3	2	1	1	1	1	1	.	.	.	.	.	.	.	.	.	.	.	.	.	.	.	.	.	.	.	.	.	.	.	.	.	.	.
3370	2	1	3	2	2	1	2	1	2	.	.	.	.	.	.	.	.	.	.	.	.	.	.	.	.	.	.	.	.	.	.	.	.	.	.	.
3370	2	1	3	2	3	1	1	2	3	.	.	.	.	.	.	.	.	.	.	.	.	.	.	.	.	.	.	.	.	.	.	.	.	.	.	.
3370	2	1	3	2	4	1	2	2	4	.	.	.	.	.	.	.	.	.	.	.	.	.	.	.	.	.	.	.	.	.	.	.	.	.	.	.
3370	2	1	3	3	5	1	1	1	1	-0.093	0.104	0.154	0.019	0.007	0.111	.	-0.207	0.214	0.519	0.001	0.001	0.591	0.309	0.006	0.745	5.723	1.245	0.173	0.007	0.771	4.688	-0.551	0.040	2	.	.
3370	2	1	3	3	6	1	2	1	2	0.082	0.098	0.093	0.013	0.006	0.104	.	0.223	0.198	0.519	0.001	0.001	0.591	0.309	0.006	0.745	5.723	1.245	0.173	0.007	0.771	4.688	-0.551	0.040	2	.	.
3370	2	1	3	3	7	1	1	2	3	0.059	0.108	0.175	0.023	0.006	0.114	.	0.112	0.221	0.519	0.001	0.001	0.591	0.309	0.006	0.745	5.723	1.245	0.173	0.007	0.771	4.688	-0.551	0.040	2	.	.
3370	2	1	3	3	8	1	2	2	4	0.195	0.103	0.045	0.008	0.035	0.138	.	0.386	0.207	0.519	0.001	0.001	0.591	0.309	0.006	0.745	5.723	1.245	0.173	0.007	0.771	4.688	-0.551	0.040	2	.	.
3370	2	1	3	4	9	1	1	1	1	-0.022	0.108	0.355	0.039	0.022	0.129	.	-0.048	0.212	0.519	0.006	0.001	0.591	0.309	0.006	0.745	5.723	1.245	0.173	0.007	0.771	4.688	-0.551	0.040	2	.	.
3370	2	1	3	4	10	1	2	1	2	0.006	0.105	0.204	0.023	0.017	0.122	.	0.007	0.204	0.519	0.006	0.001	0.591	0.309	0.006	0.745	5.723	1.245	0.173	0.007	0.771	4.688	-0.551	0.040	2	.	.
3370	2	1	3	4	11	1	1	2	3	0.187	0.126	0.324	0.041	0.012	0.138	.	0.358	0.244	0.519	0.006	0.001	0.591	0.309	0.006	0.745	5.723	1.245	0.173	0.007	0.771	4.688	-0.551	0.040	2	.	.
3370	2	1	3	4	12	1	2	2	4	0.305	0.114	0.251	0.030	0.035	0.149	.	0.577	0.218	0.519	0.006	0.001	0.591	0.309	0.006	0.745	5.723	1.245	0.173	0.007	0.771	4.688	-0.551	0.040	2	.	.
3370	2	1	3	6	13	1	1	1	1	-0.014	0.120	0.124	0.022	0.007	0.127	.	-0.051	0.233	0.519	0.002	0.001	0.591	0.309	0.006	0.745	5.723	1.245	0.173	0.007	0.771	4.688	-0.551	0.040	2	.	.
3370	2	1	3	6	14	1	2	1	2	-0.022	0.123	0.085	0.015	0.006	0.130	.	-0.043	0.238	0.519	0.002	0.001	0.591	0.309	0.006	0.745	5.723	1.245	0.173	0.007	0.771	4.688	-0.551	0.040	2	.	.
3370	2	1	3	6	15	1	1	2	3	0.255	0.116	0.119	0.019	0.010	0.126	.	0.484	0.226	0.519	0.002	0.001	0.591	0.309	0.006	0.745	5.723	1.245	0.173	0.007	0.771	4.688	-0.551	0.040	2	.	.
3370	2	1	3	6	16	1	2	2	4	0.257	0.111	0.077	0.017	0.013	0.125	.	0.492	0.217	0.519	0.002	0.001	0.591	0.309	0.006	0.745	5.723	1.245	0.173	0.007	0.771	4.688	-0.551	0.040	2	.	.
3370	2	1	3	7	17	1	1	1	1	-0.007	0.137	0.180	0.025	0.037	0.175	.	-0.015	0.267	0.519	0.008	0.001	0.591	0.309	0.006	0.745	5.723	1.245	0.173	0.007	0.771	4.688	-0.551	0.040	2	.	.
3370	2	1	3	7	18	1	2	1	2	0.033	0.126	0.088	0.012	0.026	0.153	.	0.054	0.241	0.519	0.008	0.001	0.591	0.309	0.006	0.745	5.723	1.245	0.173	0.007	0.771	4.688	-0.551	0.040	2	.	.
3370	2	1	3	7	19	1	1	2	3	.	.	.	.	.	.	.	.	.	.	.	.	.	.	.	.	.	.	.	.	.	.	.	.	.	.	.
3370	2	1	3	7	20	1	2	2	4	.	.	.	.	.	.	.	.	.	.	.	.	.	.	.	.	.	.	.	.	.	.	.	.	.	.	.
3370	2	1	3	8	21	1	1	1	1	.	.	.	.	.	.	.	.	.	.	.	.	.	.	.	.	.	.	.	.	.	.	.	.	.	.	.
3370	2	1	3	8	22	1	2	1	2	.	.	.	.	.	.	.	.	.	.	.	.	.	.	.	.	.	.	.	.	.	.	.	.	.	.	.
3370	2	1	3	8	23	1	1	2	3	.	.	.	.	.	.	.	.	.	.	.	.	.	.	.	.	.	.	.	.	.	.	.	.	.	.	.
3370	2	1	3	8	24	1	2	2	4	.	.	.	.	.	.	.	.	.	.	.	.	.	.	.	.	.	.	.	.	.	.	.	.	.	.	.
3370	2	1	3	10	25	1	1	1	1	.	.	.	.	.	.	.	.	.	.	.	.	.	.	.	.	.	.	.	.	.	.	.	.	.	.	.
3370	2	1	3	10	26	1	2	1	2	.	.	.	.	.	.	.	.	.	.	.	.	.	.	.	.	.	.	.	.	.	.	.	.	.	.	.
3370	2	1	3	10	27	1	1	2	3	0.200	0.155	0.513	0.079	0.012	0.167	.	0.379	0.298	0.519	0.038	0.006	0.591	0.309	0.006	0.745	5.723	1.245	0.173	0.007	0.771	4.688	-0.551	0.040	2	.	.

3370	2	1	3	10	28	1	2	2	4	0.301	0.143	0.296	0.044	0.036	0.179	.	0.564	0.277	0.519	0.038	0.006	0.591	0.309	0.006	0.745	5.723	1.245	0.173	0.007	0.771	4.688	-0.551	0.040	2	.	.
3370	2	1	3	11	29	1	1	1	1	0.000	0.127	0.138	0.023	0.006	0.133	.	0.001	0.246	0.519	0.005	0.001	0.591	0.309	0.006	0.745	5.723	1.245	0.173	0.007	0.771	4.688	-0.551	0.040	2	.	.
3370	2	1	3	11	30	1	2	1	2	-0.038	0.124	0.084	0.012	0.007	0.131	.	-0.081	0.236	0.519	0.005	0.001	0.591	0.309	0.006	0.745	5.723	1.245	0.173	0.007	0.771	4.688	-0.551	0.040	2	.	.
3370	2	1	3	11	31	1	1	2	3	0.180	0.162	0.152	0.027	0.010	0.172	.	0.342	0.314	0.519	0.005	0.001	0.591	0.309	0.006	0.745	5.723	1.245	0.173	0.007	0.771	4.688	-0.551	0.040	2	.	.
3370	2	1	3	11	32	1	2	2	4	0.341	0.127	0.062	0.010	0.055	0.181	.	0.638	0.240	0.519	0.005	0.001	0.591	0.309	0.006	0.745	5.723	1.245	0.173	0.007	0.771	4.688	-0.551	0.040	2	.	.
3370	2	1	3	2	33	3	3	3	5	.	.	.	.	.	.	.	.	.	.	.	.	.	.	.	.	.	.	.	.	.	.	.	.	.	.	.
3370	2	1	3	3	34	3	3	3	5	0.000	0.163	0.137	0.021	0.000	0.163	0.648	0.001	0.638	0.258	0.001	0.001	0.438	0.294	0.006	0.294	1.071	0.832	0.165	0.007	0.313	1.081	-0.544	-0.106	1	0.657	0.379
3370	2	1	3	4	35	3	3	3	5	0.045	0.141	0.311	0.043	0.000	0.141	.	0.176	0.557	0.258	0.005	0.002	0.438	0.294	0.006	0.294	1.071	0.832	0.165	0.007	0.313	1.081	-0.544	-0.106	1	.	.
3370	2	1	3	6	36	3	3	3	5	0.008	0.169	0.107	0.014	0.000	0.169	.	0.027	0.676	0.258	0.004	0.002	0.438	0.294	0.006	0.294	1.071	0.832	0.165	0.007	0.313	1.081	-0.544	-0.106	1	.	.
3370	2	1	3	7	37	3	3	3	5	.	.	.	.	.	.	.	.	.	.	.	.	.	.	.	.	.	.	.	.	.	.	.	.	.	.	.
3370	2	1	3	8	38	3	3	3	5	.	.	.	.	.	.	.	.	.	.	.	.	.	.	.	.	.	.	.	.	.	.	.	.	.	.	.
3370	2	1	3	10	39	3	3	3	5	.	.	.	.	.	.	.	.	.	.	.	.	.	.	.	.	.	.	.	.	.	.	.	.	.	.	.
3370	2	1	3	11	40	3	3	3	5	0.034	0.167	0.033	0.005	0.000	0.167	.	0.146	0.647	0.258	0.003	0.001	0.438	0.294	0.006	0.294	1.071	0.832	0.165	0.007	0.313	1.081	-0.544	-0.106	1	.	.
3370	2	1	4	2	1	1	1	1	1	.	.	.	.	.	.	.	.	.	.	.	.	.	.	.	.	.	.	.	.	.	.	.	.	.	.	.
3370	2	1	4	2	2	1	2	1	2	.	.	.	.	.	.	.	.	.	.	.	.	.	.	.	.	.	.	.	.	.	.	.	.	.	.	.
3370	2	1	4	2	3	1	1	2	3	.	.	.	.	.	.	.	.	.	.	.	.	.	.	.	.	.	.	.	.	.	.	.	.	.	.	.
3370	2	1	4	2	4	1	2	2	4	.	.	.	.	.	.	.	.	.	.	.	.	.	.	.	.	.	.	.	.	.	.	.	.	.	.	.
3370	2	1	4	3	5	1	1	1	1	.	.	.	.	.	.	.	.	.	.	.	.	.	.	.	.	.	.	.	.	.	.	.	.	.	.	.
3370	2	1	4	3	6	1	2	1	2	.	.	.	.	.	.	.	.	.	.	.	.	.	.	.	.	.	.	.	.	.	.	.	.	.	.	.
3370	2	1	4	3	7	1	1	2	3	.	.	.	.	.	.	.	.	.	.	.	.	.	.	.	.	.	.	.	.	.	.	.	.	.	.	.
3370	2	1	4	3	8	1	2	2	4	.	.	.	.	.	.	.	.	.	.	.	.	.	.	.	.	.	.	.	.	.	.	.	.	.	.	.
3370	2	1	4	4	9	1	1	1	1	.	.	.	.	.	.	.	.	.	.	.	.	.	.	.	.	.	.	.	.	.	.	.	.	.	.	.
3370	2	1	4	4	10	1	2	1	2	.	.	.	.	.	.	.	.	.	.	.	.	.	.	.	.	.	.	.	.	.	.	.	.	.	.	.
3370	2	1	4	4	11	1	1	2	3	.	.	.	.	.	.	.	.	.	.	.	.	.	.	.	.	.	.	.	.	.	.	.	.	.	.	.
3370	2	1	4	4	12	1	2	2	4	.	.	.	.	.	.	.	.	.	.	.	.	.	.	.	.	.	.	.	.	.	.	.	.	.	.	.
3370	2	1	4	6	13	1	1	1	1	.	.	.	.	.	.	.	.	.	.	.	.	.	.	.	.	.	.	.	.	.	.	.	.	.	.	.
3370	2	1	4	6	14	1	2	1	2	.	.	.	.	.	.	.	.	.	.	.	.	.	.	.	.	.	.	.	.	.	.	.	.	.	.	.
3370	2	1	4	6	15	1	1	2	3	.	.	.	.	.	.	.	.	.	.	.	.	.	.	.	.	.	.	.	.	.	.	.	.	.	.	.
3370	2	1	4	6	16	1	2	2	4	.	.	.	.	.	.	.	.	.	.	.	.	.	.	.	.	.	.	.	.	.	.	.	.	.	.	.
3370	2	1	4	7	17	1	1	1	1	.	.	.	.	.	.	.	.	.	.	.	.	.	.	.	.	.	.	.	.	.	.	.	.	.	.	.
3370	2	1	4	7	18	1	2	1	2	.	.	.	.	.	.	.	.	.	.	.	.	.	.	.	.	.	.	.	.	.	.	.	.	.	.	.
3370	2	1	4	7	19	1	1	2	3	.	.	.	.	.	.	.	.	.	.	.	.	.	.	.	.	.	.	.	.	.	.	.	.	.	.	.
3370	2	1	4	7	20	1	2	2	4	.	.	.	.	.	.	.	.	.	.	.	.	.	.	.	.	.	.	.	.	.	.	.	.	.	.	.
3370	2	1	4	8	21	1	1	1	1	.	.	.	.	.	.	.	.	.	.	.	.	.	.	.	.	.	.	.	.	.	.	.	.	.	.	.
3370	2	1	4	8	22	1	2	1	2	.	.	.	.	.	.	.	.	.	.	.	.	.	.	.	.	.	.	.	.	.	.	.	.	.	.	.
3370	2	1	4	8	23	1	1	2	3	.	.	.	.	.	.	.	.	.	.	.	.	.	.	.	.	.	.	.	.	.	.	.	.	.	.	.
3370	2	1	4	8	24	1	2	2	4	.	.	.	.	.	.	.	.	.	.	.	.	.	.	.	.	.	.	.	.	.	.	.	.	.	.	.
3370	2	1	4	10	25	1	1	1	1	.	.	.	.	.	.	.	.	.	.	.	.	.	.	.	.	.	.	.	.	.	.	.	.	.	.	.
3370	2	1	4	10	26	1	2	1	2	.	.	.	.	.	.	.	.	.	.	.	.	.	.	.	.	.	.	.	.	.	.	.	.	.	.	.
3370	2	1	4	10	27	1	1	2	3	.	.	.	.	.	.	.	.	.	.	.	.	.	.	.	.	.	.	.	.	.	.	.	.	.	.	.
3370	2	1	4	10	28	1	2	2	4	.	.	.	.	.	.	.	.	.	.	.	.	.	.	.	.	.	.	.	.	.	.	.	.	.	.	.
3370	2	1	4	11	29	1	1	1	1	.	.	.	.	.	.	.	.	.	.	.	.	.	.	.	.	.	.	.	.	.	.	.	.	.	.	.
3370	2	1	4	11	30	1	2	1	2	.	.	.	.	.	.	.	.	.	.	.	.	.	.	.	.	.	.	.	.	.	.	.	.	.	.	.
3370	2	1	4	11	31	1	1	2	3	.	.	.	.	.	.	.	.	.	.	.	.	.	.	.	.	.	.	.	.	.	.	.	.	.	.	.
3370	2	1	4	11	32	1	2	2	4	.	.	.	.	.	.	.	.	.	.	.	.	.	.	.	.	.	.	.	.	.	.	.	.	.	.	.
3370	2	1	4	2	33	3	3	3	5	.	.	.	.	.	.	.	.	.	.	.	.	.	.	.	.	.	.	.	.	.	.	.	.	.	.	.
3370	2	1	4	3	34	3	3	3	5	.	.	.	.	.	.	.	.	.	.	.	.	.	.	.	.	.	.	.	.	.	.	.	.	.	.	.
3370	2	1	4	4	35	3	3	3	5	.	.	.	.	.	.	.	.	.	.	.	.	.	.	.	.	.	.	.	.	.	.	.	.	.	.	.
3370	2	1	4	6	36	3	3	3	5	.	.	.	.	.	.	.	.	.	.	.	.	.	.	.	.	.	.	.	.	.	.	.	.	.	.	.
3370	2	1	4	7	37	3	3	3	5	.	.	.	.	.	.	.	.	.	.	.	.	.	.	.	.	.	.	.	.	.	.	.	.	.	.	.
3370	2	1	4	8	38	3	3	3	5	.	.	.	.	.	.	.	.	.	.	.	.	.	.	.	.	.	.	.	.	.	.	.	.	.	.	.
3370	2	1	4	10	39	3	3	3	5	.	.	.	.	.	.	.	.	.	.	.	.	.	.	.	.	.	.	.	.	.	.	.	.	.	.	.
3370	2	1	4	11	40	3	3	3	5	.	.	.	.	.	.	.	.	.	.	.	.	.	.	.	.	.	.	.	.	.	.	.	.	.	.	.
3362	2	2	1	2	1	1	1	1	1	.	.	.	.	.	.	.	.	.	.	.	.	.	.	.	.	.	.	.	.	.	.	.	.	.	.	.
3362	2	2	1	2	2	1	2	1	2	.	.	.	.	.	.	.	.	.	.	.	.	.	.	.	.	.	.	.	.	.	.	.	.	.	.	.
3362	2	2	1	2	3	1	1	2	3	.	.	.	.	.	.	.	.	.	.	.	.	.	.	.	.	.	.	.	.	.	.	.	.	.	.	.
3362	2	2	1	2	4	1	2	2	4	.	.	.	.	.	.	.	.	.	.	.	.	.	.	.	.	.	.	.	.	.	.	.	.	.	.	.
3362	2	2	1	3	5	1	1	1	1	-0.021	0.115	0.437	0.051	0.015	0.130	.	-0.047	0.245	0.476	0.008	0.002	0.464	0.306	0.004	0.057	1.117	1.123	0.389	0.004	0.041	0.743	-1.155	-0.681	2	.	.
3362	2	2	1	3	6	1	2	1	2	-0.069	0.118	0.218	0.027	0.011	0.130	.	-0.139	0.250	0.476	0.008	0.002	0.464	0.306	0.004	0.057	1.117	1.123	0.389	0.004	0.041	0.743	-1.155	-0.681	2	.	.
3362	2	2	1	3	7	1	1	2	3	0.170	0.128	0.335	0.042	0.014	0.143	.	0.363	0.276	0.476	0.008	0.002	0.464	0.306	0.004	0.057	1.117	1.123	0.389	0.004	0.041	0.743	-1.155	-0.681	2	.	.
3362	2	2	1	3	8	1	2	2	4	0.251	0.113	0.155	0.018	0.028	0.143	.	0.534	0.240	0.476	0.008	0.002	0.464	0.306	0.004	0.057	1.117	1.123	0.389	0.004	0.041	0.743	-1.155	-0.681	2	.	.
3362	2	2	1	4	9	1	1	1	1	-0.229	0.114	0.156	0.023	0.005	0.119	.	-0.515	0.246	0.476	0.002	0.001	0.464	0.306	0.004	0.057	1.117	1.123	0.389	0.004	0.041	0.743	-1.155	-0.681	2	.	.
3362	2	2	1	4	10	1	2	1	2	-0.234	0.109	0.100	0.015	0.005	0.114	.	-0.492	0.239	0.476	0.002	0.001	0.464	0.306	0.004	0.057	1.117	1.123	0.389	0.004	0.041	0.743	-1.155	-0.681	2	.	.
3362	2	2	1	4	11	1	1	2	3	-0.045	0.139	0.090	0.014	0.010	0.149	.	-0.096	0.304	0.476	0.002	0.001	0.464	0.306	0.004	0.057	1.117	1.123	0.389	0.004	0.041	0.743	-1.155	-0.681	2	.	.
3362	2	2	1	4	12	1	2	2	4	0.117	0.139	0.075	0.010	0.023	0.162	.	0.263	0.307	0.476	0.002	0.001	0.464	0.306	0.004	0.057	1.117	1.123	0.389	0.004	0.041	0.743	-1.155	-0.681	2	.	.
3362	2	2	1	6	13	1	1	1	1	-0.021	0.112	0.148	0.017	0.009	0.121	.	-0.022	0.255	0.476	0.001	0.001	0.464	0.306	0.004	0.057	1.117	1.123	0.389	0.004	0.041	0.743	-1.155	-0.681	2	.	.
3362	2	2	1	6	14	1	2	1	2	0.054	0.124	0.047	0.005	0.017	0.140	.	0.144	0.275	0.476	0.001	0.001	0.464	0.306	0.004	0.057	1.117	1.123	0.389	0.004	0.041	0.743	-1.155	-0.681	2	.	.
3362	2	2	1	6	15	1	1	2	3	0.255	0.116	0.167	0.018	0.017	0.132	.	0.566	0.251	0.476	0.001	0.001	0.464	0.306	0.004	0.057	1.117	1.123	0.389	0.004	0.041	0.743	-1.155	-0.681	2	.	.
3362	2	2	1	6	16	1	2	2	4	0.289	0.114	0.080	0.011	0.022	0.136	.	0.665	0.242	0.476	0.001	0.001	0.464	0.306	0.004	0.057	1.117	1.123	0.389	0.004	0.041	0.743	-1.155	-0.681	2	.	.
3362	2	2	1	7	17	1	1	1	1	0.088	0.112	0.065	0.009	0.005	0.117	.	0.231	0.295	0.476	0.001	0.001	0.464	0.306	0.004	0.057	1.117	1.123	0.389	0.004	0.041	0.743	-1.155	-0.681	2	.	.
3362	2	2	1	7	18	1	2	1	2	0.097	0.134	0.030	0.004	0.005	0.139	.	0.265	0.351	0.476	0.001	0.001	0.464	0.306	0.004	0.057	1.117	1.123	0.389	0.004	0.041	0.743	-1.155	-0.681	2	.	.
3362	2	2	1	7	19	1	1	2	3	.	.	.	.	.	.	.	.	.	.	.	.	.	.	.	.	.	.	.	.	.	.	.	.	.	.	.
3362	2	2	1	7	20	1	2	2	4	.	.	.	.	.	.	.	.	.	.	.	.	.	.	.	.	.	.	.	.	.	.	.	.	.	.	.
3362	2	2	1	8	21	1	1	1	1	-0.075	0.114	0.397	0.044	0.011	0.124	.	-0.151	0.241	0.476	0.020	0.003	0.464	0.306	0.004	0.057	1.117	1.123	0.389	0.004	0.041	0.743	-1.155	-0.681	2	.	.
3362	2	2	1	8	22	1	2	1	2	-0.057	0.115	0.271	0.029	0.011	0.126	.	-0.107	0.243	0.476	0.016	0.003	0.464	0.306	0.004	0.057	1.117	1.123	0.389	0.004	0.041	0.743	-1.155	-0.681	2	.	.
3362	2	2	1	8	23	1	1	2	3	0.128	0.132	0.536	0.069	0.016	0.148	.	0.285	0.279	0.476	0.020	0.003	0.464	0.306	0.004	0.057	1.117	1.123	0.389	0.004	0.041	0.743	-1.155	-0.681	2	.	.
3362	2	2	1	8	24	1	2	2	4	0.245	0.130	0.360	0.045	0.025	0.154	.	0.522	0.272	0.476	0.020	0.003	0.464	0.306	0.004	0.057	1.117	1.123	0.389	0.004	0.041	0.743	-1.155	-0.681	2	.	.
3362	2	2	1	10	25	1	1	1	1	.	.	.	.	.	.	.	.	.	.	.	.	.	.	.	.	.	.	.	.	.	.	.	.	.	.	.
3362	2	2	1	10	26	1	2	1	2	.	.	.	.	.	.	.	.	.	.	.	.	.	.	.	.	.	.	.	.	.	.	.	.	.	.	.
3362	2	2	1	10	27	1	1	2	3	0.277	0.155	0.068	0.008	0.020	0.175	.	0.605	0.350	0.476	0.001	0.001	0.464	0.306	0.004	0.057	1.117	1.123	0.389	0.004	0.041	0.743	-1.155	-0.681	2	.	.
3362	2	2	1	10	28	1	2	2	4	0.138	0.140	0.044	0.006	0.008	0.149	.	0.299	0.310	0.476	0.001	0.001	0.464	0.306	0.004	0.057	1.117	1.123	0.389	0.004	0.041	0.743	-1.155	-0.681	2	.	.
3362	2	2	1	11	29	1	1	1	1	0.000	0.119	0.467	0.054	0.009	0.128	.	0.001	0.256	0.476	0.016	0.002	0.464	0.306	0.004	0.057	1.117	1.123	0.389	0.004	0.041	0.743	-1.155	-0.681	2	.	.
3362	2	2	1	11	30	1	2	1	2	-0.082	0.109	0.266	0.029	0.009	0.118	.	-0.182	0.233	0.476	0.016	0.002	0.464	0.306	0.004	0.057	1.117	1.123	0.389	0.004	0.041	0.743	-1.155	-0.681	2	.	.
3362	2	2	1	11	31	1	1	2	3	0.154	0.134	0.384	0.051	0.017	0.151	.	0.323	0.290	0.476	0.016	0.002	0.464	0.306	0.004	0.057	1.117	1.123	0.389	0.004	0.041	0.743	-1.155	-0.681	2	.	.
3362	2	2	1	11	32	1	2	2	4	0.248	0.132	0.288	0.044	0.022	0.151	.	0.534	0.277	0.476	0.016	0.002	0.464	0.306	0.004	0.057	1.117	1.123	0.389	0.004	0.041	0.743	-1.155	-0.681	2	.	.
3362	2	2	1	2	33	3	3	3	5	.	.	.	.	.	.	.	.	.	.	.	.	.	.	.	.	.	.	.	.	.	.	.	.	.	.	.
3362	2	2	1	3	34	3	3	3	5	0.000	0.184	0.284	0.051	0.014	0.198	2.046	0.001	0.720	0.256	0.006	0.001	0.488	0.252	0.004	0.127	7.687	1.087	0.390	0.004	0.111	7.704	-0.744	-0.106	2	0.807	0.542
3362	2	2	1	4	35	3	3	3	5	0.001	0.185	0.159	0.017	0.000	0.185	.	0.007	0.717	0.256	0.002	0.001	0.488	0.252	0.004	0.127	7.687	1.087	0.390	0.004	0.111	7.704	-0.744	-0.106	2	.	.
3362	2	2	1	6	36	3	3	3	5	0.023	0.136	0.213	0.015	0.000	0.136	.	0.066	0.541	0.256	0.001	0.001	0.488	0.252	0.004	0.127	7.687	1.087	0.390	0.004	0.111	7.704	-0.744	-0.106	2	.	.
3362	2	2	1	7	37	3	3	3	5	.	.	.	.	.	.	.	.	.	.	.	.	.	.	.	.	.	.	.	.	.	.	.	.	.	.	.
3362	2	2	1	8	38	3	3	3	5	-0.002	0.137	0.082	0.010	0.000	0.137	.	-0.019	0.546	0.256	0.026	0.005	0.488	0.252	0.004	0.127	7.687	1.087	0.390	0.004	0.111	7.704	-0.744	-0.106	2	.	.
3362	2	2	1	10	39	3	3	3	5	.	.	.	.	.	.	.	.	.	.	.	.	.	.	.	.	.	.	.	.	.	.	.	.	.	.	.
3362	2	2	1	11	40	3	3	3	5	-0.011	0.167	0.064	0.007	0.000	0.167	.	-0.049	0.653	0.256	0.018	0.003	0.488	0.252	0.004	0.127	7.687	1.087	0.390	0.004	0.111	7.704	-0.744	-0.106	2	.	.
3362	2	2	2	2	1	1	1	1	1	.	.	.	.	.	.	.	.	.	.	.	.	.	.	.	.	.	.	.	.	.	.	.	.	.	.	.
3362	2	2	2	2	2	1	2	1	2	.	.	.	.	.	.	.	.	.	.	.	.	.	.	.	.	.	.	.	.	.	.	.	.	.	.	.
3362	2	2	2	2	3	1	1	2	3	.	.	.	.	.	.	.	.	.	.	.	.	.	.	.	.	.	.	.	.	.	.	.	.	.	.	.
3362	2	2	2	2	4	1	2	2	4	.	.	.	.	.	.	.	.	.	.	.	.	.	.	.	.	.	.	.	.	.	.	.	.	.	.	.
3362	2	2	2	3	5	1	1	1	1	-0.119	0.117	0.415	0.052	0.012	0.129	.	-0.236	0.226	0.497	0.007	0.007	0.538	0.214	0.001	0.021	25.559	1.095	0.189	0.001	0.021	25.733	-0.679	-0.142	2	.	.
3362	2	2	2	3	6	1	2	1	2	-0.037	0.112	0.212	0.023	0.019	0.131	.	-0.071	0.214	0.497	0.007	0.007	0.538	0.214	0.001	0.021	25.559	1.095	0.189	0.001	0.021	25.733	-0.679	-0.142	2	.	.
3362	2	2	2	3	7	1	1	2	3	0.069	0.138	0.500	0.065	0.011	0.148	.	0.127	0.278	0.497	0.007	0.007	0.538	0.214	0.001	0.021	25.559	1.095	0.189	0.001	0.021	25.733	-0.679	-0.142	2	.	.
3362	2	2	2	3	8	1	2	2	4	0.163	0.134	0.233	0.031	0.015	0.149	.	0.304	0.272	0.497	0.007	0.007	0.538	0.214	0.001	0.021	25.559	1.095	0.189	0.001	0.021	25.733	-0.679	-0.142	2	.	.
3362	2	2	2	4	9	1	1	1	1	-0.084	0.142	0.207	0.024	0.031	0.173	.	-0.186	0.285	0.497	0.001	0.001	0.538	0.214	0.001	0.021	25.559	1.095	0.189	0.001	0.021	25.733	-0.679	-0.142	2	.	.
3362	2	2	2	4	10	1	2	1	2	-0.151	0.130	0.107	0.014	0.015	0.145	.	-0.329	0.266	0.497	0.001	0.001	0.538	0.214	0.001	0.021	25.559	1.095	0.189	0.001	0.021	25.733	-0.679	-0.142	2	.	.
3362	2	2	2	4	11	1	1	2	3	0.129	0.153	0.225	0.025	0.037	0.196	.	0.213	0.333	0.497	0.001	0.001	0.538	0.214	0.001	0.021	25.559	1.095	0.189	0.001	0.021	25.733	-0.679	-0.142	2	.	.
3362	2	2	2	4	12	1	2	2	4	0.240	0.140	0.073	0.009	0.035	0.175	.	0.494	0.295	0.497	0.001	0.001	0.538	0.214	0.001	0.021	25.559	1.095	0.189	0.001	0.021	25.733	-0.679	-0.142	2	.	.
3362	2	2	2	6	13	1	1	1	1	-0.157	0.141	0.114	0.014	0.008	0.149	.	-0.337	0.296	0.497	0.001	0.001	0.538	0.214	0.001	0.021	25.559	1.095	0.189	0.001	0.021	25.733	-0.679	-0.142	2	.	.
3362	2	2	2	6	14	1	2	1	2	-0.108	0.118	0.051	0.006	0.011	0.129	.	-0.228	0.249	0.497	0.001	0.001	0.538	0.214	0.001	0.021	25.559	1.095	0.189	0.001	0.021	25.733	-0.679	-0.142	2	.	.
3362	2	2	2	6	15	1	1	2	3	0.108	0.159	0.197	0.021	0.027	0.185	.	0.233	0.341	0.497	0.001	0.001	0.538	0.214	0.001	0.021	25.559	1.095	0.189	0.001	0.021	25.733	-0.679	-0.142	2	.	.
3362	2	2	2	6	16	1	2	2	4	0.106	0.171	0.103	0.014	0.010	0.181	.	0.161	0.338	0.497	0.001	0.001	0.538	0.214	0.001	0.021	25.559	1.095	0.189	0.001	0.021	25.733	-0.679	-0.142	2	.	.
3362	2	2	2	7	17	1	1	1	1	-0.070	0.134	0.207	0.024	0.016	0.150	.	-0.131	0.276	0.497	0.001	0.001	0.538	0.214	0.001	0.021	25.559	1.095	0.189	0.001	0.021	25.733	-0.679	-0.142	2	.	.
3362	2	2	2	7	18	1	2	1	2	-0.031	0.116	0.121	0.013	0.025	0.142	.	-0.071	0.231	0.497	0.001	0.001	0.538	0.214	0.001	0.021	25.559	1.095	0.189	0.001	0.021	25.733	-0.679	-0.142	2	.	.
3362	2	2	2	7	19	1	1	2	3	.	.	.	.	.	.	.	.	.	.	.	.	.	.	.	.	.	.	.	.	.	.	.	.	.	.	.
3362	2	2	2	7	20	1	2	2	4	.	.	.	.	.	.	.	.	.	.	.	.	.	.	.	.	.	.	.	.	.	.	.	.	.	.	.
3362	2	2	2	8	21	1	1	1	1	-0.028	0.130	0.378	0.046	0.013	0.142	.	-0.057	0.263	0.497	0.009	0.003	0.538	0.214	0.001	0.021	25.559	1.095	0.189	0.001	0.021	25.733	-0.679	-0.142	2	.	.
3362	2	2	2	8	22	1	2	1	2	-0.067	0.128	0.256	0.030	0.012	0.141	.	-0.131	0.259	0.497	0.009	0.003	0.538	0.214	0.001	0.021	25.559	1.095	0.189	0.001	0.021	25.733	-0.679	-0.142	2	.	.
3362	2	2	2	8	23	1	1	2	3	0.208	0.160	0.301	0.037	0.017	0.178	.	0.418	0.326	0.497	0.009	0.003	0.538	0.214	0.001	0.021	25.559	1.095	0.189	0.001	0.021	25.733	-0.679	-0.142	2	.	.
3362	2	2	2	8	24	1	2	2	4	0.241	0.140	0.197	0.023	0.011	0.151	.	0.494	0.281	0.497	0.009	0.003	0.538	0.214	0.001	0.021	25.559	1.095	0.189	0.001	0.021	25.733	-0.679	-0.142	2	.	.
3362	2	2	2	10	25	1	1	1	1	.	.	.	.	.	.	.	.	.	.	.	.	.	.	.	.	.	.	.	.	.	.	.	.	.	.	.
3362	2	2	2	10	26	1	2	1	2	.	.	.	.	.	.	.	.	.	.	.	.	.	.	.	.	.	.	.	.	.	.	.	.	.	.	.
3362	2	2	2	10	27	1	1	2	3	0.160	0.135	0.386	0.052	0.014	0.149	.	0.316	0.276	0.497	0.005	0.001	0.538	0.214	0.001	0.021	25.559	1.095	0.189	0.001	0.021	25.733	-0.679	-0.142	2	.	.
3362	2	2	2	10	28	1	2	2	4	0.143	0.131	0.257	0.033	0.010	0.141	.	0.284	0.265	0.497	0.005	0.001	0.538	0.214	0.001	0.021	25.559	1.095	0.189	0.001	0.021	25.733	-0.679	-0.142	2	.	.
3362	2	2	2	11	29	1	1	1	1	0.000	0.119	0.405	0.044	0.010	0.130	0.072	0.001	0.243	0.497	0.001	0.001	0.538	0.214	0.001	0.021	25.559	1.095	0.189	0.001	0.021	25.733	-0.679	-0.142	2	.	.
3362	2	2	2	11	30	1	2	1	2	-0.108	0.135	0.171	0.019	0.007	0.142	.	-0.225	0.276	0.497	0.001	0.001	0.538	0.214	0.001	0.021	25.559	1.095	0.189	0.001	0.021	25.733	-0.679	-0.142	2	.	.
3362	2	2	2	11	31	1	1	2	3	0.124	0.134	0.424	0.047	0.006	0.140	.	0.249	0.270	0.497	0.001	0.001	0.538	0.214	0.001	0.021	25.559	1.095	0.189	0.001	0.021	25.733	-0.679	-0.142	2	.	.
3362	2	2	2	11	32	1	2	2	4	0.252	0.140	0.144	0.017	0.031	0.171	.	0.507	0.281	0.497	0.001	0.001	0.538	0.214	0.001	0.021	25.559	1.095	0.189	0.001	0.021	25.733	-0.679	-0.142	2	.	.
3362	2	2	2	2	33	3	3	3	5	.	.	.	.	.	.	.	.	.	.	.	.	.	.	.	.	.	.	.	.	.	.	.	.	.	.	.
3362	2	2	2	3	34	3	3	3	5	0.000	0.216	0.318	0.049	0.000	0.216	1.315	0.001	0.837	0.262	0.007	0.001	0.470	0.239	0.001	0.008	23.559	0.978	0.209	0.002	0.011	23.699	-0.930	-0.474	2	0.954	0.620
3362	2	2	2	4	35	3	3	3	5	0.092	0.176	0.215	0.024	0.000	0.176	.	0.360	0.678	0.262	0.001	0.001	0.470	0.239	0.001	0.008	23.559	0.978	0.209	0.002	0.011	23.699	-0.930	-0.474	2	.	.
3362	2	2	2	6	36	3	3	3	5	0.089	0.152	0.057	0.006	0.000	0.152	.	0.359	0.595	0.262	0.001	0.001	0.470	0.239	0.001	0.008	23.559	0.978	0.209	0.002	0.011	23.699	-0.930	-0.474	2	.	.
3362	2	2	2	7	37	3	3	3	5	.	.	.	.	.	.	.	.	.	.	.	.	.	.	.	.	.	.	.	.	.	.	.	.	.	.	.
3362	2	2	2	8	38	3	3	3	5	0.075	0.154	0.303	0.034	0.000	0.154	.	0.282	0.614	0.262	0.009	0.003	0.470	0.239	0.001	0.008	23.559	0.978	0.209	0.002	0.011	23.699	-0.930	-0.474	2	.	.
3362	2	2	2	10	39	3	3	3	5	.	.	.	.	.	.	.	.	.	.	.	.	.	.	.	.	.	.	.	.	.	.	.	.	.	.	.
3362	2	2	2	11	40	3	3	3	5	0.005	0.142	0.085	0.009	0.000	0.142	.	0.009	0.580	0.262	0.001	0.001	0.470	0.239	0.001	0.008	23.559	0.978	0.209	0.002	0.011	23.699	-0.930	-0.474	2	.	.
3362	2	2	3	2	1	1	1	1	1	.	.	.	.	.	.	.	.	.	.	.	.	.	.	.	.	.	.	.	.	.	.	.	.	.	.	.
3362	2	2	3	2	2	1	2	1	2	.	.	.	.	.	.	.	.	.	.	.	.	.	.	.	.	.	.	.	.	.	.	.	.	.	.	.
3362	2	2	3	2	3	1	1	2	3	.	.	.	.	.	.	.	.	.	.	.	.	.	.	.	.	.	.	.	.	.	.	.	.	.	.	.
3362	2	2	3	2	4	1	2	2	4	.	.	.	.	.	.	.	.	.	.	.	.	.	.	.	.	.	.	.	.	.	.	.	.	.	.	.
3362	2	2	3	3	5	1	1	1	1	.	.	.	.	.	.	.	.	.	.	.	.	.	.	.	.	.	.	.	.	.	.	.	.	.	.	.
3362	2	2	3	3	6	1	2	1	2	.	.	.	.	.	.	.	.	.	.	.	.	.	.	.	.	.	.	.	.	.	.	.	.	.	.	.

3362	2	2	3	3	7	1	1	2	3	.	.	.	.	.	.	.	.	.	.	.	.	.	.	.	.	.	.	.	.	.	.	.	.	.	.	.
3362	2	2	3	3	8	1	2	2	4	.	.	.	.	.	.	.	.	.	.	.	.	.	.	.	.	.	.	.	.	.	.	.	.	.	.	.
3362	2	2	3	4	9	1	1	1	1	-0.066	0.126	0.236	0.027	0.006	0.132	.	-0.136	0.249	0.509	0.056	0.014	0.609	0.079	0.001	0.042	2.596	1.234	0.061	0.001	0.035	2.618	-1.016	-0.417	2	.	.
3362	2	2	3	4	10	1	2	1	2	-0.033	0.130	0.148	0.017	0.005	0.135	.	-0.072	0.259	0.509	0.060	0.015	0.609	0.079	0.001	0.042	2.596	1.234	0.061	0.001	0.035	2.618	-1.016	-0.417	2	.	.
3362	2	2	3	4	11	1	1	2	3	0.180	0.141	0.206	0.028	0.008	0.149	.	0.348	0.278	0.509	0.060	0.015	0.609	0.079	0.001	0.042	2.596	1.234	0.061	0.001	0.035	2.618	-1.016	-0.417	2	.	.
3362	2	2	3	4	12	1	2	2	4	0.268	0.127	0.134	0.017	0.012	0.139	.	0.532	0.251	0.509	0.060	0.015	0.609	0.079	0.001	0.042	2.596	1.234	0.061	0.001	0.035	2.618	-1.016	-0.417	2	.	.
3362	2	2	3	6	13	1	1	1	1	-0.079	0.128	0.252	0.027	0.009	0.137	.	-0.159	0.255	0.509	0.002	0.001	0.609	0.079	0.001	0.042	2.596	1.234	0.061	0.001	0.035	2.618	-1.016	-0.417	2	.	.
3362	2	2	3	6	14	1	2	1	2	-0.031	0.137	0.090	0.010	0.009	0.145	.	-0.054	0.269	0.509	0.002	0.001	0.609	0.079	0.001	0.042	2.596	1.234	0.061	0.001	0.035	2.618	-1.016	-0.417	2	.	.
3362	2	2	3	6	15	1	1	2	3	0.177	0.142	0.213	0.025	0.010	0.152	.	0.350	0.282	0.509	0.002	0.001	0.609	0.079	0.001	0.042	2.596	1.234	0.061	0.001	0.035	2.618	-1.016	-0.417	2	.	.
3362	2	2	3	6	16	1	2	2	4	0.251	0.139	0.123	0.013	0.032	0.171	.	0.504	0.277	0.509	0.002	0.001	0.609	0.079	0.001	0.042	2.596	1.234	0.061	0.001	0.035	2.618	-1.016	-0.417	2	.	.
3362	2	2	3	7	17	1	1	1	1	-0.058	0.134	0.163	0.019	0.024	0.158	.	-0.124	0.263	0.509	0.003	0.001	0.609	0.079	0.001	0.042	2.596	1.234	0.061	0.001	0.035	2.618	-1.016	-0.417	2	.	.
3362	2	2	3	7	18	1	2	1	2	-0.014	0.115	0.060	0.007	0.009	0.124	.	-0.027	0.221	0.509	0.003	0.001	0.609	0.079	0.001	0.042	2.596	1.234	0.061	0.001	0.035	2.618	-1.016	-0.417	2	.	.
3362	2	2	3	7	19	1	1	2	3	.	.	.	.	.	.	.	.	.	.	.	.	.	.	.	.	.	.	.	.	.	.	.	.	.	.	.
3362	2	2	3	7	20	1	2	2	4	.	.	.	.	.	.	.	.	.	.	.	.	.	.	.	.	.	.	.	.	.	.	.	.	.	.	.
3362	2	2	3	8	21	1	1	1	1	.	.	.	.	.	.	.	.	.	.	.	.	.	.	.	.	.	.	.	.	.	.	.	.	.	.	.
3362	2	2	3	8	22	1	2	1	2	.	.	.	.	.	.	.	.	.	.	.	.	.	.	.	.	.	.	.	.	.	.	.	.	.	.	.
3362	2	2	3	8	23	1	1	2	3	.	.	.	.	.	.	.	.	.	.	.	.	.	.	.	.	.	.	.	.	.	.	.	.	.	.	.
3362	2	2	3	8	24	1	2	2	4	.	.	.	.	.	.	.	.	.	.	.	.	.	.	.	.	.	.	.	.	.	.	.	.	.	.	.
3362	2	2	3	10	25	1	1	1	1	.	.	.	.	.	.	.	.	.	.	.	.	.	.	.	.	.	.	.	.	.	.	.	.	.	.	.
3362	2	2	3	10	26	1	2	1	2	.	.	.	.	.	.	.	.	.	.	.	.	.	.	.	.	.	.	.	.	.	.	.	.	.	.	.
3362	2	2	3	10	27	1	1	2	3	0.158	0.154	0.225	0.028	0.009	0.163	.	0.310	0.304	0.509	0.041	0.010	0.609	0.079	0.001	0.042	2.596	1.234	0.061	0.001	0.035	2.618	-1.016	-0.417	2	.	.
3362	2	2	3	10	28	1	2	2	4	0.229	0.134	0.151	0.018	0.009	0.143	.	0.453	0.266	0.509	0.041	0.010	0.609	0.079	0.001	0.042	2.596	1.234	0.061	0.001	0.035	2.618	-1.016	-0.417	2	.	.
3362	2	2	3	11	29	1	1	1	1	0.000	0.119	0.084	0.009	0.011	0.131	.	0.001	0.235	0.509	0.039	0.009	0.609	0.079	0.001	0.042	2.596	1.234	0.061	0.001	0.035	2.618	-1.016	-0.417	2	1.330	.
3362	2	2	3	11	30	1	2	1	2	-0.059	0.123	0.049	0.005	0.006	0.129	.	-0.116	0.244	0.509	0.002	0.001	0.609	0.079	0.001	0.042	2.596	1.234	0.061	0.001	0.035	2.618	-1.016	-0.417	2	.	.
3362	2	2	3	11	31	1	1	2	3	0.197	0.120	0.088	0.010	0.007	0.128	.	0.390	0.239	0.509	0.002	0.001	0.609	0.079	0.001	0.042	2.596	1.234	0.061	0.001	0.035	2.618	-1.016	-0.417	2	.	.
3362	2	2	3	11	32	1	2	2	4	0.251	0.127	0.064	0.007	0.033	0.160	.	0.493	0.252	0.509	0.002	0.001	0.609	0.079	0.001	0.042	2.596	1.234	0.061	0.001	0.035	2.618	-1.016	-0.417	2	.	.
3362	2	2	3	2	33	3	3	3	5	-0.117	0.133	0.013	0.002	0.000	0.133	.	-0.305	0.346	0.255	0.001	0.001	0.477	0.221	0.001	0.201	11.037	1.037	0.238	0.001	0.144	11.197	-0.562	-0.106	2	.	.
3362	2	2	3	3	34	3	3	3	5	0.000	0.232	0.193	0.033	0.000	0.232	0.524	0.001	0.940	0.255	0.001	0.001	0.477	0.221	0.001	0.201	11.037	1.037	0.238	0.001	0.144	11.197	-0.562	-0.106	2	0.857	0.515
3362	2	2	3	4	35	3	3	3	5	-0.019	0.166	0.256	0.043	0.000	0.166	.	-0.048	0.697	0.255	0.057	0.014	0.477	0.221	0.001	0.201	11.037	1.037	0.238	0.001	0.144	11.197	-0.562	-0.106	2	.	.
3362	2	2	3	6	36	3	3	3	5	-0.016	0.171	0.313	0.035	0.000	0.171	.	-0.008	0.710	0.255	0.005	0.002	0.477	0.221	0.001	0.201	11.037	1.037	0.238	0.001	0.144	11.197	-0.562	-0.106	2	.	.
3362	2	2	3	7	37	3	3	3	5	.	.	.	.	.	.	.	.	.	.	.	.	.	.	.	.	.	.	.	.	.	.	.	.	.	.	.
3362	2	2	3	8	38	3	3	3	5	.	.	.	.	.	.	.	.	.	.	.	.	.	.	.	.	.	.	.	.	.	.	.	.	.	.	.
3362	2	2	3	10	39	3	3	3	5	.	.	.	.	.	.	.	.	.	.	.	.	.	.	.	.	.	.	.	.	.	.	.	.	.	.	.
3362	2	2	3	11	40	3	3	3	5	-0.013	0.148	0.077	0.008	0.000	0.148	.	0.038	0.584	0.255	0.010	0.002	0.477	0.221	0.001	0.201	11.037	1.037	0.238	0.001	0.144	11.197	-0.562	-0.106	2	.	.
3362	2	2	4	2	1	1	1	1	1	0.078	0.108	0.273	0.030	0.003	0.111	.	0.137	0.229	0.477	0.001	0.001	0.553	1.007	0.002	10.543	32.771	1.231	0.869	0.002	10.373	34.008	-0.984	-0.428	2	.	.
3362	2	2	4	2	2	1	2	1	2	0.024	0.134	0.160	0.018	0.005	0.139	.	0.051	0.282	0.477	0.001	0.001	0.553	1.007	0.002	10.543	32.771	1.231	0.869	0.002	10.373	34.008	-0.984	-0.428	2	.	.
3362	2	2	4	2	3	1	1	2	3	0.307	0.128	0.358	0.040	0.010	0.138	.	0.635	0.273	0.477	0.001	0.001	0.553	1.007	0.002	10.543	32.771	1.231	0.869	0.002	10.373	34.008	-0.984	-0.428	2	.	.
3362	2	2	4	2	4	1	2	2	4	0.448	0.134	0.065	0.007	0.022	0.156	.	0.935	0.273	0.477	0.001	0.001	0.553	1.007	0.002	10.543	32.771	1.231	0.869	0.002	10.373	34.008	-0.984	-0.428	2	.	.
3362	2	2	4	3	5	1	1	1	1	-0.047	0.119	0.383	0.041	0.009	0.128	.	-0.098	0.255	0.477	0.021	0.003	0.553	1.007	0.002	10.543	32.771	1.231	0.869	0.002	10.373	34.008	-0.984	-0.428	2	.	.
3362	2	2	4	3	6	1	2	1	2	-0.061	0.127	0.246	0.030	0.010	0.137	.	-0.148	0.271	0.477	0.021	0.003	0.553	1.007	0.002	10.543	32.771	1.231	0.869	0.002	10.373	34.008	-0.984	-0.428	2	.	.
3362	2	2	4	3	7	1	1	2	3	0.155	0.128	0.356	0.031	0.006	0.134	.	0.320	0.275	0.477	0.021	0.003	0.553	1.007	0.002	10.543	32.771	1.231	0.869	0.002	10.373	34.008	-0.984	-0.428	2	.	.
3362	2	2	4	3	8	1	2	2	4	0.110	0.141	0.172	0.024	0.010	0.151	.	0.199	0.303	0.477	0.021	0.003	0.553	1.007	0.002	10.543	32.771	1.231	0.869	0.002	10.373	34.008	-0.984	-0.428	2	.	.
3362	2	2	4	4	9	1	1	1	1	-0.124	0.128	0.335	0.042	0.007	0.135	.	-0.273	0.277	0.477	0.019	0.003	0.553	1.007	0.002	10.543	32.771	1.231	0.869	0.002	10.373	34.008	-0.984	-0.428	2	.	.
3362	2	2	4	4	10	1	2	1	2	-0.146	0.115	0.163	0.019	0.006	0.122	.	-0.337	0.252	0.477	0.019	0.003	0.553	1.007	0.002	10.543	32.771	1.231	0.869	0.002	10.373	34.008	-0.984	-0.428	2	.	.
3362	2	2	4	4	11	1	1	2	3	0.119	0.127	0.257	0.033	0.010	0.136	.	0.244	0.276	0.477	0.019	0.003	0.553	1.007	0.002	10.543	32.771	1.231	0.869	0.002	10.373	34.008	-0.984	-0.428	2	.	.
3362	2	2	4	4	12	1	2	2	4	0.011	0.148	0.161	0.024	0.008	0.155	.	-0.002	0.326	0.477	0.019	0.003	0.553	1.007	0.002	10.543	32.771	1.231	0.869	0.002	10.373	34.008	-0.984	-0.428	2	.	.
3362	2	2	4	6	13	1	1	1	1	-0.220	0.144	0.112	0.012	0.005	0.149	.	-0.514	0.327	0.477	0.001	0.001	0.553	1.007	0.002	10.543	32.771	1.231	0.869	0.002	10.373	34.008	-0.984	-0.428	2	.	.
3362	2	2	4	6	14	1	2	1	2	-0.071	0.136	0.039	0.004	0.005	0.141	.	-0.173	0.311	0.477	0.001	0.001	0.553	1.007	0.002	10.543	32.771	1.231	0.869	0.002	10.373	34.008	-0.984	-0.428	2	.	.
3362	2	2	4	6	15	1	1	2	3	-0.136	0.149	0.126	0.013	0.005	0.154	.	-0.328	0.336	0.477	0.001	0.001	0.553	1.007	0.002	10.543	32.771	1.231	0.869	0.002	10.373	34.008	-0.984	-0.428	2	.	.
3362	2	2	4	6	16	1	2	2	4	0.162	0.120	0.059	0.006	0.005	0.125	.	0.339	0.271	0.477	0.001	0.001	0.553	1.007	0.002	10.543	32.771	1.231	0.869	0.002	10.373	34.008	-0.984	-0.428	2	.	.
3362	2	2	4	7	17	1	1	1	1	-0.070	0.142	0.364	0.041	0.005	0.147	.	-0.148	0.300	0.477	0.021	0.003	0.553	1.007	0.002	10.543	32.771	1.231	0.869	0.002	10.373	34.008	-0.984	-0.428	2	.	.
3362	2	2	4	7	18	1	2	1	2	-0.014	0.136	0.147	0.016	0.008	0.144	.	-0.039	0.287	0.477	0.021	0.003	0.553	1.007	0.002	10.543	32.771	1.231	0.869	0.002	10.373	34.008	-0.984	-0.428	2	.	.
3362	2	2	4	7	19	1	1	2	3	.	.	.	.	.	.	.	.	.	.	.	.	.	.	.	.	.	.	.	.	.	.	.	.	.	.	.
3362	2	2	4	7	20	1	2	2	4	.	.	.	.	.	.	.	.	.	.	.	.	.	.	.	.	.	.	.	.	.	.	.	.	.	.	.
3362	2	2	4	8	21	1	1	1	1	-0.055	0.129	0.353	0.044	0.008	0.136	.	-0.128	0.272	0.477	0.014	0.002	0.553	1.007	0.002	10.543	32.771	1.231	0.869	0.002	10.373	34.008	-0.984	-0.428	2	.	.
3362	2	2	4	8	22	1	2	1	2	-0.110	0.130	0.232	0.031	0.005	0.135	.	-0.240	0.273	0.477	0.014	0.002	0.553	1.007	0.002	10.543	32.771	1.231	0.869	0.002	10.373	34.008	-0.984	-0.428	2	.	.
3362	2	2	4	8	23	1	1	2	3	0.167	0.137	0.304	0.036	0.006	0.142	.	0.338	0.287	0.477	0.014	0.002	0.553	1.007	0.002	10.543	32.771	1.231	0.869	0.002	10.373	34.008	-0.984	-0.428	2	.	.
3362	2	2	4	8	24	1	2	2	4	0.217	0.129	0.194	0.026	0.007	0.136	.	0.448	0.272	0.477	0.014	0.002	0.553	1.007	0.002	10.543	32.771	1.231	0.869	0.002	10.373	34.008	-0.984	-0.428	2	.	.
3362	2	2	4	10	25	1	1	1	1	.	.	.	.	.	.	.	.	.	.	.	.	.	.	.	.	.	.	.	.	.	.	.	.	.	.	.
3362	2	2	4	10	26	1	2	1	2	.	.	.	.	.	.	.	.	.	.	.	.	.	.	.	.	.	.	.	.	.	.	.	.	.	.	.
3362	2	2	4	10	27	1	1	2	3	0.159	0.157	0.350	0.049	0.008	0.165	.	0.326	0.329	0.477	0.043	0.007	0.553	1.007	0.002	10.543	32.771	1.231	0.869	0.002	10.373	34.008	-0.984	-0.428	2	.	.
3362	2	2	4	10	28	1	2	2	4	0.176	0.130	0.255	0.032	0.016	0.146	.	0.373	0.275	0.477	0.043	0.007	0.553	1.007	0.002	10.543	32.771	1.231	0.869	0.002	10.373	34.008	-0.984	-0.428	2	.	.
3362	2	2	4	11	29	1	1	1	1	0.000	0.114	0.306	0.037	0.006	0.119	.	0.001	0.240	0.477	0.001	0.001	0.553	1.007	0.002	10.543	32.771	1.231	0.869	0.002	10.373	34.008	-0.984	-0.428	2	.	.
3362	2	2	4	11	30	1	2	1	2	-0.138	0.116	0.239	0.029	0.008	0.124	.	-0.284	0.244	0.477	0.001	0.001	0.553	1.007	0.002	10.543	32.771	1.231	0.869	0.002	10.373	34.008	-0.984	-0.428	2	.	.
3362	2	2	4	11	31	1	1	2	3	0.151	0.124	0.272	0.034	0.005	0.129	.	0.314	0.257	0.477	0.001	0.001	0.553	1.007	0.002	10.543	32.771	1.231	0.869	0.002	10.373	34.008	-0.984	-0.428	2	.	.
3362	2	2	4	11	32	1	2	2	4	0.278	0.132	0.169	0.021	0.019	0.150	.	0.579	0.276	0.477	0.001	0.001	0.553	1.007	0.002	10.543	32.771	1.231	0.869	0.002	10.373	34.008	-0.984	-0.428	2	.	.
3362	2	2	4	2	33	3	3	3	5	-0.053	0.245	0.150	0.016	0.000	0.245	.	-0.216	1.012	0.234	0.001	0.001	0.532	1.674	0.002	10.750	162.229	1.092	1.724	0.002	10.272	163.694	-0.754	-0.213	2	.	.
3362	2	2	4	3	34	3	3	3	5	0.000	0.203	0.484	0.067	0.000	0.203	0.994	0.001	0.871	0.234	0.025	0.004	0.532	1.674	0.002	10.750	162.229	1.092	1.724	0.002	10.272	163.694	-0.754	-0.213	2	0.598	0.301
3362	2	2	4	4	35	3	3	3	5	0.020	0.177	0.116	0.017	0.000	0.177	.	0.083	0.757	0.234	0.014	0.002	0.532	1.674	0.002	10.750	162.229	1.092	1.724	0.002	10.272	163.694	-0.754	-0.213	2	.	.
3362	2	2	4	6	36	3	3	3	5	0.017	0.180	0.217	0.022	0.000	0.180	.	0.082	0.783	0.234	0.010	0.002	0.532	1.674	0.002	10.750	162.229	1.092	1.724	0.002	10.272	163.694	-0.754	-0.213	2	.	.
3362	2	2	4	7	37	3	3	3	5	.	.	.	.	.	.	.	.	.	.	.	.	.	.	.	.	.	.	.	.	.	.	.	.	.	.	.
3362	2	2	4	8	38	3	3	3	5	-0.008	0.164	0.166	0.020	0.000	0.164	.	-0.026	0.701	0.234	0.013	0.002	0.532	1.674	0.002	10.750	162.229	1.092	1.724	0.002	10.272	163.694	-0.754	-0.213	2	.	.
3362	2	2	4	10	39	3	3	3	5	.	.	.	.	.	.	.	.	.	.	.	.	.	.	.	.	.	.	.	.	.	.	.	.	.	.	.
3362	2	2	4	11	40	3	3	3	5	0.006	0.178	0.020	0.002	0.000	0.178	.	0.025	0.745	0.234	0.002	0.001	0.532	1.674	0.002	10.750	162.229	1.092	1.724	0.002	10.272	163.694	-0.754	-0.213	2	.	.
4032	1	1	1	2	1	1	1	1	1	-0.033	0.125	0.050	0.005	0.005	0.130	.	-0.068	0.250	0.510	0.001	0.001	0.517	0.107	0.001	0.076	0.021	1.043	0.110	0.004	0.072	0.040	-0.629	-0.094	2	.	.
4032	1	1	1	2	2	1	2	1	2	0.055	0.138	0.030	0.003	0.005	0.143	.	0.125	0.275	0.500	0.001	0.001	0.515	0.106	0.001	0.075	0.021	1.050	0.108	0.004	0.071	0.039	-0.677	-0.143	2	.	.
4032	1	1	1	2	3	1	1	2	3	0.164	0.163	0.047	0.005	0.008	0.171	.	0.327	0.324	0.500	0.001	0.001	0.515	0.106	0.001	0.075	0.021	1.050	0.108	0.004	0.071	0.039	-0.677	-0.143	2	.	.
4032	1	1	1	2	4	1	2	2	4	0.279	0.171	0.047	0.005	0.015	0.186	.	0.543	0.331	0.500	0.001	0.001	0.515	0.106	0.001	0.075	0.021	1.050	0.108	0.004	0.071	0.039	-0.677	-0.143	2	.	.
4032	1	1	1	3	5	1	1	1	1	-0.033	0.135	0.296	0.040	0.005	0.140	.	-0.063	0.275	0.500	0.006	0.002	0.515	0.106	0.001	0.075	0.021	1.050	0.108	0.004	0.071	0.039	-0.677	-0.143	2	.	.
4032	1	1	1	3	6	1	2	1	2	0.019	0.122	0.256	0.031	0.006	0.128	.	0.044	0.249	0.500	0.006	0.002	0.515	0.106	0.001	0.075	0.021	1.050	0.108	0.004	0.071	0.039	-0.677	-0.143	2	.	.
4032	1	1	1	3	7	1	1	2	3	0.247	0.136	0.272	0.037	0.006	0.142	.	0.513	0.278	0.500	0.006	0.002	0.515	0.106	0.001	0.075	0.021	1.050	0.108	0.004	0.071	0.039	-0.677	-0.143	2	.	.
4032	1	1	1	3	8	1	2	2	4	0.264	0.130	0.242	0.031	0.006	0.137	.	0.538	0.265	0.500	0.006	0.002	0.515	0.106	0.001	0.075	0.021	1.050	0.108	0.004	0.071	0.039	-0.677	-0.143	2	.	.
4032	1	1	1	4	9	1	1	1	1	-0.093	0.127	0.206	0.024	0.007	0.139	.	-0.186	0.265	0.500	0.009	0.003	0.515	0.106	0.001	0.075	0.021	1.050	0.108	0.004	0.071	0.039	-0.677	-0.143	2	.	.
4032	1	1	1	4	10	1	2	1	2	-0.012	0.119	0.067	0.007	0.009	0.129	.	-0.024	0.240	0.500	0.009	0.003	0.515	0.106	0.001	0.075	0.021	1.050	0.108	0.004	0.071	0.039	-0.677	-0.143	2	.	.
4032	1	1	1	4	11	1	1	2	3	0.168	0.133	0.155	0.019	0.009	0.140	.	0.351	0.262	0.500	0.009	0.003	0.515	0.106	0.001	0.075	0.021	1.050	0.108	0.004	0.071	0.039	-0.677	-0.143	2	.	.
4032	1	1	1	4	12	1	2	2	4	0.257	0.135	0.103	0.012	0.009	0.148	.	0.520	0.274	0.500	0.009	0.003	0.515	0.106	0.001	0.075	0.021	1.050	0.108	0.004	0.071	0.039	-0.677	-0.143	2	.	.
4032	1	1	1	6	13	1	1	1	1	-0.105	0.121	0.314	0.027	0.008	0.129	.	-0.217	0.241	0.500	0.005	0.002	0.515	0.106	0.001	0.075	0.021	1.050	0.108	0.004	0.071	0.039	-0.677	-0.143	2	.	.
4032	1	1	1	6	14	1	2	1	2	-0.039	0.122	0.126	0.013	0.010	0.132	.	-0.081	0.244	0.500	0.005	0.002	0.515	0.106	0.001	0.075	0.021	1.050	0.108	0.004	0.071	0.039	-0.677	-0.143	2	.	.
4032	1	1	1	6	15	1	1	2	3	0.154	0.124	0.263	0.028	0.008	0.132	.	0.305	0.247	0.500	0.005	0.002	0.515	0.106	0.001	0.075	0.021	1.050	0.108	0.004	0.071	0.039	-0.677	-0.143	2	.	.
4032	1	1	1	6	16	1	2	2	4	0.196	0.134	0.100	0.011	0.015	0.149	.	0.385	0.268	0.500	0.005	0.002	0.515	0.106	0.001	0.075	0.021	1.050	0.108	0.004	0.071	0.039	-0.677	-0.143	2	.	.
4032	1	1	1	7	17	1	1	1	1	-0.114	0.138	0.140	0.017	0.010	0.147	.	-0.229	0.276	0.500	0.006	0.002	0.515	0.106	0.001	0.075	0.021	1.050	0.108	0.004	0.071	0.039	-0.677	-0.143	2	.	.
4032	1	1	1	7	18	1	2	1	2	0.035	0.114	0.105	0.011	0.013	0.127	.	0.068	0.228	0.500	0.006	0.002	0.515	0.106	0.001	0.075	0.021	1.050	0.108	0.004	0.071	0.039	-0.677	-0.143	2	.	.
4032	1	1	1	7	19	1	1	2	3	.	.	.	.	.	.	.	.	.	.	.	.	.	.	.	.	.	.	.	.	.	.	.	.	.	.	.
4032	1	1	1	7	20	1	2	2	4	.	.	.	.	.	.	.	.	.	.	.	.	.	.	.	.	.	.	.	.	.	.	.	.	.	.	.
4032	1	1	1	8	21	1	1	1	1	0.026	0.122	0.127	0.014	0.007	0.130	.	0.055	0.245	0.500	0.003	0.001	0.515	0.106	0.001	0.075	0.021	1.050	0.108	0.004	0.071	0.039	-0.677	-0.143	2	.	.
4032	1	1	1	8	22	1	2	1	2	0.085	0.120	0.079	0.009	0.016	0.136	.	0.187	0.239	0.500	0.003	0.001	0.515	0.106	0.001	0.075	0.021	1.050	0.108	0.004	0.071	0.039	-0.677	-0.143	2	.	.
4032	1	1	1	8	23	1	1	2	3	0.250	0.128	0.101	0.011	0.008	0.136	.	0.497	0.253	0.500	0.003	0.001	0.515	0.106	0.001	0.075	0.021	1.050	0.108	0.004	0.071	0.039	-0.677	-0.143	2	.	.
4032	1	1	1	8	24	1	2	2	4	0.277	0.137	0.084	0.009	0.014	0.150	.	0.568	0.272	0.500	0.003	0.001	0.515	0.106	0.001	0.075	0.021	1.050	0.108	0.004	0.071	0.039	-0.677	-0.143	2	.	.
4032	1	1	1	10	25	1	1	1	1	.	.	.	.	.	.	.	.	.	.	.	.	.	.	.	.	.	.	.	.	.	.	.	.	.	.	.
4032	1	1	1	10	26	1	2	1	2	.	.	.	.	.	.	.	.	.	.	.	.	.	.	.	.	.	.	.	.	.	.	.	.	.	.	.
4032	1	1	1	10	27	1	1	2	3	0.224	0.149	0.363	0.053	0.008	0.157	.	0.449	0.299	0.500	0.021	0.003	0.515	0.106	0.001	0.075	0.021	1.050	0.108	0.004	0.071	0.039	-0.677	-0.143	2	.	.
4032	1	1	1	10	28	1	2	2	4	0.218	0.148	0.292	0.044	0.020	0.168	.	0.425	0.297	0.500	0.021	0.003	0.515	0.106	0.001	0.075	0.021	1.050	0.108	0.004	0.071	0.039	-0.677	-0.143	2	.	.
4032	1	1	1	11	29	1	1	1	1	0.000	0.121	0.066	0.007	0.005	0.126	.	0.001	0.243	0.500	0.001	0.001	0.515	0.106	0.001	0.075	0.021	1.050	0.108	0.004	0.071	0.039	-0.677	-0.143	2	.	.
4032	1	1	1	11	30	1	2	1	2	-0.043	0.121	0.042	0.005	0.005	0.126	.	-0.084	0.244	0.500	0.001	0.001	0.515	0.106	0.001	0.075	0.021	1.050	0.108	0.004	0.071	0.039	-0.677	-0.143	2	.	.
4032	1	1	1	11	31	1	1	2	3	0.175	0.129	0.064	0.007	0.005	0.134	.	0.347	0.257	0.500	0.001	0.001	0.515	0.106	0.001	0.075	0.021	1.050	0.108	0.004	0.071	0.039	-0.677	-0.143	2	.	.
4032	1	1	1	11	32	1	2	2	4	0.275	0.139	0.039	0.004	0.020	0.160	.	0.547	0.274	0.500	0.001	0.001	0.515	0.106	0.001	0.075	0.021	1.050	0.108	0.004	0.071	0.039	-0.677	-0.143	2	.	.
4032	1	1	1	2	33	3	3	3	5	-0.011	0.143	0.032	0.004	0.000	0.143	.	-0.042	0.570	0.273	0.001	0.001	0.576	0.108	0.001	0.003	0.040	1.081	0.087	0.001	0.015	0.021	-0.528	0.057	1	.	.
4032	1	1	1	3	34	3	3	3	5	0.000	0.195	0.227	0.043	0.000	0.195	0.426	0.001	0.728	0.273	0.006	0.002	0.576	0.108	0.001	0.003	0.040	1.081	0.087	0.001	0.015	0.021	-0.528	0.057	1	0.601	0.334
4032	1	1	1	4	35	3	3	3	5	-0.041	0.172	0.282	0.026	0.000	0.172	.	-0.136	0.645	0.273	0.009	0.003	0.576	0.108	0.001	0.003	0.040	1.081	0.087	0.001	0.015	0.021	-0.528	0.057	1	.	.
4032	1	1	1	6	36	3	3	3	5	-0.134	0.187	0.092	0.009	0.000	0.187	.	-0.520	0.753	0.273	0.005	0.002	0.576	0.108	0.001	0.003	0.040	1.081	0.087	0.001	0.015	0.021	-0.528	0.057	1	.	.
4032	1	1	1	7	37	3	3	3	5	.	.	.	.	.	.	.	.	.	.	.	.	.	.	.	.	.	.	.	.	.	.	.	.	.	.	.
4032	1	1	1	8	38	3	3	3	5	0.000	0.202	0.077	0.008	0.000	0.202	.	-0.029	0.769	0.273	0.003	0.001	0.576	0.108	0.001	0.003	0.040	1.081	0.087	0.001	0.015	0.021	-0.528	0.057	1	.	.
4032	1	1	1	10	39	3	3	3	5	.	.	.	.	.	.	.	.	.	.	.	.	.	.	.	.	.	.	.	.	.	.	.	.	.	.	.
4032	1	1	1	11	40	3	3	3	5	-0.043	0.195	0.027	0.003	0.000	0.195	.	-0.169	0.781	0.273	0.001	0.001	0.576	0.108	0.001	0.003	0.040	1.081	0.087	0.001	0.015	0.021	-0.528	0.057	1	.	.
4032	1	1	2	2	1	1	1	1	1	0.206	0.181	0.503	0.054	0.019	0.200	.	0.348	0.307	0.560	0.001	0.001	0.544	0.215	0.001	0.006	0.544	1.180	0.228	0.001	0.044	0.711	-0.809	-0.265	2	.	.
4032	1	1	2	2	2	1	2	1	2	0.407	0.128	0.085	0.015	0.008	0.136	.	0.689	0.218	0.560	0.001	0.001	0.544	0.215	0.001	0.006	0.544	1.180	0.228	0.001	0.044	0.711	-0.809	-0.265	2	.	.
4032	1	1	2	2	3	1	1	2	3	0.630	0.167	0.451	0.051	0.030	0.197	.	1.067	0.282	0.560	0.001	0.001	0.544	0.215	0.001	0.006	0.544	1.180	0.228	0.001	0.044	0.711	-0.809	-0.265	2	.	.
4032	1	1	2	2	4	1	2	2	4	0.480	0.089	0.021	0.003	0.005	0.094	.	0.805	0.149	0.560	0.001	0.001	0.544	0.215	0.001	0.006	0.544	1.180	0.228	0.001	0.044	0.711	-0.809	-0.265	2	.	.
4032	1	1	2	3	5	1	1	1	1	-0.028	0.130	0.234	0.027	0.005	0.135	.	-0.060	0.235	0.560	0.001	0.001	0.544	0.215	0.001	0.006	0.544	1.180	0.228	0.001	0.044	0.711	-0.809	-0.265	2	.	.
4032	1	1	2	3	6	1	2	1	2	0.023	0.113	0.108	0.011	0.006	0.119	.	0.084	0.207	0.560	0.001	0.001	0.544	0.215	0.001	0.006	0.544	1.180	0.228	0.001	0.044	0.711	-0.809	-0.265	2	.	.
4032	1	1	2	3	7	1	1	2	3	0.299	0.154	0.316	0.042	0.019	0.172	.	0.527	0.275	0.560	0.001	0.001	0.544	0.215	0.001	0.006	0.544	1.180	0.228	0.001	0.044	0.711	-0.809	-0.265	2	.	.
4032	1	1	2	3	8	1	2	2	4	0.139	0.154	0.172	0.023	0.015	0.169	.	0.253	0.279	0.560	0.001	0.001	0.544	0.215	0.001	0.006	0.544	1.180	0.228	0.001	0.044	0.711	-0.809	-0.265	2	.	.
4032	1	1	2	4	9	1	1	1	1	-0.030	0.133	0.172	0.022	0.006	0.139	.	-0.052	0.228	0.560	0.007	0.002	0.544	0.215	0.001	0.006	0.544	1.180	0.228	0.001	0.044	0.711	-0.809	-0.265	2	.	.
4032	1	1	2	4	10	1	2	1	2	0.055	0.126	0.069	0.009	0.007	0.133	.	0.092	0.216	0.560	0.007	0.002	0.544	0.215	0.001	0.006	0.544	1.180	0.228	0.001	0.044	0.711	-0.809	-0.265	2	.	.
4032	1	1	2	4	11	1	1	2	3	0.217	0.149	0.208	0.027	0.005	0.154	.	0.374	0.255	0.560	0.007	0.002	0.544	0.215	0.001	0.006	0.544	1.180	0.228	0.001	0.044	0.711	-0.809	-0.265	2	.	.
4032	1	1	2	4	12	1	2	2	4	0.313	0.167	0.082	0.009	0.039	0.206	.	0.527	0.291	0.560	0.007	0.002	0.544	0.215	0.001	0.006	0.544	1.180	0.228	0.001	0.044	0.711	-0.809	-0.265	2	.	.
4032	1	1	2	6	13	1	1	1	1	0.014	0.131	0.121	0.013	0.005	0.136	.	0.025	0.221	0.560	0.007	0.002	0.544	0.215	0.001	0.006	0.544	1.180	0.228	0.001	0.044	0.711	-0.809	-0.265	2	.	.
4032	1	1	2	6	14	1	2	1	2	0.147	0.122	0.070	0.008	0.005	0.127	.	0.251	0.206	0.560	0.007	0.002	0.544	0.215	0.001	0.006	0.544	1.180	0.228	0.001	0.044	0.711	-0.809	-0.265	2	.	.
4032	1	1	2	6	15	1	1	2	3	0.343	0.123	0.083	0.009	0.005	0.128	.	0.582	0.207	0.560	0.007	0.002	0.544	0.215	0.001	0.006	0.544	1.180	0.228	0.001	0.044	0.711	-0.809	-0.265	2	.	.
4032	1	1	2	6	16	1	2	2	4	0.362	0.119	0.076	0.008	0.005	0.124	.	0.614	0.201	0.560	0.007	0.002	0.544	0.215	0.001	0.006	0.544	1.180	0.228	0.001	0.044	0.711	-0.809	-0.265	2	.	.
4032	1	1	2	7	17	1	1	1	1	0.004	0.151	0.471	0.073	0.006	0.156	.	0.010	0.272	0.560	0.015	0.003	0.544	0.215	0.001	0.006	0.544	1.180	0.228	0.001	0.044	0.711	-0.809	-0.265	2	.	.
4032	1	1	2	7	18	1	2	1	2	-0.059	0.133	0.331	0.041	0.005	0.138	.	-0.108	0.240	0.560	0.015	0.003	0.544	0.215	0.001	0.006	0.544	1.180	0.228	0.001	0.044	0.711	-0.809	-0.265	2	.	.
4032	1	1	2	7	19	1	1	2	3	.	.	.	.	.	.	.	.	.	.	.	.	.	.	.	.	.	.	.	.	.	.	.	.	.	.	.
4032	1	1	2	7	20	1	2	2	4	.	.	.	.	.	.	.	.	.	.	.	.	.	.	.	.	.	.	.	.	.	.	.	.	.	.	.
4032	1	1	2	8	21	1	1	1	1	-0.037	0.141	0.141	0.015	0.005	0.146	.	-0.062	0.239	0.560	0.003	0.002	0.544	0.215	0.001	0.006	0.544	1.180	0.228	0.001	0.044	0.711	-0.809	-0.265	2	.	.
4032	1	1	2	8	22	1	2	1	2	-0.009	0.120	0.055	0.006	0.005	0.125	.	-0.013	0.203	0.560	0.003	0.002	0.544	0.215	0.001	0.006	0.544	1.180	0.228	0.001	0.044	0.711	-0.809	-0.265	2	.	.
4032	1	1	2	8	23	1	1	2	3	0.404	0.171	0.081	0.008	0.019	0.190	.	0.714	0.302	0.560	0.003	0.002	0.544	0.215	0.001	0.006	0.544	1.180	0.228	0.001	0.044	0.711	-0.809	-0.265	2	.	.
4032	1	1	2	8	24	1	2	2	4	0.175	0.205	0.050	0.005	0.007	0.211	.	0.309	0.365	0.560	0.003	0.002	0.544	0.215	0.001	0.006	0.544	1.180	0.228	0.001	0.044	0.711	-0.809	-0.265	2	.	.
4032	1	1	2	10	25	1	1	1	1	.	.	.	.	.	.	.	.	.	.	.	.	.	.	.	.	.	.	.	.	.	.	.	.	.	.	.

4032	1	1	2	10	26	1	2	1	2	.	.	.	.	.	.	.	.	.	.	.	.	.	.	.	.	.	.	.	.	.	.	.	.	.	.	.
4032	1	1	2	10	27	1	1	2	3	0.353	0.150	0.173	0.024	0.013	0.163	.	0.609	0.266	0.560	0.027	0.005	0.544	0.215	0.001	0.006	0.544	1.180	0.228	0.001	0.044	0.711	-0.809	-0.265	2	.	.
4032	1	1	2	10	28	1	2	2	4	0.259	0.156	0.119	0.015	0.007	0.163	.	0.452	0.277	0.560	0.027	0.005	0.544	0.215	0.001	0.006	0.544	1.180	0.228	0.001	0.044	0.711	-0.809	-0.265	2	.	.
4032	1	1	2	11	29	1	1	1	1	0.000	0.125	0.223	0.025	0.007	0.132	.	0.001	0.223	0.560	0.005	0.002	0.544	0.215	0.001	0.006	0.544	1.180	0.228	0.001	0.044	0.711	-0.809	-0.265	2	.	.
4032	1	1	2	11	30	1	2	1	2	-0.032	0.123	0.132	0.015	0.006	0.129	.	-0.061	0.222	0.560	0.005	0.002	0.544	0.215	0.001	0.006	0.544	1.180	0.228	0.001	0.044	0.711	-0.809	-0.265	2	.	.
4032	1	1	2	11	31	1	1	2	3	0.327	0.153	0.217	0.029	0.018	0.172	.	0.575	0.275	0.560	0.005	0.002	0.544	0.215	0.001	0.006	0.544	1.180	0.228	0.001	0.044	0.711	-0.809	-0.265	2	.	.
4032	1	1	2	11	32	1	2	2	4	0.245	0.166	0.121	0.016	0.041	0.207	.	0.439	0.296	0.560	0.005	0.002	0.544	0.215	0.001	0.006	0.544	1.180	0.228	0.001	0.044	0.711	-0.809	-0.265	2	.	.
4032	1	1	2	2	33	3	3	3	5	.	.	.	.	.	.	.	.	.	.	.	.	.	.	.	.	.	.	.	.	.	.	.	.	.	.	.
4032	1	1	2	3	34	3	3	3	5	0.000	0.205	0.394	0.077	0.000	0.205	0.893	0.001	0.839	0.245	0.001	0.001	0.398	0.232	0.001	0.005	0.328	0.708	0.301	0.001	0.031	1.098	-0.843	-0.491	2	0.445	0.405
4032	1	1	2	4	35	3	3	3	5	0.005	0.167	0.405	0.052	0.000	0.167	.	0.030	0.684	0.245	0.007	0.002	0.398	0.232	0.001	0.005	0.328	0.708	0.301	0.001	0.031	1.098	-0.843	-0.491	2	.	.
4032	1	1	2	6	36	3	3	3	5	-0.005	0.178	0.074	0.008	0.000	0.178	.	-0.002	0.740	0.245	0.007	0.002	0.398	0.232	0.001	0.005	0.328	0.708	0.301	0.001	0.031	1.098	-0.843	-0.491	2	.	.
4032	1	1	2	7	37	3	3	3	5	.	.	.	.	.	.	.	.	.	.	.	.	.	.	.	.	.	.	.	.	.	.	.	.	.	.	.
4032	1	1	2	8	38	3	3	3	5	0.076	0.156	0.143	0.020	0.000	0.156	.	0.312	0.660	0.245	0.015	0.003	0.398	0.232	0.001	0.005	0.328	0.708	0.301	0.001	0.031	1.098	-0.843	-0.491	2	.	.
4032	1	1	2	10	39	3	3	3	5	.	.	.	.	.	.	.	.	.	.	.	.	.	.	.	.	.	.	.	.	.	.	.	.	.	.	.
4032	1	1	2	11	40	3	3	3	5	0.108	0.178	0.170	0.024	0.000	0.178	.	0.528	0.837	0.245	0.005	0.002	0.398	0.232	0.001	0.005	0.328	0.708	0.301	0.001	0.031	1.098	-0.843	-0.491	2	.	.
4032	1	1	3	2	1	1	1	1	1	0.037	0.153	0.234	0.026	0.005	0.158	.	0.098	0.250	0.615	0.036	0.012	0.465	0.199	0.002	0.415	2.519	0.879	0.302	0.002	0.448	2.248	-0.437	0.031	1	.	.
4032	1	1	3	2	2	1	2	1	2	0.069	0.158	0.135	0.015	0.005	0.163	.	0.137	0.258	0.615	0.036	0.012	0.465	0.199	0.002	0.415	2.519	0.879	0.302	0.002	0.448	2.248	-0.437	0.031	1	.	.
4032	1	1	3	2	3	1	1	2	3	0.351	0.177	0.296	0.033	0.006	0.183	.	0.601	0.290	0.615	0.036	0.012	0.465	0.199	0.002	0.415	2.519	0.879	0.302	0.002	0.448	2.248	-0.437	0.031	1	.	.
4032	1	1	3	2	4	1	2	2	4	0.361	0.171	0.212	0.024	0.008	0.178	.	0.634	0.278	0.615	0.036	0.012	0.465	0.199	0.002	0.415	2.519	0.879	0.302	0.002	0.448	2.248	-0.437	0.031	1	.	.
4032	1	1	3	3	5	1	1	1	1	0.118	0.121	0.357	0.042	0.005	0.126	.	0.200	0.205	0.615	0.073	0.025	0.465	0.199	0.002	0.415	2.519	0.879	0.302	0.002	0.448	2.248	-0.437	0.031	1	.	.
4032	1	1	3	3	6	1	2	1	2	0.067	0.144	0.140	0.020	0.005	0.149	.	0.104	0.247	0.615	0.073	0.025	0.465	0.199	0.002	0.415	2.519	0.879	0.302	0.002	0.448	2.248	-0.437	0.031	1	.	.
4032	1	1	3	3	7	1	1	2	3	0.331	0.152	0.483	0.076	0.009	0.161	.	0.550	0.255	0.615	0.073	0.025	0.465	0.199	0.002	0.415	2.519	0.879	0.302	0.002	0.448	2.248	-0.437	0.031	1	.	.
4032	1	1	3	3	8	1	2	2	4	0.461	0.176	0.223	0.039	0.036	0.212	.	0.766	0.295	0.615	0.073	0.025	0.465	0.199	0.002	0.415	2.519	0.879	0.302	0.002	0.448	2.248	-0.437	0.031	1	.	.
4032	1	1	3	4	9	1	1	1	1	0.063	0.130	0.197	0.025	0.005	0.135	.	0.097	0.206	0.615	0.027	0.009	0.465	0.199	0.002	0.415	2.519	0.879	0.302	0.002	0.448	2.248	-0.437	0.031	1	.	.
4032	1	1	3	4	10	1	2	1	2	0.059	0.130	0.097	0.013	0.005	0.135	.	0.091	0.206	0.615	0.027	0.009	0.465	0.199	0.002	0.415	2.519	0.879	0.302	0.002	0.448	2.248	-0.437	0.031	1	.	.
4032	1	1	3	4	11	1	1	2	3	0.254	0.190	0.439	0.092	0.005	0.195	.	0.400	0.300	0.615	0.027	0.009	0.465	0.199	0.002	0.415	2.519	0.879	0.302	0.002	0.448	2.248	-0.437	0.031	1	.	.
4032	1	1	3	4	12	1	2	2	4	0.442	0.167	0.301	0.039	0.007	0.174	.	0.697	0.264	0.615	0.027	0.009	0.465	0.199	0.002	0.415	2.519	0.879	0.302	0.002	0.448	2.248	-0.437	0.031	1	.	.
4032	1	1	3	6	13	1	1	1	1	0.110	0.127	0.280	0.035	0.038	0.164	.	0.185	0.211	0.615	0.001	0.001	0.465	0.199	0.002	0.415	2.519	0.879	0.302	0.002	0.448	2.248	-0.437	0.031	1	.	.
4032	1	1	3	6	14	1	2	1	2	0.086	0.107	0.192	0.020	0.005	0.112	.	0.139	0.180	0.615	0.001	0.001	0.465	0.199	0.002	0.415	2.519	0.879	0.302	0.002	0.448	2.248	-0.437	0.031	1	.	.
4032	1	1	3	6	15	1	1	2	3	0.280	0.160	0.328	0.045	0.006	0.165	.	0.462	0.263	0.615	0.001	0.001	0.465	0.199	0.002	0.415	2.519	0.879	0.302	0.002	0.448	2.248	-0.437	0.031	1	.	.
4032	1	1	3	6	16	1	2	2	4	0.377	0.155	0.198	0.027	0.020	0.175	.	0.621	0.255	0.615	0.001	0.001	0.465	0.199	0.002	0.415	2.519	0.879	0.302	0.002	0.448	2.248	-0.437	0.031	1	.	.
4032	1	1	3	7	17	1	1	1	1	-0.011	0.171	0.163	0.022	0.012	0.183	.	-0.021	0.273	0.615	0.008	0.002	0.465	0.199	0.002	0.415	2.519	0.879	0.302	0.002	0.448	2.248	-0.437	0.031	1	.	.
4032	1	1	3	7	18	1	2	1	2	0.020	0.156	0.030	0.003	0.019	0.175	.	0.032	0.251	0.615	0.008	0.002	0.465	0.199	0.002	0.415	2.519	0.879	0.302	0.002	0.448	2.248	-0.437	0.031	1	.	.
4032	1	1	3	7	19	1	1	2	3	.	.	.	.	.	.	.	.	.	.	.	.	.	.	.	.	.	.	.	.	.	.	.	.	.	.	.
4032	1	1	3	7	20	1	2	2	4	.	.	.	.	.	.	.	.	.	.	.	.	.	.	.	.	.	.	.	.	.	.	.	.	.	.	.
4032	1	1	3	8	21	1	1	1	1	0.185	0.135	0.251	0.026	0.007	0.142	.	0.282	0.212	0.615	0.001	0.001	0.465	0.199	0.002	0.415	2.519	0.879	0.302	0.002	0.448	2.248	-0.437	0.031	1	.	.
4032	1	1	3	8	22	1	2	1	2	0.123	0.130	0.211	0.022	0.005	0.135	.	0.185	0.200	0.615	0.001	0.001	0.465	0.199	0.002	0.415	2.519	0.879	0.302	0.002	0.448	2.248	-0.437	0.031	1	.	.
4032	1	1	3	8	23	1	1	2	3	0.372	0.154	0.364	0.052	0.006	0.160	.	0.602	0.256	0.615	0.001	0.001	0.465	0.199	0.002	0.415	2.519	0.879	0.302	0.002	0.448	2.248	-0.437	0.031	1	.	.
4032	1	1	3	8	24	1	2	2	4	0.425	0.146	0.269	0.035	0.041	0.188	.	0.704	0.244	0.615	0.001	0.001	0.465	0.199	0.002	0.415	2.519	0.879	0.302	0.002	0.448	2.248	-0.437	0.031	1	.	.
4032	1	1	3	10	25	1	1	1	1	.	.	.	.	.	.	.	.	.	.	.	.	.	.	.	.	.	.	.	.	.	.	.	.	.	.	.
4032	1	1	3	10	26	1	2	1	2	.	.	.	.	.	.	.	.	.	.	.	.	.	.	.	.	.	.	.	.	.	.	.	.	.	.	.
4032	1	1	3	10	27	1	1	2	3	0.362	0.181	0.322	0.060	0.013	0.193	.	0.577	0.286	0.615	0.028	0.010	0.465	0.199	0.002	0.415	2.519	0.879	0.302	0.002	0.448	2.248	-0.437	0.031	1	.	.
4032	1	1	3	10	28	1	2	2	4	0.402	0.150	0.212	0.033	0.018	0.168	.	0.635	0.236	0.615	0.028	0.010	0.465	0.199	0.002	0.415	2.519	0.879	0.302	0.002	0.448	2.248	-0.437	0.031	1	.	.
4032	1	1	3	11	29	1	1	1	1	0.000	0.144	0.141	0.018	0.006	0.150	.	0.001	0.236	0.615	0.001	0.001	0.465	0.199	0.002	0.415	2.519	0.879	0.302	0.002	0.448	2.248	-0.437	0.031	1	.	.
4032	1	1	3	11	30	1	2	1	2	0.099	0.132	0.040	0.004	0.005	0.137	.	0.168	0.216	0.615	0.001	0.001	0.465	0.199	0.002	0.415	2.519	0.879	0.302	0.002	0.448	2.248	-0.437	0.031	1	.	.
4032	1	1	3	11	31	1	1	2	3	0.391	0.174	0.153	0.020	0.014	0.188	.	0.632	0.282	0.615	0.001	0.001	0.465	0.199	0.002	0.415	2.519	0.879	0.302	0.002	0.448	2.248	-0.437	0.031	1	.	.
4032	1	1	3	11	32	1	2	2	4	0.250	0.172	0.062	0.007	0.006	0.177	.	0.410	0.277	0.615	0.001	0.001	0.465	0.199	0.002	0.415	2.519	0.879	0.302	0.002	0.448	2.248	-0.437	0.031	1	.	.
4032	1	1	3	2	33	3	3	3	5	-0.034	0.192	0.324	0.054	0.000	0.192	.	-0.146	0.890	0.223	0.036	0.012	0.371	0.115	0.002	0.155	4.346	0.640	0.347	0.002	0.072	2.135	-0.302	0.069	2	.	.
4032	1	1	3	3	34	3	3	3	5	0.000	0.217	0.271	0.059	0.000	0.217	0.659	0.001	0.975	0.223	0.073	0.025	0.371	0.115	0.002	0.155	4.346	0.640	0.347	0.002	0.072	2.135	-0.302	0.069	2	.	0.357
4032	1	1	3	4	35	3	3	3	5	0.028	0.175	0.112	0.019	0.000	0.175	.	0.128	0.809	0.223	0.027	0.009	0.371	0.115	0.002	0.155	4.346	0.640	0.347	0.002	0.072	2.135	-0.302	0.069	2	.	.
4032	1	1	3	6	36	3	3	3	5	0.069	0.175	0.640	0.112	0.000	0.175	.	0.315	0.795	0.223	0.001	0.001	0.371	0.115	0.002	0.155	4.346	0.640	0.347	0.002	0.072	2.135	-0.302	0.069	2	.	.
4032	1	1	3	7	37	3	3	3	5	.	.	.	.	.	.	.	.	.	.	.	.	.	.	.	.	.	.	.	.	.	.	.	.	.	.	.
4032	1	1	3	8	38	3	3	3	5	-0.234	0.134	0.110	0.014	0.000	0.134	.	-1.000	0.573	0.223	0.001	0.001	0.371	0.115	0.002	0.155	4.346	0.640	0.347	0.002	0.072	2.135	-0.302	0.069	2	.	.
4032	1	1	3	10	39	3	3	3	5	.	.	.	.	.	.	.	.	.	.	.	.	.	.	.	.	.	.	.	.	.	.	.	.	.	.	.
4032	1	1	3	11	40	3	3	3	5	-0.011	0.189	0.211	0.034	0.000	0.189	.	-0.054	0.819	0.223	0.001	0.001	0.371	0.115	0.002	0.155	4.346	0.640	0.347	0.002	0.072	2.135	-0.302	0.069	2	.	.
4032	1	1	4	2	1	1	1	1	1	-0.058	0.129	0.186	0.020	0.014	0.137	.	-0.106	0.223	0.575	0.001	0.001	0.525	0.211	0.040	0.007	0.190	0.961	0.180	0.035	0.005	0.262	-0.398	0.128	1	.	.
4032	1	1	4	2	2	1	2	1	2	-0.032	0.145	0.061	0.006	0.024	0.168	.	-0.059	0.260	0.575	0.001	0.001	0.525	0.211	0.040	0.007	0.190	0.961	0.180	0.035	0.005	0.262	-0.398	0.128	1	.	.
4032	1	1	4	2	3	1	1	2	3	0.306	0.179	0.271	0.029	0.028	0.168	.	0.514	0.303	0.575	0.001	0.001	0.525	0.211	0.040	0.007	0.190	0.961	0.180	0.035	0.005	0.262	-0.398	0.128	2	.	.
4032	1	1	4	2	4	1	2	2	4	0.232	0.224	0.121	0.014	0.022	0.296	.	0.397	0.381	0.575	0.001	0.001	0.525	0.211	0.040	0.007	0.190	0.961	0.180	0.035	0.005	0.262	-0.398	0.128	2	.	.
4032	1	1	4	3	5	1	1	1	1	0.013	0.134	0.400	0.044	0.005	0.139	.	0.012	0.231	0.575	0.015	0.002	0.525	0.211	0.040	0.007	0.190	0.961	0.180	0.035	0.005	0.262	-0.398	0.128	2	.	.
4032	1	1	4	3	6	1	2	1	2	0.017	0.124	0.142	0.015	0.018	0.142	.	0.020	0.215	0.575	0.015	0.002	0.525	0.211	0.040	0.007	0.190	0.961	0.180	0.035	0.005	0.262	-0.398	0.128	2	.	.
4032	1	1	4	3	7	1	1	2	3	0.307	0.168	0.263	0.028	0.014	0.183	.	0.523	0.294	0.575	0.015	0.002	0.525	0.211	0.040	0.007	0.190	0.961	0.180	0.035	0.005	0.262	-0.398	0.128	2	.	.
4032	1	1	4	3	8	1	2	2	4	0.278	0.193	0.275	0.029	0.029	0.222	.	0.481	0.329	0.575	0.015	0.002	0.525	0.211	0.040	0.007	0.190	0.961	0.180	0.035	0.005	0.262	-0.398	0.128	2	.	.
4032	1	1	4	4	9	1	1	1	1	0.039	0.133	0.290	0.031	0.010	0.143	.	0.046	0.241	0.575	0.018	0.006	0.525	0.211	0.040	0.007	0.190	0.961	0.180	0.035	0.005	0.262	-0.398	0.128	2	.	.
4032	1	1	4	4	10	1	2	1	2	0.111	0.124	0.166	0.017	0.007	0.131	.	0.186	0.205	0.575	0.018	0.006	0.525	0.211	0.040	0.007	0.190	0.961	0.180	0.035	0.005	0.262	-0.398	0.128	2	.	.
4032	1	1	4	4	11	1	1	2	3	0.347	0.177	0.187	0.020	0.009	0.186	.	0.590	0.302	0.575	0.018	0.006	0.525	0.211	0.040	0.007	0.190	0.961	0.180	0.035	0.005	0.262	-0.398	0.128	2	.	.
4032	1	1	4	4	12	1	2	2	4	0.397	0.181	0.114	0.013	0.040	0.220	.	0.676	0.310	0.575	0.018	0.006	0.525	0.211	0.040	0.007	0.190	0.961	0.180	0.035	0.005	0.262	-0.398	0.128	2	.	.
4032	1	1	4	6	13	1	1	1	1	-0.040	0.169	0.072	0.008	0.008	0.178	.	-0.059	0.298	0.575	0.002	0.001	0.525	0.211	0.040	0.007	0.190	0.961	0.180	0.035	0.005	0.262	-0.398	0.128	2	.	.
4032	1	1	4	6	14	1	2	1	2	0.086	0.128	0.040	0.004	0.008	0.136	.	0.205	0.241	0.575	0.002	0.001	0.525	0.211	0.040	0.007	0.190	0.961	0.180	0.035	0.005	0.262	-0.398	0.128	2	.	.
4032	1	1	4	6	15	1	1	2	3	0.215	0.182	0.226	0.024	0.009	0.190	.	0.401	0.337	0.575	0.002	0.001	0.525	0.211	0.040	0.007	0.190	0.961	0.180	0.035	0.005	0.262	-0.398	0.128	2	.	.
4032	1	1	4	6	16	1	2	2	4	0.323	0.153	0.128	0.014	0.014	0.167	.	0.636	0.294	0.575	0.002	0.001	0.525	0.211	0.040	0.007	0.190	0.961	0.180	0.035	0.005	0.262	-0.398	0.128	2	.	.
4032	1	1	4	7	17	1	1	1	1	-0.114	0.169	0.211	0.024	0.020	0.190	.	-0.192	0.284	0.575	0.008	0.002	0.525	0.211	0.040	0.007	0.190	0.961	0.180	0.035	0.005	0.262	-0.398	0.128	2	.	.
4032	1	1	4	7	18	1	2	1	2	-0.042	0.161	0.093	0.010	0.055	0.216	.	-0.072	0.275	0.575	0.008	0.002	0.525	0.211	0.040	0.007	0.190	0.961	0.180	0.035	0.005	0.262	-0.398	0.128	2	.	.
4032	1	1	4	7	19	1	1	2	3	.	.	.	.	.	.	.	.	.	.	.	.	.	.	.	.	.	.	.	.	.	.	.	.	.	.	.
4032	1	1	4	7	20	1	2	2	4	.	.	.	.	.	.	.	.	.	.	.	.	.	.	.	.	.	.	.	.	.	.	.	.	.	.	.
4032	1	1	4	8	21	1	1	1	1	0.087	0.163	0.094	0.011	0.027	0.189	.	0.165	0.292	0.575	0.002	0.001	0.525	0.211	0.040	0.007	0.190	0.961	0.180	0.035	0.005	0.262	-0.398	0.128	2	.	.
4032	1	1	4	8	22	1	2	1	2	0.011	0.150	0.032	0.004	0.011	0.161	.	0.024	0.321	0.575	0.002	0.001	0.525	0.211	0.040	0.007	0.190	0.961	0.180	0.035	0.005	0.262	-0.398	0.128	2	.	.
4032	1	1	4	8	23	1	1	2	3	0.327	0.180	0.054	0.006	0.037	0.217	.	0.532	0.292	0.575	0.002	0.001	0.525	0.211	0.040	0.007	0.190	0.961	0.180	0.035	0.005	0.262	-0.398	0.128	2	.	.
4032	1	1	4	8	24	1	2	2	4	.	.	.	.	.	.	.	.	.	.	.	.	.	.	.	.	.	.	.	.	.	.	.	.	.	.	.
4032	1	1	4	10	25	1	1	1	1	.	.	.	.	.	.	.	.	.	.	.	.	.	.	.	.	.	.	.	.	.	.	.	.	.	.	.
4032	1	1	4	10	26	1	2	1	2	.	.	.	.	.	.	.	.	.	.	.	.	.	.	.	.	.	.	.	.	.	.	.	.	.	.	.
4032	1	1	4	10	27	1	1	2	3	0.285	0.160	0.060	0.007	0.022	0.182	.	0.483	0.270	0.575	0.002	0.001	0.525	0.211	0.040	0.007	0.190	0.961	0.180	0.035	0.005	0.262	-0.398	0.128	2	.	.
4032	1	1	4	10	28	1	2	2	4	0.264	0.137	0.033	0.004	0.022	0.159	.	0.487	0.245	0.575	0.002	0.001	0.525	0.211	0.040	0.007	0.190	0.961	0.180	0.035	0.005	0.262	-0.398	0.128	2	.	.
4032	1	1	4	11	29	1	1	1	1	0.000	0.125	0.095	0.010	0.011	0.136	.	0.001	0.220	0.575	0.002	0.001	0.525	0.211	0.040	0.007	0.190	0.961	0.180	0.035	0.005	0.262	-0.398	0.128	2	.	.
4032	1	1	4	11	30	1	2	1	2	-0.038	0.139	0.041	0.004	0.007	0.141	.	-0.020	0.231	0.575	0.002	0.001	0.525	0.211	0.040	0.007	0.190	0.961	0.180	0.035	0.005	0.262	-0.398	0.128	2	.	.
4032	1	1	4	11	31	1	1	2	3	0.271	0.172	0.214	0.023	0.015	0.187	.	0.466	0.301	0.575	0.002	0.001	0.525	0.211	0.040	0.007	0.190	0.961	0.180	0.035	0.005	0.262	-0.398	0.128	2	.	.
4032	1	1	4	11	32	1	2	2	4	0.243	0.156	0.164	0.017	0.035	0.191	.	0.437	0.269	0.575	0.002	0.001	0.525	0.211	0.040	0.007	0.190	0.961	0.180	0.035	0.005	0.262	-0.398	0.128	2	.	.
4032	1	1	4	2	33	3	3	3	5	-0.020	0.182	0.085	0.009	0.000	0.182	.	-0.121	0.978	0.191	0.001	0.001	0.469	0.141	0.030	0.004	0.101	1.026	0.101	0.025	0.002	0.166	-0.221	0.253	1	.	.
4032	1	1	4	3	34	3	3	3	5	0.000	0.178	0.322	0.034	0.000	0.178	2.312	0.001	0.937	0.191	0.015	0.002	0.469	0.141	0.030	0.004	0.101	1.026	0.101	0.025	0.002	0.166	-0.221	0.253	1	0.557	0.557
4032	1	1	4	4	35	3	3	3	5	0.042	0.156	0.107	0.012	0.000	0.156	.	0.242	0.899	0.191	0.018	0.006	0.469	0.141	0.030	0.004	0.101	1.026	0.101	0.025	0.002	0.166	-0.221	0.253	1	.	.
4032	1	1	4	6	36	3	3	3	5	0.039	0.169	0.061	0.006	0.000	0.169	.	0.213	0.904	0.191	0.002	0.001	0.469	0.141	0.030	0.004	0.101	1.026	0.101	0.025	0.002	0.166	-0.221	0.253	1	.	.
4032	1	1	4	7	37	3	3	3	5	.	.	.	.	.	.	.	.	.	.	.	.	.	.	.	.	.	.	.	.	.	.	.	.	.	.	.
4032	1	1	4	8	38	3	3	3	5	0.195	0.167	0.092	0.010	0.000	0.167	.	0.920	0.788	0.191	0.002	0.001	0.469	0.141	0.030	0.004	0.101	1.026	0.101	0.025	0.002	0.166	-0.221	0.253	1	.	.
4032	1	1	4	10	39	3	3	3	5	.	.	.	.	.	.	.	.	.	.	.	.	.	.	.	.	.	.	.	.	.	.	.	.	.	.	.
4032	1	1	4	11	40	3	3	3	5	0.032	0.176	0.326	0.033	0.000	0.176	.	0.164	0.922	0.191	0.002	0.001	0.469	0.141	0.030	0.004	0.101	1.026	0.101	0.025	0.002	0.166	-0.221	0.253	1	.	.
4036	1	2	1	2	1	1	1	1	1	-0.217	0.133	0.018	0.020	0.005	0.138	.	-0.419	0.257	0.554	0.001	0.001	0.628	0.535	0.001	0.352	20.449	1.062	0.384	0.001	0.310	20.807	-0.906	-0.277	2	.	.
4036	1	2	1	2	2	1	2	1	2	-0.100	0.105	0.019	0.020	0.005	0.110	.	-0.193	0.203	0.554	0.001	0.001	0.628	0.535	0.001	0.352	20.449	1.062	0.384	0.001	0.310	20.807	-0.906	-0.277	2	.	.
4036	1	2	1	2	3	1	1	2	3	0.278	0.167	0.055	0.007	0.022	0.189	.	0.531	0.318	0.554	0.001	0.001	0.628	0.535	0.001	0.352	20.449	1.062	0.384	0.001	0.310	20.807	-0.906	-0.277	2	0.278	.
4036	1	2	1	2	4	1	2	2	4	0.115	0.161	0.048	0.005	0.008	0.169	.	0.217	0.308	0.554	0.001	0.001	0.628	0.535	0.001	0.352	20.449	1.062	0.384	0.001	0.310	20.807	-0.906	-0.277	2	.	.
4036	1	2	1	3	5	1	1	1	1	-0.044	0.136	0.297	0.043	0.005	0.140	.	-0.093	0.249	0.554	0.024	0.005	0.628	0.535	0.001	0.352	20.449	1.062	0.384	0.001	0.310	20.807	-0.906	-0.277	2	.	.
4036	1	2	1	3	6	1	2	1	2	-0.007	0.153	0.226	0.033	0.006	0.159	.	-0.031	0.282	0.554	0.024	0.005	0.628	0.535	0.001	0.352	20.449	1.062	0.384	0.001	0.310	20.807	-0.906	-0.277	2	.	.
4036	1	2	1	3	7	1	1	2	3	0.226	0.160	0.278	0.042	0.006	0.166	.	0.411	0.293	0.554	0.024	0.005	0.628	0.535	0.001	0.352	20.449	1.062	0.384	0.001	0.310	20.807	-0.906	-0.277	2	.	.
4036	1	2	1	3	8	1	2	2	4	0.327	0.178	0.211	0.035	0.019	0.197	.	0.578	0.331	0.554	0.024	0.005	0.628	0.535	0.001	0.352	20.449	1.062	0.384	0.001	0.310	20.807	-0.906	-0.277	2	.	.
4036	1	2	1	4	9	1	1	1	1	-0.067	0.147	0.236	0.036	0.006	0.152	.	-0.141	0.263	0.554	0.021	0.004	0.628	0.535	0.001	0.352	20.449	1.062	0.384	0.001	0.310	20.807	-0.906	-0.277	2	.	.
4036	1	2	1	4	10	1	2	1	2	0.010	0.128	0.094	0.012	0.005	0.133	.	0.014	0.230	0.554	0.021	0.004	0.628	0.535	0.001	0.352	20.449	1.062	0.384	0.001	0.310	20.807	-0.906	-0.277	2	.	.
4036	1	2	1	4	11	1	1	2	3	0.258	0.160	0.232	0.033	0.009	0.169	.	0.445	0.289	0.554	0.021	0.004	0.628	0.535	0.001	0.352	20.449	1.062	0.384	0.001	0.310	20.807	-0.906	-0.277	2	.	.
4036	1	2	1	4	12	1	2	2	4	0.287	0.163	0.140	0.020	0.016	0.179	.	0.510	0.296	0.554	0.021	0.004	0.628	0.535	0.001	0.352	20.449	1.062	0.384	0.001	0.310	20.807	-0.906	-0.277	2	.	.
4036	1	2	1	6	13	1	1	1	1	-0.114	0.159	0.097	0.013	0.005	0.164	.	-0.208	0.255	0.554	0.004	0.001	0.628	0.535	0.001	0.352	20.449	1.062	0.384	0.001	0.310	20.807	-0.906	-0.277	2	.	.
4036	1	2	1	6	14	1	2	1	2	0.054	0.135	0.076	0.008	0.005	0.140	.	0.060	0.220	0.554	0.004	0.001	0.628	0.535	0.001	0.352	20.449	1.062	0.384	0.001	0.310	20.807	-0.906	-0.277	2	.	.
4036	1	2	1	6	15	1	1	2	3	0.306	0.191	0.168	0.021	0.009	0.200	.	0.482	0.317	0.554	0.004	0.001	0.628	0.535	0.001	0.352	20.449	1.062	0.384	0.001	0.310	20.807	-0.906	-0.277	2	.	.
4036	1	2	1	6	16	1	2	2	4	0.253	0.162	0.045	0.005	0.007	0.169	.	0.374	0.270	0.554	0.004	0.001	0.628	0.535	0.001	0.352	20.449	1.062	0.384	0.001	0.310	20.807	-0.906	-0.277	2	.	.
4036	1	2	1	7	17	1	1	1	1	-0.002	0.145	0.320	0.044	0.028	0.173	.	0.008	0.277	0.554	0.008	0.001	0.628	0.535	0.001	0.352	20.449	1.062	0.384	0.001	0.310	20.807	-0.906	-0.277	2	.	.
4036	1	2	1	7	18	1	2	1	2	0.076	0.128	0.133	0.016	0.010	0.138	.	0.166	0.238	0.554	0.008	0.001	0.628	0.535	0.001	0.352	20.449	1.062	0.384	0.001	0.310	20.807	-0.906	-0.277	2	.	.
4036	1	2	1	7	19	1	1	2	3	.	.	.	.	.	.	.	.	.	.	.	.	.	.	.	.	.	.	.	.	.	.	.	.	.	.	.
4036	1	2	1	7	20	1	2	2	4	.	.	.	.	.	.	.	.	.	.	.	.	.	.	.	.	.	.	.	.	.	.	.	.	.	.	.
4036	1	2	1	8	21	1	1	1	1	-0.115	0.114	0.150	0.012	0.008	0.122	.	-0.191	0.185	0.554	0.001	0.001	0.628	0.535	0.001	0.352	20.449	1.062	0.384	0.001	0.310	20.807	-0.906	-0.277	2	.	.
4036	1	2	1	8	22	1	2	1	2	-0.123	0.134	0.070	0.008	0.005	0.139	.	-0.188	0.217	0.554	0.001	0.001	0.628	0.535	0.001	0.352	20.449	1.062	0.384	0.001	0.310	20.807	-0.906	-0.277	2	.	.
4036	1	2	1	8	23	1	1	2	3	0.208	0.196	0.402	0.107	0.019	0.215	.	0.328	0.308	0.554	0.001	0.001	0.628	0.535	0.001	0.352	20.449	1.062	0.384	0.001	0.310	20.807	-0.906	-0.277	2	.	.
4036	1	2	1	8	24	1	2	2	4	0.114	0.209	0.104	0.015	0.011	0.220	.	0.202	0.330	0.554	0.001	0.001	0.628	0.535	0.001	0.352	20.449	1.062	0.384	0.001	0.310	20.807	-0.906	-0.277	2	.	.
4036	1	2	1	10	25	1	1	1	1	.	.	.	.	.	.	.	.	.	.	.	.	.	.	.	.	.	.	.	.	.	.	.	.	.	.	.
4036	1	2	1	10	26	1	2	1	2	.	.	.	.	.	.	.	.	.	.	.	.	.	.	.	.	.	.	.	.	.	.	.	.	.	.	.
4036	1	2	1	10	27	1	1	2	3	0.277	0.153	0.443	0.078	0.016	0.169	.	0.502	0.278	0.554	0.042	0.008	0.628	0.535	0.001	0.352	20.449	1.062	0.384	0.001	0.310	20.807	-0.906	-0.277	2	.	.
4036	1	2	1	10	28	1	2	2	4	0.278	0.157	0.298	0.048	0.014	0.171	.	0.502	0.288	0.554	0.042	0.008	0.628	0.535	0.001	0.352	20.449	1.062	0.384	0.001	0.310	20.807	-0.906	-0.277	2	.	.
4036	1	2	1	11	29	1	1	1	1	0.000	0.126	0.377	0.047	0.005	0.131	.	0.001	0.229	0.554	0.008	0.001	0.628	0.535	0.001	0.352	20.449	1.062	0.384	0.001	0.310	20.807	-0.906	-0.277	2	.	.
4036	1	2	1	11	30	1	2	1	2	0.054	0.132	0.291	0.035	0.005	0.137	.	0.095	0.233	0.554	0.008	0.001	0.628	0.535	0.001	0.352	20.449	1.062	0.384	0.001	0.310	20.807	-0.906	-0.277	2	.	.
4036	1	2	1	11	31	1	1	2	3	0.244	0.151	0.398	0.063	0.006	0.157	.	0.432	0.272	0.554	0.008	0.001	0.628	0.535	0.001	0.352	20.449	1.062	0.384	0.001	0.310	20.807	-0.906	-0.277	2	.	.
4036	1	2	1	11	32	1	2	2	4	0.242	0.164	0.307	0.044	0.036	0.200	.	0.396	0.301	0.554	0.008	0.001	0.628	0.535	0.001	0.352	20.449	1.062	0.384	0.001	0.310	20.807	-0.906	-0.277	2	.	.
4036	1	2	1	2	33	3	3	3	5	0.065	0.176	0.088	0.007	0.000	0.176	.	0.218	0.637	0.277	0.001	0.001	0.587	0.409	0.001	0.382	37.441	1.068	0.318	0.001	0.364	37.745	-0.579	0.007	1	.	.
4036	1	2	1	3	34	3	3	3	5	0.000	0.237	0.422	0.083	0.000	0.237	1.452	0.001	0.853	0.277	0.024	0.005	0.587	0.409	0.001	0.382	37.441	1.068	0.318	0.001	0.364	37.745	-0.579	0.007	1	0.703	.
4036	1	2	1	4	35	3	3	3	5	-0.009	0.214	0.188	0.040	0.000	0.214	.	-0.038	0.769	0.277	0.021	0.004	0.587	0.409	0.001	0.382	37.441	1.068	0.318	0.001	0.364	37.745	-0.579	0.007	1	.	.
4036	1	2	1	6	36	3	3	3	5	0.131	0.161	0.218	0.033	0.000	0.161	.	0.457	0.568	0.277	0.004	0.001	0.587	0.409	0.001	0.382	37.441	1.068	0.318	0.001	0.364	37.745	-0.579	0.007	1	.	.
4036	1	2	1	7	37	3	3	3	5	.	.	.	.	.	.	.	.	.	.	.	.	.	.	.	.	.	.	.	.	.	.	.	.	.	.	.
4036	1	2	1	8	38	3	3	3	5	0.084	0.136	0.062	0.007	0.000	0.136	.	0.290	0.487	0.277	0.001	0.001	0.587	0.409	0.001	0.382	37.441	1.068	0.318	0.001	0.364	37.745	-0.579	0.007	1	.	.
4036	1	2	1	10	39	3	3	3	5	.	.	.	.	.	.	.	.	.	.	.	.	.	.	.	.	.	.	.	.	.	.	.	.	.	.	.
4036	1	2	1	11	40	3	3	3	5	0.134	0.167	0.277	0.048	0.000	0.167	.	0.490	0.596	0.277	0.008	0.001	0.587	0.409	0.001	0.382	37.441	1.068	0.318	0.001	0.364	37.745	-0.579	0.007	1	.	.
4036	1	2	2	2	1	1	1	1	1	.	.	.	.	.	.	.	.	.	.	.	.	.	.	.	.	.	.	.	.	.	.	.	.	.	.	.
4036	1	2	2	2	2	1	2	1	2	.	.	.	.	.	.	.	.	.	.	.	.	.	.	.	.	.	.	.	.	.	.	.	.	.	.	.
4036	1	2	2	2	3	1	1	2	3	.	.	.	.	.	.	.	.	.	.	.	.	.	.	.	.	.	.	.	.	.	.	.	.	.	.	.
4036	1	2	2	2	4	1	2	2	4	.	.	.	.	.	.	.	.	.	.	.	.	.	.	.	.	.	.	.	.	.	.	.	.	.	.	.

4036	1	2	2	3	5	1	1	1	1	0.011	0.131	0.243	0.034	0.008	0.140	.	0.019	0.270	0.491	0.008	0.001	0.545	0.121	0.004	0.009	0.104	0.957	0.034	0.003	0.015	0.093	-0.794	-0.269	2	.	.
4036	1	2	2	3	6	1	2	1	2	-0.020	0.123	0.096	0.010	0.005	0.128	.	0.002	0.247	0.491	0.008	0.001	0.545	0.121	0.004	0.009	0.104	0.957	0.034	0.003	0.015	0.093	-0.794	-0.269	2	.	.
4036	1	2	2	3	7	1	1	2	3	0.174	0.145	0.179	0.026	0.005	0.150	.	0.355	0.299	0.491	0.008	0.001	0.545	0.121	0.004	0.009	0.104	0.957	0.034	0.003	0.015	0.093	-0.794	-0.269	2	.	.
4036	1	2	2	3	8	1	2	2	4	0.329	0.134	0.090	0.010	0.008	0.141	.	0.684	0.285	0.491	0.008	0.001	0.545	0.121	0.004	0.009	0.104	0.957	0.034	0.003	0.015	0.093	-0.794	-0.269	2	.	.
4036	1	2	2	4	9	1	1	1	1	-0.027	0.142	0.432	0.060	0.006	0.148	.	-0.038	0.290	0.491	0.013	0.003	0.545	0.121	0.004	0.009	0.104	0.957	0.034	0.003	0.015	0.093	-0.794	-0.269	2	.	.
4036	1	2	2	4	10	1	2	1	2	-0.020	0.139	0.288	0.037	0.008	0.147	.	-0.016	0.286	0.491	0.013	0.003	0.545	0.121	0.004	0.009	0.104	0.957	0.034	0.003	0.015	0.093	-0.794	-0.269	2	.	.
4036	1	2	2	4	11	1	1	2	3	0.253	0.154	0.595	0.091	0.011	0.165	.	0.529	0.315	0.491	0.013	0.003	0.545	0.121	0.004	0.009	0.104	0.957	0.034	0.003	0.015	0.093	-0.794	-0.269	2	.	.
4036	1	2	2	4	12	1	2	2	4	0.269	0.149	0.383	0.056	0.012	0.161	.	0.568	0.305	0.491	0.013	0.003	0.545	0.121	0.004	0.009	0.104	0.957	0.034	0.003	0.015	0.093	-0.794	-0.269	2	.	.
4036	1	2	2	6	13	1	1	1	1	0.055	0.138	0.197	0.025	0.019	0.157	.	0.130	0.291	0.491	0.003	0.001	0.545	0.121	0.004	0.009	0.104	0.957	0.034	0.003	0.015	0.093	-0.794	-0.269	2	.	.
4036	1	2	2	6	14	1	2	1	2	-0.052	0.130	0.096	0.010	0.006	0.136	.	-0.097	0.282	0.491	0.003	0.001	0.545	0.121	0.004	0.009	0.104	0.957	0.034	0.003	0.015	0.093	-0.794	-0.269	2	.	.
4036	1	2	2	6	15	1	1	2	3	0.200	0.148	0.218	0.028	0.006	0.154	.	0.438	0.314	0.491	0.003	0.001	0.545	0.121	0.004	0.009	0.104	0.957	0.034	0.003	0.015	0.093	-0.794	-0.269	2	.	.
4036	1	2	2	6	16	1	2	2	4	0.406	0.169	0.120	0.016	0.013	0.182	.	0.900	0.359	0.491	0.003	0.001	0.545	0.121	0.004	0.009	0.104	0.957	0.034	0.003	0.015	0.093	-0.794	-0.269	2	.	.
4036	1	2	2	7	17	1	1	1	1	0.017	0.157	0.249	0.040	0.002	0.159	.	0.065	0.335	0.491	0.002	0.001	0.545	0.121	0.004	0.009	0.104	0.957	0.034	0.003	0.015	0.093	-0.794	-0.269	2	.	.
4036	1	2	2	7	18	1	2	1	2	0.002	0.149	0.162	0.024	0.010	0.159	.	0.000	0.326	0.491	0.002	0.001	0.545	0.121	0.004	0.009	0.104	0.957	0.034	0.003	0.015	0.093	-0.794	-0.269	2	.	.
4036	1	2	2	7	19	1	1	2	3	.	.	.	.	.	.	.	.	.	.	.	.	.	.	.	.	.	.	.	.	.	.	.	.	.	.	.
4036	1	2	2	7	20	1	2	2	4	.	.	.	.	.	.	.	.	.	.	.	.	.	.	.	.	.	.	.	.	.	.	.	.	.	.	.
4036	1	2	2	8	21	1	1	1	1	0.017	0.136	0.080	0.008	0.008	0.144	.	0.043	0.290	0.491	0.001	0.001	0.545	0.121	0.004	0.009	0.104	0.957	0.034	0.003	0.015	0.093	-0.794	-0.269	2	.	.
4036	1	2	2	8	22	1	2	1	2	-0.078	0.133	0.050	0.005	0.005	0.138	.	-0.180	0.306	0.491	0.001	0.001	0.545	0.121	0.004	0.009	0.104	0.957	0.034	0.003	0.015	0.093	-0.794	-0.269	2	.	.
4036	1	2	2	8	23	1	1	2	3	0.150	0.137	0.108	0.011	0.007	0.144	.	0.308	0.280	0.491	0.001	0.001	0.545	0.121	0.004	0.009	0.104	0.957	0.034	0.003	0.015	0.093	-0.794	-0.269	2	.	.
4036	1	2	2	8	24	1	2	2	4	0.295	0.132	0.132	0.015	0.017	0.149	.	0.605	0.267	0.491	0.001	0.001	0.545	0.121	0.004	0.009	0.104	0.957	0.034	0.003	0.015	0.093	-0.794	-0.269	2	.	.
4036	1	2	2	10	25	1	1	1	1	.	.	.	.	.	.	.	.	.	.	.	.	.	.	.	.	.	.	.	.	.	.	.	.	.	.	.
4036	1	2	2	10	26	1	2	1	2	.	.	.	.	.	.	.	.	.	.	.	.	.	.	.	.	.	.	.	.	.	.	.	.	.	.	.
4036	1	2	2	10	27	1	1	2	3	0.236	0.154	0.604	0.094	0.008	0.163	.	0.490	0.324	0.491	0.034	0.005	0.545	0.121	0.004	0.009	0.104	0.957	0.034	0.003	0.015	0.093	-0.794	-0.269	2	.	.
4036	1	2	2	10	28	1	2	2	4	0.290	0.155	0.361	0.055	0.007	0.162	.	0.639	0.335	0.491	0.034	0.005	0.545	0.121	0.004	0.009	0.104	0.957	0.034	0.003	0.015	0.093	-0.794	-0.269	2	.	.
4036	1	2	2	11	29	1	1	1	1	0.000	0.133	0.194	0.023	0.008	0.141	.	0.001	0.280	0.491	0.001	0.001	0.545	0.121	0.004	0.009	0.104	0.957	0.034	0.003	0.015	0.093	-0.794	-0.269	2	.	.
4036	1	2	2	11	30	1	2	1	2	0.008	0.144	0.108	0.014	0.005	0.149	.	0.036	0.280	0.491	0.001	0.001	0.545	0.121	0.004	0.009	0.104	0.957	0.034	0.003	0.015	0.093	-0.794	-0.269	2	.	.
4036	1	2	2	11	31	1	1	2	3	0.205	0.155	0.270	0.022	0.007	0.162	.	0.429	0.328	0.491	0.001	0.001	0.545	0.121	0.004	0.009	0.104	0.957	0.034	0.003	0.015	0.093	-0.794	-0.269	2	.	.
4036	1	2	2	11	32	1	2	2	4	0.264	0.160	0.137	0.017	0.012	0.172	.	0.568	0.339	0.491	0.001	0.001	0.545	0.121	0.004	0.009	0.104	0.957	0.034	0.003	0.015	0.093	-0.794	-0.269	2	.	.
4036	1	2	2	2	33	3	3	3	5	-0.250	0.211	0.020	0.002	0.000	#DIV/0!	.	#DIV/0!	#DIV/0!	0.234	0.001	0.001	0.543	0.099	0.003	0.018	0.049	0.914	0.074	0.003	0.041	0.084	-0.762	-0.215	2	.	.
4036	1	2	2	3	34	3	3	3	5	0.000	0.230	0.134	0.026	0.000	0.230	0.892	0.001	0.984	0.234	0.016	0.004	0.543	0.099	0.003	0.018	0.049	0.914	0.074	0.003	0.041	0.084	-0.762	-0.215	2	1.128	0.417
4036	1	2	2	4	35	3	3	3	5	-0.072	0.243	0.468	0.114	0.000	0.243	.	-0.277	1.044	0.234	0.002	0.001	0.543	0.099	0.003	0.018	0.049	0.914	0.074	0.003	0.041	0.084	-0.762	-0.215	2	.	.
4036	1	2	2	6	36	3	3	3	5	-0.044	0.211	0.105	0.015	0.000	0.211	.	-0.169	0.904	0.234	0.002	0.001	0.543	0.099	0.003	0.018	0.049	0.914	0.074	0.003	0.041	0.084	-0.762	-0.215	2	.	.
4036	1	2	2	7	37	3	3	3	5	.	.	.	.	.	.	.	.	.	.	.	.	.	.	.	.	.	.	.	.	.	.	.	.	.	.	.
4036	1	2	2	8	38	3	3	3	5	0.022	0.172	0.140	0.020	0.000	0.172	.	0.104	0.811	0.234	0.001	0.001	0.543	0.099	0.003	0.018	0.049	0.914	0.074	0.003	0.041	0.084	-0.762	-0.215	2	.	.
4036	1	2	2	10	39	3	3	3	5	.	.	.	.	.	.	.	.	.	.	.	.	.	.	.	.	.	.	.	.	.	.	.	.	.	.	.
4036	1	2	2	11	40	3	3	3	5	0.058	0.193	0.023	0.002	0.000	0.150	.	0.300	0.826	0.234	0.001	0.001	0.543	0.099	0.003	0.018	0.049	0.914	0.074	0.003	0.041	0.084	-0.762	-0.215	2	.	.
4036	1	2	3	2	1	1	1	1	1	.	.	.	.	.	.	.	.	.	.	.	.	.	.	.	.	.	.	.	.	.	.	.	.	.	.	.
4036	1	2	3	2	2	1	2	1	2	.	.	.	.	.	.	.	.	.	.	.	.	.	.	.	.	.	.	.	.	.	.	.	.	.	.	.
4036	1	2	3	2	3	1	1	2	3	.	.	.	.	.	.	.	.	.	.	.	.	.	.	.	.	.	.	.	.	.	.	.	.	.	.	.
4036	1	2	3	2	4	1	2	2	4	.	.	.	.	.	.	.	.	.	.	.	.	.	.	.	.	.	.	.	.	.	.	.	.	.	.	.
4036	1	2	3	3	5	1	1	1	1	0.685	0.162	0.206	0.033	0.007	0.168	.	0.948	0.252	0.668	0.039	0.006	0.511	0.086	0.003	0.534	0.527	0.800	0.102	0.003	0.537	0.588	-0.672	-0.149	2	.	.
4036	1	2	3	3	6	1	2	1	2	-0.091	0.158	0.167	0.027	0.005	0.163	.	-0.149	0.247	0.668	0.039	0.006	0.511	0.086	0.003	0.534	0.527	0.800	0.102	0.003	0.537	0.588	-0.672	-0.149	2	.	.
4036	1	2	3	3	7	1	1	2	3	0.269	0.171	0.232	0.039	0.011	0.182	.	0.417	0.271	0.668	0.039	0.006	0.511	0.086	0.003	0.534	0.527	0.800	0.102	0.003	0.537	0.588	-0.672	-0.149	2	.	.
4036	1	2	3	3	8	1	2	2	4	0.311	0.151	0.187	0.027	0.005	0.156	.	0.466	0.238	0.668	0.039	0.006	0.511	0.086	0.003	0.534	0.527	0.800	0.102	0.003	0.537	0.588	-0.672	-0.149	2	.	.
4036	1	2	3	4	9	1	1	1	1	-0.033	0.157	0.183	0.032	0.005	0.162	.	-0.049	0.230	0.668	0.004	0.001	0.511	0.086	0.003	0.534	0.527	0.800	0.102	0.003	0.537	0.588	-0.672	-0.149	2	.	.
4036	1	2	3	4	10	1	2	1	2	0.129	0.164	0.068	0.012	0.005	0.169	.	0.188	0.239	0.668	0.004	0.001	0.511	0.086	0.003	0.534	0.527	0.800	0.102	0.003	0.537	0.588	-0.672	-0.149	2	.	.
4036	1	2	3	4	11	1	1	2	3	0.209	0.166	0.093	0.015	0.005	0.171	.	0.328	0.270	0.668	0.004	0.001	0.511	0.086	0.003	0.534	0.527	0.800	0.102	0.003	0.537	0.588	-0.672	-0.149	2	.	.
4036	1	2	3	4	12	1	2	2	4	0.408	0.164	0.085	0.013	0.005	0.169	.	0.657	0.265	0.668	0.004	0.001	0.511	0.086	0.003	0.534	0.527	0.800	0.102	0.003	0.537	0.588	-0.672	-0.149	2	.	.
4036	1	2	3	6	13	1	1	1	1	0.000	0.154	0.209	0.018	0.006	0.160	.	0.001	0.236	0.668	0.004	0.001	0.511	0.086	0.003	0.534	0.527	0.800	0.102	0.003	0.537	0.588	-0.672	-0.149	2	.	.
4036	1	2	3	6	14	1	2	1	2	0.102	0.161	0.053	0.006	0.005	0.166	.	0.147	0.233	0.668	0.004	0.001	0.511	0.086	0.003	0.534	0.527	0.800	0.102	0.003	0.537	0.588	-0.672	-0.149	2	.	.
4036	1	2	3	6	15	1	1	2	3	0.420	0.162	0.082	0.012	0.005	0.167	.	0.618	0.234	0.668	0.004	0.001	0.511	0.086	0.003	0.534	0.527	0.800	0.102	0.003	0.537	0.588	-0.672	-0.149	2	.	.
4036	1	2	3	6	16	1	2	2	4	0.392	0.142	0.056	0.007	0.005	0.147	.	0.564	0.206	0.668	0.004	0.001	0.511	0.086	0.003	0.534	0.527	0.800	0.102	0.003	0.537	0.588	-0.672	-0.149	2	.	.
4036	1	2	3	7	17	1	1	1	1	-0.011	0.136	0.028	0.003	0.005	0.141	.	-0.020	0.183	0.668	0.001	0.001	0.511	0.086	0.003	0.534	0.527	0.800	0.102	0.003	0.537	0.588	-0.672	-0.149	2	.	.
4036	1	2	3	7	18	1	2	1	2	-0.067	0.126	0.020	0.002	0.005	0.131	.	-0.086	0.162	0.668	0.001	0.001	0.511	0.086	0.003	0.534	0.527	0.800	0.102	0.003	0.537	0.588	-0.672	-0.149	2	.	.
4036	1	2	3	7	19	1	1	2	3	.	.	.	.	.	.	.	.	.	.	.	.	.	.	.	.	.	.	.	.	.	.	.	.	.	.	.
4036	1	2	3	7	20	1	2	2	4	.	.	.	.	.	.	.	.	.	.	.	.	.	.	.	.	.	.	.	.	.	.	.	.	.	.	.
4036	1	2	3	8	21	1	1	1	1	0.040	0.140	0.067	0.007	0.019	0.159	.	0.033	0.233	0.668	0.001	0.001	0.511	0.086	0.003	0.534	0.527	0.800	0.102	0.003	0.537	0.588	-0.672	-0.149	2	.	.
4036	1	2	3	8	22	1	2	1	2	.	.	.	.	.	.	.	.	.	.	.	.	.	.	.	.	.	.	.	.	.	.	.	.	.	.	.
4036	1	2	3	8	23	1	1	2	3	0.419	0.173	0.040	0.004	0.005	0.178	.	0.588	0.235	0.668	0.001	0.001	0.511	0.086	0.003	0.534	0.527	0.800	0.102	0.003	0.537	0.588	-0.672	-0.149	2	.	.
4036	1	2	3	8	24	1	2	2	4	.	.	.	.	.	.	.	.	.	.	.	.	.	.	.	.	.	.	.	.	.	.	.	.	.	.	.
4036	1	2	3	10	25	1	1	1	1	.	.	.	.	.	.	.	.	.	.	.	.	.	.	.	.	.	.	.	.	.	.	.	.	.	.	.
4036	1	2	3	10	26	1	2	1	2	.	.	.	.	.	.	.	.	.	.	.	.	.	.	.	.	.	.	.	.	.	.	.	.	.	.	.
4036	1	2	3	10	27	1	1	2	3	0.144	0.163	0.222	0.033	0.005	0.168	.	0.205	0.238	0.668	0.012	0.002	0.511	0.086	0.003	0.534	0.527	0.800	0.102	0.003	0.537	0.588	-0.672	-0.149	2	.	.
4036	1	2	3	10	28	1	2	2	4	0.321	0.177	0.042	0.006	0.025	0.202	.	0.462	0.257	0.668	0.012	0.002	0.511	0.086	0.003	0.534	0.527	0.800	0.102	0.003	0.537	0.588	-0.672	-0.149	2	.	.
4036	1	2	3	11	29	1	1	1	1	0.000	0.148	0.525	0.072	0.020	0.168	.	0.001	0.223	0.668	0.006	0.001	0.511	0.086	0.003	0.534	0.527	0.800	0.102	0.003	0.537	0.588	-0.672	-0.149	2	.	.
4036	1	2	3	11	30	1	2	1	2	-0.044	0.150	0.144	0.015	0.005	0.155	.	-0.063	0.215	0.668	0.006	0.001	0.511	0.086	0.003	0.534	0.527	0.800	0.102	0.003	0.537	0.588	-0.672	-0.149	2	.	.
4036	1	2	3	11	31	1	1	2	3	0.399	0.159	0.696	0.115	0.026	0.186	.	0.592	0.245	0.668	0.006	0.001	0.511	0.086	0.003	0.534	0.527	0.800	0.102	0.003	0.537	0.588	-0.672	-0.149	2	.	.
4036	1	2	3	11	32	1	2	2	4	0.268	0.161	0.309	0.043	0.012	0.173	.	0.390	0.244	0.668	0.006	0.001	0.511	0.086	0.003	0.534	0.527	0.800	0.102	0.003	0.537	0.588	-0.672	-0.149	2	.	.
4036	1	2	3	2	33	3	3	3	5	.	.	.	.	.	.	.	.	.	.	.	.	.	.	.	.	.	.	.	.	.	.	.	.	.	.	.
4036	1	2	3	3	34	3	3	3	5	0.000	0.256	0.591	0.153	0.000	0.256	.	0.001	1.000	0.256	0.019	0.003	0.432	0.058	0.004	0.867	0.013	0.849	0.047	0.004	0.851	0.009	0.032	0.464	1	.	0.404
4036	1	2	3	4	35	3	3	3	5	0.017	0.213	0.520	0.126	0.000	0.213	.	0.063	0.836	0.256	0.019	0.003	0.432	0.058	0.004	0.867	0.013	0.849	0.047	0.004	0.851	0.009	0.032	0.464	1	.	.
4036	1	2	3	6	36	3	3	3	5	0.040	0.160	0.101	0.009	0.000	0.160	.	0.156	0.620	0.256	0.005	0.001	0.432	0.058	0.004	0.867	0.013	0.849	0.047	0.004	0.851	0.009	0.032	0.464	1	.	.
4036	1	2	3	7	37	3	3	3	5	.	.	.	.	.	.	.	.	.	.	.	.	.	.	.	.	.	.	.	.	.	.	.	.	.	.	.
4036	1	2	3	8	38	3	3	3	5	.	.	.	.	.	.	.	.	.	.	.	.	.	.	.	.	.	.	.	.	.	.	.	.	.	.	.
4036	1	2	3	10	39	3	3	3	5	.	.	.	.	.	.	.	.	.	.	.	.	.	.	.	.	.	.	.	.	.	.	.	.	.	.	.
4036	1	2	3	11	40	3	3	3	5	0.030	0.200	0.351	0.083	0.000	0.200	.	0.107	0.785	0.256	0.007	0.001	0.432	0.058	0.004	0.867	0.013	0.849	0.047	0.004	0.851	0.009	0.032	0.464	1	.	.
4036	1	2	4	2	1	1	1	1	1	.	.	.	.	.	.	.	.	.	.	.	.	.	.	.	.	.	.	.	.	.	.	.	.	.	.	.
4036	1	2	4	2	2	1	2	1	2	.	.	.	.	.	.	.	.	.	.	.	.	.	.	.	.	.	.	.	.	.	.	.	.	.	.	.
4036	1	2	4	2	3	1	1	2	3	.	.	.	.	.	.	.	.	.	.	.	.	.	.	.	.	.	.	.	.	.	.	.	.	.	.	.
4036	1	2	4	2	4	1	2	2	4	.	.	.	.	.	.	.	.	.	.	.	.	.	.	.	.	.	.	.	.	.	.	.	.	.	.	.
4036	1	2	4	3	5	1	1	1	1	0.208	0.141	0.582	0.050	0.005	0.146	.	0.295	0.215	0.659	0.005	0.001	0.581	0.381	0.005	0.335	6.676	1.072	0.290	0.005	0.393	3.683	-0.458	0.123	2	.	.
4036	1	2	4	3	6	1	2	1	2	0.359	0.128	0.294	0.038	0.005	0.133	.	0.526	0.193	0.659	0.005	0.001	0.581	0.381	0.005	0.335	6.676	1.072	0.290	0.005	0.393	3.683	-0.458	0.123	2	.	.
4036	1	2	4	3	7	1	1	2	3	0.501	0.149	0.201	0.030	0.005	0.154	.	0.740	0.230	0.659	0.005	0.001	0.581	0.381	0.005	0.335	6.676	1.072	0.290	0.005	0.393	3.683	-0.458	0.123	2	.	.
4036	1	2	4	3	8	1	2	2	4	0.662	0.134	0.128	0.017	0.007	0.141	.	0.988	0.202	0.659	0.005	0.001	0.581	0.381	0.005	0.335	6.676	1.072	0.290	0.005	0.393	3.683	-0.458	0.123	2	.	.
4036	1	2	4	4	9	1	1	1	1	0.242	0.129	0.533	0.075	0.005	0.134	.	0.330	0.199	0.659	0.043	0.010	0.581	0.381	0.005	0.335	6.676	1.072	0.290	0.005	0.393	3.683	-0.458	0.123	2	.	.
4036	1	2	4	4	10	1	2	1	2	0.232	0.127	0.256	0.028	0.005	0.132	.	0.331	0.191	0.659	0.043	0.010	0.581	0.381	0.005	0.335	6.676	1.072	0.290	0.005	0.393	3.683	-0.458	0.123	2	.	.
4036	1	2	4	4	11	1	1	2	3	0.470	0.183	0.310	0.062	0.005	0.188	.	0.675	0.276	0.659	0.043	0.010	0.581	0.381	0.005	0.335	6.676	1.072	0.290	0.005	0.393	3.683	-0.458	0.123	2	.	.
4036	1	2	4	4	12	1	2	2	4	0.551	0.170	0.173	0.030	0.005	0.175	.	0.817	0.260	0.659	0.043	0.010	0.581	0.381	0.005	0.335	6.676	1.072	0.290	0.005	0.393	3.683	-0.458	0.123	2	.	.
4036	1	2	4	6	13	1	1	1	1	0.153	0.133	0.030	0.003	0.005	0.138	.	0.235	0.204	0.659	0.001	0.001	0.581	0.381	0.005	0.335	6.676	1.072	0.290	0.005	0.393	3.683	-0.458	0.123	2	.	.
4036	1	2	4	6	14	1	2	1	2	-0.013	0.146	0.020	0.002	0.005	0.151	.	-0.020	0.224	0.659	0.001	0.001	0.581	0.381	0.005	0.335	6.676	1.072	0.290	0.005	0.393	3.683	-0.458	0.123	2	.	.
4036	1	2	4	6	15	1	1	2	3	0.357	0.167	0.040	0.004	0.005	0.172	.	0.557	0.261	0.659	0.001	0.001	0.581	0.381	0.005	0.335	6.676	1.072	0.290	0.005	0.393	3.683	-0.458	0.123	2	.	.
4036	1	2	4	6	16	1	2	2	4	0.357	0.167	0.040	0.004	0.005	0.172	.	0.557	0.261	0.659	0.001	0.001	0.581	0.381	0.005	0.335	6.676	1.072	0.290	0.005	0.393	3.683	-0.458	0.123	2	.	.
4036	1	2	4	7	17	1	1	1	1	0.228	0.133	0.106	0.010	0.005	0.138	.	0.386	0.225	0.659	0.001	0.001	0.581	0.381	0.005	0.335	6.676	1.072	0.290	0.005	0.393	3.683	-0.458	0.123	2	.	.
4036	1	2	4	7	18	1	2	1	2	0.351	0.200	0.012	0.002	0.005	0.205	.	0.595	0.339	0.659	0.001	0.001	0.581	0.381	0.005	0.335	6.676	1.072	0.290	0.005	0.393	3.683	-0.458	0.123	2	.	.
4036	1	2	4	7	19	1	1	2	3	.	.	.	.	.	.	.	.	.	.	.	.	.	.	.	.	.	.	.	.	.	.	.	.	.	.	.
4036	1	2	4	7	20	1	2	2	4	.	.	.	.	.	.	.	.	.	.	.	.	.	.	.	.	.	.	.	.	.	.	.	.	.	.	.
4036	1	2	4	8	21	1	1	1	1	0.320	0.155	0.176	0.019	0.005	0.160	.	0.431	0.221	0.659	0.001	0.001	0.581	0.381	0.005	0.335	6.676	1.072	0.290	0.005	0.393	3.683	-0.458	0.123	2	.	.
4036	1	2	4	8	22	1	2	1	2	0.327	0.124	0.093	0.008	0.005	0.129	.	0.456	0.177	0.659	0.001	0.001	0.581	0.381	0.005	0.335	6.676	1.072	0.290	0.005	0.393	3.683	-0.458	0.123	2	.	.
4036	1	2	4	8	23	1	1	2	3	0.540	0.173	0.181	0.033	0.005	0.178	.	0.752	0.250	0.659	0.001	0.001	0.581	0.381	0.005	0.335	6.676	1.072	0.290	0.005	0.393	3.683	-0.458	0.123	2	.	.
4036	1	2	4	8	24	1	2	2	4	0.723	0.149	0.037	0.004	0.005	0.154	.	1.019	0.211	0.659	0.001	0.001	0.581	0.381	0.005	0.335	6.676	1.072	0.290	0.005	0.393	3.683	-0.458	0.123	2	.	.
4036	1	2	4	10	25	1	1	1	1	.	.	.	.	.	.	.	.	.	.	.	.	.	.	.	.	.	.	.	.	.	.	.	.	.	.	.
4036	1	2	4	10	26	1	2	1	2	.	.	.	.	.	.	.	.	.	.	.	.	.	.	.	.	.	.	.	.	.	.	.	.	.	.	.
4036	1	2	4	10	27	1	1	2	3	0.465	0.190	0.160	0.028	0.005	0.195	.	0.652	0.269	0.659	0.001	0.001	0.581	0.381	0.005	0.335	6.676	1.072	0.290	0.005	0.393	3.683	-0.458	0.123	2	.	.
4036	1	2	4	10	28	1	2	2	4	0.444	0.236	0.032	0.003	0.005	0.241	.	0.648	0.333	0.659	0.001	0.001	0.581	0.381	0.005	0.335	6.676	1.072	0.290	0.005	0.393	3.683	-0.458	0.123	2	.	.
4036	1	2	4	11	29	1	1	1	1	0.000	0.142	0.062	0.007	0.005	0.147	.	0.001	0.216	0.659	0.001	0.001	0.581	0.381	0.005	0.335	6.676	1.072	0.290	0.005	0.393	3.683	-0.458	0.123	2	.	.
4036	1	2	4	11	30	1	2	1	2	0.075	0.134	0.051	0.005	0.005	0.139	.	0.128	0.204	0.659	0.001	0.001	0.581	0.381	0.005	0.335	6.676	1.072	0.290	0.005	0.393	3.683	-0.458	0.123	2	.	.
4036	1	2	4	11	31	1	1	2	3	0.294	0.212	0.051	0.006	0.005	0.217	.	0.455	0.319	0.659	0.001	0.001	0.581	0.381	0.005	0.335	6.676	1.072	0.290	0.005	0.393	3.683	-0.458	0.123	2	.	.
4036	1	2	4	11	32	1	2	2	4	0.404	0.179	0.035	0.004	0.005	0.184	.	0.613	0.270	0.659	0.001	0.001	0.581	0.381	0.005	0.335	6.676	1.072	0.290	0.005	0.393	3.683	-0.458	0.123	2	.	.
4036	1	2	4	2	33	3	3	3	5	.	.	.	.	.	.	.	.	.	.	.	.	.	.	.	.	.	.	.	.	.	.	.	.	.	.	.
4036	1	2	4	3	34	3	3	3	5	0.000	0.198	0.134	0.026	0.005	0.203	0.941	0.001	1.000	0.198	0.043	0.010	0.488	0.353	0.005	0.049	2.482	0.856	0.547	0.004	0.102	2.772	-0.498	-0.010	2	.	0.348
4036	1	2	4	4	35	3	3	3	5	-0.028	0.184	0.089	0.016	0.005	0.189	.	-0.139	0.930	0.198	0.001	0.001	0.488	0.353	0.005	0.049	2.482	0.856	0.547	0.004	0.102	2.772	-0.498	-0.010	2	.	.
4036	1	2	4	6	36	3	3	3	5	-0.056	0.184	0.010	0.002	0.005	0.189	.	-0.279	0.915	0.198	0.001	0.001	0.488	0.353	0.005	0.049	2.482	0.856	0.547	0.004	0.102	2.772	-0.498	-0.010	2	.	.
4036	1	2	4	7	37	3	3	3	5	.	.	.	.	.	.	.	.	.	.	.	.	.	.	.	.	.	.	.	.	.	.	.	.	.	.	.
4036	1	2	4	8	38	3	3	3	5	.	.	.	.	.	.	.	.	.	.	.	.	.	.	.	.	.	.	.	.	.	.	.	.	.	.	.
4036	1	2	4	10	39	3	3	3	5	.	.	.	.	.	.	.	.	.	.	.	.	.	.	.	.	.	.	.	.	.	.	.	.	.	.	.
4036	1	2	4	11	40	3	3	3	5	.	.	.	.	.	.	.	.	.	.	.	.	.														
