# Supplementary material for: Muscle activity and airflow dynamics upon gradual weight-gain volumetric enlargement or acute surgical reduction of the tongue base during chewing and swallowing
Source: PLoS One. 2026 Jul 29;21(7):e0352976. doi: 10.1371/journal.pone.0352976 (PMC13419230; doi:10.1371/journal.pone.0352976)
Supplement: Attachment — Submitted filename: Point to point response.docx [file pone.0352976.s002.docx]

**Point to point responses to journal requirements and reviewers’ comments**

Journal Requirements:

When submitting your revision, we need you to address these additional requirements.

1. Please ensure that your manuscript meets PLOS ONE's style requirements, including those for file naming. The PLOS ONE style templates can be found at

[https://journals.plos.org/plosone/s/file?id=wjVg/PLOSOne_formatting_sample_main_body.pdf](https://urldefense.com/v3/__https:/journals.plos.org/plosone/s/file?id=wjVg*PLOSOne_formatting_sample_main_body.pdf__;Lw!!K-Hz7m0Vt54!ij03i-dd5eg_dOh4x4hcbIGxga0TYYRIWUoxT6WOA2jNLT-j5q5HQLlqmBGRWzDpK39JTdCgtznqqQ$) and

[https://journals.plos.org/plosone/s/file?id=ba62/PLOSOne_formatting_sample_title_authors_affiliations.pdf](https://urldefense.com/v3/__https:/track.editorialmanager.com/CL0/https:*2F*2Fjournals.plos.org*2Fplosone*2Fs*2Ffile*3Fid=ba62*2FPLOSOne_formatting_sample_title_authors_affiliations.pdf/1/010f019b4de96223-f401055e-d7ab-4d0b-9ade-6c9b974eb982-000000/d01fOFQCwHAeoWfdVNG6pDHkoHs0qJNF_DSK4p_uMq8=243__;JSUlJSUlJQ!!K-Hz7m0Vt54!ij03i-dd5eg_dOh4x4hcbIGxga0TYYRIWUoxT6WOA2jNLT-j5q5HQLlqmBGRWzDpK39JTdAbbrPxoA$)

***Response:*** We appreciate the efficient revision process once the new Editor was assigned. We believe the revised version of the manuscript meets the style requirements.

2. [Thank you for stating in your Funding Statement:
“This study was supported by the grant R01 DE028864 from NIH/NIDCR (Z.J.L.).”

Please provide an amended statement that declares *all* the funding or sources of support (whether external or internal to your organization) received during this study, as detailed online in our guide for authors at http://journals.plos.org/plosone/s/submit-now. Please also include the statement “There was no additional external funding received for this study.” in your updated Funding Statement.
Please include your amended Funding Statement within your cover letter. We will change the online submission form on your behalf.](https://urldefense.com/v3/__http:/track.editorialmanager.com/CL0/file:*2F*2F*2FC:*2FUsers*2Fe753958*2FDesktop*2FPRTC*2520Send*2520backs*2F12-24-25*2FThank*2520you*2520for*2520stating*2520in*2520your*2520Funding*2520Statement:*2520/1/010f019b4de96223-f401055e-d7ab-4d0b-9ade-6c9b974eb982-000000/ROg6jM7BbEBEqWTAaOWI_vK8axxa5SHH6n3nIOConS0=243__;JSUlJSUlJSUlJSUlJSUlJSUlJQ!!K-Hz7m0Vt54!ij03i-dd5eg_dOh4x4hcbIGxga0TYYRIWUoxT6WOA2jNLT-j5q5HQLlqmBGRWzDpK39JTdCsIBu-rQ$)

***Response:*** *The suggested statement was added accordingly.*

3. Thank you for stating the following financial disclosure:
“This study was supported by the grant R01 DE028864 from NIH/NIDCR (Z.J.L.).”

Please state what role the funders took in the study. If the funders had no role, please state: "The funders had no role in study design, data collection and analysis, decision to publish, or preparation of the manuscript."
If this statement is not correct you must amend it as needed.
Please include this amended Role of Funder statement in your cover letter; we will change the online submission form on your behalf.

***Response:*** *The suggested statement was added accordingly.*

4. Thank you for stating the following in your Competing Interests section:
“Authors have no competing interests or other interests that might be perceived to influence the results and/or discussion reported in this paper.”

Please complete your Competing Interests on the online submission form to state any Competing Interests. If you have no competing interests, please state "The authors have declared that no competing interests exist.", as detailed online in our guide for authors at [http://journals.plos.org/plosone/s/submit-now](https://urldefense.com/v3/__http:/journals.plos.org/plosone/s/submit-now__;!!K-Hz7m0Vt54!ij03i-dd5eg_dOh4x4hcbIGxga0TYYRIWUoxT6WOA2jNLT-j5q5HQLlqmBGRWzDpK39JTdAYsTr6Gg$)
This information should be included in your cover letter; we will change the online submission form on your behalf.

***Response:*** *The suggested statement was added accordingly in the corresponding section and cover letter.*

5. In the online submission form, you indicated that [The data underlying the results presented in the study are available upon request].
All PLOS journals now require all data underlying the findings described in their manuscript to be freely available to other researchers, either 1. In a public repository, 2. Within the manuscript itself, or 3. Uploaded as supplementary information.
This policy applies to all data except where public deposition would breach compliance with the protocol approved by your research ethics board. If your data cannot be made publicly available for ethical or legal reasons (e.g., public availability would compromise patient privacy), please explain your reasons on resubmission and your exemption request will be escalated for approval.

***Response:*** *All datasets are available as supplementary information within the manuscript and attached to this submission.*

6. When completing the data availability statement of the submission form, you indicated that you will make your data available on acceptance. We strongly recommend all authors decide on a data sharing plan before acceptance, as the process can be lengthy and hold up publication timelines. Please note that, though access restrictions are acceptable now, your entire data will need to be made freely accessible if your manuscript is accepted for publication. This policy applies to all data except where public deposition would breach compliance with the protocol approved by your research ethics board. If you are unable to adhere to our open data policy, please kindly revise your statement to explain your reasoning and we will seek the editor's input on an exemption. Please be assured that once you have provided your new statement, the assessment of your exemption will not hold up the peer review process.

***Response:*** All datasets are available as supplementary information within the manuscript and attached to this submission.

7. We note that Figure 1 in your submission contain copyrighted image. All PLOS content is published under the Creative Commons Attribution License (CC BY 4.0), which means that the manuscript, images, and Supporting Information files will be freely available online, and any third party is permitted to access, download, copy, distribute, and use these materials in any way, even commercially, with proper attribution. For more information, see our copyright guidelines: [http://journals.plos.org/plosone/s/licenses-and-copyright](https://urldefense.com/v3/__http:/journals.plos.org/plosone/s/licenses-and-copyright__;!!K-Hz7m0Vt54!ij03i-dd5eg_dOh4x4hcbIGxga0TYYRIWUoxT6WOA2jNLT-j5q5HQLlqmBGRWzDpK39JTdCWTN5oDw$).

We require you to either (1) present written permission from the copyright holder to publish these figures specifically under the CC BY 4.0 license, or (2) remove the figures from your submission:

a. You may seek permission from the original copyright holder of Figure 1 to publish the content specifically under the CC BY 4.0 license.

We recommend that you contact the original copyright holder with the Content Permission Form ([http://journals.plos.org/plosone/s/file?id=7c09/content-permission-form.pdf](https://urldefense.com/v3/__http:/journals.plos.org/plosone/s/file?id=7c09*content-permission-form.pdf__;Lw!!K-Hz7m0Vt54!ij03i-dd5eg_dOh4x4hcbIGxga0TYYRIWUoxT6WOA2jNLT-j5q5HQLlqmBGRWzDpK39JTdDS32TZ5A$)) and the following text:
“I request permission for the open-access journal PLOS ONE to publish XXX under the Creative Commons Attribution License (CCAL) CC BY 4.0 ([http://creativecommons.org/licenses/by/4.0/](https://urldefense.com/v3/__http:/creativecommons.org/licenses/by/4.0/__;!!K-Hz7m0Vt54!ij03i-dd5eg_dOh4x4hcbIGxga0TYYRIWUoxT6WOA2jNLT-j5q5HQLlqmBGRWzDpK39JTdAwGy4XVw$)). Please be aware that this license allows unrestricted use and distribution, even commercially, by third parties. Please reply and provide explicit written permission to publish XXX under a CC BY license and complete the attached form.”

Please upload the completed Content Permission Form or other proof of granted permissions as an "Other" file with your submission.

In the figure caption of the copyrighted figure, please include the following text: “Reprinted from [ref] under a CC BY license, with permission from [name of publisher], original copyright [original copyright year].”

b. If you are unable to obtain permission from the original copyright holder to publish these figures under the CC BY 4.0 license or if the copyright holder’s requirements are incompatible with the CC BY 4.0 license, please either i) remove the figure or ii) supply a replacement figure that complies with the CC BY 4.0 license. Please check copyright information on all replacement figures and update the figure caption with source information. If applicable, please specify in the figure caption text when a figure is similar but not identical to the original image and is therefore for illustrative purposes only.

***Response:*** *Figure 1C was edited to illustrate the cubic-shaped array of the 8 implanted SONO crystals in a dissected tongue specimen.*

8. If the reviewer comments include a recommendation to cite specific previously published works, please review and evaluate these publications to determine whether they are relevant and should be cited. There is no requirement to cite these works unless the editor has indicated otherwise. 

***Response:*** We appreciate this advice.

Additional Editor Comments:

This manuscript evaluates how volumetric changes in the tongue base affect the coordination of chewing, swallowing, and respiration, addressing a topic of considerable academic importance and societal relevance within the medical field. In this respect, the study represents a meaningful and potentially valuable contribution.
However, based on the reviewers’ evaluations, the manuscript requires major revision before it can be considered for publication. The concerns raised extend beyond issues of clarity or presentation and involve substantive matters related to the conceptual framing, interpretation of results, and overall structure of the manuscript.
Accordingly, acceptance of this manuscript is contingent upon a comprehensive, point-by-point response to each of the reviewers’ comments and questions, accompanied by substantial revisions to the manuscript. The revised version should clearly document how each concern has been addressed. I encourage the authors to carefully consider the reviewers’ feedback and to submit a thoroughly revised manuscript for further evaluation.

***Response:*** *We appreciate the comments and we have proceeded accordingly.*

Reviewers' comments:
Reviewer's Responses to Questions

**Comments to the Author**

1. Is the manuscript technically sound, and do the data support the conclusions?

The manuscript must describe a technically sound piece of scientific research with data that supports the conclusions. Experiments must have been conducted rigorously, with appropriate controls, replication, and sample sizes. The conclusions must be drawn appropriately based on the data presented.

Reviewer #1: Yes

Reviewer #2: No

2. Has the statistical analysis been performed appropriately and rigorously?

Reviewer #1: Yes

Reviewer #2: I Don't Know

3. Have the authors made all data underlying the findings in their manuscript fully available?

The [PLOS Data policy](https://urldefense.com/v3/__http:/track.editorialmanager.com/CL0/http:*2F*2Fwww.plosone.org*2Fstatic*2Fpolicies.action*23sharing/1/010f019b4de96223-f401055e-d7ab-4d0b-9ade-6c9b974eb982-000000/_pVddZm8WmFAsEUNMI6jf-0k3l5BgwsvqY3FxhaM510=243__;JSUlJSU!!K-Hz7m0Vt54!ij03i-dd5eg_dOh4x4hcbIGxga0TYYRIWUoxT6WOA2jNLT-j5q5HQLlqmBGRWzDpK39JTdCQsIJSJg$) requires authors to make all data underlying the findings described in their manuscript fully available without restriction, with rare exception (please refer to the Data Availability Statement in the manuscript PDF file). The data should be provided as part of the manuscript or its supporting information or deposited to a public repository. For example, in addition to summary statistics, the data points behind means, medians and variance measures should be available. If there are restrictions on publicly sharing data—e.g. participant privacy or use of data from a third party—those must be specified.

Reviewer #1: Yes

Reviewer #2: No

4. Is the manuscript presented in an intelligible fashion and written in standard English?

PLOS ONE does not copyedit accepted manuscripts, so the language in submitted articles must be clear, correct, and unambiguous. Any typographical or grammatical errors should be corrected at revision, so please note any specific errors here.

Reviewer #1: Yes

Reviewer #2: No

5. Review Comments to the Author

Please use the space provided to explain your answers to the questions above. You may also include additional comments for the author, including concerns about dual publication, research ethics, or publication ethics. (Please upload your review as an attachment if it exceeds 20,000 characters).

**Reviewer #1**

This study provides a mechanistically insightful analysis of how volumetric alterations of the tongue base affect the coordination of chewing, swallowing, and respiration. The combination of EMG, kinematic, and respiratory measurements in a longitudinal design is a major strength. In addition, the finding that both tongue volume reduction and enlargement induce functional adaptations in the timing and amplitude of muscle activity is particularly interesting. However, there are several concerns that should be addressed prior to publication.

***Response:*** *We appreciate these positive comments on our work.*

Major points
-The manuscript compares changes in tongue kinematics associated with tongue volume enlargement and tongue volume reduction and discusses potential differences in their underlying mechanisms. However, the experimental procedures for these two conditions are fundamentally different. Tongue volume enlargement is induced gradually through obesity, whereas tongue volume reduction is achieved surgically, resulting in an abrupt decrease in tissue volume followed by postoperative adaptation. These two approaches may probe different physiological responses (chronic adaptation vs. acute injury and recovery). Please clarify whether direct comparisons between these two conditions are appropriate and discuss this limitation more explicitly.

***Response:*** *We appreciate that the reviewer indicated this important drawback of these two different minipig models. This difference (acute injury/wound healing vs chronic adaptation to obesity) was noted in the section of Materials and Methods, and further discussed in the section of Discussion.*

-The volume-enlarged group is assumed to represent tongue base enlargement due to obesity. However, longitudinal measurements of tongue base volume were not provided. Please clarify whether tongue base volume was measured at baseline and at subsequent time points, or whether the reported values represent terminal measurements only.

***Response:*** *Since the measurements of weights and volumes of the tongue were not available in living animals. The data provided are postmortem measurements. We also provided the postmortem measurements of the control group published previously. However, it must be noted that these control animals were 5 weeks younger than those in the present study.*

-The manuscript provides little discussion of anatomical differences between minipigs and humans with respect to tongue base muscle composition, fat distribution, and pharyngeal morphology. For translational relevance, it would be important to clearly state which anatomical features are comparable and which differ between the two species.

***Response:*** *We appreciate this comment. The detailed comparison of the tongue and oropharyngeal structures between minipigs and humas have been added in the section of Discussion.*

-Please clarify why week 5 was chosen as the final observation time point. In particular, for the tongue reduction group, it is possible that this time point still represents an intermediate stage of recovery rather than full functional stabilization.

***Response:*** *The period of 5 weeks was chosen because our previous study on the surgical volumetric reduction of the tongue body showed complete would healing at 4 weeks in the same minipig model. Yes, we agree that longer time period could provide further insights into fibrosis formation, muscle regeneration and remodeling, and functional recovery/compensation. However, given the limited life span of the implanted SONO crystals and leading cables (skin button) and potential risk of continuous obesity on animals’ welfare, the 5-week experimental period was decided. This rationale was added to the section of Materials and Methods.*

Minor points
-At abstract, conclusions could be stated more concretely. The current conclusion is too broad.

***Response:*** *The conclusion in Abstract was revised accordingly.*

-Given the interdisciplinary of PLOS ONE, it would be helpful to include a schematic figure early in the manuscript illustrating the anatomical locations and principal fiber orientations of the recorded muscles (DA, MA, SG, GG, LVP, TVP, TH, MC) in the minipig.

***Response:*** *We appreciate this comment. However, given the multiple figures are already included in this MS and the detailed anatomy of these muscles can be easily found in various social media, these additions may not be necessary.*

-The description of the respiratory results could be made more concise and more explicitly linked to the figures. In particular, when comparing volume reduction and enlargement, it is important to ensure that identical time points and experimental conditions are being compared.

***Response:*** *Thank you for this comment. We do not fully understand the actual meaning of “The description of the respiratory results could be made more concise and more explicitly linked to the figures”. Yes, the data from identical time points and experimental conditions were compared. The relevant description was revised to reflect these comparisons.*

-For airflow velocity and tidal volume, the values in the volume-reduced group appear to be markedly elevated at week 3. Please clarify whether any specific physiological or experimental factors may explain this pronounced change at this time point.

***Response:*** *We appreciate this comment. We have addressed this specific finding in the section of Discussion.*

-The Discussion states that “the current findings in EMG activity timings may provide certain clues for the mechanism of these potential swallowing disorders.” However, the minipigs in the volume-reduction group did not exhibit overt swallowing disorders. Please clarify which specific changes in muscle activity are hypothesized to predispose to dysphagia and how these relate to subclinical swallowing impairment.

***Response:*** *The minipigs of the reduction group in the present study showed delayed activity onsets of the tongue and soft palate muscles, this may potentially cause swallowing disorders either during would healing process or due to fibrosis formation over time. These potential mechanism has been explained throughout the manuscript.*

-Please discuss which postoperative time period is considered the highest risk for swallowing dysfunction and airway compromise following tongue base reduction.

***Response:*** *Clinically, swallowing disorders become the worst in the first three weeks after the surgical volumetric reduction of the tongue base. The airway compromise is at highest risk in the first 2 days after this surgery due to* swelling*. There is also airway risk due to wound bleeding, if it happens, in the first three weeks after the surgery. These explanations were added to the discussion section. All of these are based upon clinical experiences by the co-author, Dr. Edward Weaver, Chief of Sleep Surgery, UW Medicine.*

-If obesity was induced systemically, is it possible that the soft palate also increased in volume? Please comment on whether changes in soft palate size or compliance may have contributed to the observed functional differences.

***Response:*** *Yes****,*** *obesity has systemic influences in the body. The enlargement and/or more fat tissue infiltration in the soft palate and other oropharyngeal structures, such as pharyngeal walls and epiglottis, might occur as well. These confounding factors cannot be rolled out in the present study. These potential confounding factors have been added in the section of Discussion.*

**Reviewer #2:**

-The authors evaluated tongue muscle function under two distinct conditions: obesity-induced tongue enlargement and surgically induced tongue base reduction, using an experimental framework established in the authors’ previous studies. The experimental methods themselves appear to be technically sound. However, there is a fundamental lack of logical consistency between the stated aims of the study (as presented in the title and throughout the manuscript) and the actual scope of the analyses. The title and main text describe the objective as clarifying the effects of tongue base volume enlargement and reduction on tongue muscle function. In practice, the study examines responses under two highly specific and physiologically distinct conditions (obesity and surgical intervention). Because these conditions are inherently asymmetric, it is difficult to attribute the observed effects solely to changes in tongue volume as a geometric factor.

***Response:*** *Thank you for this comment. Yes, the two groups are indeed different in both volumetric alterations of the tongue base and physiological responses to these alterations as indicated by the reviewer#1. Your asymmetric comments may indicate the two models with chronic systemic obesity versus acute surgical wound effects with reduction). However, the two states are highly relevant clinical states about which we seek understanding. In fact, what we analyzed is the functions of chewing, swallowing and synchronized respiration via rea-time recording of activities in the tongue, palatal, hyoid and pharyngeal, and jaw muscles. Therefore, we believe the title is consistent with the analyses.*


-The rationale for the stated hypotheses also requires further clarification. The Introduction proposes that “a volume-reduced tongue base may induce swallowing disorders whereas a volume-enlarged tongue base may induce alterations in the respiratory dynamics during feeding”. While it is reasonable to expect that tongue volume changes influence both swallowing and respiration, the manuscript does not clearly explain why these effects are hypothesized to be function-specific in this asymmetric manner. In addition, many of the reported results and corresponding discussions appear to reflect time-dependent changes associated with postoperative wound healing rather than effects of tongue volume alteration itself. Further clarification would be helpful regarding whether the authors intend to investigate the direct geometric effects of volume change, or to describe functional adaptations under these specific pathological conditions. Addressing this issue would require a fundamental redefinition of the study aims and overall structure, rather than clarification or reorganization of the existing manuscript.

***Response:*** *Thank you for the insights. Although the physiological responses to the reduction by the coblation (acute injury and wound healing) and enlargement via obesity (chronic adaptation), the comparisons were between the sibling pairs. Yes, the wound healing process could affect oropharyngeal function over time, but the clear and confirmed consequence of this intervention was the volumetric reduction of the tongue base. Therefore, what we studied was the functional alterations of volume-reduced and enlarged tongue base, rather than postoperative responses. It needs to be emphasized that both reduction and enlargement minipig models we used in the present study completely mimic the real clinical conditions which Dr. Edward Weaver (Co-author, chief of sleep surgery, UW Medicine) often deals with. Therefore, these models are highly clinically relevant.*
