# Supplementary material for: Muscle activity and airflow dynamics upon gradual weight-gain volumetric enlargement or acute surgical reduction of the tongue base during chewing and swallowing
Source: PLoS One. 2026 Jul 29;21(7):e0352976. doi: 10.1371/journal.pone.0352976 (PMC13419230; doi:10.1371/journal.pone.0352976)
Supplement: Attachment — Submitted filename: PLOS ONE Responses - final version.docx [file pone.0352976.s003.docx]

**Point by point responses to the comments from editor and reviewer #2.**

**From the academic editor:**

Thank you for submitting your manuscript to PLOS ONE. After careful consideration, we have decided that your manuscript does not meet our criteria for publication and must therefore be rejected.

Specifically:

The authors did not provide a logical and sufficiently reasoned response to the concerns raised by Reviewer 2. The rebuttal lacks a clear explanation addressing the specific methodological and conceptual issues that were pointed out. As a result, the fundamental concerns remain unresolved.

***Response: To the Question #1 asking, “If the authors have adequately addressed your comments raised in a previous round of review and you feel that this manuscript is now acceptable for publication,…” reviewer #2 answered “YES” affirmatively, and also answered “YES” affirmatively to the Questions # 2, 4, and 5 to indicate 1) the manuscript technically sound, and the data support the conclusions; 2) the authors made all data underlying the findings in their manuscript fully available; and 3) the manuscript presented in an intelligible fashion and written in standard English. In the Question #6 of Review Comments to the Author, reviewer #2 requested that we simply needed to “1) redefine the study as a comparison of clinically relevant pathological models rather than as a test of the pure effect of volume change, and 2) to provide a clearer theoretical justification for using volume as the primary comparison axis, together with an explicit statement of the limitations of that interpretation."' We believe we did those in the last revision.  We realized that our explanations might not be quite clear enough, so we are now responding to these comments more clearly particularly in terms of the conceptual framework and the limitation of the present study.***

**From Reviewer #2:**

Thank you for your response. However, my concern is primarily about the overall logical framework of the study, whereas the authors’ reply mainly emphasizes the validity of the measurements and the clinical relevance of the models. As a result, the response does not fully address the point being raised.

***Responses: In addition to the validity of the measurements and the clinical relevance of the models, we are now stating the clear conceptual framework logically in this revision (please see below).***

1. The authors’ response explains the clinical relevance of the models and provides details on the measurements, but it does not address the key issue in this review regarding the logical validity of the comparison axis. If the authors acknowledge the asymmetry between the obesity model and the post-surgical model, then at least one of the following would be needed: (1) to redefine the study as a comparison of clinically relevant pathological models rather than as a test of the pure effect of volume change; or (2) to provide a clearer theoretical justification for using volume as the primary comparison axis, together with an explicit statement of the limitations of that interpretation.

***Response: We really appreciate this very important point. It is absolutely right that the study is about the comparisons between the two clinically relevant pathological models rather than their pure symmetric effects of volumetric alterations of the tongue base. The structure or diagram of the conceptual vs theoretical framework in the present study are as follows:*** ***Theoretical: the tongue base size/volume affects oropharyngeal function, including respiration, chewing, swallowing and others; Conceptual: volume enlarged – obese minipigs with enlarged tongue base as confirmed by multiple animal and clinical studies (Baker et al. 2025; Brennick et al. 2009; Brennick et al. 2006; Isono 2011; Kim et al. 2014); volume reduced – normal weighted minipigs with surgical volume reduction of the tongue base as widely applied clinically (Li et al. 2016). These two models have high clinical relevance but are not symmetric as responded in the last revision. Therefore, despite the observed effects in the late time point (weeks 3 and 5) of reduction animals might mainly result from the proposed intervention (surgical volumetric reduction), these effects in obese animals (enlargement) might also result from enlargements of other oropharyngeal structures, such as tongue body, soft palate, lateral pharyngeal fat pads, etc. These confounding factors have been incorporated in this revision. In addition, due to the surgical injury in the volumetric reduction group and wound healing lasting usually two weeks which are based upon the data from similar model of volumetric reduction of the tongue body in our previously published study (Perkins et al. 2008; Shcherbatyy et al. 2008), the observed effects at early time point of week 1 may be confounded by the wound healing process as well. That said that acute surgical impact is also important to understand in its own right in order to appreciate the acute (not just chronic) impacts of surgery. All these confounding factors and limitations of the study have been comprehensively discussed in this revision. Additionally, text was added in the introduction section explaining the theoretical justification of these two clinically relevant models.***

2. Related to the above, the authors acknowledge the possible influence of wound healing, yet conclude that the study evaluated volume-related functional alterations rather than postoperative responses on the basis that volume reduction was confirmed. However, confirmation of a morphological change does not demonstrate that volume change is the primary driver of the observed functional changes. There remains a logical leap in this *causal interpretation.*

***Response: It is absolutely right that postoperative responses might have confounded the results in the reduction group at early time point as responded above. Therefore, we emphasized in this revision that 1) functional changes of reduction animals in EMG at early time point might not be driven by the volume reduction; and 2) functional changes of enlargement (obese) animals in EMG might also be driven by increased size/volume of other oropharyngeal structures. Nevertheless, the observed changes in both clinical pathological conditions are mainly attributed to the induced volumetric changes of the tongue base. These consequences are mostly observed in patients with either one or both alterations and progressively showing functional impairment in respiration and mastication (chewing and swallowing) with the alterations in oropharyngeal muscle activity. Thus, the present study may provide some insights into the mechanism of these oropharyngeal functional disorders due to the volumetric changes in the tongue base.***

***References***

***Baker E, Chanamolu M, Nieri C, White SF, Brandt J, Gillespie MB. 2025. The effect of tongue volume and adipose content on obstructive sleep apnea: Meta-analysis & systematic review. OTO Open. 9(2):e70067.***

***Brennick MJ, Pack AI, Ko K, Kim E, Pickup S, Maislin G, Schwab RJ. 2009. Altered upper airway and soft tissue structures in the new zealand obese mouse. Am J Respir Crit Care Med. 179(2):158-169.***

***Brennick MJ, Pickup S, Cater JR, Kuna ST. 2006. Phasic respiratory pharyngeal mechanics by magnetic resonance imaging in lean and obese zucker rats. Am J Respir Crit Care Med. 173(9):1031-1037.***

***Isono S. 2011. Obesity and obstructive sleep apnoea: Mechanisms for increased collapsibility of the passive pharyngeal airway. Respirology. 17(1):32-42.***

***Kim AM, Keenan BT, Jackson N, Chan EL, Staley B, Poptani H, Torigian DA, Pack AI, Schwab RJ. 2014. Tongue fat and its relationship to obstructive sleep apnea. Sleep. 37(10):1639-1648.***

***Li HY, Lee LA, Kezirian EJ. 2016. Coblation endoscopic lingual lightening (cell) for obstructive sleep apnea. Eur Arch Otorhinolaryngol. 273(1):231-236.***

***Perkins JA, Shcherbatyy V, Liu ZJ. 2008. Morphologic and histologic outcomes of tongue reduction surgery in an animal model. Otolaryngol Head Neck Surg. 139(2):291-297.***

***Shcherbatyy V, Perkins JA, Liu ZJ. 2008. Internal kinematics of the tongue following volume reduction. Anat Rec (Hoboken). 291(7):886-893.***
