# Supplementary material for: Muscle activity and airflow dynamics upon gradual weight-gain volumetric enlargement or acute surgical reduction of the tongue base during chewing and swallowing
Source: PLoS One. 2026 Jul 29;21(7):e0352976. doi: 10.1371/journal.pone.0352976 (PMC13419230; doi:10.1371/journal.pone.0352976)
Supplement: Peer review history

# Author comment: Author response to Decision Letter 0

**DOI:** 10.1371/journal.pone.0352976.r001

**Transfer Alert**

This paper was transferred from another journal. As a result, its full editorial history (including decision letters, peer reviews and author responses) may not be present.

12 Sep 2025

# Peer review: Decision Letter 0

**DOI:** 10.1371/journal.pone.0352976.r002

**Authors:** Kazunori Nozaki

23 Dec 2025

PONE-D-25-49745

Effects of Volumetric Enlargement or Reduction of the Tongue Base on Chewing and Swallowing

PLOS One

Dear Dr. Liu,

Thank you for submitting your manuscript to PLOS ONE. After careful consideration, we feel that it has merit but does not fully meet PLOS ONE’s publication criteria as it currently stands. Therefore, we invite you to submit a revised version of the manuscript that addresses the points raised during the review process.

Please submit your revised manuscript by Feb 06 2026 11:59PM. If you will need more time than this to complete your revisions, please reply to this message or contact the journal office at plosone@plos.org. When you're ready to submit your revision, log on to https://www.editorialmanager.com/pone/ and select the 'Submissions Needing Revision' folder to locate your manuscript file.

Please include the following items when submitting your revised manuscript:

- A letter that responds to each point raised by the academic editor and reviewer(s). You should upload this letter as a separate file labeled 'Response to Reviewers'.
- A marked-up copy of your manuscript that highlights changes made to the original version. You should upload this as a separate file labeled 'Revised Manuscript with Track Changes'.
- An unmarked version of your revised paper without tracked changes. You should upload this as a separate file labeled 'Manuscript'.

If you would like to make changes to your financial disclosure, please include your updated statement in your cover letter. Guidelines for resubmitting your figure files are available below the reviewer comments at the end of this letter.

If applicable, we recommend that you deposit your laboratory protocols in protocols.io to enhance the reproducibility of your results. Protocols.io assigns your protocol its own identifier (DOI) so that it can be cited independently in the future. For instructions see: https://journals.plos.org/plosone/s/submission-guidelines#loc-laboratory-protocols. Additionally, PLOS ONE offers an option for publishing peer-reviewed Lab Protocol articles, which describe protocols hosted on protocols.io. Read more information on sharing protocols at https://plos.org/protocols?utm_medium=editorial-email&utm_source=authorletters&utm_campaign=protocols.

We look forward to receiving your revised manuscript.

Kind regards,

Kazunori Nozaki, DDS,PhD,PhD

Academic Editor

PLOS One

Journal Requirements:

When submitting your revision, we need you to address these additional requirements.

1. Please ensure that your manuscript meets PLOS ONE's style requirements, including those for file naming. The PLOS ONE style templates can be found at

https://journals.plos.org/plosone/s/file?id=wjVg/PLOSOne_formatting_sample_main_body.pdf and

https://journals.plos.org/plosone/s/file?id=ba62/PLOSOne_formatting_sample_title_authors_affiliations.pdf

2. Thank you for stating in your Funding Statement:

“This study was supported by the grant R01 DE028864 from NIH/NIDCR (Z.J.L.).”

Please provide an amended statement that declares *all* the funding or sources of support (whether external or internal to your organization) received during this study, as detailed online in our guide for authors at http://journals.plos.org/plosone/s/submit-now. Please also include the statement “There was no additional external funding received for this study.” in your updated Funding Statement.

Please include your amended Funding Statement within your cover letter. We will change the online submission form on your behalf.

3. Thank you for stating the following financial disclosure:

“This study was supported by the grant R01 DE028864 from NIH/NIDCR (Z.J.L.).”

Please state what role the funders took in the study. If the funders had no role, please state: "The funders had no role in study design, data collection and analysis, decision to publish, or preparation of the manuscript."

If this statement is not correct you must amend it as needed.

Please include this amended Role of Funder statement in your cover letter; we will change the online submission form on your behalf.

4. Thank you for stating the following in your Competing Interests section:

“Authors have no competing interests or other interests that might be perceived to influence the results and/or discussion reported in this paper.”

Please complete your Competing Interests on the online submission form to state any Competing Interests. If you have no competing interests, please state "The authors have declared that no competing interests exist.", as detailed online in our guide for authors at http://journals.plos.org/plosone/s/submit-now

This information should be included in your cover letter; we will change the online submission form on your behalf.

5. In the online submission form, you indicated that [The data underlying the results presented in the study are available upon request].

All PLOS journals now require all data underlying the findings described in their manuscript to be freely available to other researchers, either 1. In a public repository, 2. Within the manuscript itself, or 3. Uploaded as supplementary information.

This policy applies to all data except where public deposition would breach compliance with the protocol approved by your research ethics board. If your data cannot be made publicly available for ethical or legal reasons (e.g., public availability would compromise patient privacy), please explain your reasons on resubmission and your exemption request will be escalated for approval.

6. When completing the data availability statement of the submission form, you indicated that you will make your data available on acceptance. We strongly recommend all authors decide on a data sharing plan before acceptance, as the process can be lengthy and hold up publication timelines. Please note that, though access restrictions are acceptable now, your entire data will need to be made freely accessible if your manuscript is accepted for publication. This policy applies to all data except where public deposition would breach compliance with the protocol approved by your research ethics board. If you are unable to adhere to our open data policy, please kindly revise your statement to explain your reasoning and we will seek the editor's input on an exemption. Please be assured that, once you have provided your new statement, the assessment of your exemption will not hold up the peer review process.

7. We note that Figure 1 in your submission contain copyrighted image. All PLOS content is published under the Creative Commons Attribution License (CC BY 4.0), which means that the manuscript, images, and Supporting Information files will be freely available online, and any third party is permitted to access, download, copy, distribute, and use these materials in any way, even commercially, with proper attribution. For more information, see our copyright guidelines: http://journals.plos.org/plosone/s/licenses-and-copyright.

We require you to either (1) present written permission from the copyright holder to publish these figures specifically under the CC BY 4.0 license, or (2) remove the figures from your submission:

a. You may seek permission from the original copyright holder of Figure 1 to publish the content specifically under the CC BY 4.0 license.

We recommend that you contact the original copyright holder with the Content Permission Form (http://journals.plos.org/plosone/s/file?id=7c09/content-permission-form.pdf) and the following text:

“I request permission for the open-access journal PLOS ONE to publish XXX under the Creative Commons Attribution License (CCAL) CC BY 4.0 (http://creativecommons.org/licenses/by/4.0/). Please be aware that this license allows unrestricted use and distribution, even commercially, by third parties. Please reply and provide explicit written permission to publish XXX under a CC BY license and complete the attached form.”

Please upload the completed Content Permission Form or other proof of granted permissions as an "Other" file with your submission.

In the figure caption of the copyrighted figure, please include the following text: “Reprinted from [ref] under a CC BY license, with permission from [name of publisher], original copyright [original copyright year].”

b. If you are unable to obtain permission from the original copyright holder to publish these figures under the CC BY 4.0 license or if the copyright holder’s requirements are incompatible with the CC BY 4.0 license, please either i) remove the figure or ii) supply a replacement figure that complies with the CC BY 4.0 license. Please check copyright information on all replacement figures and update the figure caption with source information. If applicable, please specify in the figure caption text when a figure is similar but not identical to the original image and is therefore for illustrative purposes only.

8. If the reviewer comments include a recommendation to cite specific previously published works, please review and evaluate these publications to determine whether they are relevant and should be cited. There is no requirement to cite these works unless the editor has indicated otherwise.

Additional Editor Comments:

This manuscript evaluates how volumetric changes in the tongue base affect the coordination of chewing, swallowing, and respiration, addressing a topic of considerable academic importance and societal relevance within the medical field. In this respect, the study represents a meaningful and potentially valuable contribution.

However, based on the reviewers’ evaluations, the manuscript requires major revision before it can be considered for publication. The concerns raised extend beyond issues of clarity or presentation and involve substantive matters related to the conceptual framing, interpretation of results, and overall structure of the manuscript.

Accordingly, acceptance of this manuscript is contingent upon a comprehensive, point-by-point response to each of the reviewers’ comments and questions, accompanied by substantial revisions to the manuscript. The revised version should clearly document how each concern has been addressed. I encourage the authors to carefully consider the reviewers’ feedback and to submit a thoroughly revised manuscript for further evaluation.

[Note: HTML markup is below. Please do not edit.]

Reviewers' comments:

Reviewer's Responses to Questions

**Comments to the Author**

1. Is the manuscript technically sound, and do the data support the conclusions?

The manuscript must describe a technically sound piece of scientific research with data that supports the conclusions. Experiments must have been conducted rigorously, with appropriate controls, replication, and sample sizes. The conclusions must be drawn appropriately based on the data presented.

Reviewer #1: Yes

Reviewer #2: No

**********

2. Has the statistical analysis been performed appropriately and rigorously?

Reviewer #1: Yes

Reviewer #2: I Don't Know

**********

3. Have the authors made all data underlying the findings in their manuscript fully available?

The PLOS Data policy requires authors to make all data underlying the findings described in their manuscript fully available without restriction, with rare exception (please refer to the Data Availability Statement in the manuscript PDF file). The data should be provided as part of the manuscript or its supporting information, or deposited to a public repository. For example, in addition to summary statistics, the data points behind means, medians and variance measures should be available. If there are restrictions on publicly sharing data—e.g. participant privacy or use of data from a third party—those must be specified.

Reviewer #1: Yes

Reviewer #2: No

**********

4. Is the manuscript presented in an intelligible fashion and written in standard English?

PLOS ONE does not copyedit accepted manuscripts, so the language in submitted articles must be clear, correct, and unambiguous. Any typographical or grammatical errors should be corrected at revision, so please note any specific errors here.

Reviewer #1: Yes

Reviewer #2: No

**********

5. Review Comments to the Author

Please use the space provided to explain your answers to the questions above. You may also include additional comments for the author, including concerns about dual publication, research ethics, or publication ethics. (Please upload your review as an attachment if it exceeds 20,000 characters)

Reviewer #1: This study provides a mechanistically insightful analysis of how volumetric alterations of the tongue base affect the coordination of chewing, swallowing, and respiration. The combination of EMG, kinematic, and respiratory measurements in a longitudinal design is a major strength. In addition, the finding that both tongue volume reduction and enlargement induce functional adaptations in the timing and amplitude of muscle activity is particularly interesting. However, there are several concerns that should be addressed prior to publication.

Major points

The manuscript compares changes in tongue kinematics associated with tongue volume enlargement and tongue volume reduction and discusses potential differences in their underlying mechanisms. However, the experimental procedures for these two conditions are fundamentally different. Tongue volume enlargement is induced gradually through obesity, whereas tongue volume reduction is achieved surgically, resulting in an abrupt decrease in tissue volume followed by postoperative adaptation. These two approaches may probe different physiological responses (chronic adaptation vs. acute injury and recovery). Please clarify whether direct comparisons between these two conditions are appropriate and discuss this limitation more explicitly.

The volume-enlarged group is assumed to represent tongue base enlargement due to obesity. However, longitudinal measurements of tongue base volume were not provided. Please clarify whether tongue base volume was measured at baseline and at subsequent time points, or whether the reported values represent terminal measurements only.

The manuscript provides little discussion of anatomical differences between minipigs and humans with respect to tongue base muscle composition, fat distribution, and pharyngeal morphology. For translational relevance, it would be important to clearly state which anatomical features are comparable and which differ between the two species.

Please clarify why week 5 was chosen as the final observation time point. In particular, for the tongue reduction group, it is possible that this time point still represents an intermediate stage of recovery rather than full functional stabilization.

Minor points

At abstract, conclusions could be stated more concretely. The current conclusion is too broad.

Given the interdisciplinary of PLOS ONE, it would be helpful to include a schematic figure early in the manuscript illustrating the anatomical locations and principal fiber orientations of the recorded muscles (DA, MA, SG, GG, LVP, TVP, TH, MC) in the minipig.

The description of the respiratory results could be made more concise and more explicitly linked to the figures. In particular, when comparing volume reduction and enlargement, it is important to ensure that identical time points and experimental conditions are being compared.

For airflow velocity and tidal volume, the values in the volume-reduced group appear to be markedly elevated at week 3. Please clarify whether any specific physiological or experimental factors may explain this pronounced change at this time point.

The Discussion states that “the current findings in EMG activity timings may provide certain clues for the mechanism of these potential swallowing disorders.” However, the minipigs in the volume-reduction group did not exhibit overt swallowing disorders. Please clarify which specific changes in muscle activity are hypothesized to predispose to dysphagia and how these relate to subclinical swallowing impairment.

Please discuss which postoperative time period is considered the highest risk for swallowing dysfunction and airway compromise following tongue base reduction.

If obesity was induced systemically, is it possible that the soft palate also increased in volume? Please comment on whether changes in soft palate size or compliance may have contributed to the observed functional differences.

Reviewer #2: The authors evaluated tongue muscle function under two distinct conditions: obesity-induced tongue enlargement and surgically induced tongue base reduction, using an experimental framework established in the authors’ previous studies. The experimental methods themselves appear to be technically sound. However, there is a fundamental lack of logical consistency between the stated aims of the study (as presented in the title and throughout the manuscript) and the actual scope of the analyses.

The title and main text describe the objective as clarifying the effects of tongue base volume enlargement and reduction on tongue muscle function. In practice, the study examines responses under two highly specific and physiologically distinct conditions (obesity and surgical intervention). Because these conditions are inherently asymmetric, it is difficult to attribute the observed effects solely to changes in tongue volume as a geometric factor.

The rationale for the stated hypotheses also requires further clarification. The Introduction proposes that “a volume-reduced tongue base may induce swallowing disorders whereas a volume-enlarged tongue base may induce alterations in the respiratory dynamics during feeding”. While it is reasonable to expect that tongue volume changes influence both swallowing and respiration, the manuscript does not clearly explain why these effects are hypothesized to be function-specific in this asymmetric manner.

In addition, many of the reported results and corresponding discussions appear to reflect time-dependent changes associated with postoperative wound healing rather than effects of tongue volume alteration itself. Further clarification would be helpful regarding whether the authors intend to investigate the direct geometric effects of volume change, or to describe functional adaptations under these specific pathological conditions. Addressing this issue would require a fundamental redefinition of the study aims and overall structure, rather than clarification or reorganization of the existing manuscript.

**********

6. PLOS authors have the option to publish the peer review history of their article (what does this mean?). If published, this will include your full peer review and any attached files.

If you choose “no”, your identity will remain anonymous but your review may still be made public.

**Do you want your identity to be public for this peer review?**  For information about this choice, including consent withdrawal, please see our Privacy Policy.

Reviewer #1: No

Reviewer #2: No

**********

[NOTE: If reviewer comments were submitted as an attachment file, they will be attached to this email and accessible via the submission site. Please log into your account, locate the manuscript record, and check for the action link "View Attachments". If this link does not appear, there are no attachment files.]

To ensure your figures meet our technical requirements, please review our figure guidelines: https://journals.plos.org/plosone/s/figures

You may also use PLOS’s free figure tool, NAAS, to help you prepare publication quality figures: https://journals.plos.org/plosone/s/figures#loc-tools-for-figure-preparation.

NAAS will assess whether your figures meet our technical requirements by comparing each figure against our figure specifications.

# Author comment: Author response to Decision Letter 1

**DOI:** 10.1371/journal.pone.0352976.r003

4 Feb 2026

Point to point responses to journal requirements and reviewers’ comments

Journal Requirements:

When submitting your revision, we need you to address these additional requirements.

1. Please ensure that your manuscript meets PLOS ONE's style requirements, including those for file naming. The PLOS ONE style templates can be found at

https://journals.plos.org/plosone/s/file?id=wjVg/PLOSOne_formatting_sample_main_body.pdf and

https://journals.plos.org/plosone/s/file?id=ba62/PLOSOne_formatting_sample_title_authors_affiliations.pdf

Response: We appreciate the efficient revision process once the new Editor was assigned. We believe the revised version of the manuscript meets the style requirements.

2. Thank you for stating in your Funding Statement:

“This study was supported by the grant R01 DE028864 from NIH/NIDCR (Z.J.L.).”

Please provide an amended statement that declares *all* the funding or sources of support (whether external or internal to your organization) received during this study, as detailed online in our guide for authors at http://journals.plos.org/plosone/s/submit-now. Please also include the statement “There was no additional external funding received for this study.” in your updated Funding Statement.

Please include your amended Funding Statement within your cover letter. We will change the online submission form on your behalf.

Response: The suggested statement was added accordingly.

3. Thank you for stating the following financial disclosure:

“This study was supported by the grant R01 DE028864 from NIH/NIDCR (Z.J.L.).”

Please state what role the funders took in the study. If the funders had no role, please state: "The funders had no role in study design, data collection and analysis, decision to publish, or preparation of the manuscript."

If this statement is not correct you must amend it as needed.

Please include this amended Role of Funder statement in your cover letter; we will change the online submission form on your behalf.

Response: The suggested statement was added accordingly.

4. Thank you for stating the following in your Competing Interests section:

“Authors have no competing interests or other interests that might be perceived to influence the results and/or discussion reported in this paper.”

Please complete your Competing Interests on the online submission form to state any Competing Interests. If you have no competing interests, please state "The authors have declared that no competing interests exist.", as detailed online in our guide for authors at http://journals.plos.org/plosone/s/submit-now

This information should be included in your cover letter; we will change the online submission form on your behalf.

Response: The suggested statement was added accordingly in the corresponding section and cover letter.

5. In the online submission form, you indicated that [The data underlying the results presented in the study are available upon request].

All PLOS journals now require all data underlying the findings described in their manuscript to be freely available to other researchers, either 1. In a public repository, 2. Within the manuscript itself, or 3. Uploaded as supplementary information.

This policy applies to all data except where public deposition would breach compliance with the protocol approved by your research ethics board. If your data cannot be made publicly available for ethical or legal reasons (e.g., public availability would compromise patient privacy), please explain your reasons on resubmission and your exemption request will be escalated for approval.

Response: All datasets are available as supplementary information within the manuscript and attached to this submission.

6. When completing the data availability statement of the submission form, you indicated that you will make your data available on acceptance. We strongly recommend all authors decide on a data sharing plan before acceptance, as the process can be lengthy and hold up publication timelines. Please note that, though access restrictions are acceptable now, your entire data will need to be made freely accessible if your manuscript is accepted for publication. This policy applies to all data except where public deposition would breach compliance with the protocol approved by your research ethics board. If you are unable to adhere to our open data policy, please kindly revise your statement to explain your reasoning and we will seek the editor's input on an exemption. Please be assured that once you have provided your new statement, the assessment of your exemption will not hold up the peer review process.

Response: All datasets are available as supplementary information within the manuscript and attached to this submission.

7. We note that Figure 1 in your submission contain copyrighted image. All PLOS content is published under the Creative Commons Attribution License (CC BY 4.0), which means that the manuscript, images, and Supporting Information files will be freely available online, and any third party is permitted to access, download, copy, distribute, and use these materials in any way, even commercially, with proper attribution. For more information, see our copyright guidelines: http://journals.plos.org/plosone/s/licenses-and-copyright.

We require you to either (1) present written permission from the copyright holder to publish these figures specifically under the CC BY 4.0 license, or (2) remove the figures from your submission:

a. You may seek permission from the original copyright holder of Figure 1 to publish the content specifically under the CC BY 4.0 license.

We recommend that you contact the original copyright holder with the Content Permission Form (http://journals.plos.org/plosone/s/file?id=7c09/content-permission-form.pdf) and the following text:

“I request permission for the open-access journal PLOS ONE to publish XXX under the Creative Commons Attribution License (CCAL) CC BY 4.0 (http://creativecommons.org/licenses/by/4.0/). Please be aware that this license allows unrestricted use and distribution, even commercially, by third parties. Please reply and provide explicit written permission to publish XXX under a CC BY license and complete the attached form.”

Please upload the completed Content Permission Form or other proof of granted permissions as an "Other" file with your submission.

In the figure caption of the copyrighted figure, please include the following text: “Reprinted from [ref] under a CC BY license, with permission from [name of publisher], original copyright [original copyright year].”

b. If you are unable to obtain permission from the original copyright holder to publish these figures under the CC BY 4.0 license or if the copyright holder’s requirements are incompatible with the CC BY 4.0 license, please either i) remove the figure or ii) supply a replacement figure that complies with the CC BY 4.0 license. Please check copyright information on all replacement figures and update the figure caption with source information. If applicable, please specify in the figure caption text when a figure is similar but not identical to the original image and is therefore for illustrative purposes only.

Response: Figure 1C was edited to illustrate the cubic-shaped array of the 8 implanted SONO crystals in a dissected tongue specimen.

8. If the reviewer comments include a recommendation to cite specific previously published works, please review and evaluate these publications to determine whether they are relevant and should be cited. There is no requirement to cite these works unless the editor has indicated otherwise.

Response: We appreciate this advice.

Additional Editor Comments:

This manuscript evaluates how volumetric changes in the tongue base affect the coordination of chewing, swallowing, and respiration, addressing a topic of considerable academic importance and societal relevance within the medical field. In this respect, the study represents a meaningful and potentially valuable contribution.

However, based on the reviewers’ evaluations, the manuscript requires major revision before it can be considered for publication. The concerns raised extend beyond issues of clarity or presentation and involve substantive matters related to the conceptual framing, interpretation of results, and overall structure of the manuscript.

Accordingly, acceptance of this manuscript is contingent upon a comprehensive, point-by-point response to each of the reviewers’ comments and questions, accompanied by substantial revisions to the manuscript. The revised version should clearly document how each concern has been addressed. I encourage the authors to carefully consider the reviewers’ feedback and to submit a thoroughly revised manuscript for further evaluation.

Response: We appreciate the comments and we have proceeded accordingly.

Reviewers' comments:

Reviewer's Responses to Questions

Comments to the Author

1. Is the manuscript technically sound, and do the data support the conclusions?

The manuscript must describe a technically sound piece of scientific research with data that supports the conclusions. Experiments must have been conducted rigorously, with appropriate controls, replication, and sample sizes. The conclusions must be drawn appropriately based on the data presented.

Reviewer #1: Yes

Reviewer #2: No

________________________________________

2. Has the statistical analysis been performed appropriately and rigorously?

Reviewer #1: Yes

Reviewer #2: I Don't Know

________________________________________

3. Have the authors made all data underlying the findings in their manuscript fully available?

The PLOS Data policy requires authors to make all data underlying the findings described in their manuscript fully available without restriction, with rare exception (please refer to the Data Availability Statement in the manuscript PDF file). The data should be provided as part of the manuscript or its supporting information or deposited to a public repository. For example, in addition to summary statistics, the data points behind means, medians and variance measures should be available. If there are restrictions on publicly sharing data—e.g. participant privacy or use of data from a third party—those must be specified.

Reviewer #1: Yes

Reviewer #2: No

________________________________________

4. Is the manuscript presented in an intelligible fashion and written in standard English?

PLOS ONE does not copyedit accepted manuscripts, so the language in submitted articles must be clear, correct, and unambiguous. Any typographical or grammatical errors should be corrected at revision, so please note any specific errors here.

Reviewer #1: Yes

Reviewer #2: No

________________________________________

5. Review Comments to the Author

Please use the space provided to explain your answers to the questions above. You may also include additional comments for the author, including concerns about dual publication, research ethics, or publication ethics. (Please upload your review as an attachment if it exceeds 20,000 characters).

Reviewer #1

This study provides a mechanistically insightful analysis of how volumetric alterations of the tongue base affect the coordination of chewing, swallowing, and respiration. The combination of EMG, kinematic, and respiratory measurements in a longitudinal design is a major strength. In addition, the finding that both tongue volume reduction and enlargement induce functional adaptations in the timing and amplitude of muscle activity is particularly interesting. However, there are several concerns that should be addressed prior to publication.

Response: We appreciate these positive comments on our work.

Major points

-The manuscript compares changes in tongue kinematics associated with tongue volume enlargement and tongue volume reduction and discusses potential differences in their underlying mechanisms. However, the experimental procedures for these two conditions are fundamentally different. Tongue volume enlargement is induced gradually through obesity, whereas tongue volume reduction is achieved surgically, resulting in an abrupt decrease in tissue volume followed by postoperative adaptation. These two approaches may probe different physiological responses (chronic adaptation vs. acute injury and recovery). Please clarify whether direct comparisons between these two conditions are appropriate and discuss this limitation more explicitly.

Response: We appreciate that the reviewer indicated this important drawback of these two different minipig models. This difference (acute injury/wound healing vs chronic adaptation to obesity) was noted in the section of Materials and Methods, and further discussed in the section of Discussion.

-The volume-enlarged group is assumed to represent tongue base enlargement due to obesity. However, longitudinal measurements of tongue base volume were not provided. Please clarify whether tongue base volume was measured at baseline and at subsequent time points, or whether the reported values represent terminal measurements only.

Response: Since the measurements of weights and volumes of the tongue were not available in living animals. The data provided are postmortem measurements. We also provided the postmortem measurements of the control group published previously. However, it must be noted that these control animals were 5 weeks younger than those in the present study.

-The manuscript provides little discussion of anatomical differences between minipigs and humans with respect to tongue base muscle composition, fat distribution, and pharyngeal morphology. For translational relevance, it would be important to clearly state which anatomical features are comparable and which differ between the two species.

Response: We appreciate this comment. The detailed comparison of the tongue and oropharyngeal structures between minipigs and humas have been added in the section of Discussion.

-Please clarify why week 5 was chosen as the final observation time point. In particular, for the tongue reduction group, it is possible that this time point still represents an intermediate stage of recovery rather than full functional stabilization.

Response: The period of 5 weeks was chosen because our previous study on the surgical volumetric reduction of the tongue body showed complete would healing at 4 weeks in the same minipig model. Yes, we agree that longer time period could provide further insights into fibrosis formation, muscle regeneration and remodeling, and functional recovery/compensation. However, given the limited life span of the implanted SONO crystals and leading cables (skin button) and potential risk of continuous obesity on animals’ welfare, the 5-week experimental period was decided. This rationale was added to the section of Materials and Methods.

Minor points

-At abstract, conclusions could be stated more concretely. The current conclusion is too broad.

Response: The conclusion in Abstract was revised accordingly.

-Given the interdisciplinary of PLOS ONE, it would be helpful to include a schematic figure early in the manuscript illustrating the anatomical locations and principal fiber orientations of the recorded muscles (DA, MA, SG, GG, LVP, TVP, TH, MC) in the minipig.

Response: We appreciate this comment. However, given the multiple figures are already included in this MS and the detailed anatomy of these muscles can be easily found in various social media, these additions may not be necessary.

-The description of the respiratory results could be made more concise and more explicitly linked to the figures. In particular, when comparing volume reduction and enlargement, it is important to ensure that identical time points and experimental conditions are being compared.

Response: Thank you for this comment. We do not fully understand the actual meaning of “The description of the respiratory results could be made more concise and more explicitly linked to the figures”. Yes, the data from identical time points and experimental conditions were compared. The relevant description was revised to reflect these comparisons.

-For airflow velocity and tidal volume, the values in the volume-reduced g

Attachment Submitted filename: Point to point response.docx

# Peer review: Decision Letter 1

**DOI:** 10.1371/journal.pone.0352976.r004

**Authors:** Kazunori Nozaki

23 Feb 2026

PONE-D-25-49745R1

Effects of Volumetric Enlargement or Reduction of the Tongue Base on Chewing and Swallowing

PLOS One

Dear Dr. Liu,

Thank you for submitting your manuscript to PLOS ONE. After careful consideration, we have decided that your manuscript does not meet our criteria for publication and must therefore be rejected.

Specifically:

The authors did not provide a logical and sufficiently reasoned response to the concerns raised by Reviewer 2. The rebuttal lacks a clear explanation addressing the specific methodological and conceptual issues that were pointed out. As a result, the fundamental concerns remain unresolved.

I am sorry that we cannot be more positive on this occasion, but hope that you appreciate the reasons for this decision.

Kind regards,

Kazunori Nozaki, DDS,PhD,PhD

Academic Editor

PLOS One

[Note: HTML markup is below. Please do not edit.]

Reviewers' comments:

Reviewer's Responses to Questions

**Comments to the Author**

1. If the authors have adequately addressed your comments raised in a previous round of review and you feel that this manuscript is now acceptable for publication, you may indicate that here to bypass the “Comments to the Author” section, enter your conflict of interest statement in the “Confidential to Editor” section, and submit your "Accept" recommendation.

Reviewer #1: All comments have been addressed

Reviewer #2: All comments have been addressed

**********

2. Is the manuscript technically sound, and do the data support the conclusions?

The manuscript must describe a technically sound piece of scientific research with data that supports the conclusions. Experiments must have been conducted rigorously, with appropriate controls, replication, and sample sizes. The conclusions must be drawn appropriately based on the data presented.

Reviewer #1: (No Response)

Reviewer #2: Yes

**********

3. Has the statistical analysis been performed appropriately and rigorously?

Reviewer #1: (No Response)

Reviewer #2: I Don't Know

**********

4. Have the authors made all data underlying the findings in their manuscript fully available?

The PLOS Data policy requires authors to make all data underlying the findings described in their manuscript fully available without restriction, with rare exception (please refer to the Data Availability Statement in the manuscript PDF file). The data should be provided as part of the manuscript or its supporting information, or deposited to a public repository. For example, in addition to summary statistics, the data points behind means, medians and variance measures should be available. If there are restrictions on publicly sharing data—e.g. participant privacy or use of data from a third party—those must be specified.

Reviewer #1: (No Response)

Reviewer #2: Yes

**********

5. Is the manuscript presented in an intelligible fashion and written in standard English?

PLOS ONE does not copyedit accepted manuscripts, so the language in submitted articles must be clear, correct, and unambiguous. Any typographical or grammatical errors should be corrected at revision, so please note any specific errors here.

Reviewer #1: (No Response)

Reviewer #2: Yes

**********

6. Review Comments to the Author

Please use the space provided to explain your answers to the questions above. You may also include additional comments for the author, including concerns about dual publication, research ethics, or publication ethics. (Please upload your review as an attachment if it exceeds 20,000 characters)

Reviewer #1: (No Response)

Reviewer #2: Thank you for your response. However, my concern is primarily about the overall logical framework of the study, whereas the authors’ reply mainly emphasizes the validity of the measurements and the clinical relevance of the models. As a result, the response does not fully address the point being raised.

1. The authors’ response explains the clinical relevance of the models and provides details on the measurements, but it does not address the key issue in this review regarding the logical validity of the comparison axis. If the authors acknowledge the asymmetry between the obesity model and the post-surgical model, then at least one of the following would be needed: (1) to redefine the study as a comparison of clinically relevant pathological models rather than as a test of the pure effect of volume change; or (2) to provide a clearer theoretical justification for using volume as the primary comparison axis, together with an explicit statement of the limitations of that interpretation.

2. Related to the above, the authors acknowledge the possible influence of wound healing, yet conclude that the study evaluated volume-related functional alterations rather than postoperative responses on the basis that volume reduction was confirmed. However, confirmation of a morphological change does not demonstrate that volume change is the primary driver of the observed functional changes. There remains a logical leap in this causal interpretation.

**********

7. PLOS authors have the option to publish the peer review history of their article (what does this mean?). If published, this will include your full peer review and any attached files.

If you choose “no”, your identity will remain anonymous but your review may still be made public.

**Do you want your identity to be public for this peer review?**  For information about this choice, including consent withdrawal, please see our Privacy Policy.

Reviewer #1: No

Reviewer #2: No

**********

[NOTE: If reviewer comments were submitted as an attachment file, they will be attached to this email and accessible via the submission site. Please log into your account, locate the manuscript record, and check for the action link "View Attachments". If this link does not appear, there are no attachment files.]

- - - - -

For journal use only: PONEDEC3

# Author comment: Author response to Decision Letter 2

**DOI:** 10.1371/journal.pone.0352976.r005

2 Apr 2026

Point by point responses to the comments from editor and reviewer #2.

From the academic editor:

Thank you for submitting your manuscript to PLOS ONE. After careful consideration, we have decided that your manuscript does not meet our criteria for publication and must therefore be rejected.

Specifically:

The authors did not provide a logical and sufficiently reasoned response to the concerns raised by Reviewer 2. The rebuttal lacks a clear explanation addressing the specific methodological and conceptual issues that were pointed out. As a result, the fundamental concerns remain unresolved.

Response: To the Question #1 asking, “If the authors have adequately addressed your comments raised in a previous round of review and you feel that this manuscript is now acceptable for publication,…” reviewer #2 answered “YES” affirmatively, and also answered “YES” affirmatively to the Questions # 2, 4, and 5 to indicate 1) the manuscript technically sound, and the data support the conclusions; 2) the authors made all data underlying the findings in their manuscript fully available; and 3) the manuscript presented in an intelligible fashion and written in standard English. In the Question #6 of Review Comments to the Author, reviewer #2 requested that we simply needed to “1) redefine the study as a comparison of clinically relevant pathological models rather than as a test of the pure effect of volume change, and 2) to provide a clearer theoretical justification for using volume as the primary comparison axis, together with an explicit statement of the limitations of that interpretation."' We believe we did those in the last revision. We realized that our explanations might not be quite clear enough, so we are now responding to these comments more clearly particularly in terms of the conceptual framework and the limitation of the present study.

From Reviewer #2:

Thank you for your response. However, my concern is primarily about the overall logical framework of the study, whereas the authors’ reply mainly emphasizes the validity of the measurements and the clinical relevance of the models. As a result, the response does not fully address the point being raised.

Responses: In addition to the validity of the measurements and the clinical relevance of the models, we are now stating the clear conceptual framework logically in this revision (please see below).

1. The authors’ response explains the clinical relevance of the models and provides details on the measurements, but it does not address the key issue in this review regarding the logical validity of the comparison axis. If the authors acknowledge the asymmetry between the obesity model and the post-surgical model, then at least one of the following would be needed: (1) to redefine the study as a comparison of clinically relevant pathological models rather than as a test of the pure effect of volume change; or (2) to provide a clearer theoretical justification for using volume as the primary comparison axis, together with an explicit statement of the limitations of that interpretation.

Response: We really appreciate this very important point. It is absolutely right that the study is about the comparisons between the two clinically relevant pathological models rather than their pure symmetric effects of volumetric alterations of the tongue base. The structure or diagram of the conceptual vs theoretical framework in the present study are as follows: Theoretical: the tongue base size/volume affects oropharyngeal function, including respiration, chewing, swallowing and others; Conceptual: volume enlarged – obese minipigs with enlarged tongue base as confirmed by multiple animal and clinical studies (Baker et al. 2025; Brennick et al. 2009; Brennick et al. 2006; Isono 2011; Kim et al. 2014); volume reduced – normal weighted minipigs with surgical volume reduction of the tongue base as widely applied clinically (Li et al. 2016). These two models have high clinical relevance but are not symmetric as responded in the last revision. Therefore, despite the observed effects in the late time point (weeks 3 and 5) of reduction animals might mainly result from the proposed intervention (surgical volumetric reduction), these effects in obese animals (enlargement) might also result from enlargements of other oropharyngeal structures, such as tongue body, soft palate, lateral pharyngeal fat pads, etc. These confounding factors have been incorporated in this revision. In addition, due to the surgical injury in the volumetric reduction group and wound healing lasting usually two weeks which are based upon the data from similar model of volumetric reduction of the tongue body in our previously published study (Perkins et al. 2008; Shcherbatyy et al. 2008), the observed effects at early time point of week 1 may be confounded by the wound healing process as well. That said that acute surgical impact is also important to understand in its own right in order to appreciate the acute (not just chronic) impacts of surgery. All these confounding factors and limitations of the study have been comprehensively discussed in this revision. Additionally, text was added in the introduction section explaining the theoretical justification of these two clinically relevant models.

2. Related to the above, the authors acknowledge the possible influence of wound healing, yet conclude that the study evaluated volume-related functional alterations rather than postoperative responses on the basis that volume reduction was confirmed. However, confirmation of a morphological change does not demonstrate that volume change is the primary driver of the observed functional changes. There remains a logical leap in this causal interpretation.

Response: It is absolutely right that postoperative responses might have confounded the results in the reduction group at early time point as responded above. Therefore, we emphasized in this revision that 1) functional changes of reduction animals in EMG at early time point might not be driven by the volume reduction; and 2) functional changes of enlargement (obese) animals in EMG might also be driven by increased size/volume of other oropharyngeal structures. Nevertheless, the observed changes in both clinical pathological conditions are mainly attributed to the induced volumetric changes of the tongue base. These consequences are mostly observed in patients with either one or both alterations and progressively showing functional impairment in respiration and mastication (chewing and swallowing) with the alterations in oropharyngeal muscle activity. Thus, the present study may provide some insights into the mechanism of these oropharyngeal functional disorders due to the volumetric changes in the tongue base.

References

Baker E, Chanamolu M, Nieri C, White SF, Brandt J, Gillespie MB. 2025. The effect of tongue volume and adipose content on obstructive sleep apnea: Meta-analysis & systematic review. OTO Open. 9(2):e70067.

Brennick MJ, Pack AI, Ko K, Kim E, Pickup S, Maislin G, Schwab RJ. 2009. Altered upper airway and soft tissue structures in the new zealand obese mouse. Am J Respir Crit Care Med. 179(2):158-169.

Brennick MJ, Pickup S, Cater JR, Kuna ST. 2006. Phasic respiratory pharyngeal mechanics by magnetic resonance imaging in lean and obese zucker rats. Am J Respir Crit Care Med. 173(9):1031-1037.

Isono S. 2011. Obesity and obstructive sleep apnoea: Mechanisms for increased collapsibility of the passive pharyngeal airway. Respirology. 17(1):32-42.

Kim AM, Keenan BT, Jackson N, Chan EL, Staley B, Poptani H, Torigian DA, Pack AI, Schwab RJ. 2014. Tongue fat and its relationship to obstructive sleep apnea. Sleep. 37(10):1639-1648.

Li HY, Lee LA, Kezirian EJ. 2016. Coblation endoscopic lingual lightening (cell) for obstructive sleep apnea. Eur Arch Otorhinolaryngol. 273(1):231-236.

Perkins JA, Shcherbatyy V, Liu ZJ. 2008. Morphologic and histologic outcomes of tongue reduction surgery in an animal model. Otolaryngol Head Neck Surg. 139(2):291-297.

Shcherbatyy V, Perkins JA, Liu ZJ. 2008. Internal kinematics of the tongue following volume reduction. Anat Rec (Hoboken). 291(7):886-893.

Attachment Submitted filename: PLOS ONE Responses - final version.docx

# Peer review: Decision Letter 2

**DOI:** 10.1371/journal.pone.0352976.r006

**Authors:** Ayako Mochizuki

18 Jun 2026

Muscle Activity and Airflow Dynamics upon Gradual Weight-Gain Volumetric Enlargement or Acute Surgical Reduction of the Tongue Base during Chewing and Swallowing

PONE-D-25-49745R2

Dear Dr. Zi-Jun Liu,

We’re pleased to inform you that your manuscript has been judged scientifically suitable for publication and will be formally accepted for publication once it meets all outstanding technical requirements.

Within one week, you’ll receive an e-mail detailing the required amendments. When these have been addressed, you’ll receive a formal acceptance letter and your manuscript will be scheduled for publication.

An invoice will be generated when your article is formally accepted. Please note, if your institution has a publishing partnership with PLOS and your article meets the relevant criteria, all or part of your publication costs will be covered. Please make sure your user information is up-to-date by logging into Editorial Manager at Editorial Manager® and clicking the ‘Update My Information' link at the top of the page. For questions related to billing, please contact billing support.

If your institution or institutions have a press office, please notify them about your upcoming paper to help maximize its impact. If they’ll be preparing press materials, please inform our press team as soon as possible -- no later than 48 hours after receiving the formal acceptance. Your manuscript will remain under strict press embargo until 2 pm Eastern Time on the date of publication. For more information, please contact onepress@plos.org.

Kind regards,

Ayako Mochizuki

Academic Editor

PLOS One

Additional Editor Comments (optional):

The authors have responded to and incorporated the feedback received from multiple reviewers with a reasonable degree of sincerity, and the manuscript has been improved to a satisfactory level. No further comments.

Reviewers' comments:

Reviewer's Responses to Questions

**Comments to the Author**

1. If the authors have adequately addressed your comments raised in a previous round of review and you feel that this manuscript is now acceptable for publication, you may indicate that here to bypass the “Comments to the Author” section, enter your conflict of interest statement in the “Confidential to Editor” section, and submit your "Accept" recommendation.

Reviewer #2: (No Response)

**********

2. Is the manuscript technically sound, and do the data support the conclusions?

The manuscript must describe a technically sound piece of scientific research with data that supports the conclusions. Experiments must have been conducted rigorously, with appropriate controls, replication, and sample sizes. The conclusions must be drawn appropriately based on the data presented.

Reviewer #2: (No Response)

**********

3. Has the statistical analysis been performed appropriately and rigorously?

Reviewer #2: (No Response)

**********

4. Have the authors made all data underlying the findings in their manuscript fully available?

The PLOS Data policy requires authors to make all data underlying the findings described in their manuscript fully available without restriction, with rare exception (please refer to the Data Availability Statement in the manuscript PDF file). The data should be provided as part of the manuscript or its supporting information, or deposited to a public repository. For example, in addition to summary statistics, the data points behind means, medians and variance measures should be available. If there are restrictions on publicly sharing data—e.g. participant privacy or use of data from a third party—those must be specified.

Reviewer #2: (No Response)

**********

5. Is the manuscript presented in an intelligible fashion and written in standard English?

PLOS ONE does not copyedit accepted manuscripts, so the language in submitted articles must be clear, correct, and unambiguous. Any typographical or grammatical errors should be corrected at revision, so please note any specific errors here.

Reviewer #2: (No Response)

**********

6. Review Comments to the Author

Please use the space provided to explain your answers to the questions above. You may also include additional comments for the author, including concerns about dual publication, research ethics, or publication ethics. (Please upload your review as an attachment if it exceeds 20,000 characters)

Reviewer #2: (No Response)

**********

7. PLOS authors have the option to publish the peer review history of their article (what does this mean?). If published, this will include your full peer review and any attached files.

If you choose “no”, your identity will remain anonymous but your review may still be made public.

**Do you want your identity to be public for this peer review?**  For information about this choice, including consent withdrawal, please see our Privacy Policy.

Reviewer #2: No

**********

# Editor report: Acceptance letter

**DOI:** 10.1371/journal.pone.0352976.r007

**Authors:** Ayako Mochizuki

PONE-D-25-49745R2

PLOS One

Dear Dr. Rosero Salazar,

I'm pleased to inform you that your manuscript has been deemed suitable for publication in PLOS One. Congratulations! Your manuscript is now being handed over to our production team.

At this stage, our production department will prepare your paper for publication. This includes ensuring the following:

* All references, tables, and figures are properly cited

* All relevant supporting information is included in the manuscript submission,

* There are no issues that prevent the paper from being properly typeset

You will receive further instructions from the production team, including instructions on how to review your proof when it is ready. Please keep in mind that we are working through a large volume of accepted articles, so please give us a few days to review your paper and let you know the next and final steps.

Lastly, if your institution or institutions have a press office, please let them know about your upcoming paper now to help maximize its impact. If they'll be preparing press materials, please inform our press team within the next 48 hours. Your manuscript will remain under strict press embargo until 2 pm Eastern Time on the date of publication. For more information, please contact onepress@plos.org.

You will receive an invoice from PLOS for your publication fee after your manuscript has reached the completed accept phase. If you receive an email requesting payment before acceptance or for any other service, this may be a phishing scheme. Learn how to identify phishing emails and protect your accounts at https://explore.plos.org/phishing.

If we can help with anything else, please email us at customercare@plos.org.

Thank you for submitting your work to PLOS ONE and supporting open access.

Kind regards,

PLOS ONE Editorial Office Staff

on behalf of

Dr. Ayako Mochizuki

Academic Editor

PLOS One
